# A novel bioinformatics pipeline to discover genes related to arbuscular mycorrhizal symbiosis based on their evolutionary conservation pattern among higher plants

**DOI:** 10.1186/s12870-014-0333-0

**Published:** 2014-12-03

**Authors:** Patrick Favre, Laure Bapaume, Eligio Bossolini, Mauro Delorenzi, Laurent Falquet, Didier Reinhardt

**Affiliations:** Department of Biology, University of Fribourg, Fribourg, Switzerland; Ludwig Center for Cancer Research, University of Lausanne, Lausanne, Switzerland; Swiss Institute of Bioinformatics, Fribourg, Switzerland; Oncology Department, University of Lausanne, Lausanne, Switzerland; SIB Swiss Institute of Bioinformatics, Lausanne, Switzerland; Current address: Crop Genetics, Bayer CropScience NV, Ghent, Belgium

**Keywords:** Arbuscular mycorrhiza, Symbiosis, Symbiosis signaling, Common SYM gene, Conservation, Gene clustering, Proteome analysis, Bioinformatics

## Abstract

**Background:**

Genes involved in arbuscular mycorrhizal (AM) symbiosis have been identified primarily by mutant screens, followed by identification of the mutated genes (forward genetics). In addition, a number of AM-related genes has been identified by their AM-related expression patterns, and their function has subsequently been elucidated by knock-down or knock-out approaches (reverse genetics). However, genes that are members of functionally redundant gene families, or genes that have a vital function and therefore result in lethal mutant phenotypes, are difficult to identify. If such genes are constitutively expressed and therefore escape differential expression analyses, they remain elusive. The goal of this study was to systematically search for AM-related genes with a bioinformatics strategy that is insensitive to these problems. The central element of our approach is based on the fact that many AM-related genes are conserved only among AM-competent species.

**Results:**

Our approach involves genome-wide comparisons at the proteome level of AM-competent host species with non-mycorrhizal species. Using a clustering method we first established orthologous/paralogous relationships and subsequently identified protein clusters that contain members only of the AM-competent species. Proteins of these clusters were then analyzed in an extended set of 16 plant species and ranked based on their relatedness among AM-competent monocot and dicot species, relative to non-mycorrhizal species. In addition, we combined the information on the protein-coding sequence with gene expression data and with promoter analysis. As a result we present a list of yet uncharacterized proteins that show a strongly AM-related pattern of sequence conservation, indicating that the respective genes may have been under selection for a function in AM. Among the top candidates are three genes that encode a small family of similar receptor-like kinases that are related to the S-locus receptor kinases involved in sporophytic self-incompatibility.

**Conclusions:**

We present a new systematic strategy of gene discovery based on conservation of the protein-coding sequence that complements classical forward and reverse genetics. This strategy can be applied to diverse other biological phenomena if species with established genome sequences fall into distinguished groups that differ in a defined functional trait of interest.

**Electronic supplementary material:**

The online version of this article (doi:10.1186/s12870-014-0333-0) contains supplementary material, which is available to authorized users.

## Background

Most land plants engage in symbiotic associations with fungi (*Glomeromycota*) that colonize their roots and provide them with phosphate and other mineral nutrients [1]. This association, referred to as arbuscular mycorrhiza (AM), is found in most major taxa of land plants [2], and is thought to have emerged monophyletically in an early progenitor of the vascular plants [3]. The strongest argument for this assumption is the fact that mycorrhizal development requires a conserved signalling pathway that consists of approximately 10 genes that encode receptor components such as SYMRK, and signalling intermediates such as CCaMK [4]. The genes involved in this pathway are conserved between monocots and dicots, and occur also in lycopods and mosses [5,6], suggesting that the origin of AM dates back to the early vascular plants at the time when land became colonized [3]. This assumption is consistent with the fossil record, which provides evidence for AM-like associations in the sediments of the Rhynie chert that is estimated to originate from the Ordovician period around 450 My ago [7]. More than 350 My after the evolution of AM, a subsequent event in a small subset of the dicots (Fabales, Fagales, Cucurbitales, Rosales), allowed for the emergence of a new form of symbiosis, root nodule symbiosis (RNS) with rhizobacteria [8-10]. Interestingly, RNS, as well as the actinorrhizal symbiosis with cyanobacterial endosymbionts [11,12], involve the same signalling pathway as AM, which therefore is referred to as common symbiosis signalling pathway (common SYM pathway) [4,13]. A central element of the common SYM pathway is calcium spiking, a rythmic change in perinuclear calcium concentration, which is perceived and transmitted by calcium and calmodulin-dependent protein kinase (CCaMK) to induce symbiotic gene expression [14,15].

AM is formed by more than 80% of the vascular plants [1], indicating that this association provides a significant selective advantage over non-mycorrhizal plants. However, some plant taxa do not form AM, among them the *Brassicaceae* with the best-characterized model plant species *Arabidopsis thaliana*, and the *Chenopodiaceae* with the economically important crop species *Beta vulgaris* (sugar beet). AM-related genes are often conserved among AM-competent plant species, while they are less conserved or even missing in non-mycorrhizal species. This phenomenon has been described for *VAPYRIN* (VPY), which is essential for infection and development of the fungal feeding structures, the arbuscules, in *Petunia hybrida* as well as in *Medicago truncatula* [16,17]. VPY is entirely missing from non-mycorrhizal plant species [16-18], and the same is true for numerous genes that are expressed specifically in AM [19,20]. Such a pattern of conservation was also observed in AM-related genes that are members of large ubiquitous gene families such as the ABC transporters STUNTED ARBUSCULE (STR) and STR2, or the GRAS-type transcription factor REQUIRED FOR ARBUSCULAR MYCORRHIZA1 (RAM1), which both belong to subfamilies which are restricted to AM-competent plant species [21,22]. Similarly, several components of the common SYM pathway are missing from the non-mycorrhizal model species *Arabidopsis thaliana* [6], whereas they are conserved among AM-competent dicots and monocots.

Traditionally, AM-related genes have been identified either by mutant screenings followed by characterization of the mutated gene (forward genetics), or by transcript profiling, followed by mutational analysis of AM-induced genes (reverse genetics). Considering the increasing number of sequenced plant genomes, the loss of AM-related genes from the genomes of non-mycorrhizal species could serve as a criterion to detect new AM-related genes by comparative genomics. This third way of gene discovery could potentially identify AM-related genes that have escaped characterization via traditional genetic approaches because of functional redundancy, lethal phenotypes, or constitutive gene expression. Conceptually, such an approach represents a substractive procedure where the proteomes of non-mycorrhizal plants such as *A. thaliana* are substracted from a panel of proteomes of AM-competent species to result in a set of proteins that are consistently conserved among AM-competent plants and absent from non-mycorrhizal reference species.

Here, we describe a novel approach to identify new AM-related genes based on genome substraction. The approach consists of a multistep procedure that uses protein sequence conservation and gene expression as criteria for the enrichment of potential symbiosis-related genes. We have compared a set of six AM-competent angiosperm species (*Solanum lycopersicum, Solanum tuberosum, Vitis vinifera, Medicago truncatula, Glycine max, Populus trichocarpa*), and three non-AM species (*Arabidopsis thaliana, Arabidopsis lyrata, and Brassica rapa*) with an initial clustering approach that resulted in a set of potential candidate proteins comprising approximately 10% of the entire proteome. At a second step, a clustering of these genes based on gene expression patterns in *Medicago truncatula* provided a set of conserved genes that are induced in AM. Finally, to focus on conserved constitutively expressed genes (such as the common SYM genes), a proteome blast of the conserved genes to an extended panel of proteomes (including monocots) allowed to perform quantitative statistics on the E-values, hence providing a set of proteins that are significantly more conserved among AM-competent plant species than towards the non-mycorrhizal *Brassicaceae*. This strategy was validated with a number of known AM-related genes that passed our selection scheme. The resulting list of predicted AM-related proteins will be functionally tested by reverse genetic approaches.

## Results

### Patterns of sequence conservation in AM-related genes

**Figure 1:**
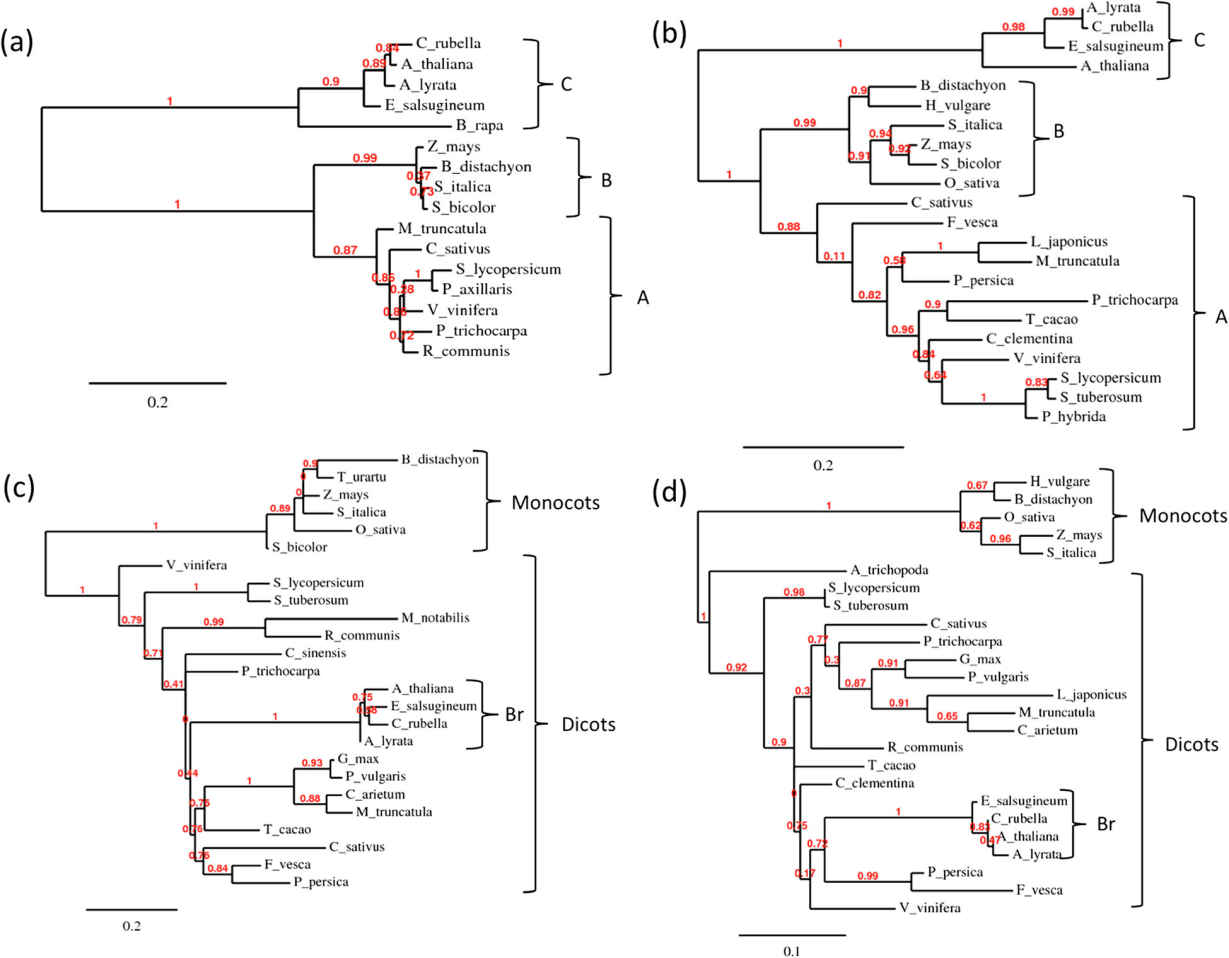
**Phylogenetic analysis of AM-related genes relative to house-keeping genes.** Phylograms of AM-induced RAM1 **(a)** and PT4 **(b)**, in relation to the housekeeping genes cyclin D6 **(c)** and RPL5 **(d)**.

In order to explore the potential for differential conservation of AM-related genes, we established the phylogeny of two central AM-related proteins, the GRAS-type transcription factor REQUIRED FOR ARBUSCULAR MYCORRHIZA1 (RAM1), which is an essential regulator of AM symbiosis [21], and PHOSPHATE TRANSPORTER4 (PT4), which is required for symbiotic phosphate transfer and arbuscule functioning [23,24] (Additional file 1: File S1). A phylogenetic tree of RAM1 shows a clear bisection between mycorrhizal plants (group A and B), and non-mycorrhizal plants (group C) (Figure 1a). Notably, the sequences of AM-competent dicots and monocots (groups A and B) grouped significantly closer together than the dicots among each other (groups A and C). A similar pattern was observed in a phylogenetic tree of PT4 and its closest homologues in various monocot and dicot species (Figure 1b). As with RAM1, the homologues from AM-competent dicots (group A) and monocots (group B) grouped more closely together than the homologues of the phylogenetically related groups of the AM-competent dicots (group A) and the non-mycorrhizal dicots (group C).

A contrasting pattern was observed when two housekeeping genes were analysed, that encode cyclin D6 and chloroplast ribosomal protein L5 (Figure 1c and d). These proteins showed a pattern of conservation that reflects the closer relationship among the dicots, relative to the monocots, which clustered separately from all the dicot species (Figure 1c and d). Taken together, this evidence suggests that PT4 and RAM1, and perhaps other AM-related genes, are under diversifying selection in AM-competent species. Hence, AM-related genes could potentially be identified based on the relative conservation pattern of their encoded proteins.

### Hierarchical clustering to identify protein phylogeny

**Figure 2:**
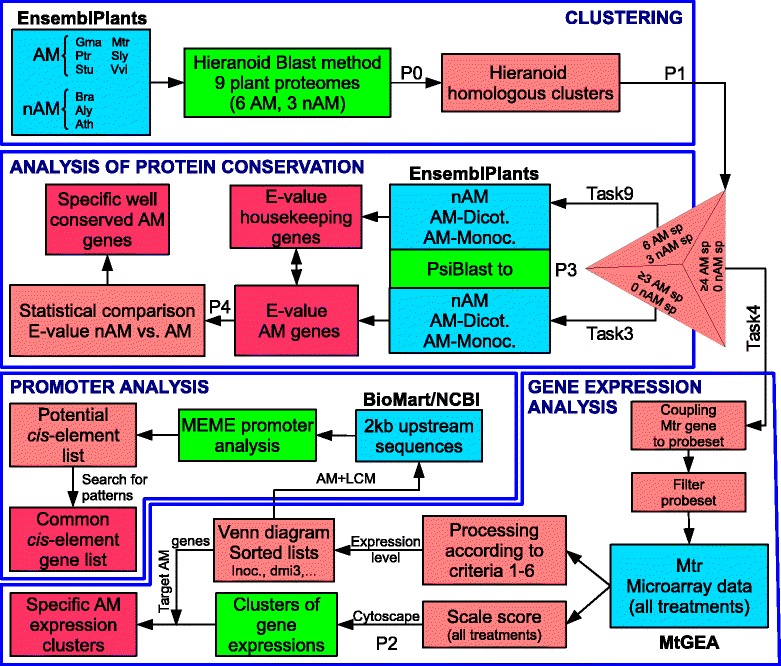
**Strategy used to identify AM-related genes based on sequence conservation.** The flow chart reflects the stepwise identification of potential AM-related proteins based on their pattern of sequence conservation at the protein level, the pattern of gene expression, and predicted regulatory elements in their promoters. Sp: Plant species. P0-P4 correspond to protocols, files, or scripts provided in supplementary materials (Additional file 4: File S3). Blue boxes: Databases; green boxes: Tools and processes; pink boxes: intermediate outputs; red boxes: final outputs.

In order to identify AM-related genes in a systematic way, we first applied the clustering software Hieranoid [25] to the proteome sequences of six AM-competent species, namely *Medicago truncatula* (Mtr), *Glycine max* (Gma), *Vitis vinifera* (Vvi), *Solanum lycopersicum* (Sly), *Solanum tuberosum* (Stu) and *Populus trichocarpa* (Ptr), and three non-mycorrhizal species, namely *Arabidopsis thaliana* (Ath), *Arabidopsis lyrata* (Aly), and *Brassica rapa* (Bra). Pairwise clustering proceeded based on a conceptual phylogenetic tree of the involved plant species (Additional file 2: Figure S1; Additional file 3: File S2, script P0_HieraProcedure.txt). Figure 2 describes the work-flow of our strategy. Briefly, the proteomes of 9 species were used for clustering. The resulting trees of orthologous/paralogous proteins were filtered to yield lists of protein clusters that satisfied certain defined criteria referred to as Task3, Task4, Task9 (see next section). These gene lists were further processed to isolate AM-related genes based on gene expression, on conservation of the protein-coding region, and on the promoter sequence, as discussed in detail in the following sections. Scripts involved in the different processes (P0, P1, P2, P3, and P4 in Figure 2) are provided in Additional file 3: File S2.

After an initial round of clustering, we noticed that, unexpectedly, the known AM-related VAPYRIN protein was not found among the trees generated by Hieranoid, although it is believed to generally occur in all AM-competent plants [17,26], whereas it is absent from the *Brassicaceae* [16,18]. Closer inspection of the Mtr proteome from ENSEMBL revealed that VAPYRIN, as well as the two AM-related genes CASTOR and PT4 were missing from the *M. truncatula* proteome (database: Ensembl Plants release 20). However, they could be identified in the UniProtKB database [27] and were added to the Mtr proteome (VAPYRIN: Mtr_D3J162; CASTOR: Mtr_D6C5X5; PT4: Mtr_AAM76743.1), and Hieranoid was restarted. Final clustering from the proteomes of the 9 plant species gave rise to a total of 28’528 clusters of orthologous and/or paralogous proteins (Figure 2, Additional file 4: File S3).

### Selecting clusters with potential AM-related proteins

In order to select among all the clusters generated by Hieranoid those that showed the conservation pattern known for AM-related proteins (Figure 1a,b), we performed *in silico* substraction by selecting protein clusters that contained at least one protein of each of the 6 AM-competent species, but none of the non-mycorrhizal species Ath, Aly, and Bra (Task6). In order to account for cases where an individual protein may be missing because of an incomplete proteome (as observed in *M. truncatula* for VAPYRIN, CASTOR and PT4; see above), we also performed a more permissive search for clusters that contained proteins of at least 5 of the 6 AM-competent species, but none from the three non-mycorrhizal species (Task5), and we also carried out the corresponding subtractions with Task4 and Task3 (Figure 2, P1; see P1_hieranoid_output_treatments.sh in Additional file 3: File S2).

The numbers of clusters passing through these filters are listed in Table 1. In all filtering trials, *M. truncatula* showed considerably lower numbers than the other species, indicating that its proteome is less complete than those of the other AM-competent species (Table 1). Hence, to account for the incompleteness of the *M. truncatula* proteome, and for other potentially missing proteins, we selected the clusters identified by Task4 for further analysis.

**Table 1:** **Number of protein clusters from hierarchical clustering after filtering**

	**Task3**	**Task4**	**Task5**	**Task6**
Number of clusters	4438	2327	1184	479
Number of clusters with Sly	3238 (73%)	1986 (85%)	1150 (97%)	479 (100%)
Number of clusters with Stu	3205 (72%)	1959 (84%)	1137 (96%)	479 (100%)
Number of clusters with Vvi	3138 (71%)	1967 (85%)	1057 (89%)	479 (100%)
Number of clusters with Gma	2971 (67%)	1934 (83%)	1059 (89%)	479 (100%)
Number of clusters with Ptr	2703 (61%)	1763 (76%)	1029 (87%)	479 (100%)
Number of clusters with Mtr	2049 (46%)	1362 (59%)	867 (73%)	479 (100%)
Number of Mtr genes	3203	2117	1338	717
Number of Mtr genes with at least one MtGEA probeset	2373	1618	1028	563
Number of clusters with at least one MtGEA probeset	1637	1109	708	392

Among the total of 28’528 clusters obtained from Hieranoid, those were selected that did not have a member from the Brassicaceae (*A. thaliana*, *A. lyrata*, *B. rapa*), but had at least one hit from at least 3, 4, 5, or 6 of the AM-competent species, respectively. These are referred to as Task3, Task4, Task5 and Task6, respectively. In addition, the occurrence of the 6 AM-competent species in the respective clusters is indicated. Information of the relative representation of genes from *M. truncatula* (Mtr) in the *Medicago* gene Atlas is provided as well.

In order to assess the efficiency of Hieranoid clustering and subsequent filtering, we tested whether known AM-related genes passed the selection process. We assembled a list of 23 proteins with known function and/or expression pattern in AM (Additional file 5: Table S1, Additional file 6: File S4). They are known either as components of the common SYM signalling pathway (gene 1-9), as receptor of nod factor and potentially myc factor (NFP), as genes required specifically in AM development (RAM1, RAM2, STR), or as genes specifically induced in mycorrhizal roots (genes 14-19). An additional set of proteins served as negative controls that were not expected to pass the filter, either because they function as housekeeping genes and are therefore ubiquitous (PT1, PT6), or because they are primarily involved in nodulation but not AM (NODULATION SIGNALING PATHWAY1; NSP1).

As expected, most (5/8) of the components of the SYM signalling pathway passed the filter applied by Task4 (Additional file 5: Table S1), with the exception of DMI1/POLLUX and the nucleoporins NUP133, and NENA, which were known before to share close homologues with *A. thaliana* [28-30]. NUP85 was lost during the Hieranoid clustering and therefore cannot be used in this context. Furthermore, the LysM-type receptor kinase, as well as RAM1 and RAM2 were retained, whereas the ABC transporter STR was excluded. Genes that are induced during AM, or expressed exclusively in mycorrhizal roots, were also retained through filtering, with the exception of subtilase and PR10, with the latter being represented by a slightly more distant relative (MTR_2g035150 in Additional file 5: Table S1). Importantly, the negative controls (proteins encoded by constitutively expressed genes or by nodulation-specific genes) were eliminated by filtering (Additional file 5: Table S1). These results show that our clustering and filtering procedure has the potential to identify AM-related genes based on conservation of the protein sequence. The fact that STR was removed despite its conservation pattern that would be expected to allow it to pass through our filtering approach [22], can be explained with the fact that it is part of a large gene family (ABC transporters).

### Identifying AM-related proteins by gene expression pattern

AM-related genes can potentially be identified based on their induction during AM development. Genome-wide analysis of AM-related gene expression has been performed in a number of plant species including *M. truncatula*, *L. japonicus*, *S. lycopersicum* (tomato), *Oryza sativa* (rice), and *Petunia hybrida* [19,31-35]. A large set of transcriptomic data is available online for the model legume *M. truncatula* (Medicago GeneAtlas; http://mtgea.noble.org/v3). We used this resource to identify among the protein clusters resulting from Task4 those that are induced at the transcriptional level during AM. We first extracted for all the trees retained by Task4 (2327 clusters) those that had a member from *M. truncatula* (1362 clusters). These clusters represented a total of 2117 genes of *M. truncatula*, of which 1618 genes had at least one Affymetrix probe set in the *M. truncatula* Gene Atlas (MtGEA). After removal of unreliable probesets (see Methods), the expression data for 1526 *M. truncatula* genes representing 1054 protein clusters were used for further analysis.

Expression data are available from various conditions including mycorrhizal roots with *Rhizophagus irregularis* and with *Glomus mosseae*, and laser-microdissected root cortex cells with arbuscules from *R. irregularis*. In addition, expression data of inoculated roots of the *doesn’t make infection3* (*dmi3*) mutant [36,37], roots treated with myc factor (mycLCO) for 6 h and 24 h, and roots treated with low phosphate levels are available. The values of gene expression under these treatments and of the corresponding control treatments, were extracted from the *Medicago* Gene Atlas to calculate induction ratios according to 6 criteria (Table 2). In order to focus on proteins that are induced robustly in mycorrhizal roots, an induction threshold of 3-fold was applied for further filtering of candidate genes. A complete list of genes identified by Task4 with the corresponding expression ratios according to criteria 1-6 is provided in Additional file 7: Table S2.

**Table 2:** **Criteria used for gene expression filtering of** ***M. truncatula*** **genes identified in Task4**

	**Name (Figure** 3 **)**		**Name of treatment in MtGEA**
Criterion 1	LCM	test	Root LCM arbuscular
		cont	Root LCM cortical
Criterion 2	AM	test*	Root (28dpi) Myc (*G. intraradices*) 6wk 20 uM P
			Root (28dpi) Myc (*G. mosseae*) 6wk 20 uM P
		cont	Root non-Myc (control) 6wk 20 uM P
Criterion 3	dmi3	test	Root DMI3 inoculated with *Gigaspora* (early contact)
		cont	Root DMI3 control
Criterion 4	MF_6 h	test	Root WT nsMyc-LCOs 6 h
		cont	Root WT MF control 6 h
Criterion 5	MF_24 h	test	Root WT nsMyc-LCOs 24 h
		cont	Root WT MF control 24 h
Criterion 6	P-repressed	test	Root non-Myc (control) 6wk 20 uM P
		cont	Root non-Myc 6wk 2 mM P

These 6 criteria were applied to the 1526 genes of Task4 (Additional file 7: Table S2) for which a reliabel probe set was available in the *M. truncatula* Gene Atlas (http://mtgea.noble.org/v3). *Expression values were averaged for the two samples inoculated by *G. intraradices* and *G. mosseae*, respectively.

**Figure 3:**
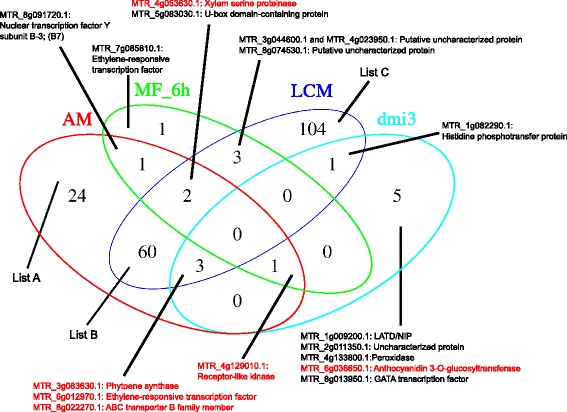
**Venn diagram of** ***M. truncatula*** **genes up-regulated under various AM-related conditions.** Genes listed in Additional file 7: Table S2 (Task4) were subjected to combinatorial analysis according to the criteria listed in Table 2. Red domain, genes induced in mycorrhizal roots; green domain, genes induced by Myc-LCO after 6 h; dark blue domain, genes induced in laser-microdissected cortex cells with arbuscules; light blue domain, genes induced in the *dmi3* mutant (compare with Table 2). Criterion 5 (MF-24 h) did not yield any result. Genes identified according to Criterion 6 are marked in red. List A, B, and C are separately shown in Additional file 8: Table S3.

Combinatorial analysis revealed that 65 genes were commonly induced in mycorrhizal roots and in microdissected arbusculated cells, whereas 26 genes were induced only in mycorrhizal roots, and 173 were induced only in microdissected arbusculated cells, respectively (Figure 3, Additional file 8: Table S3). Only 7 genes were induced by mycLCO after 6 h of treatment, while none of them remained induced after 24 h of treatment (Figure 3). Interestingly, 10 genes were induced in the *dmi3* mutant which is defective for CCaMK, indicating that their expression is regulated independently of the common SYM pathway. Three of these genes were also induced in AM roots, in microdissected arbusculated cells, and in P-starved roots (Table 1, criterion 6) (Figure 3). It will be interesting to explore how these genes are induced in the absence of *DMI3*.

**Figure 4:**
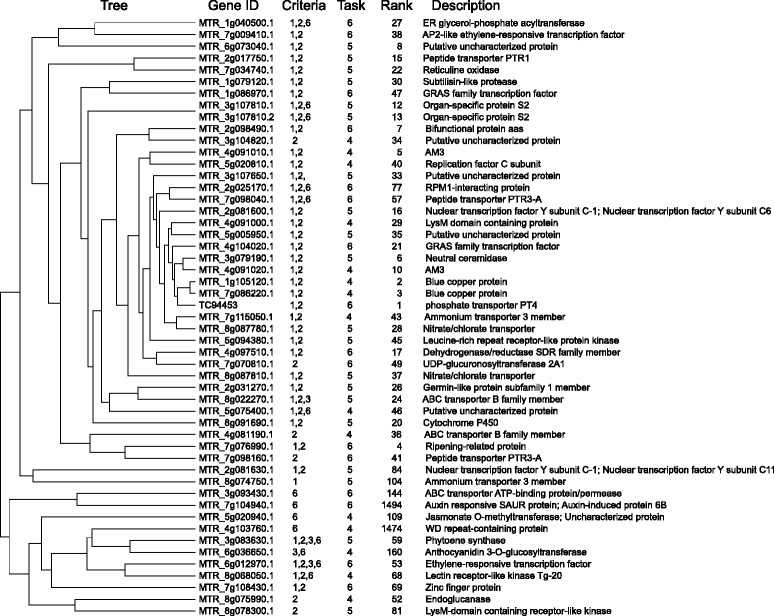
**Details of the AM-related gene cluster shown in Additional file** **9** **: Figure S2.** The hierarchical cluster contains genes induced in mycorrhizal roots. Numbers in the column “criteria” indicate under which conditions the gene was induced at least 3-fold (compare to Table 2). Numbers in the column “Task” indicate in which task the gene was still retained (compare to Table 1). Ranks are assigned according to gene induction in mycorrhizal roots (corresponding to the rank in Additional file 7: Table S2).

**Figure 5:**
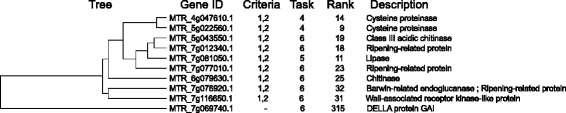
**Cluster of genes induced in both, mycorrhizal roots, and nodule symbiosis.** Details from cluster shown in Additional file 8: Table S3. Numbers in the column “criteria” indicate under which conditions the gene was induced at least 3-fold (compare to Table 2). Numbers in the column “Task” indicate in which task the gene was still retained (compare to Table 1). Ranks are assigned according to gene induction in mycorrhizal roots (corresponding to the rank in Additional file 7: Table S2).

While gene expression patterns can be identified based on defined conditions (e.g. criteria 1-6 in this study), global expression analysis by clustering can identify groups of genes with similar expression patterns over a large set of expression data like the 254 different treatments and conditions covered by the *Medicago* Gene Atlas (http://mtgea.noble.org/v3). This approach can identify groups of genes that are co-regulated and therefore might be functionally related. On the other hand, this approach can lead to the discovery of common regulatory elements in promoters, which are the reason for co-regulation (see below). Hence, we decided to use pairwise average linkage and Pearson correlation in order to identify genes with shared expression pattern (Figure 2, P2; see P2_task4cytoallr.cys in Additional file 3: File S2). All proteins identified by Task4 were correlated based on their standardized gene expression score, i.e. the ratios between the individual expression levels divided by the average of all expression levels as a relative indicator of expression (see Methods). Particular attention was paid to groups of genes that comprised AM-induced genes. One conspicuous cluster of 51 genes with a significantly correlated expression pattern turned out to be highly specific for AM, resulting in apparent vertical red stripes in the visual representation of the cluster (Additional file 9: Figure S2 and Figure 4). A further relevant group comprised genes that are induced commonly in AM and in RNS (Additional file 10: Figure S3 and Additional file 11: Figure S5). These genes may encode proteins that play a general role in symbiotic interactions. Interestingly, this cluster consisted primarily of chitinases, cysteine proteases, a glucanase and several ripening-related proteins (Figure 5).

### Analysis of relative conservation of proteins among angiosperms

Many genes with a role in AM are induced during the interaction. This includes nutrient transporters such as AM-related phosphate transporters [23,38-40] (see also above), and the ammonium transporter AMT2 [41], as well as regulatory components such as the transcription factor RAM1 [21]. In contrast, the genes involved in the early steps of the interaction are constitutively expressed. For example, the expression of the nod factor receptors (NFRs) and of the common SYM genes is not significantly altered during AM development in petunia [19], consistent with their early function in symbiont recognition and signalling.

In order to identify constitutively expressed genes with a potential role in AM development, we decided to compare the relative sequence conservation of proteins in the context of the three AM-relevant plant groups: AM-competent dicots (group A), AM-competent monocots (group B), and non-mycorrhizal dicots (group C) (compare with Figure 1a,b). In order to avoid to miss relevant genes due to proteome incompleteness, we chose the relatively permissive Task3 for this approach (clusters with at least 3 homologues of the 6 AM-competent species but none of the non-mycorrhizal species). Firstly, multiple sequence alignment (MSA) from the sequences represented in the individual clusters retained by Task3 were calculated using MAFFT [42], and secondly, these MSA consensus sequences were used as queries to search by psi-blast the proteomes used for Hieranoid clustering and in addition a number of monocot species, namely *O. sativa* (Osa), *Zea mays* (Zma), *Triticum urartu* (Tur), *Sorghum bicolor* (Sbi), *Hordeum vulgare* (Hvu), *Brachypodium distachyon* (Bdi), and *Aegilops tauschii* (Ata) (group B) (Figure 2, P3; see P3_psiblastProcedure.txt in Additional file 3: File S2).

**Figure 6:**
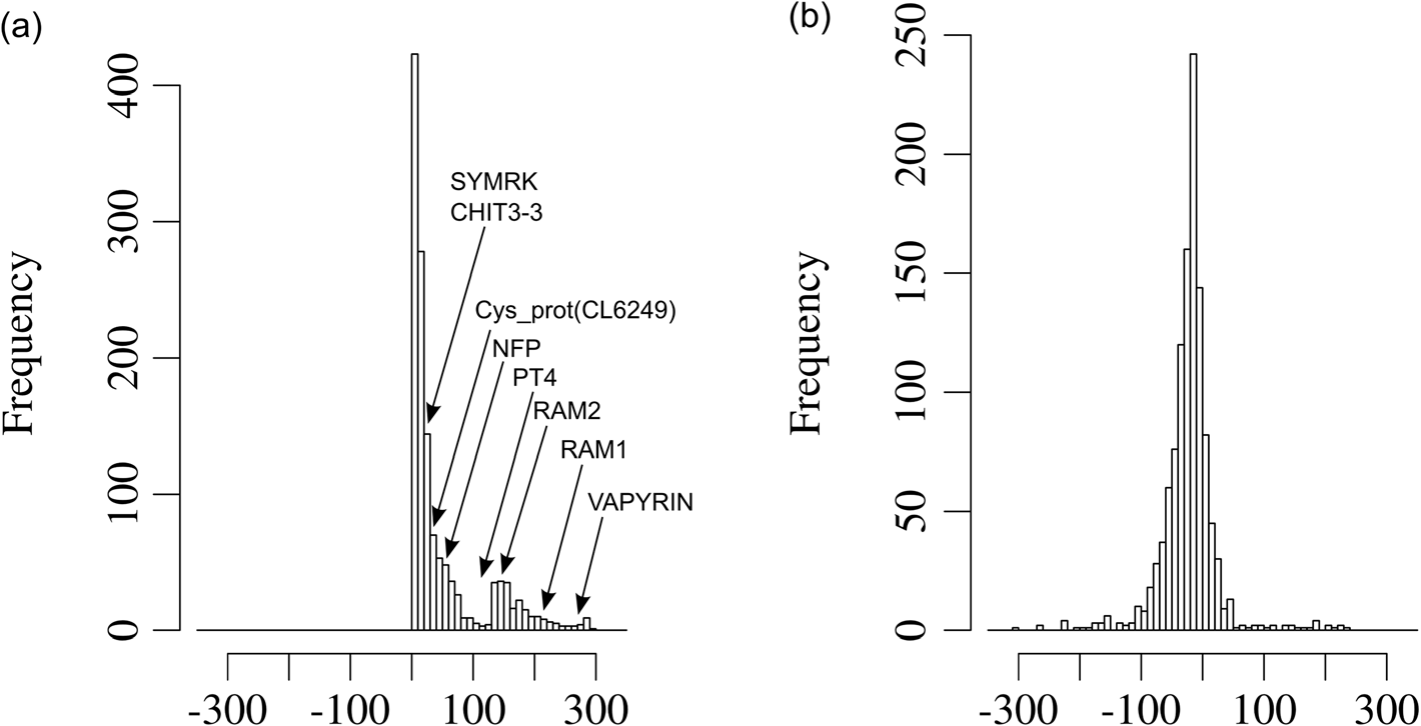
**Conservation ratios of potentially AM-related proteins averaged for relevant plant groups with significant difference between non-AM and AM species.** Histograms represent the frequency distributions of the ratios of log10 of the E-values from psi-blast. The query sequences for psi-blast were generated by calculating MSA consensus sequences based on the results of Task3. These query sequences were blasted against AM-competent dicot species (group A), monocot species (group B), and non-mycorrhizal dicot species (group C) (compare with Additional file 12: Table S4). To derive conservation ratios, the E-values were averaged group-wise for groups A, B, and C, respectively, and the following ratios were generated: C/A and C/B. Conservation ratios were included only if the difference between the groups were significant (p < 0.05 for Wilcoxon test, compare with Additional file 12: Table S4). **(a)** Ratios for log10(group C)/log10(group A). **(b)** Ratios for log10(group C)/log10(group B). Note that 8 control genes from Additional file 5: Table S1 passed the filter in **(a)**, whereas none passed in **(b)**.

The E-values were then compared between the groups by Wilcoxon test (Figure 2, P4; see P4_eval_wilcox.R in Additional file 3: File S2) to identify genes for which the populations of E-values between groups C and A, or between C and B were significantly different (Additional file 12: Table S4). For proteins that exhibited significant differences, the E-values were averaged among the three groups, and the ratios between the log(10) of these values for C/A and C/B were calculated as a relative measure for AM-related sequence conservation (conservation ratio). The higher the conservation ratio, the farther the non-mycorrhizal homologues are from the consensus sequences relative to the homologues from AM-competent species, indicative for AM-related conservation. Establishing the frequency distribution of the conservation ratios revealed that several of our test genes, such as SYMRK, VAPYRIN, RAM1, RAM2, and PT4 passed this filter (Figure 6a), hence their pattern of sequence conservation was significantly related to the competence to engage in AM symbiosis. Surprisingly, none of the test genes passed the comparison between non-mycorrhizal dicots and mycorrhizal monocots (Figure 6b), although at least VAPYRIN, RAM1, and PT4 are more closely related between the AM-competent dicots and the monocots, than between the AM-competent and the non-mycorrhizal dicots [16] (Figure 1a,b).

Since the significance threshold of the Wilcoxon test eliminated many genes, we sought for an alternative way to evaluate the degree of AM-related conservation. Instead of a fixed threshold level, we defined the conservation ratios for a set of household proteins that do not exhibit an AM-related bias in conservation. To this end, we extracted from the Hieranoid clusters those that have representative homologues from all plants, including AM-competent and non-mycorrhizal species (results from Task9; Additional file 13: Table S5). Thus, this set contains conserved house-keeping proteins that can serve as reference for the conservation patterns of proteins identified by Task3. We proceeded in the same way as with the proteins identified by Task3, i.e. an MSA consensus sequence was calculated based on the 6 AM-competent species from each protein cluster, and these MSA sequences were used as queries for psi-blast against all proteomes. As expected, most of the genes selected in this way showed a conservation pattern consistent with the closer phylogenetic relatedness among all the dicots vs. the monocots (Additional file 13: Table S5). This fact is reflected in the phylogenetic trees of the house-keeping genes cyclin D6 and RPL5 (Figure 1a and b), which are also represented in the list resulting from Task9 (Additional file 13: Table S5).

We selected a set of 150 proteins from the results of Task9 with intermediate E-values (excluding E-value = 0) and with genes represented in most monocots (excluding genes marked with NaN in Additional file 13: Table S5). For these 150 genes (marked in yellow in Additional file 14: Table S6), the ratios were calculated as for the genes obtained with Task3 (see above). These values tended to be more to the negative, reflecting the closer position of the *Brassicaceae* homologues from the MSA consensus sequences relative to the proteins obtained with Task4. Hence, the 150 reference genes resulting from Task9 define the range of conservation ratios for housekeeping proteins and therefore allows to define the range that contains proteins with an AM-related bias of conservation (Additional file 14: Table S6).

**Figure 7:**
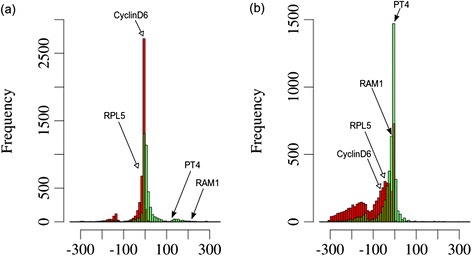
**Conservation ratios of potentially AM-related proteins in comparison with housekeeping genes averaged for relevant groups of plant species.** Conservation ratios were generated as for Figure 6 (see legend of Figure 6). However, no statistics were performed and all ratios are shown. Conservation ratios are compared for potential AM-related proteins extracted by Task3 (green), and for potential house-keeping genes identified by Task9 (red). For comparison, the position of the proteins represented in Figure 1 is indicated (RAM1: AES78316; PT4: AAM76743; cyclin D6: AES67335; RPL5: AES80278). **(a)** Ratios for log10(group C)/log10(group A). **(b)** Ratios for log10(group C)/log10(group B).

Global comparison of the conservation ratios of proteins identified by Task3 and Task9, revealed considerably higher values for the former group (Figure 7), reflecting the different conservation patterns among proteins selected by Task3 and Task9. In the comparison between group A (AM-competent dicots) and group C (non-mycorrhizal dicots), the AM-related genes RAM1 and PT4 were clearly seprated from the housekeeping controls cyclin D6 and RPL5 (Figure 7a), while this distinction was much less clear in the comparison between group B (AM-competent monocots) and group C (Figure 7b). These results show that the conservation ratio can be used as a comparative proxy to evaluate the relative degree of conservation of a given protein among AM-competent species relative to the non-mycorrhizal species.

### Outcome of the *in silico* substraction approach

**Figure 8:**
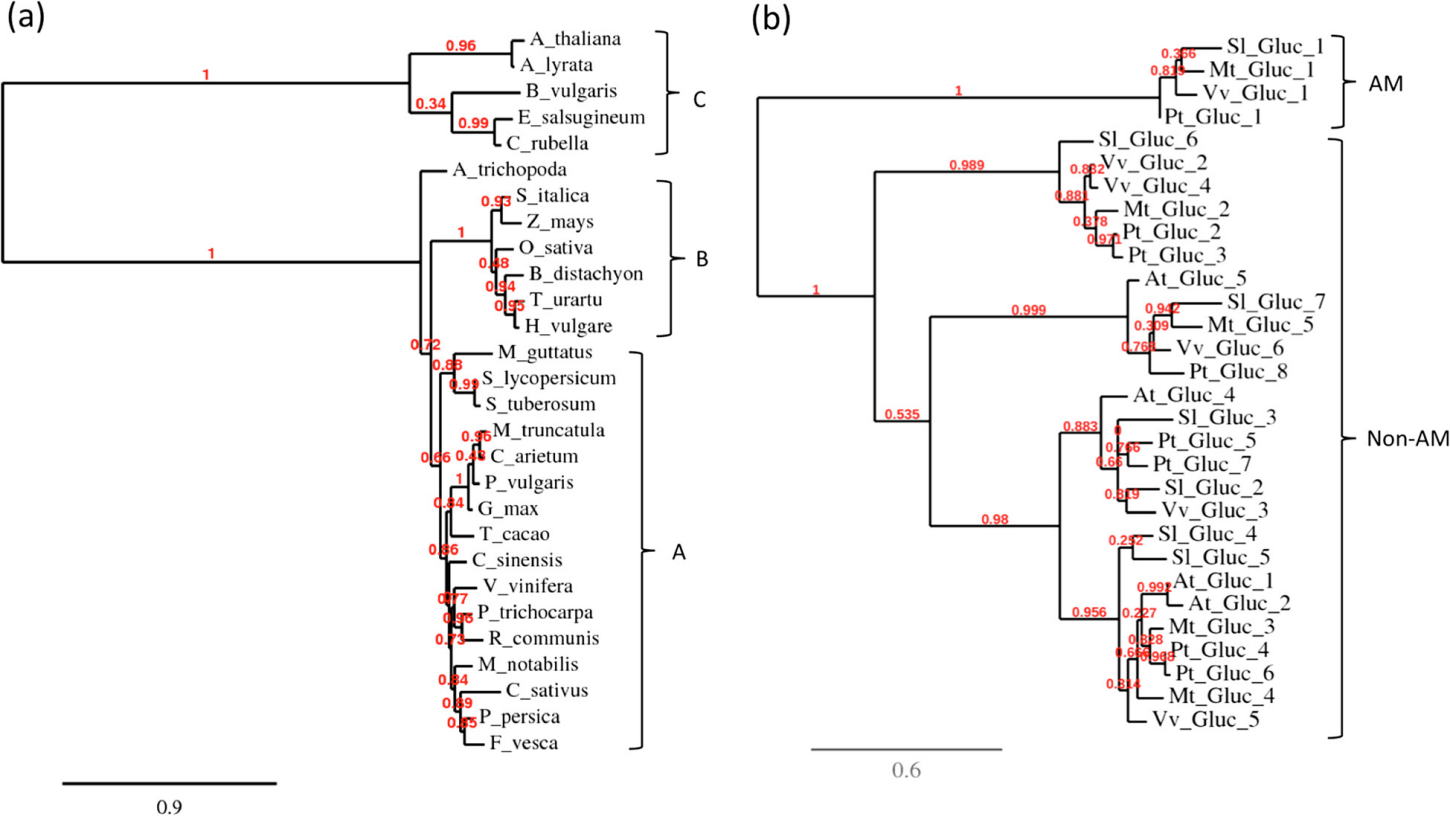
**Phylogenetic analysis of an α-glucosidase with high conservation ratio.** The first hit in the list of predicted AM-related proteins was an α-glucosidase (AES81209, Additional file 12: Table S4). The *S. lycopersicum* sequence was used to retrieve the closest homologues in a wide range of species (Additional file 1: File S1) for phylogenetic analysis. **(a)** Tree as in Figure 1 with the first hit per species identified at NCBI by blastp against non-redundant protein database using the tomato α-glucosidase (Solyc03g094020.2.1) as a query. **(b)** Tree with all homologues of Solyc03g094020.2.1 from tomato (*S. lycopersicum*), grape vine (*V. vinifera*), poplar (*P. trichocarpa*), *M. truncatula*, and *A. thaliana*. Note the AM-related branch (Gluc_1) that has no homologue from *A. thaliana*. The closest *Arabidopsis* homologue (At_Gluc_1) clusters far away.

The goal of this study was to identify AM-related genes based on the conservation pattern of their orthologues between AM-competent and non-mycorrhizal plant species. This approach is particularly targeted to identify AM-related genes that are not induced during symbiosis, hence, we focused on the proteins identified by Task3 that are not induced at the gene level (Additional file 14: Table S6). To evaluate the efficiency of the approach, this list was ordered according to the ratios of E-values between the averaged *Brassicaceae* and AM-competent dicot plants, respectively (Additional file 14: Table S6). The list was sorted in descending order, since the highest values for the conservation ratios indicate the proteins that are conserved to a higher degree among AM-competent species than between AM-competent and non-mycorrhizal species. In this list, the first protein, a predicted α-glucosidase/ xylosidase, was chosen to evaluate its conservation pattern in detail. Indeed, a phylogenetic tree prepared as in Figure 1 shows an extremely skewed pattern of conservation with a clearly resolved common branch of the AM-competent monocots and dicots, including the basal lineage *Amborella trichopoda*, whereas all the non-mycorrhizal species, the *Brassicaceae*, and *B. vulgaris* as a representative of the *Chenopodiaceae*, form an outlier group (Figure 8a).

Hydrolases are often encoded by gene families, and this is also the case for this α-glucosidase/xylosidase. In order to test whether the member with the AM-related conservation pattern forms a dedicated group in AM-competent species, we isolated all the homologues available from the protein database at NCBI for the species *V. vinifera*, *P. trichocarpa*, *S. lycopersicum*, *M. truncatula* and *A. thaliana*. They are numbered in each species based on their similarity to the AM-related homologue in *S. lycopersicum*. A phylogenetic tree with all these sequences revealed that all AM-competent species have a single AM-related homologue, resulting in a clearly separated AM-related branch (Figure 8b). The closest homologue of *A.thaliana* falls into the large containing the remaining sequences. Hence, *A. thaliana* misses only the AM-related form of the α-glucosidase/ xylosidase gene family.

### Search for potential *cis*-regulatory elements in promoters of AM-related genes

Besides the conservation of the ORF, we investigated the non-coding upstream sequences of AM-related genes by searching for potential *cis*-regulatory promoter elements that may control gene activity during symbiosis. We selected the *M. truncatula* proteins identified by Task4 that were at least 3-fold induced in mycorrhizal roots relative to non-mycorrhizal control roots (Figure 3, Additional file 7: Table S2 with criteria LCM or AM >3; 190 genes). Their promoter sequences (2 kb upstream of the start codon), were downloaded from Ensembl Plants BioMart (http://plants.ensembl.org/biomart/martview), and analyzed with the pattern recognition software MEME (http://meme.nbcr.net/meme/doc/cite.html) for overrepresented sequences. A first search revealed a series of conserved predicted promoter elements (Additional file 15: Figure S4a). A highly conserved element comprised the core sequence GGGGTTCGAACCCC (Myc1 element) with the bold letters being almost invariant (Additional file 15: Figure S4, Additional file 16: Table S7). The palindromic nature of this element, and the fact that many *cis*-elements in plant promoters are palindromes [43-45] prompted us to search specifically for palindromic sequences. Interestingly, 7 of the 10 elements predicted by the first search were confirmed by the second search for palindromes (Additional file 15: Figure S4b, compare with Additional file 15: Figure S4a). Besides the Myc1 element, a second element with the well-defined core sequence TGAGCTTAGCTCA (element Myc2) emerged (Additional file 15: Figure S4). An element with completely invariant sequence was the palindrome GCCGGC that tended to be located within the 500 bp immediately upstream of the ATG (Additional file 17: File S5), indicating that it represents a relevant promoter element (Additional file 17: File S5). Besides the GCCGGC element, none of the predicted elements were described previously. An element that could have been expected to result from our MEME search is the AM-related CTTC element (sequence: CTTGTTC), also known as MYCS element [46,47]. This element is present in the promoters of AM-related phosphate transporter genes, and of the AM-related SNARE LjVTI12 [46,47], however, it was not found by MEME in our sample of 190 AM-induced promoter sequences. This surprising result prompted us to search for it in the VAPYRIN promoter, which is induced in mycorrhizal roots of both, *M. truncatula* and *Petunia hybrida* [17,19], indicating that this promoter may contain conserved AM-related promoter elements. Indeed, we found an extended version of the CTTC element (sequence GACTTGTTC) in all promoters of 14 VAPYRIN genes from various monocots and dicots, indicating that it is of a wider significance for AM-related gene regulation (data not shown).

The elements Myc1 and Myc2, the GCCGGC element, and the CTTC element were selected for further statistical analysis in the genome of *M. truncatula*. In order to estimate their relative frequency in the promoters of AM-related genes, we selected three samples: the promoters of the genes identified with Task4 (n = 1547), those of the AM-induced genes whithin Task4 (n = 190), and all promoters of *M. truncatula* (n = 46014). These promoter sequences were searched by blast for the presence of the four elements of interest. For the non-palindromic CTTC element, both orientations were considered separately, and the two respective longer variants (GACTTGTTC and its reverse complement) were included as well (Additional file 16: Table S7). Indeed, element Myc1 and Myc2, as well as the GCCGGC element were significantly overrepresented among the genes identified by Task4 compared to the statistical expectations, and relative to the observed frequency in all promoters (Additional file 16: Table S7). Overrepresentation was even more evident when the AM-induced genes were considered (Additional file 16: Table S7). Significance of these results was confirmed by binomial test. In contrast to the Myc1 and Myc2 elements, and to the GCCGGC element, the CTTC element was not overrepresented in promoters identified by Task4, and only moderately in the AM-induced genes, when its original sequence was considered (CTTGTTC) (Additional file 16: Table S7). However, the longer form (GACTTGTTC) was significantly overrepresented in both orientations in the promoters of the genes identified by Task4 and even more of the AM-induced genes (Additional file 16: Table S7). This indicates that the longer version is the relevant form of the CTTC element.

## Discussion

During the past decade, many screens for AM- or RNS-defective mutants have been carried out and a significant number of genes involved in signalling and symbiotic functioning have been identified [4]. In addition, transcript profiling has identified many AM- and RNS-related transcripts, however, only few of them have been functionally characterized by reverse genetic approaches. A central finding of the majority of these genetic studies was, that the genes involved in symbiosis are well conserved among AM-competent species, whereas they are conserved to a lesser extent, or entirely missing, in non-mycorrhizal species such as *A. thaliana*. Based on this finding, we designed a strategy for a third avenue to identify yet unexplored AM-related genes using the conservation of their coding region as the main criterion. We compared the proteomes of AM-competent and non-mycorrhizal plant species, thereby isolating the proteins that are conserved only, or to a higher degree, in AM-competent species. Essentially, this approach represents an *in silico* substraction procedure, where the proteomes of the non-mycorrhizal species are substracted from the proteomes of the AM-competent species to yield a fraction of the proteome that is expected to be enriched in AM-related proteins.

Since function is selected for at the level of the protein, and since sequence conservation at the DNA level is affected by the degeneracy of the genetic code, we compared the species at the proteome level. Ideally, an *in silico* substractive approach would involve an equal number of proteomes of the AM-competent and the non-mycorrhizal group. However, due to the wide success of AM in nature, non-mycorrhizal species are the exception (estimated 10-20% of the angiosperm species), and only few of them have been sequenced to a degree that they could be used for such an approach. As non-mycorrhizal species, only *A. thaliana*, *A. lyrata*, and *B. rapa* were available as proteomes in the ENSEMBL database. In contrast, a wide range of AM-competent species is available, among them various dicots (legumes, Solanaceae, etc.) and cereals. Hence, in a first approach, we selected two AM-competent monocots (*O. sativa*, *S. bicolor*), four AM-competent dicots (*S. lycopersicum*, *V. vinifera*, *M. truncatula*, and *G. max*), and the two non-mycorrhizal species *A. thaliana* and *B. rapa*.

The first step in this approach was to establish the orthologous relationships between the proteomes of the eight selected species. This task was performed with Hieranoid, which establishes clusters of orthologous/paralogous proteins in a hierarchical step-wise procedure that proceeds according to a previously defined phylogenetic tree of the involved species. The subsequent step is to identify protein clusters that have members in all or most of the AM-competent species, but none in the non-mycorrhizal species. However, after a first run with Hieranoid, we noticed that in many cases, the orthologous trees of known single proteins fell into a monocot and a dicot tree, thus preventing our strategy. Therefore, we decided to perform a clustering just using dicot species, namely *G. max*, *M. truncatula*, *P. trichocarpa*, *V. vinifera*, *S. lycopersicum*, and *S. tuberosum*, together with the three non-mycorrhizal species *A. thaliana*, *A. lyrata*, and *B. rapa* (see Additional file 2: Figure S1). In order to assess the efficiency of the procedure, we used a number of known AM-related genes and a few housekeeping genes for reference (Additional file 5: Table S1).

Clustering of all nine proteomes resulted in a total of 28’528 protein clusters. Subsequent selection of clusters that contain orthologues only in AM-competent species established a first set of potential AM-related proteins (Figure 2, Task6). In order to account for cases were individual orthologues may be missing from one or two proteomes, we relaxed the stringency of the filtering by allowing also for clusters were 1 or 2 members of AM-competent species were missing (Task5 and Task4, respectively) (Table 1, Figure 2). An even more permissive filter (Task3) was applied to obtain a large population of proteins that were subsequently subjected to quantitative assessment of amino acid sequence conservation based on the comparison of E-values (Figure 2, Additional file 12: Table S4, Additional file 13: S5 and Additional file 14: S6). The fact that Task3 and Task4 yielded rather long lists of candidate proteins (>1000 clusters) was not considered a problem, since subsequent filtering and sorting narrowed the list further down (see below).

A next step was to use gene expression as a criterion for selection, hence, we defined expression ratios relevant for AM (Table 2). Based on these criteria we sorted the list of proteins identified by Task4. By applying a stringent threshold induction ratio of 3-fold, we restricted the analysis to genes with a robust transcriptional induction in mycorrhizal roots (Figure 3, Additional file 7: Table S2). Besides known AM-related proteins such as PT4, AM3, VAPYRIN, RAM2, and IPD3, this list included several interesting proteins like the LysM receptor kinase LYR1, a close homologue of the *Lotus japonicus* nod factor receptor NFR5 [48], and several ABC transporters and peptide transporters, that may play a role in AM development. Highly induced candidates with yet unknown functions in AM include a triacylglycerol lipase (MTR_7g081050.1), neutral ceramidase (MTR_3g079190.1), an enzyme with a role in sphingolipid metabolism [49], and reticuline oxidase (MTR_7g034740.1) an enzyme involved in the biosynthesis of the alkaloid intermediate reticuline [50]. The fact that many known AM-induced genes were recovered by our selection procedure (Additional file 7: Table S2 and S3) documents the potential of gene discovery by comparative phylogenetics.

The main goal of this study was to identify new AM-related proteins based on conservation of the coding sequence, i.e. independently of AM-related gene induction. In order to obtain a quantitative measure for conservation of the open reading frame (ORF) in AM-competent species, we performed a secondary filtering step by blasting the conserved consensus sequences derived from the 6 AM-competent species (MSA) against a wider panel of plant proteomes including dicots, monocots, the liverwort *Selaginella moellendorfii*, the moss *Physcomitrella patens*, and the unicellular alga *Chlamydomonas reinhardtii*. The interest to include these three lower plants is based on the finding that several of the AM-related genes were found in liverworths and/or mosses [5,6], potentially revealing the evolutionary origin of the common SYM pathway in lower land plants [8]. The psiblast was performed with the results of the relatively permissive Task3 (protein clusters containing at least 3 of the 6 AM-competent species). With this approach 1329 proteins were identified for which the E-values between the three relevant groups of plant species were significantly different (Wilcoxon test, p < 0.05) between the non-mycorrhizal species, and the AM-competent dicots and monocots, respectively (Additional file 12: Table S4, Figure 6), indicating that they are conserved particularly well in AM-competent species. Interestingly, the 50 proteins with the lowest p-values (i.e. highest AM-related conservation) included 13 proteins with annotations as receptors, protein kinases, phosphatases, disease resistant proteins, or G-protein regulating protein, implying roles in signalling.

In view of the essential role of AM in plant nutrition, a highly conserved ammonium transporter (Additional file 12: Table S4, rank 572), and the oligomineral nutrient transporter Nramp5 (rank 495) are of particular interest. Ammonium is the form in which nitrogen is thought to be transferred from the AM fungus to the plant [51,52], and therefore, ammonium transporters are of particular importance in AM [41]. Transporters for other mineral nutrients such as zinc and copper remain to be identified. Many of the conserved proteins were also found to be well conserved in the liverwort *S. moelledorfii*, and some of them even in the moss *P. patens*, whereas *C. reinhardtii* proved to be phylogenetically much more distant (Additional file 12: Table S4).

In order to obtain a direct quantitative measure for the degree of AM-related conservation, we defined the conservation ratio, which represents the ratio of the averaged E-values from psiblast (as log10) between the non-mycorrhizal species, and the AM-competent species (dicots or monocots, respectively) (Additional file 14: Table S6a). For comparison, the results from Task9 (proteins found in all species) were included as representatives of generally well conserved housekeeping proteins. The larger the conservation ratio, the more a protein sequence is divergent between the non-AM plants and the AM-competent species. Therefore, the proteins identified by Task3 have mostly positive values, whereas the housekeeping proteins (Task9) have neutral or negative values (Figures 6 and 7). Of particular interest were proteins with a conservation ratio >100, which comprised for example PT4 and RAM1 (Figure 7a), which are known to show a highly AM-related conservation pattern (Figure 1). A general overview over the conservation ratios (Additional file 14: Table S6a) revealed that for 220 proteins, the conservation ratio could not be calculated because they were entirely missing from the psiblast results in the non-mycorrhizal species (NaN in Additional file 14: Table S6a). In addition, a further 272 proteins exhibited conservation ratios of >100, indicative of a high degree of AM-related conservation. In order to define a range above which the conservation ratios can be considered as being skewed towards the AM-competent species, 150 proteins identified by Task9 (houskeeping genes) were included for reference in the results from Task3 (see Additional file 14: Table S6a; proteins marked in yellow).

Among the 220 proteins that were missing from the non-mycorrhizal species (Additional file 14: Table S6a), 123 have unknown functions. Interestingly, three proteins each are predicted chitinases (TASK3_1488, TASK3_1548, TASK3_1649), and glucanases (TASK3_566, TASK3_581, TASK3_4113), indicating potential roles in cell wall modification or signaling. Three predicted CLE peptides (TASK3_3616, TASK3_3634, TASK3_4020) might be involved in AM-related signaling as it was shown for RNS [53]. A known component in symbiotic signaling comprised INTERACTING PROTEIN of DMI3 (IPD3)/ CYCLOPS (TASK3_1003), an interactor of the central symbiosis regulator CCaMK encoded by *DMI3* [54-56]. High conservation ratios were also found for VAPYRIN (TASK3_795), which is required both for AM and RNS [16,17,26] and VAPYRIN-like (TASK3_1469).

16 proteins exhibited a value >100 for both conservation ratios (Additional file 14: Table S6b), i.e. they are highly conserved among both, AM-competent dicots and monocots, relative to the non-mycorrhizal species. Of particular interest is TASK3_196 which encodes a predicted α-glucosidase (Additional file 12: Table S4 and Additional file 14: S6b) which is in some species classified as α-xylosidase (e.g. XP_006358190.1 in *S. tuberosum*). While the conserved protein is missing from *A. thaliana* (Figure 8a), there are related glycosidases that occur in all examined plant species including *A. thaliana*, indicating that the non-mycorrhizal model species misses only the AM-related isoform (Figure 8b). The potential role for α-glucosidase or α-xylosidase in AM is unknown, but given the important contribution of xylose in the hemicelluloses of the cell wall [57], it may be involved in the modifications of cell walls during AM infection and functioning [20]. Interestingly, a predicted α-xylosidase of petunia is among the most strongly repressed genes under conditions of high phosphate, which inhibits AM [19].

Five of the 16 proteins with high conservation ratios (>100) represent receptors and protein kinases, of which three (Task3_354, Task3_238, and Task3_4055) are closely related receptor-like protein kinases (RLKs) (Additional file 14: Table S6b). They are similar to S-locus receptor kinase (SRK) [58], which mediates sporophytic self-incompatibility in the *Brassicaceae* [59], and to the maize receptor kinase ZmPK1 [60]. Phylogenetic analysis showed that they indeed fall into a large clade of proteins found in all AM-competent species, but with considerably lower conservation in the non-mycorrhizal species (Additional file 11: Figure S5). A conserved C-rich stretch in the amino acid sequence is shared among the three AM-related RLKs, as well as with SRK, and ZmPK1 (Additional file 18: Figure S6), indicating that they may have similar tertiary structures mediated by cysteine bridges. Interestingly, *ZmPK1* is expressed in roots and young seedlings, rather then floral tissues [60] suggesting that this family of receptor kinases can have other functions than self-incompatibility. Taken together, the examples of α-glucosidae/xylosidase and the RLKs show that our approach can identify proteins with AM-related conservation patterns even if they are members of large families. Furthermore, the case of the three related RLKs highlights the potential of our approach to identify proteins that could potentially escape genetic mutant screens because of functional redundancy.

Transcription is controlled primarily by *cis*-acting regulatory sequences in the promoters of the genes. Hence, co-regulated genes can be expected to share common regulatory elements in their promoters. Based on this logic, we screened the promoters of 190 AM-induced genes for overrepresented sequences that may represent *cis*-acting regulatory promoter elements. The prediction program MEME identified two new palindromic elements (elements Myc1 and Myc2) and found the GCCGGC element to be overrepresented in AM-induced promoters as well as in the promoters of genes identified by Task4. Statistical analysis confirmed the overrepresentation of these elements in AM-related promoters (Additional file 16: Table S7). The statistical significance of element Myc1 and Myc2 is emphasized by their rather unusual length (14 nt and 13 nt, respectively) compared to other *cis*-acting elements that are typically less then 10 nt in length [61]. In addition, element Myc1 and Myc2 are often coupled (see Additional file 17: File S5), underscoring their relevance for AM-related gene induction, and suggesting that they may function in conjunction.

The predominant position of the GCCGGC element in a range of 500 bp upstream of the ATG start codon is indicative of functional relevance as well. The GCCGGC element can be bound by the transcription factor RAP.2.11 in ethylene-responsive promoters involved in the potassium starvation response of *A.thaliana* [62], and it was classified as wound- and pathogen-inducible [63]. GCCGGC represents a palindromic variant of the common GCC element (sequence GCCGCC) that is widely found in the promoters of pathogenesis-related genes and in ethylene-responsive promoters of various plants [64-70]. In fact, GCC elements occur in different variants, which are recognized by specific members of the ETHYLENE RESPONSE FACTOR family of transcription factors [69]. The overrepresentation of the GCCGGC element in AM-related promoters may explain the frequency of predicted stress- and pathogen-related transcripts in the transcriptome of mycorrhizal roots in plant species such as rice, potato, petunia, parsley, *M. truncatula*, and *L. japonicus* [19,32-35,71-73].

Surprisingly, the AM-related CTTC/MYCS element (sequence: CTTGTTC) [46,74] was not found by MEME in the 190 AM-induced promoters. Furthermore, it was not overrepresented in the genes resulting from Task4, and only moderately in the AM-induced genes (Additional file 16: Table S7). However, an extended form (GACTTGTTC), was found to be significantly overrepresented, indicating that this may represent the complete AM-inducible element in *M. truncatula*. An overview over the occurrence of predicted promoter elements in the analyzed promoters is provided in Additional file 19: Table S8.

Taken together, we have identified two new potentially AM-associated *cis*-regulatory promoter elements. In addition, we identified the GCCGGC element as a potential AM-related element that may indicate an involvement of ethylene in AM development, and we present a modified (elongated) form of the CTTC element. Future work will address the functional relevance of these elements for AM development and functioning,

## Conclusions

We describe a non-targeted approach to identify new AM-related genes by genome-wide comparative sequence analysis of AM-competent and non-mycorrhizal plant species. The validity of this approach has been confirmed by the finding that it identified many known AM-related genes from the genomes of AM-competent plant species. Following this strategy, we identified many new potentially AM-related genes based on the conservation pattern of the coding sequence, on gene expression pattern, and on predicted regulatory promoter elements. These genes will be further evaluated for their predicted function (pathway), the degree of their conservation, and the copy number, or the number of similar genes in the case of gene families. This process will result in a short list of genes that will be further functionally analyzed by insertional mutagenesis in *P. hybrida*, *M. truncatula* or *L. japonicus* using established transposon-related protocols [74-76].

## Methods

### Phylogenetic sequence analysis

**Figure 9:**
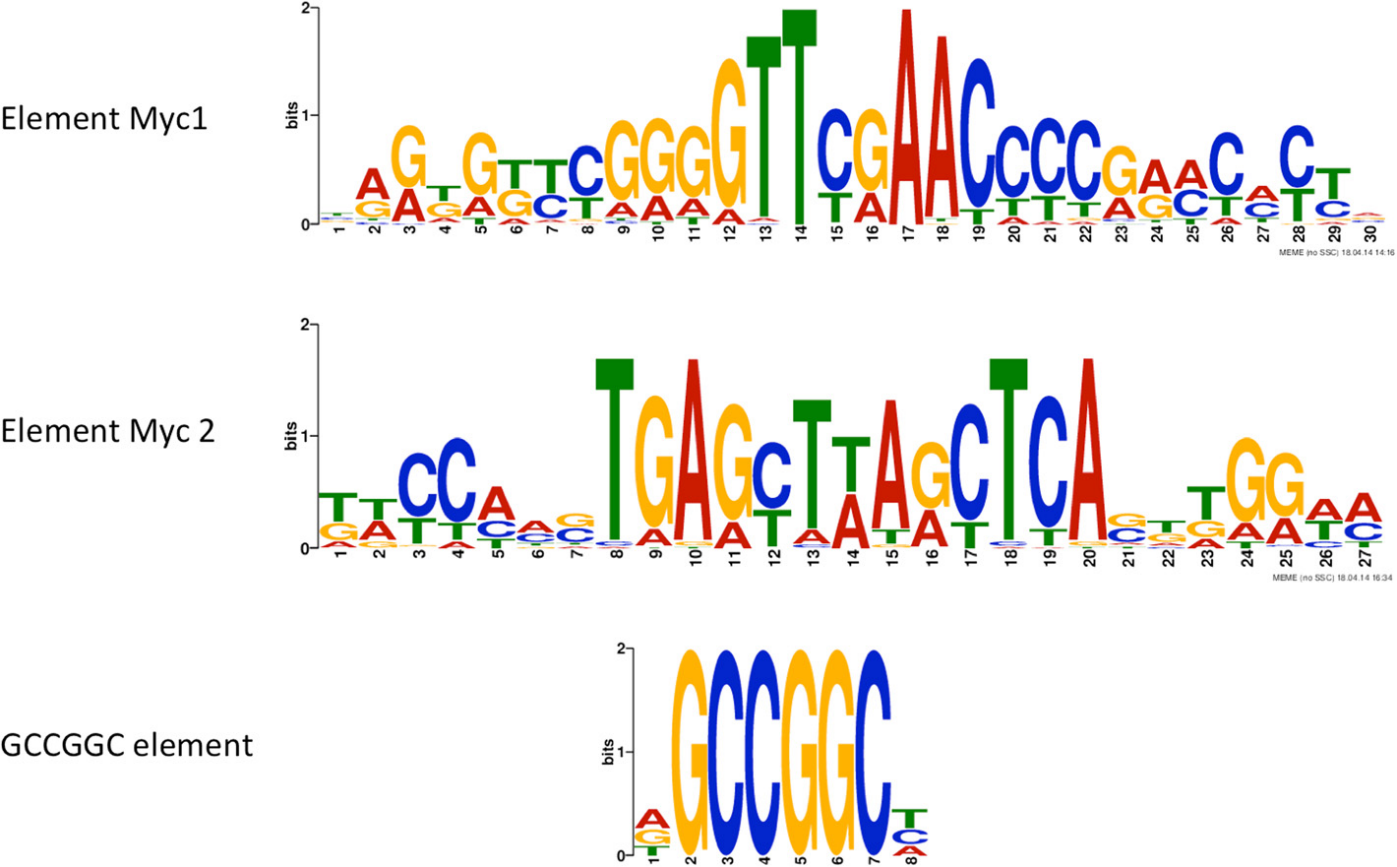
**Predicted regulatory elements in the promoters of AM-responsive genes.** Promoter elements were identified by MEME from 190 promoters of genes represented in Task4 that are significantly upregulated (>3x) in mycorrhizal roots or in microdissected cells with arbuscules (red and blue domains in Figure 3, Additional file 8: Table S3). The search was set to palindromic sequences of 6-30 nucleotides.

Phylogenetic sequence analysis for Figures 1 and 9 was performed using the protein sequences provided in Additional file 1: File S1. To obtain these sequences, the first sequence for each group (marked in bold in Additional file 1: File S1; e.g. M. truncatula RAM1 for the RAM1 group) was used as a query for a protein blast at NCBI against the non-redundant protein database. From the results of this blast, the first hit for each plant species was retrieved. Phylogenetic analysis was performed as described [77] with the basic function (“One-click”). Red numbers on the branches of phylogenetic trees represent indices from an approximate Likelyhood Ratio Test (aLRT) [78]. aLRT vales are equivalent to bootstrap values and indicate well supported branches (close to 1) or weakly supported branches (close to 0).

### Databases

For clustering with Hieranoid, the proteomes of the following species were downloaded from the Ensembl Plants website (www.ensemblgenomes.org; database release 20) [79]: *Arabidopsis thaliana* (Ath), *Arabidopsis lyrata* (Aly), *Brassica rapa* (Bra), *Medicago truncatula* (Mtr), *Glycine max* (Gma), *Vitis vinifera* (Vvi), *Solanum lycopersicum* (Sly), *Solanum tuberosum* (Stu) and *Populus trichocarpa* (Ptr). The proteome database contains 35387 proteins for Ath, 32668 for Aly, 41026 for Bra, 46020 for Mtr, 73320 for Gma, 29928 for Vvi, 34676 for Sly, 56211 for Stu, and 45779 for Ptr. Three protein sequences (PT4, CASTOR and VAPYRIN), that are involved in mycorrhizal development and functioning, were missing from the proteome of *M. truncatula*, and were therefore added to the fasta Mtr file before clustering and further analysis with MtGEA. For calculation of conservation ratios (see below), the proteomes of the following additional species were downloaded: *Oryza sativa* (Osa), *Zea mays* (Zma), *Triticum urartu* (Tur), *Sorghum bicolor* (Sbi), *Hordeum vulgare* (Hvu), *Brachypodium distachyon* (Bdi), and *Aegilops tauschii* (Ata).

### Orthology/homology inference with Hieranoid

Clustering of the proteomes was performed with the Hieranoid software [25] that is based on a pairwise hierarchical method guided by a phylogenetic tree (Additional file 2: Figure S1). The relative distances between the branches of this tree are irrelevant for Hieranoid, hence, they were arbitrarily set to 4. Only the topology of the tree is considered in a heuristic process referred to as hierarchical orthology inference. The resulting clusters of homologues protein sequences were saved in a file called eudicotyledons.OGTree.txt (Additional file 4: File S3, script P0_HieraProcedure). Additional filtering steps were applied to this file using the Newick utilities [80] keeping only clusters that contained sequences from at least X among the six non-Brassicaceae species, but lacked orthologues of all three Brassicaceae proteomes (Additional file 4: File S3, P1_hieranoid_output_treatment.sh). The respective results are named TaskX.

### Gene expression data

All expression data were downloaded from the *Medicago truncatula* Gene Expression Atlas (MtGEA version 3; http://mtgea.noble.org/v3) [81,82]. Expression values from the treatments listed in Table 2 were downloaded to calculate expression ratios according to criteria 1-6 (Table 2). These probesets used for this approach covered 1110 protein clusters, i.e. approximately 48% of all clusters resulting from Task4 (see Table 1). In order to remove unreliable probesets, those with the extension “_x_at” and “_s_at”, which signifies probes that are not unique in the genome, or that recognize multiple similar genes, respectively, were eliminated. In addition, probesets that showed incomplete identity with the target sequence were removed if they had a “Genome Identity Factoring All Probes in the Same Probeset” inferior to 70% (MtGEA, MGAG Gene to Affymetrix GeneChip Mapping). This curation resulted in a set of a total of 1526 Mtr genes that represent 1054 protein clusters (45% of all clusters identified by Task4).

### ClusterMaker

In order to cluster the genes according to shared expression patterns, the open-source software Cytoscape v. 2.8.3 [83] was used with the ClusterMaker plugin [84] to process the microarray data from MtGEA. The affymetrix values (x) were standardised with the scale-score method as follows (eq. 1).

1 $$ Y=\frac{\left(X-\mu \right)}{\rho } $$

with X = ln(x), μ: average of all X values (from all probesets) per treatment, ρ: standard deviation of all X values (from all probesets) per treatment. ClusterMaker v.1.11 was run with the following options: “pairwise average-linkage” for Linkage, and “Pearson correlation” for Distance Metric. The option “all the array sources” (Microarray data in log scale) with the choices “Cluster attributes as well as nodes” and “Ignore nodes/edges with no data”.

### Comparison of conservation level between different plant groups

First, a consensus sequence was built for each tree in Task3 by multiple sequence alignment (MSA) using MAFFT [42]. These consensus sequences were used as queries to psi-blast the proteomes used for Hieranoid clustering (see above) and an additional set of AM-competent monocots. Significant differences in the level of amino acid sequence conservation between groups A (AM-competent dicots), B (AM-competent monocots), and C (non-mycorrhizal dicots) was determined by Wilcoxon test on the E-values (resulting in the p-values in Additional file 12: Table S4 and Additional file 13: Table S5).

For the calculation of conservation ratios, the E-values were averaged among group A (AM-competent dicots: Mtr, Gma, Sly, Stu, Vvi, Ptr); group B (AM-competent monocots: Osa, Zma, Tur, Sbi, Hvu, Bdi, and Ata); and group C (non-mycorrhizal dicots: Ath, Aly, Bra). Subsequently, the ratios of averaged E-values were calculated to determine the relative relatedness of proteins identified by Task3 (AM-related genes) and Task9 (houskeeping genes) between group A, B, and C (Additional file 14: Table S6, Figures 6 and 7).

### Analysis of promoter elements

For the search of potential regulatory elements in promoters, the 2 kb upstream sequences of all predicted promoters of *M. truncatula* were downloaded from Ensembl Plants BioMart (http://plants.ensembl.org/biomart/martview). To predict potential regulatory *cis*-acting elements related to AM, the promoters of the genes identified by Task4 and induced at least 3-fold in AM and/or in cortical cells with arbuscules (n = 190) were subjected to analysis by the pattern recognition software MEME (http://meme.nbcr.net/meme/doc/cite.html) [85]. In a first approach, the search was set to sequences of a length of 6-30 nt, a second search was performed to identify preferentially palindromic sequences.

To evaluate the frequency of predicted *cis*-acting elements in the promoters of AM-related genes, the Myc1 and Myc2 element (Figure 9), as well as the GCCGGC element and the CTTC/MYCS element [46,47] were searched for in the promoters of the *M. truncatula* genes identified by Task4 (n = 1547), in the promoters from Task4 that were induced >3-fold in mycorrhizal roots and/or in laser-microdissected cells with arbuscules (n = 190), and in all promoters of *M. truncatula* (46014) by using Jemboss “Nucleic-Motif-Fuzznuc” [86]. Overrepresentation of the predicted elements in the respective samples (relative to the expected frequency based on random sequences) was tested for by binomial test with a coefficient level of 95% compared to a Fisher value of a = 0.05 (Additional file 16: Table S7). Fold overrepresentation in promoters from genes identified by Task4 (Task4/all), and in AM-induced promoters (AMup/all) relative to all promoters was calculated as the ratios of the relative frequencies (% rel. freq.).

## Availability of supporting data

Microarray data are available in the *Medicago truncatula* Gene Expression Atlas (MtGEA version 3; http://mtgea.noble.org/v3). Plant proteomes were downloaded from the Ensembl Plants website (www.ensemblgenomes.org; database release 20). Promoter sequences of *M. truncatula* were downloaded from Ensembl Plants BioMart (http://plants.ensembl.org/biomart/martview). Phylogenetic trees were prepared with the online tool www.phylogeny.fr using the standard mode (“One-Click”) with the sequences provided in Additional file 1: File S1. All Supporting Information has been deposited on LabArchives (http://www.labarchives.com).

### Note added in proof

Since the submission of this manuscript a comparable gene discovery workflow has been published [87]. This paper addresses the evolution of symbiosis-related genes in plants. We provide a comparative list (Additional file 20: Table S9) that documents the overlap of predicted symbiosis-related core genes from Delaux et al. and from our study.

## Additional files

Additional file 1: File S1. Protein sequences used for phylogenetic analysis of RAM1, PT4, RPL5, cyclinD6 (Figure 1), α-glucosidase/xylosidase (Figure 8), and SRK homologues (Additional file 11: Figure S5). Additional file 2: Figure S1. Conceptual phylogenetic tree of plant species for clustering with Hieranoid. This qualitative phylogenetic tree was used for pair-wise comparison of protein sequences in a step-wise scheme for the construction of protein clusters by Hieranoid. Distances between nodes are irrelevant for this procedure and were set randomly at 4. Additional file 3: File S2. Collection of procedures and scripts shown in Figure 2. Zip file of procedures (P0 and P3), scripts (P1 and P4) and Cytoscape file (P2) described in Figure 2 and in the Methods. The following scripts (blastcheck.sh, compilMSA.sh, compilMSAinfo.sh, OGT2MSA.pl and task.lua) and data (all9.tre and MappingsMtrAffy.csv) were necessary to properly execute the scripts. Additional file 4: File S3. Source file from Hieranoid clustering. Text file containing the information for all 28’528 trees of orthologous/paralogous protein trees used for filtering with Task3 to Task9. Additional file 5: Table S1. Control proteins as reference for the filtering strategy. Proteins with known function were subjected to filtering by Task4, Task5, and Task6 to test whether the filtering procedure can distinguish AM-related proteins form housekeeping proteins. AM-related proteins were retained either with all three filters (≤6), with Task4 and Task5 (≤5), or only through Task 4 (≤4). Proteins were exluded either because they had homologues in non-mycorrhizal species such as *A. thaliana* (classified as “No”), or because homologues were found in less than 4 AM-competent species (classified as “No†”). See Additional file 6: File S4 for cited references. Additional file 6: File S4. References cited in Additional file 5: Table S1. Additional file 7: Table S2. Expression analysis for genes identified by AM-related conservation of their coding sequence The gene list of Task4 was tested for their expression according to criteria 1-6 (see Table 2). For AM-induced genes (criterion 2), the induction ratios were averaged between the expression results with *R. irregularis* (referred to as *G. intraradices* on the Affymetrix array), and *G. mosseae*. The list is ordered according to decreasing induction ratios of criterion 2. Additional file 8: Table S3. Gene lists resulting from combinatorial analysis of gene expression according to criteria 1-6 (compare with Additional file 8: Table S3 and Figure 3). List a: Genes induced only in mycorrhizal roots. List b: Genes induced in mycorrhizal roots and in microdissected roots with arbuscules. List c: Genes induced exclusively in microdissected roots with arbuscules. Additional file 9: Figure S2. AM-inducible genes identified by clustering based on global gene expression patterns. Hierarchical gene clustering according to gene expression patterns was performed using the entire dataset in MtGEA including all 254 treatments for all probe sets. The cluster was selected based on its high induction of gene expression in mycorrhizal roots (criterion 2). Values corresponding to the different treatments (x-axis) for each gene (y-axis) are displayed in red if the value is above the average scale-score and green if it is lower. Additional file 10: Figure S3. AM- and nodulation-inducible genes identified by clustering based on global gene expression patterns. Hierarchical gene clustering according to gene expression patterns was performed using the entire dataset in MtGEA including all 254 treatments for all probe sets. The cluster was selected based on its high induction of gene expression in both, mycorrhizal roots (criterion 2) and root nodules. Values corresponding to the different treatments (x-axis) for each gene (y-axis) are displayed in red if the value is above the average scale-score and green if it is lower. Additional file 11: Figure S5. Phylogenetic tree of receptor-like protein kinases of diverse plant species. An unrooted phylogenetic tree was generated with the three *M. truncatula* sequences identified in Additional file 14: Table S6b (Task3_238, Task3_354, and Task3_4055; see arrows), together with the closest homologues from diverse plant species as indicated. Maize lectine-like protein kinase (ZmPK1; asterisk) is included for reference. Additional file 12: Table S4. List of AM-related genes resulting from Task3. MSA consensus sequences were computed from Task3 protein clusters and used as queries for psiblast against the proteomes of the indicated species. P-values of <0.05 (Wilcoxon-test) indicate significantly different E-values between the groups as indicated. Additional file 13: Table S5. List of housekeeping genes resulting from Task9. Gene list as in Additional file 12: Table S4, but for proteins identified by Task9 (housekeeping genes). Additional file 14: Table S6. Proteins with AM-related pattern of sequence conservation. (a) Gene list combined from Additional file 12: Table S4 (AM-related genes) and 150 genes from Additional file 13: Table S5 (houskeeping genes). Conservation ratios were calculated by averaging the log(10) of the E-values per group of plant species (A, B, C), and by dividing C/A and C/B. In order to calculate reliable conservation ratios for the housekeeping genes, only the proteins were selected from Additional file 13: Table S5 that had E-values greater than zero, and not more than one missing member in the different plant species. Housekeeping genes are highlighted in yellow to reveal the range of conservation, above which conservation is biased in AM-competent species. The indices TASK3_xxx and TASK9_xxx corresponds to the indices in Additional file 12: Table S4 and Additional file 13: S5, respectively. Protein annotation from different species was obtained by using the first possible hit in the proteomes with the following series of priority: *M. truncatula, S. lycopersicum, S. tuberosum, O. sativa, A. lyrata, A. thaliana, A. tauschii, B. distachyon, B. rapa, C. reinhardtii, G. max, H. vulgare, P. patens, P. trichocarpa, S. bicolor, S. moellendorfii, T. urartu, Z. mays*, and *V. vinifera*. Proteins marked in green represent members with a highly AM-related conservation pattern as shown in Additional file 14: Table S6b. (b) Extract from Additional file 14: Table S6a with proteins with highest conservation ratio (>100) in both, AM-competent dicots and monocots, relative to non-mycorrhizal species. Additional file 15: Figure S4. Potential *cis*-regulatory elements predicted from 190 AM-induced promoters. Potential regulatory elements were predicted by MEME for any elements between 6-30 nt (a), and for palindromic elements between 6-30 nt (b). Numbers behind the sequences represent the number of occurrences among the 190 promoters analyzed, and an E-value provided by MEME. Blue lines link related sequences that came up with both searches; black lines underline common sequence elements between related predictions. Additional file 16: Table S7. Statistical analysis of the frequency of predicted *cis*-acting elements in the promoters of different groups of genes. The elements predicted by MEME as conserved regulatory *cis*-elements (Myc1 and Myc2 element, and GCCGGC), and the CTTC element were blasted against the promoters of genes identified by Task4, and of AM-induced genes (AM up). For reference, the predicted promoter elements were blasted against the promoter sequences of all *M. truncatula* genes. Total: number of promoters (n); Hits: number of promoters with element; p-value: Result from binomial test; adjusted p-value: p-value*n; Rel. freq.: Hits/Total. Additional file 17: File S5. Results from MEME analysis for palindromic regulatory elements in AM-induced promoters. (compare with Figure 9 and Additional file 15: Figure S4). Additional file 18: Figure S6. Conservation of the C-rich region of S_RLK homologues. Alignement of the C-rich region of *Brassica oleracea* S_RLK6 (BoSRK6), ZmPK1, and the three *M. truncatula* proteins identified in this study (Mt238, Mt354, and Mt4055). Additional file 19: Table S8. Overview of all *Medicago truncatula* genes corresponding to proteins that showed a significant difference between non-mycorrhizal and AM-competent species. Included are also gene induction in LSM-dissected cortex cells with arbuscules (LCM), and the p-value for the comparison of the E-values from nonAM and AM-competent dicots (p-value Bra/Dic). For the different predicted *cis*-elements, the number of hits in the respective promoters is listed. Additional file 20: Table S9. Comparison with the list of conserved genes of Delaux et al. (2014) [87]. The list of 174 conserved AM-related genes (Additional file 20: Table S9 in Delaux et al., 2014 [87]) was compared with the list of AM-related proteins listed in Additional file 19: Table S8 (see notes added in proof). 50 common genes were identified in both lists. Rank numbers refer to the ranks in Additional file 7: Table S2.
