# Supplementary material for: A novel bioinformatics pipeline to discover genes related to arbuscular mycorrhizal symbiosis based on their evolutionary conservation pattern among higher plants
Source: BMC Plant Biol. 2014 Dec 3;14:333. doi: 10.1186/s12870-014-0333-0 (PMC4274732; doi:10.1186/s12870-014-0333-0)
Supplement: Additional file 1: File S1. — Protein sequences used for phylogenetic analysis of RAM1, PT4, RPL5, cyclinD6 (Figure 1), α-glucosidase/xylosidase (Figure 8), and SRK homologues (Additional file 11: Figure S5). [file 12870_2014_333_MOESM1_ESM.docx]

**Favre et al. - Supplemental File S1**

**Protein sequences used for phylogenetic analysis as shown in Figure 1, Figure 8, and Figure S5**

**RAM1**

**>M_truncatula (**AFK81971.2**)**

MINSLCGSSNSLKEKCLQPNSSNQTNTHSKKNATNSCGDLEQINVLTPQSLNLPSLKFDLDGDVEVQSPDSSMWEAFFNDHLDNDFMISSPIRNINPNSPQASTYNNCNYNYAQGMQIQSLSGCSPPRFASQIGSLNSNNQQKGKGLSPLHRVFNSPNNQYMQHVENLSLPAIEEFLEDFQGDVDHFSSTKVSSECFDMETPISTILDSLTMQNSSSYGASVNEESTLLHGGNSSSQISQESDIYHQMGSMASASLSQALQQERYQEKHQKMQAQQQSLTVPIQIGIEQEQDSGLQLVHLLLACAEAVAKGEYMLARRYLHQLNRVVTPLGDSMQRVASCFTESLSARLAATLTTKSSSTKKLAPSSLSSSSSSSCLSTFPSNPMEVLKIYQIVYQACPYIKFAHFTANQAIFEAFEAEERVHVIDLDILQGYQWPAFMQALAARPGGAPFLRITGVGPCIESVRETGRCLTELAHSLRIPFEFHPVGEQLEDLKPHMFNRRVGEALAVNTVNRLHRVPGNHLGNLLSMIRDQAPNIVTLVEQEASHNGPYFLGRFLEALHYYSAIFDSLDATFPVESAPRAKVEQYIFAPEIRNIVACEGEERIERHERLEKWRKIMEGKGFKGVPLSPNAVTQSRILLGLYSCDGYRLTEDKGCLLLGWQDRAIIAASAWRC

>R_communis (XM_002512850.1)

MINSLSGCMGSVKSMSNSSCTKTHQPTSANGSSSVFESKRTTLSSDLEHTSLTPPSLSFPAVKFELDGDVEVQSPDSSLWETFFSDHSDSDFMILSPVRNLPSPQTSTCNYNHVHAMQGQSFSGCSPPRHLYQLGSFSSSHKGKGQSPLHRVFNSPNNQYMQIESLSSLPGIEDFLDDYQRDGLVGGYQPTRLSSGTGNSASSELFDMQTAAGPPLLDCLSMPSSSVFGGSVSETSPSGSQLAQDRDIYHMESLRNAPPLSQQLQQEQQENQRQPPPSTTPPPPPPVATTPPERQQQNLNPSLMVPLPIGPEQEQDSGLQLVHLLLACAEAVAKEDYMLARKYLHHLNRVVTPLGDSMQRVASCFTEALSARLAATLTTQPSNTAPKPYSSYPSNSMEILKIYQIVYQACPYIKFAHFTANQAIFEAFEAEERVHVIDLDILQGYQWPAFMQALAARPGGAPFLRITGVGSCIESVRETGRCLTELAHSLHVPFEFHPVAEELEDLKPHMFNRRVGEALAVNSVNRLHHVPGNCLPNLLAMIRDQAPNIVTIVEKEASHNGPYFLGRFLEALHYYSAIFDSLDATFPPDSTQRAKVEQYIFAPEIRNIVACEGPERTERHERLEKWRKLMEGKGFKGVPLSANAVTQSKILLGLYSCDGYRLTEDKGCLLLGWQDRAILAASAWRC

>P_trichocarpa (XM_002298611.1)

MINALCGSMGSLKSENSCNKLQPTSPNESSSVTESKKTTQSFEFEQNSLTPTSLNLPAVKFELDGDVEVQSPDSSMWESFFTDNFDSDFMISSPVRNLPSPQTSSYNHNYAHAMQGQSLSGCSPPRYLSQLGAAFSSIHKGKGQSPLHRMCNSPNNQFMQVESLSLPGIEDFLDDFQRDGYGEGYQQPPKMSGSSGSSTQLFDMSTTVPAMLDCLTIQNPSRFCSGSVSETSSGSPMTQESDIFQMGSIGIAPSSQKLLQENQQQPQPLPQPTTQPQPPPTTQSLQQQPQSLNHTLMVSLPIGSEQEQDSGLQLLNLLLACAEAVSNEDYMLARRYLHHLNRVVSPLGDSMQRVASCFTEALSARLAATLTTKPSTSSSKAFSPFPPNSMEILKIYQILYQACPYVKFAHFTANQAIFEAFETEERVHVIDLDILQGYQWPAFMQALAARPGGAPFLRITGVGSSMENVRETGRCLTELAHSLHVPFEYHPVAEELVDLKPHMFNRRVGEALAVNSVNRLHRVPGNCLGNLLAMIRDQAPNIVTVVEQEASHNGPYFLGRFLEALHYYSAIFDSLDSTFPPDSSQRAKVEQYIFAPEIRNIVACEGAERFERHERLEKWRKLMEGKGFKGVPLSANAVTQSKILLGLYSCDGYRLTEDKGCLLLGWQDRAILAASAWRC

>V_vinifera (XM_002274798.2)

MINSLCGSMGSLNSESSCTKLQQPTSPNKSILESRKAPLSSDLESNSLAPSSLNLHGLKFDVDGDAEVQSPDSSMWEAFFTDHFDSDFMISSPVRSSPMNYNYNHVQSMQGQSLLGCSPPRNSSQLGSFSSTHKGKGQSPLHKVFNSPTYQYIQPDTLSLPPLDSLLDDYQRDGVGASSYPMMKIPAVGISGASSLAQYLDMPITIHPTALDLPLQNTWRFSGPVSESSATGGSQLTQEKDMYQMGSMSSTASLSQQLQEEHQQEQQLQQKQQQLQEIQNPNHGLMVPHPLGSEQEHDSGLQLVHFLLACAEAVAKEDYMLARRYLHHLNRVVTPLGDSMQRVASCFTEALSARLAATLTPKPSTSTTKPFNPFPPNSLEILKIYQILYQACPYIKFAHFTANQAIFEAFEAEERVHVIDLDILQGYQWPAFIQALAARPGGAPFLRITGVGCSPESVRETGRCLTELAHSLHVPFEFHPVGEELEDLKPHMFNRRVGEALAVNSANRLHRVPTNFLGNLLAMIRDQAPNIVTIVEQEASHNGPYFLGRFLEALHYYSAIFDSLDATFPPDSAQRAKLEQYIFAPVIRNIVACEGAERVMRHERLEKWRKLMEGKGFQGVPLSANAVTQSKILLGLYSCDGYRLTEDKGCLLLGWQDRAILAASAWRC

>C_sativus (XM_004138750.1)

MINSLCGSIGSRKTTNTTTDQTSSTCTTKQQHHLPHPTSPTDSVSAKTVPISSSDLEQTALTPPSLDFPAPKFDIDGDIEIQSPDNSVWDSLFADQLDCDFMISSPAPSLPSPQNLSFNYYNYNYGQAMMQCSPPRSCSQVGASSSVQKGKGLSPLHKVFNSPSNQYMQAIEGNNNSSNSIQTIGELLEDYQEEGFETYHQNMSKISGIGESLQYYDISTSSLPPIIFEDLALPNSSNIICGSNQEPSTVEREFYNQIGSSNITTASLPQQGDHQEQENPPQLPPPSLPLLPPPKQPQNQLNHSLMAPLPVGSEQEQDSGLQLVHLLLACAEAVAKEDYMLARRYLHHLNRVVTPIGDSMQRVASCFTEALTARLAATLTTSKPSSSIPPFPQNSLEILKIYQIVYQACPYVKFAHFTANQAIFEAFEAEERVHVIDLDILQGYQWPAFMQALAARPGGSPFLRITGVGPSIDAVRETGRCLTELAHSLNVPFEFHAIGEQLESLKPNMFNRRVGEALAVNAVNRLHRVPGKSLGNLLGMIRDQAPNIVTLVEQEASHNGPYFLGRFLEALHYYSAIFDSLDATFPPDSAQRAKVEQYIFAPEIRNIVACEGPERIERHERLEKWRKLMEAKGFKGVALSSNAVTQSKILLGLYSCDGYRLTEDKGCLLLGWQDRALIAASAWRC

>S_lycopersicum (XM_004233535.1)

MGSLKNDIIKNEVGSEITSFKDQNSYIKLLPEDSLASSESKKVTPISSDFELDCGSLIPTSLTFPPGGGDDDDVEIQSPDNSIWESFFADQLEADFMISSPVRNLSSTSTFCTTTTTHNNNTYTHHNQGIHGQSMMMCSPPRSPLRPNNYNSTNKGKGLSPFQKVFNSPNNQFMQIESFNLPALESFLDDDLASEYSTLKVSDVGSSSESLSVIPDFLECLALPNSSSNTSASFMGSLLSNTSVGQVDDEIFQTGSIAPLSQQLHQERHHEKQQKQIPTHVQLPSTQQQYTQMINHNLVVAAPDQEQDSGLQLVHLLLACAEAVSKEDYMLARRYLHHLNRVVTPIGDSMQRVASCFTEALTARLAATLATKPSTSVPKPFNPFPPNSLEILKIYQILYQACPYVKFAHFTANQAIFEAFEAEERVHVIDLDILQGYQWPAFMQALAARPGGAPFLRITGVGSYPEAVRETGRCLTELAQSLHVPFEFHPVGEQLEDLKPHMFNRRIGEALAVNSVNRLHRVPGNCIGNLLGMIRDQAPNIVTIVEQEASHNGPYFLGRFLEALHYYSAIFDSLDATFPGDSSQRAKLEQYIFGPEIMNIVSCEGMERMVRHERLEKWRRVMEGKGFKGVALSANAVTQSKILLGLYSCDGYKLTEDNGCLLLGWQDRAILAASAWRC

>P_axillaris (Peaxi162Scf00420g06006.1)

MGSLNNEVGSIKCEDGSLKSENSFTKLPSEESLASESKKVTPVSSDFELNCGSLAPTSLSFPAMKFEDDIEIQSPDNSIWESFFADHLEGDFMISSPIRNLPSPQPASSFSSSHNNSNNNNNNNYNIYAHRQGIHGQDMMMCSPPRSPLGLYNHPSHKGKGLSPLQKVFNSPNNQFMQIESFNLPALESFLDDDYDKEEDLASSYSTLKVSGTGVAGSSSESFDALSVVPDDLLECLALPSSLSNTSGGFMESLLSDTSAVQVINPDDEIFRTGSIAPLSQQLHQERQQEKQQKQRPVTHARPQQQQIINHNVLVPLAAAPDQEQDSGLQLVHLLLACAEAVSKEDYMLARRYLHHLNRVVTPLGDSMQRVASCFTEALSARLAATLATKPSTSARKPFNPFPPNSLEILKIYQILYQACPYVKFAHFTANQAIFEAFEAEERVHIIDLDILQGYQWPAFMQALAARPGGAPFLRITGVGPSPEAVRETGRCLTELAHSLHVPFEFHPVGEQLEDLKPHMFNRRVGEALAVNSVNRLHRVPVNCIGNLLAMIRDQAPNIVTIVEQEASHNGPYFLGRFLEALHYYSAIFDSLDATFPADSSQRAKLEQYIFAPEIMNIVSCEGQERNVRHERLEKWRRLMEGKGFKGVALSANAVTQSKILLGLYSCDGYKLTEDDGCLLLGWQDRAILAASAWRC

>S_italica (XM_004980464.1)

MGMLNCADTSSGAKLQQLQAPTSPTASVSESNIVVSSTDPDANDALAGLQALKFDGDIDVEIQSPDIAMWESLFAEQMGASGGDFLMSFSPRRDFTATGSPRRDFMVSSPKRDYMMSSPKRDYMVSSPKRDYMMSSPKREYMVTSPRRDSSPRRSTFSNLYSGTGSHQQGYVDGVHGAEGGSGSGGGQPLYGGLANHGKGKSQSPLHKVYINNAHSNGGRSTGPSSLSCSSSYGHGESLSLPSMDPFLDEYKEGGGYLAGYQLPVKTGMENGAASAATVTTVAPSPSQLPTLSECLAMPEPVYGGSEAAAAGGLQMGAGLPAELYYGGQFGGDGFTLQHQMAKSDQWAGDSSLHSMLGSVIQTEAEQEQDSGLQLVHLLLACADFVSKGDQPSALRHLHLLRRVASPLGDSMQRVASYFADALAARLSLSSNPSSSSSSSGAATPRGGAAAGVAPYTFPPSPETLKIYQILYQACPYIKFAHFTANQAIFEAFAGEDRVHVVDLDILQGYQWPAFLQALAARPGGPPTLRLTGVGHPAAAVRETGRHLASLAASLRVPFEFHAAAADRLERLRPAALQRRVGEALAVNAVNRLHRVPSAHLGPLLSMIRDQAPKIMTLVEQEAGHNGPYFLGRFLEALHYYSAIFDSLDATFPADSAPRMKVEQCLLAPEIRNVVACEGAERVARHERLDRWRRLMEGRGFEPVPLSPAAIGQSQVLLGLYGASDGYRLTEDKGCLLLGWQDRAIIAASAWQC

>S_bicolor (XP_002450783.1)

MGTTMLNCVDTSSSGAKLQQQQAPTSPTASVSESNIVVPSSADIDANDALASLQALRFDGDIDAEIQSPDIAMWESLFAEQMGASGGDFLMFSPRRDFMATGSPKRDFMVSSPKRDYMMSSPKRDYMMSSPRRDFMASSPKREYMVSSPRRDSSPRRSPFSANLFSTGGGGGGHQQGYAHGLDHQGSSAGAQALYGGLLVANNHGKGKSQSPLHKVYINNNAQSNSGKQSTAGPSSLSCSSSYGHGENDLSLPSMEPSFLDDYKYKEGGYLGYQQMLVGKQAADGGLIENGRRSSATVAPPSSSGQLPTLSECLAMPEPPAYVGAEEAVAAAAAAGLQMGLGLPSDLYYAGQFGGGDGLMTTLQHQMAKSDQWAAAADSSLHSMLGSVIQAEAEQEQDSGLQLVHLLLACADFVSKGDQPSALRHLHLLRRVASPLGDSMQRVASYFADALAARLTLSSNPSSCSSSGGVATPRGGAGAGVAPYTFPPSPDTLKIYQILYQACPYVKFAHFTANQAIFEAFHGEDRVHVVDLDILQGYQWPAFLQALAARPGGPPTLRLTGVGHPSAAVRETGRHLASLAASLRVPFEFHAAVADRLERLRPGALQRRVGEALAVNAVNRLHRVPGVHLGPLLSMIRDQAPKIMTLVEQEAGHNGPYFLGRFLEALHYYSAIFDSLDATFPADSAPRMKVEQCLLAPEIRNVVACEGAERVARHERLDRWRRLMEGRGFEPVPLSPAAVGQSQVLLGLYGAGDGYRLTEDKGCLLLGWQDRAIIAASAWRC

>B_distachyon (XP_003576038.1)

MGTLNCSDTTSSVKLQQQQGPTSPTASFSESNIVASSTDPDAIDALAGLQALRFDGDIDGEIQSPDLAMWESLFADQIGASGADFLMSSPRRDFSPLRDFMVSSPKRDYMVSSPKRDYMMSSPKRDYMMSSPKRDYMVSSPKREMGVSSPRRSTFSNLYSSTINQANQQSYMHGMEGSPQTQYSNLASQGNKGKSSPSPLHKVYINNVNAHSNSGKSNGPSSLSCSSSYAHGENLPLPSMDPFLEEYKEGYLAYQLPEKAGGSESARTTAPTSSQLPTLSECLAMPEPGYGDGDDDTAAAIVARAGIQVGGLQQTDHLYYASQFGAAEGSLSSLQHQMAKPEQWADSSSLHSMLGSVIQSEADQQQDSGLQLVHLLLACADLVSKGDQPSALRHLHLLRRVASPLGDSMQRVASYFADALAARLALACPSSVVSPGGAPFPFPPSPDTLKIYQILYQACPYIKFAHFTANQAIFEAFQGEDRVHVVDLDILQGYQWPAFLQALAARPGGPPTLRLTGVGHPAAAVRETGRHLASLAASLRVPFEFHAAVADKLERLRPAALQRRVGEALAVNAVNRLHRVPGAHLAPLLSMIRDQAPKIMTLVEQEAGHNGPYFLGRFLEALHYYSAIFDSLDATFPADSAPRMKVEQCLLAPEIRNVVACEGAERVARHERLDRWRRIMEGRGFEAVPLSPAAVGQSQVLLGLYGAGDGYRLNEDKGCLLLGWQDRAIIGASAWRC

>Z_mays (AFW60842.1)

MGMLNCVDTTSGAGAKLQHQQQAPTSPTASVSESNIVAPSVSADIDANDALASLQALRFDGDIDVEIQSPDIAMWESLFAEQMGAAGGDFLMLSPRRDFMAAGSPSRRDFMVSSPKRDYMVSSSPKRDYMMSSPMRDLMASSPKREYMVSSPRRDSSSSRRSPFSPNLFSTSGGGHQHGYAAHGPTDGGVGGAGQPLYGGLANHHGKGKSQSPLHKGYINNSGNKQSTGPSSLSCSSSYGHADNDLPSMDTCFLDDYKDGGYIGYQQQMPGKQQAAPGIMVNNNGGCSTAVTTVGVAPSPSSHQLPTLSECLAMPEPAFVGGEEAAAGGLQMGVGLPSDLYYAGQFAGGGGGGLTTTSSSLQHQMAKSDHQWAAAESSLHSMLGSVIQTEADEQEQDSGLQLVHLLLACADFVSKGDQPSALRHLHLLRRVASPLGDSMQRVASYFADALAARLSSNNPSSSAGAGAGAGVAPYTFPPSPDTLKVYQILYQACPYIKFAHFTANQAIFEAFHGEDRVHVVDLDILQGYQWPAFLQALAARPGGPPTLRLTGVGHPAAAVRETGRHLASLAASLRVPFEFHAAVADRLERLRPAALHRRVGEALAVNAVNRLHRVPAVHLGPLLSMIRDQAPKIMTLVEQEAGHNGPYFLGRFLEALHYYSAIFDSLDATFPADSAQRMKVEQCLLAPEIRNVVACEGAERVARHERLDRWRRIMEGRGFEPVPLSPAAVAQSQVLLGLYGAGDGYRLTEDRGCLLLGWQDRATIAASAWRC

>A_lyrata (XP_002887024.1)

MKREHNHRESSTGEGGSSSMTTVIKEEAAGVDELLVVLGYKVRSSDMADVAHKLEQLEMVLGDGISNLSDETVHYNPSDLSGWVESMLSDLDPARNQEKPDSEYDLRAIPGSAVYPREEHVTRRNKRTRIESELSSTRSVVVLDSQETGVRLVHALLACAEAVQQTNLKLADALVKHVGLLASSQAGAMRKVATYFAEGLARRIYRIYPRDDVALSSFSDTLQIHFYESCPYLKFAHFTANQAILEAFATAEKVHVIDLGLNHGLQWPALIQALALRPNGPPDFRLTGIGSSLTDIQEVGWKLGQLASTIGVNFEFKSIALNHLSDLKPEMLDIRPGSESVAVNSVFELHRLLAHPGSIDKFLSTIKSIRPNIMTVVEQEANHNGANFLDRFTESLHYYSSLFDSLEGPPSQDRVMSELFLGRQILNLVACEGEDRVERHETLNQWRNRFGSRGFKPVNIGSNAYKQASMLLALYAGADGYNVEEDEGCLLLGWQTRPLIATSAWRINLVE

>A_thaliana (CAA12242.1)

MEEVSSEMEVEVQNRQLSDSSPAQNVKKFGLKNSIQTNFGSDYVFQIVPKIDWTAIAVSLSTNTVKLYSPVTGQYYGECKGHSDTVNQIAFSSDSAASPHVLHSCSSDGTIRSWDTRSFQQVSRIDTGNDQEIFSFSYGGAADNLLAGGCKEQREHNHRESSAGEGGSSSMTTVIKEEAAGVDELLVVLGYKVRSSDMADVAHKLEQLEMVLGDGISNLSDETVHYNPSDLSGWVESMLSDLDPTRIQEKPDSEYDLRAIPGSAVYPRDEHVTRRSKRTEIESELSSTRSVVVLDSQETGVRLVHALLACAEAVQQNNLKLADALVKHVGLLASSQAGAMRKVATYFAEGLARRIYRIYPRDDVASSSFSDTLQIHFYESCPYLKFAHFTANQAILEVFATAEKVHVIDLGLNHGLQWPALIQALALRPNGPPDFRLTGIGYSLTDIQEVGWKLGQLASTIGVNFEFKSIALNNLSDLKPEMLDIRPGLESVAVNSVFELHRLLAHPGSIDKFLSTIKSIRPDIMTVVEQEANHNGTVFLDRFTESLHYYSSLFDSLEGPPSQDRVMSELFLGRQILNLVACEGEDRVERHETLNQWRNRFGLGGFKPVSIGSNAYKQASMLLALYAGADGYNVEENEGCLLLGWQTRPLIATSAWRINRVE

>E_salsugineum (XP_006391387.1)

MKREHNHQESSGGKVGTSKEEAGGFDELLVVLGYKVRSSDMADVAHKLEQLEMVLGDGISNLSDDTVHYNPSDISGWVETLISDLNPTRNQEKPDLDSEYDLSAIPGSAAYPRGKRTRMGSDSSTTRPMVVMDSQETGVRLVHALLACAEAVQQNNFKLADALVKHVGLLASSQAGAMRKVATYFAEGLARRIYRIYPRDDIGLSSFSDTLQVHFYESCPYLKFAHFTANQAMLEAFATVEKVHVIDLGLNQGLQWPALIQALAVRPNGPPDFRLTGIGSSLTGESIQEVGWKLGQLASTIGVNFEFKSIVLNNLSDLKPEMIEIRPGLESVAVNSVFELHRLLANSGSIEKFLSAIKLIEPDIMTVVEQEANHNGANFLDRFTESLHYYSSLFDSLEGPPSQDRVMSELYLGRQILNLVACEGEDRVERHETLNQWRNRLGSSGFKPVNIGSNAFKQASMLLALYAGADGYKVEENEGCLLLGWQTRPLIATSAWRFNRVE

>C_rubella (XP_006302161.1)

MKRELNHRESSAGEGGSSPMTTVIKEEAAGVDELLVVLGYKVRSSDMADVAHKLEQLEMVLGDGISNLCDETVHYNPSDLSGWVESMLSDLDPTRSQEKPGSEYDLRAIAGSAVYPREEHVSRRSKRTRVEPEMSTTRSVVVLDSQETGVRLVHALLACAEAVQQNNLKLADALVKHVGLLASSQAGAMRKVATYFAEGLARRIYRIYPRDDDVALSDTLQIHFYESCPYLKFAHFTANQAILEAFAMADKVHVIDLGLNHGLQWPALIQALALRPNGPPDFRLTGIGYSLTELQEVGWKLGQLASTIGVNYEFKSIALNNLSDLKPEMLDIRSGSESVAVNSVFDLHRLLAHPGSIDKFLSTIRSIRPDIMTIVEQEANHNGTVFLDRFTESLHYYSSLFDSLEGSPSQDRVMSELFLGRQILNLVACEGEDRVERHETLNQWRNRFGSGGFKPVNIGSNAYKQASMLLALHAGADGYSVDENEGCLLLGWQTRPLIATSAWSL

>B_rapa (Q5BN22.1)

MKRDLHQFQGPPDTRFPNHGTANTGSSSKDKMMMVKEEEDGGNMDELLAVLGYKVRSSEMAEVALKLEQLETMMGNVQEDGLSNLATDTVHYNPSELYSWLDNMLTEFNPPPPEINNSFLAGAGGSDYDLKAIPGNAIYARSDQFAIDSSSSSNQAGDNSQSTKRLKSCSSPDSLVTGTTVTTTTTESTRSVGLAAESTRSMVLVDSQENGVRLVHALMACAEAIQNNDLSIAEALVKQIGFLAVSQAGAMRKVATYFAEALARRIYRLSPPQTQIDHSLSDTLQMHFYETCPYLKFAHFTANQAILEAFEGKKRVHVIDFSMNQGLQWPALMQALALREGGPPVFRLTGIGPPAADNSDHLHEVGCKLAQLAEAIHVEFEYRGFVANSLADLDASMLELRPSEIEAVAVNSVFELHKLLGRTGGIEKVLGVVKQIKPVIFTVVEQESSHNGPVFLDRFTESLHYYSTLFDSLEGVPSSQDKVMSEVYLGKQICNLVACEGPDRVERHETLSQWANRFGSSGFAPAHLGSNAFKQASMLLALFNGGEGYRVEENNGCLMLGWHTRPLITTSAWKLSAAH

**Phosphate transporter 4**

>P_hybrida (ACB37441.1)

MASDNLVVLNALDTARTQWYHVTAVIIAGMGFFTDAYDLFCISTVSKLLGRLYYYDPSTKAPGKLPHMANNWVIGVALVGTLSGQLVFGWLGDKLGRKKVYGLTLILMVICALCSGLSLGYSPKSVIGTLCFFRFWLGFGIGGDYPLSATIMSEYANKSTRGAFIAAVFAMQGVGIIFAGLVSMTISKVFLMNFEGKPFNVDEVLSTEPEADYVWRIVLMLGALPALLTYYWRMKMPETGRYTAIIEGNAKQAAIDMGKVLDIEIQAEGDKLAQFKAANEYSLLSNEFFQRHGLHLIGTMSTWFLLDIAFYSQNLTQKDIFPVMGLTSKANTISALREMFETSRAMFVIALFGTFPGYWFTVFFIEKIGRFKIQLVGFFMMSVFMAIIGVKYDYLRNKEHKWTFAALYGLTFFFANFGPNSTTFVLPAELFPTRVRSTCHALSAALGKAGAMISAFGIQQYTQDQDVRKIKTAMLLLAFTNMVGFCCTFLVTETKGRSLEEISGEDGRQNETQMKTTRPVSGHPDDGWE

>S_lycopersicumn (AAV97730.2)

MIIHQPISSNTRKSNNMASDNLVVLNALDTARTQWYHVTAVIIAGMGFFTDAYDLFCITTISKLLGRLYYYDPTTHAPGKLPHVANNWVIGVALVGTLSGQLVFGWLGDKLGRKKVYGLTLILMVLCALCSGLSLGYSAKGVIGTLCFFRFWLGFGIGGDYPLSATIMSEYANKATRGAFIAAVFAMQGVGIIFAGLVSMIISKLFLMRYEGEPFNVDEILSTEPQADYVWRIVLMLGALPALLTYYWRMKMPETGRYTAIIEGNAKQAAINMGKVLDIEIQAESDKLAQFKAANEYSLLSNEFFQRHGLHLIGTMSTWFLLDIAFYSQNLTQKDIFPVMGLTSNANTISALREMFETSRAMFVIALFGTFPGYWFTVFFIEKIGRFRIQLMGFFMMSVFMAIIGVKYDYLKSKEHKWTFATLYGLTFFFANFGPNSTTFVLPAELFPTRVRSTCHALSAASGKAGAMISAFGIQQYTQDGNVHKIKTAMILLAVTNMAGFCCTFLVTETKGRSLEEITGEDGGQNETQMKTSKPVSGHQDDGWE

>S_tuberosum (AAW51149.1)

MASDNLVVLNAPDTARTQWYHVTAVIIAGMGFFTDAYDLFCITTISKLLGRLYYYDPTTHAPGKLPHVANNWVIGVALVGTLSGQLVFGWLGDKLGRKKVYGLTLILMVLCAICSGLSFGYSAKGVIGTLCFFRFWLGFGIGGDYPLSATIMSEYANKATRGAFIAAVFAMQGVGIIFAGLVSMIISKLFLMKYEGKPFDVDEILSTEPEADYVWRIVLMLGALPALLTYYWRMKMPETGRYTAIIEGNAKQAAINMGKVLDIEIQAEGDKLAQFKAANEYSLLSNEFFQRHGLHLIGTMSTWFLLDIAFYSQNLTQKDIFPVMGLTSKANTISALREMFETSRAMFVIALFGTFPGYWFTVFFIEKIGRFRIQLMGFFMMSVFMAIIGIKYDYLKTKEHKWTFATLYGLTFFFANFGPNSTTFVLPAELFPTRVRSTCHALSAASGKAGAMISAFGIQQYTQDGNVHKIKTAMILMAVTNMVGFCCTFLVTETKGRSLEEISGEDGGKNETQMKTSKPVSGHQDDGWE

>C_clementina (XP_006422114.1)

MSNNKLAVLNALDTARTQWYHVTAIVIAGMGFFTDAYDLFCISTVSKLLGRLYYYDPANPKHNPGKLPTPINNFVIGVALVGTLTGQLFFGWLGDKLGRKKVYGITLILMVICAICSGLSFGSSARSVIGTLCFFRFWLGFGIGGDYPLSATIMSEYANKKTRGAFIAAVFAMQGVGIIFAGLVSMILSKIFLQLFPAPAFEDDEIFSTQPEGDYLWRIVLMLGALPALTTYYWRMKMPETGRYTAIIEGNAKQAASDMGRVLDIEIDEEHDKLSQFKEVNKYPLLSREFFQRHGLHLIGTMSTWFLLDIAFYSQNLTQKDIFPAMGLVHKAPQVNALREVFETSRAMFVVALLGTFPGYWFTVFLIEKIGRFIIQLVGFFMMSLFMLIIGIKYDYLKNDNKWLFATLYGLTFFFANFGPNSTTFVLPAELFPTRVRSTCHALSAAAGKAGAMIGAFVVQSYTLDEDSKKIQHAVLVLAFTNMLGFCCTFLVTETKGRSLEEISGEDGGVGYYHDTEMSSRPTGARQSGRMEAI

>V_vinifera (XP_002267369.1)

MSDGLAVLHALDSARTQWYHITAIVIAGMGFFTDAYDLFCISTVSKLLGRLYYYDPTKDKPGKLPHGVNNFVIGVALVGTLSGQLVFGWLGDKLGRKKVYGMTLILMSICAICSGLSFGFSNKSVITTLCFFRFWLGFGIGGDYPLSATIMSEYANKKTRGAFIAAVFAMQGVGIVFAGLVSMILSKLFLLKYETVPFSEEPILSTQPEADYLWRIVLMLGALPALLTYYWRMKMPETGRYTALIEGNAKQAAADMGRVLEIEIQAEADKVAEFKAANEYSLWSREFFDRHGRHLIGTMSTWFLLDIAFYSQNLTQKDIFPAMNLVKKDYEVSALREMFETSRAMFVVALLGTFPGYWFTVFFIDRIGRFIIQLVGFFMMSLFMLIIGIKYEYLRDDNKWLFAVLYGLTFFFANFGPNSTTFVLPAELFPTRVRSTCHAMSAAAGKAGAMIGAFVVATYTLDGKANEIRIAMITMACTNMLGFFCTFLVTETKGRSLEEISGEDGGINETEMASRPS

>P_persica (XP_007219848.1)

MALAVLNALDSARTQWYHVTAIVIAGMGFFTDAYDLFCISTVSKLLGRLYYFNPDSKVPGKLPPRVNNIVIGVALVGTLSGQLVFGWLGDKLGRKKVYGVTLIMMVICAICSGLSFGSSAPAVMTTLCFFRFWLGFGIGGDYPLSATIMSEYANKMTRGAFIAAVFAMQGVGIIFAGLVSMVLSRIFLSFYPAPAFHDSPTDYAHVLSTQPQADYLWRIVLMIGALPAIVTYYWRMKMPETGRYTALIEGNAKQAAADMGKVLDIEIQAEQEKLAQFKAANEYPLLSMEFYRRHGRHLIGTMTTWFLLDIAFYSQNLTQKDIFPVMGLTHKDVEVNALQEMFETSRAMFVIAFFGTFPGYWFTVFFIEKLGRFKIQLVGFFMMSFFMLIIGIKYDYLTTNKYMFATLYGLTFFFANFGPNSTTFVLPAELFPTRVRSTCHALSAAAGKAGAMVGAFGVQNYTLGKGTKDIQKAMFFLAFTNMLGFCFTFLVTETKGRSLEEISGEDGSGETAGTQMTGTNSSSVTR

>T_cacao (XP_007038944.1)

MASNNNLAVLEALDTARTQWYHIKAIVIAGMGFFTDAYDLFCITTVSKLLGRLYYYDPAHSDRPGKLPHYVNNLVTGVALFGTLSGQLVFGWLGDKLGRKKVYGITLILMVICAICSGLSFGSTADSVIGTLCFFRFWLGFGIGGDYPLSATIMSEYANKKTRGAFIAAVFAMQGVGIIFAGLVSMILSAIFRKRYPAPSFKADAIFSTQPEADFLWRIVLMFGALPALLTYYWRMKMPETGRYTALIEGNAKQAAADMGRVLDIELDAEGEKLSQFKAANQFPLLSHEFYMRHGRHLFGTMSTWFLLDIAFYSQNLTQKDVFPAMGLTRKAPDINAIEEVYETSRAMFIVALLGTFPGYWFTVLFIEKIGRFIIQLVGFLMMSIFMLLLGIKYDYLRDENKLLFVVLYVLTFFFANFGPNSTTFVLPAELFPTRLRSTCHALSAASGKAGAVIGAFVVQSYTLDESVGKIKRAIMVLAFTNLLGFCFTFLVPETKGRSLEEISGEDGGAQNETQMAARRSATIKGSGRLEVI

>L_japonicus (BAG71408.1)

MALEVLEALDSARTQWYHVTAIVIAGMGFFTDAYDLFCITTVSKLLGRLYYFDPSTGKPGKLPNNVNNLVTGVALVGTLSGQLFFGYLGDKLGRKKVYGVTLILMVACAICSGLSFGASAKSVMGTLCFFRFWLGFGIGGDYPLSATIMSEYANKRTRGAFIAAVFAMQGVGIIFAGLVSMCLSAGFKASYHAPSFHDDPIMSTQPQGDLMWRLVLMIGAVPAAMTYYWRMKMPETGRYTAIIEGNAKQAAADMARVLDIEIQAEQDKLAEFKAANDYPLWSNEFFTRHGRHLIGTMTSWFLLDIAFYSQNLTQKDIFPAMGLIDKDFEMNAIQEVFETSRAMFVIALFGTFPGYWFTVFFIEKLGRYKIQLIGFFMMSVFMFIIGVKYDYLRNENSHMFALLYGLTFFFANFGPNSTTFVLPAELFPTRVRSTCHALSAAAGKAGAMVGAFGIQNYTQKGEQKQIKHAMMILAVTNLIGFFCSFLVTETKGRSLEEISGEDGRESELTPTPPNNRVPTRQEPRSETM

>C_sativus (XP_004149464.1)

MAIAVLDALDNARTQWYHITAIVIAGMGFFTDAYDLFCISTVSKLLGRLYYFNPDSTKPGKLPNQINNAVVGVALVGTLMGQLFFGWLGDKLGRKKVYGVTLVLMALCAICSGLSFGSTSKSVIGTLCFFRFWLGFGIGGDYPLSATIMSEYANKKTRGAFIAAVFAMQGMGLIFAGLVSMILSKIFLSLHKAPSYKAEPVFSTQPEGDFLWRIVLMLGALPAILTYYWRMKMPETGRYTALIEGNAKQAAADMGKVLEIQIQAEQEKLANFKSANEYGLLSKEFFDRHGLHLIGTTTTWFLLDIAFYSNNLTQKDIFPAMNLTKKPETVSALEEVYETSKAMFLVALLGTFPGYWFTVFLIEKLGRFKIQLIGFFMMSVFMAVLGVRYNYMKNHPVEFAIIYGLTFFFANFGPNSTTFVLPAELFPTRVRSTCHALSAASGKAGAIVGAFGVQNYTLDGNPSKIQKAMIFLAFTNMLGFVFTFLVTETKGRSLEEISGEDGGSAGENETQMPVRSMGKGHEMEGQNV

>F_vesca (XP_004309163.1)

MAIAVLAALDNAKTQWYHMTAIVIAGMGFFTDAYDLFCISTVSKLLGRLYYYDPSKGAPGKLPQGINNLVVGVALVGTLSGQLFFGYLGDKLGRKRVYGVTLIMMAVCAICSGLSFGSSPSAVMTTLCFFRFWLGFGIGGDYPLSATIMSEYANKTTRGAFIAAVFAMQGVGIIFAGLVSMTLSAIFLRRYPAPAFNANPTDYSDVLSTQPEADFLWRIVLMLGALPAVVTFYWRMKMPETARYTALIEGNAKQAATDMGHVLEIEIQAEQDKVAQFKAANEYKLLSGEFFRRHGKHLIGTMTTWFLLDIAFYSQNLTQKDIFPVMGLTDKPKTVNALMEVFQTSRAMFVVALLGTFPGYWFTVAFIEKLGRYKIQLVGFFMMSVFMFIIGIKYEWLINNPPVFATLYGLTFFFANFGPNSTTFVLPAELFPTRVRSTCHAISAASGKAGAMVGAFGIQNLTLDGKPKSIRKAMMILAFTNMLGFCFTFMLTETKGRSLEEISGEDGGNGGKNETQMSDDD

>P_trichocarpa (XP_006374329.1)

MSSSNLAVLNALDNARIQLYHVTAIIIAGMGFFTDAYDLFCITTVSKLLGRLYYYDPITGNPGKLPTNVNNVVTGVALVGTLSGQLVFGWAGDKLGRKKVYGVTLIIMVICAIGSGISFGSSTKSVIGTLCFFRFWLGFGIGGDYPLSATIMSEYANKKTRGKFIAAVFAMQGVGIIFAGLVSMILSKIFLSRYHAVPFSKDPILSTQPQADFLWRIVLMLGALPAMLTFYWRMKMPETGRYTALIEGNAKKAAVDMGRVLDIDIQEESDKLSEIRASNNYKLLSWEFFDRHGYHLIGTMSTWFLLDIAFYSQNLTQKDIFPTMGLTKQAADVSALEEVYETSRAMFIVALLGTFPGYWFTVLFIESLGRFFIQVMGFIMMSSFMLLMGVFYDGLKEHKWLFALLYGLTFFFANFGPNSTTFVLPAELFPTRLRSTCHALSAAAGKAGAMIGAFVVQTYTLDGDVTKIKRALLALSFTNILGACFTFFLSETKGKSLEEISGEDG

>M_truncatula (AAM76743.1)

MGLEVLEALDSARTQWYHVTAIVIAGMGFFTDAYDLFCISTVSKLLGRLYYFDPSTNKPGKLPPSVNNVVTGVALVGTLSGQLVFGWLGDKLGRKKVYGVTLIIMVACAICSGLSFGSSAKSVMITLCFFRFWLGFGIGGDYPLSATIMSEYANKRTRGAFIAAVFAMQGVGIIFAGLVSMVFSGIFKAYYQAPRFNEDPILSTQPEGDLLWRLILMIGAVPAAMTYYWRMKMPETGRYTAIVEGNAKQAAADMARVLDIEIIAEQDKLAEFKAANDYPLWSSEFFNRHGRHLIGTMSCWFLLDIAFYSQNLTQKDIYPAMGLIRQDKEMNAIDEVFQTSRAMFVVALFGTFPGYWFTVFFIEKLGRFKIQLVGFFMMSFFMFVIGVKYEYLKDENKNLFALLYGLTFFFANFGPNSTTFVLPAELFPTRVRSTCHAFSAASGKAGAMVGAFGIQYYTLDGTPRKIRRAMMILAFTNLIGFFCTFLVTETKGRSLEEISGEDGRESELTATPNDRAPGIRQDSRTEKM

>O_sativa (NP_001043759.1)

MADADGGSNLAVLDALDSARTQMYHMKAIVIAGMGFFTDAYDLFCISTVSKLLGRLYYQPDGSTDSKPGALSKTANNMVIGVALVGTLMGQLVFGYFGDKLGRKRVYGVTLILMAACAIGSGLSFGSSRKAVIGTLCFFRFWLGFGIGGDYPLSATIMSEYSNKKTRGAFIAAVFAMQGVGIIFAGLVSMIVSSIFLTYNKAPSYKGNHDLSRQMPAADYVWRIVLMIGAFPALATFYWRMKMPETARYTAIIDGNAKQAANDMQKVLSIEIEAEQEKLAKFNAANNYPLLSMEFARRHGLHLIGTTTTWFLLDIAFYSQNLTQKDIFPAMGLISGAAEVNALTEMFQISKASFLVALLGTFPGYWVTVALIDKMGRYMIQLIGFFMMSMFMLAMGILYDYLKTHHFLFGLLYALTFFFANFGPNSTTFVLPAELFPTRVRSTCHAISAAAGKAGAIVAAFGIQKLTYNSQVKSIKKALIILSITNMLGFFFTFLVPETMGRSLEEISGEDGNTGAGGGGAPAAANAGVGVSASDVSRDEKFPASSTEWQTSMHA

>B_distachyon (XP_003569484.1)

MAENESGGQNLAVLEALDSARTQMYHMKAIVIAGMGFFTDAYDLFCISTVSKLLGRIYYPLQNIDQGKPGTLPVNVNNMVIGVALVGTLMGQLVFGYYGDKLGRKRVYGITLVLMAACAIGSGLSFGYTHRAVIGTLCFFRFLLGFGIGGDYPLSATIMSEYANKKTRGAFIAAVFAMQGVGIIFAGLVSMIVSGLFLHYNPAPTWSKVDPTLSNQTPAADYVWRIVLMIGAFPALATFYWRMKMPETARYTAIIEGNAKQASNDMQKVLEIQIDDEQDKLAKFRAANEYPLLSKEFARRHGMHLIGTTTTWFLLDIAFYSQNLTQKDIFPAINLTDPPETMNALKEMFVISRAMFLVALLGTFPGYWVTVAVIDKMGRYLIQLLGFFMMSVFMLVMGVKYEYLKDHNHLLFAVLYALTFFFANFGPNSTTFVLPAELFPTRVRSTCHAISAASGKAGAIVAAFGVQRLTLKGDVKNITRALIILSVTNMLGFFFTFLVPETMGRSLEEISGEDGNTGNGAGAGAVSMSAADVSKDGKFPASSTEWQQPSMQA

>S_italica (XP_004969360.1)

MADAPAAGPNLAVLDALDSARTQMYHMKAIVIAGMGFFTDAYDLFCISTVSKLLGRLYYPFDNLNDSDASKSKPGTLPMSVNNMVVGVALVGTLVGQLVFGYFGDKLGRKRVYGITLVLMAACAIASGLSFGSSPSAVIGTLCFFRFWLGFGIGGDYPLSATIMSEYSNKKTRGAFIAAVFAMQGVGIIFAGLVSMIVSGLLLHYHPAPSFEEAIQQNPKDPRASNQWPAADYMWRIVLMIGAVPAVVTFYWRMKMPETARYTALIEGNAKQAASDMEKVMDVEIQAEQEKLARYKAANDYPLLSVEFARRHGMHLLGTATTWFLLDIAFYSQNLTQKDIFPAIHLTSPAKEINALTEVFQISKAMFLVALLGTFPGYWVTVALIDKMGRYLIQLIGFFMMSAFMLAMGIMYESLRNKRTTLFAFLYALTFFFANFGPNSTTFVLPAELFPTRVRSTCHAISAASGKAGAIVAAYGVQRLTLTDDVKYIKMALIILSITNMLGFAFTFLVPETMGRSLEEISGEDGNVGTGSGAPAGPGMGAADVSRDDKMPVSSTEWQSSMHA

>H_vulgare (BAM62781.1)

MAENGAAGGGGGQNLAVLDALDSARTQMYHMKAIVIAGMGFFTDAYDLFCITTVSKLLGRLYYPDTNLGKNPPMPGTMPVRINNMVTGVALVGTLMGQLVFGYFGDKLGRKRVYGITLVLMAACAIGSGLSFGRDPGAVIGTLCFFRFWLGFGIGGDYPLSATIMSEYANKKTRGAFIAAVFAMQGVGIIFAGLVSMIVSGIFLHYNPAPTWKEDKYYSVQDQKPAADYMWRIVLMLGAFPAVATFYWRMKMPETARYTALIEGNAKQATNDMQKVLEIRIDEEQEKVAKFRAANEYSLLSMEFARRHGLHLIGTTTTWFLLDIAFYSQNLTQKDIFPAIKLTGGADTMNALREVFVISRAMFLIALFGTFPGYWVTVALIDKMGRYLIQLLGFFMMSLFMLVMGIKYEYLKSNGHALFAILYALTFFFANFGPNSTTFVLPAELFPTRVRSTCHAISAASGKAGAIVAAFGVQTLTLKGDPKHMKQALIILSVTNMLGFFFTFLVPETMGRSLEEISGEDGNVAGAAAGHVDKDVEKAPPSSTEWQPPSSMNA

>Z_mays (NP_001105776.1)

MAAPGGSNLAVLDALDSARTQMYHMKAIVIAGMGFFTDAYDLFCISTVSKLLGRIYYPDDNLYIDKPKPGTLPVSVNNMVTGVALVGTLMGQLVFGYFGDKLGRKRVYGITLVLMAACAIGSGLSFGSSAHAVIGTLCFFRFWLGFGIGGDYPLSATIMSEYSNKKTRGAFIAAVFAMQGVGIIFAGLVSMIVSGILLHYHPAPAWKENHDRSWQDQMPAADYMWRIVLMIGAFPALATFYWRMKMPETARYTALIEGNAKQAANDMQKVMDVEIQAEQDKLARYKAANDYPLLSREFARRHGLHLIGTATTWFLLDIAFYSQNLTQKDIFPAIKLTSPVDDINALKEVFEISKAMFLVALLGTFPGYWVTVALIDKMGRYLIQLIGFFMMSVFMLLMGVMYNDLKNKHTTLFALFYALTFFFANFGPNSTTFVLPAELFPTRVRSTCHAISAASGKAGAIVAAFGVQSLTLKGDVGHIKKALIILSVTNILGFFFTFLVPETMGRSLEEISGEDGNVENGPGAPAGVAMGVADVSKDDKMPVSSTEWQSSMHA

>S_bicolor (XP_002458253.1)

MAAEGGSNLAVLDALDSARTQMYHMKAIVIAGMGFFTDAYDLFCITTVSKLLGRIYYPNDNLYLDKPKPGTLPVSTNNLVTGVALVGTLMGQLVFGYFGDKLGRKRVYGITLVLMAACAIGSGLSFGSTRHAVIGTLCFFRFWLGFGIGGDYPLSATIMSEYSNKKTRGAFIAAVFAMQGVGIIFAGLVSMVISGILLRYNPAPSWTEDHDSSLGDQLPAADYMWRIVLMLGAFPALATFYWRMKMPETARYTALIEGNAKQAANDMQKVMDVEIQSEQDKLARYKAANDYPLLSREFAQRHGLHLIGTATTWFLLDIAFYSQNLTQKDIFPAIKLTSPAGDINPLKEVFEISKAMFLVALLGTFPGYWVTVALIDKMGRYLIQLIGFFMMSVFMLLMGIMYDDLKNKYTTLFALFYALTFFFANFGPNSTTFVLPAELFPTRVRSTCHAISAASGKAGAIVAAFGVQSLTLKGDIASIKKALIILAVTNMLGFFFTFLVPETMGRSLEEISGEDGNAGNGPGVPAGAAMGAADVSKDDKIPVSSTEWQSSMQA

>A_thaliana (AAL49927.1)

MAEQQLGVLKALDVAKTQLYHFTAIVIAGMGFFTDAYDLFCVSLVTKLLGRIYYFNPESAKPGSLPPHVAAAVNGVALCGTLSGQLFFGWLGDKLGRKKVYGLTLVMMILCSVASGLSFGHEAKGVMTTLCFFRFWLGFGIGGDYPLSATIMSEYANKKTRGAFIAAVFAMQGVGILAGGFVALTVSSIFDKKFPAPTYAVNRALSTPPQVDYIWRIIVMFGALPAALTYYWRMKMPETARYTALVAKNIKQATADMSKVLQTDIELEERVEDDVKDPKQNYGLFSKEFLRRHGLHLLGTTSTWFLLDIAFYSQNLFQKDIFSAIGWIPKAATMNATHEVFRIARAQTLIALCSTVPGYWFTVAFIDTIGRFKIQLNGFFMMTVFMFAIAFPYNHWIKPENRIGFVVMYSLTFFFANFGPNATTFIVPAEIFPARLRSTCHGISAAAGKAGAIVGAFGFLYAAQSQDKAKVDAGYPPGIGVKNSLIMLGVLNFIGMLFTFLVPEPKGKSLEELSGEAEVSHDEK

>A_lyrata (XP_002877995.1)

MAGDQLNVLNALDVAKTQWYHFTAIIIAGMGFFTDAYDLFCISLVTKLLGRIYYHVDGAEKPGTLPPNVSAAVNGVAFCGTLAGQLFFGWLGDKLGRKKVYGMTLMVMVLCSIASGLSFGSDPKTVMTTLCFFRFWLGFGIGGDYPLSATIMSEYANKKTRGAFIAAVFAMQGFGILTGGIFAIIVAAAFEAKFPAPIYKVDALASTVPQADYVWRIILMVGALPAAMTYYSRSKMPETARYTALVAKDAKLAASNMSKVLQVEIEAEQQRSEDKSNSFGLFSKEFMKRHGLHLLGTTSTWFLLDIAFYSQNLFQKDIFSAIGWIPPAQTMNAIQEVFKIARAQTLIALCSTVPGYWFTVAFIDVIGRFAIQMMGFFFMTVFMFALAIPYDHWTHKENRIGFVAMYSLTFFFANFGPNATTFVVPAEIFPARFRSTCHGISAASGKLGAMVGAFGFLYLAQSPDKTKTEHGYPPGIGVKNSLIVLGVVNLLGMVFTLLVPESKGKSLEEMSGENEQNDESSSNNNSNNAVSTA

>C_rubella (XP_006292723.1)

MAGDQLNVLNALDVAKTQWYHFTAIIIAGMGFFTDAYDLFCISLVTKLLGRIYYHVDGAEKPGTLPPNVSAAVNGVAFCGTLAGQLFFGWLGDKLGRKKVYGMTLMVMVLCSVASGLSFGSDPKTVMTTLCFFRFWLGFGIGGDYPLSATIMSEYANKKTRGAFIAAVFAMQGFGILTGGIFAIIVAAAFEAKFPSPAYQVDALASTVPQADYVWRIILMVGALPAAMTYYSRSKMPETARYTALVAKDAKLAASNMSKVLQVEIEADQQKSEDKSNSFGLFSKEFMKRHGLHLLGTTSTWFLLDIAFYSQNLFQKDIFSAIGWIPPAQTMNAIQEVFKIARAQTLIAMCSTVPGYWFTVAFIDVIGRFAIQMMGFFFMTVFMFALAIPYDHWTHKDNRIGFVAMYSLTFFFANFGPNATTFVVPAEIFPARFRSTCHGISAASGKLGAMVGAFGFLYLAQSPDKNKTEHGYPPGIGVKNSLIVLGVVNLLGMVFTLLVPESKGKSLEEMSGENEQHDEISSTSNNNSNNTAPATA

>E_salsugineum (XP_006411118.1)

MARDQLQVLNALDVAKTQWYHFTAIIIAGMGFFTDAYDLFCISLVTKLLGRIYYHVEGSQKPGTLPPNVAAAVNGVAFCGTLAGQLFFGWLGDKLGRKKVYGMTLMVMVLCSIASGLSFGHEPKAVMATLCFFRFWLGFGIGGDYPLSATIMSEYANKKTRGAFIAAVFAMQGFGIMAGGIFALIISSAFDAKFPAPAYADDALGSTVPQADLVWRIILMVGAIPAAMTYYSRSKMPETARYTALVAKDAKQAASDMSKVLQMEIEPEQQKMEETSKDKSKTFSLFSKEFMRRHGLHLLGTTSTWFLLDIAFYSQNLFQKDIFSAIGWIPPAQTMNAIQEVFKIARAQTLIALCSTVPGYWFTVAFIDVIGRFAIQLMGFFFMTVFMFALAIPYNHWTHKENRIGFVVMYSLTFFFANFGPNATTFVVPAEIFPARFRSTCHGISAASGKLGAMVGAFGFLYLAQSPDKTKTDAGYPPGIGVRNSLIVLGVVNFLGIVFTFLVPESKGKSLEEMSGENEENETSNNDNRTVPIV

**CyclinD6**

>S_lycopersicum (XP_004243793.1)

MEFDLENPLPFSHDETHFDTISPLFNIEAHHMPSKTYFQILINSNFLINIREATISKILQISQPFDSHFLPYLAINYLDRFLSFHSLSDAKPWILNLIGVSCVSLAFKMKKTEYSVTDIQQDAGSIFDIETIKRMELLILGSLKWRMRSITAFAFINFFISSFKFKDLPFQQALKARATEIIFTSQNEIKILQFKPSTISASALLCASHELFPLQFSCYKTAIINCSYVHKDDLLSCCNVIQEITKKEYESILEIVSSTSTPVNVLDVQMSWSSDNEPIEEDSSVNAISSRQDNLTKRRKIITTIIDDNT

>S_tuberosum (XP_006366940.1)

MDFDLENPLPFSHDEDTISPLFNIETHHMPSKTYLQILINSDFLINIRESTISIILQISQPFDSPSLSYLAINYLDRFLSFHSLPDAKPWILKLIAVSCVSLAFKMKKTEYSVTDIQQDGGTIFDIETIKRMELLILGGLKWRMRSITAFAFINFFVSLFKFKDLPLQQALKDRATEIIFTAQNEIKILQFKPSIISASALLSASHELFPLQFSCYKTAILNCSYVHEDDLLSCYDVMQEIAKKEYESILEMVSSTSTPVNVLDVQMSWSSDNEPIEEDSSVNAFSSNSRGRQDNLTKRRKIITTIIDDNT

>F_vesca (XP_004293473.1)

MEFNLENPLTISHDIHSDSVTSLFSIESDHMPSENYFQTLQAGDFDISIRREAIASISQLCCNSDKFLSYLAVNYLDRFLSCQEKLQPKPWIIKLLAISCVSLAAKMKKTAFSILDFQSDGGIIFDTRTIERMEFLILGALKWRMRSITPFSFILFFISLFKLEDPPLRQALKARATQIIFKAQNDMKLLVFKPSIIAASGLLFASHELFPMQSPCFKKALSNCSYVNKVTLLQCYNCMQDSVVDEYDSVLEMVSSSVTPANVLDQTFSSSAESGRTTVTSVTTLKLERDIKRRKISEYCKDHHDRVVQINFSSDPEPLRPS

>P_trichocarpa (XP_002326106.2)

MDFNLENPLTNSHELHFDTTPSLFLIESDHMPSKNYLKTLKEIDFDVSFRREAISSVLRVSCNFDPSLSYLAVNYLDRFLSSQGIPQPKPWVFKLLAVACVSLAAKMKEAEFYVTDIQGDGGFVFDPQTIQKMEVLILGALNWRMRSITPFSFISFFISLFKPKDPPLRQALKARACEIIFKAQNDINLLEFRPSLTAASALLYACHELFPMQFLCFRKAISICSYVNKENLLQCYNAMQETAMDGYKSQFDMVSSSDTPVNVLDRHFSSSESENTNGTVVMISSNGSNKTWPEKGIKRRKISALCNNQTVQLSAFSDATTMLM

>P_persica (XP_007215712.1)

MEFDLENPLTISHDIHSDTLTSMFSLESDHMPSEIYFQTVQARDFDISIRREAIASISQLCCNYDNLLQYLAVNYLDRFLSCQGMLQPKPWLIKLLAISCVSLAAKMKKADFSLADVQGDGRIIFDTQTIQRMEVLILGALKWRMRSITPFSFISFFNSLFKLEDPPLLQALKARAAQIIFKSQKDVELLGFKPSIIAASALLSASHELFPMQYPCFKKALSNCSYVNKENMLQCYSAMQDIVADGFDSVLEMVSSSVTAANVLDHNFSSSAESGTTTVTGMTPLRLEKDMKRRKISEYCKDHRVQISQIQNC

>T_cacao (XP_007043433.1)

MEFDLENPLTNFNDFCPGTTIPSLFLVESHHMPKENYVKTLKARDLDISVRRGAISLISQLSCKSSPFLSYLAVNYLDRFLSIQGIPQPKTWVLRLLAISCVSLAAKMKKTEFSIAHFQGDGGFIFDAQTIERMEYLILGALKWRMRSITPFSFISFFISFFKLKDPPLRQALKARALEIIFKSQTDVKLLVFKPSITAASALLSASHELFPLQFPCFRKAISSCSYVNKDNMLECYNSMQDIAKEGYESIFDMVSTSNTPVNVLDQHFSSSESETTDGIVTTATTIL

>G_max (NP_001242717.1)

MDFDLENPLGNFHDLPCDAVPSLFLIESDHIPPPNYCQSLKASDFDISVRRDVVSLISQLSCTFDPVLPYLAINYLDRFLANQGILQPKPWANKLLAVSCFSLAAKMLKTEYSATDVQVLMNHGDGGAIFETQTIQRMEGIVLGALQWRMRSITPFSFIPFFVNLFRLKDPALRQVLKDRASEIILKSQREIKVLEFKPSTVAASALLYASHELFPFQYPCFLRAISDCSYINKETVVQCYNVIQDIAREEYKSVLNINSTSDTPVNVLDEHFLSLESEKTNGTNVVVTQEQDFKRRKTTDYGNNRRVPFSHFHQC

>V_vinifera (XP_002276869.1)

MEFDLENPLTSSQDLHSHAVASLFQAENHHMPSIDYCGSLDSVDCDVSFRRQAISSILQMSSSFDPFLSYLAINYLDRFLSRSEMPSEKPWILRLLAVSCVSLAAKMKKTEFSLADFQGEGGFIFDSETIMRMEILVLGALKWRMRSVTPFSFISFFISLFKLKDPPLLEALKARVIEIILKSQKEIKLLQFKPSIIAASTLLYACHELFPLQFPCFMTAISNCPYVNKEKMLCCYSAVREMEIKEFDSLYGVVSSSSSPVNVLDRHCLSSESEKSHTMGAESDVKRRKISVFL

>C_sinensis (XP_006469812.1)

MDFSLENPFTNFHELFNDDDEDSTETILESLFLVESDHMPSKSYIKTLKGRDLDNSLRSRAVSSILQFSCKFDPFLSYLAVSYMDRYLSSQEMPQPKPWKLRLLAVSCFSLAAKMRQIEFSYTQFQADGGLIFDTQTIQRMECLILGALKWRMRSITPFTFLSFFISLFKLKDLTVQRALKTRASEVIFQAQIDIKLIEFKPSIIAASALLFASRELFPLQFHCFRKAISNCPYVNKENLLRCYNAMQDTSMDDEYESEIDLVSSSYTPVNVLDCRVSSSESDKTNVTTTDTKATSSSSDSSTTSTELSPERDTKRRKLSSYRNNHSIQLSQTQQC

>P_vulgaris (XP_007149914.1)

MDFNLENPLGNFHDLPWDALPSLFLIESDHIPPPNYCQTLKASDFDISVRRNVVSLISQLSCTFDPVLPYLAINYLDRFLANQGILQPKPWANKLLAISCFSLAAKMLKAEYSATDVQVLLNHGEGGVVFETQTVQRMEGIVLGALQWRMRSITPFSFIPFFVDLFRLKDPAFRQVLKDRASEIILKSQREIKVLEFKPSIVAASALLYASHELFPFQYPCFLRAISDCSYVNKETVVQCYNVIQDIAREEYESVLNINSTSDTPVNVLDEHFLSLESEKTNGVIQEQEFKRRKITDYGNKHTVPFSHFHQC

>C_arietum (XP_004487348.1)

MEFDLENPLENFHDLPSHGVSSFFLIESDHIPPQNYFQILKSNDFDISLRSDIISLISHLSCTFDPFVTYLAINYLDRFLANQGILHPKPWANKLLAVTCFSLAVKMLKTEYSATDVQALLNNGDGGFIFETQTIKRMEAVVLGALQWRMRSITPFSFIPYFTNLFNLDDQSLRKVLKDRASEIIFKSQNDIKVLEFKPSIVAASSLLYASHELFPFQYPCFLGTISDCSYVNKESVMQCYNVIQDIAKEEYESMFNVNSSSGTPVNVLDEHFLSLESELTNGTNVASTTMIQQEKHFKRWKI

>M_truncatula (XP_003597084.1)

MEFDLENPLEYFHDLPNSQDVSSLFLIESDHIPPLNYFQNLKSNEFDASVRTDFISLISQLSCNFDPFVTYLAINYLDRFLANQGILQPKPWANKLLAVTCFSLAVKMLKTEYSATDVQALMNHGDGGFIFETQTIKRMEALVLGALQWRMRSITPFSFIPYFTNLFMLDDITLKVLKDRASEIILKSQKDVKVMEFKPSIVAASSLLYSSHELFPFQYPCFLGIISNCSYVNKESVMECYNVIQDIAKEEYESMFNVHSSSGTPVNVLDENFLSLESEKTNGTNVAHTTMIQEKHFKRRKI

>R_communis (XP_002524706.1)

MEFDLENPLTSSNEHQSDTIPDLFASESDHMPSRDFLKCLKTCDFYSSFRQEAISLILQAQYTCNFEPFFAYLAINYMDRCVSRQEIPQGKPWLLRLLAISCLSLAAKMKDTHFPLSNLQREESFNFDMQTVSRMELLILGALNWRMRSITPFSFLHFFISLFELKDPPLTQALKDRATEIIFKAHHEIKLLEFRPSVIAASALLVASHELFPLQYPSFKCSIFSCECVNKENLLRCLNALQQMVEMVWYESMLDTVSSTRTPLSILDRHCTKSESETTSITTATALTDKKEIKRYKTIGYRSSE

>C_sativus (XP_004140053.1)

MDFDLENPLTHLHQLHSDDASLFLTESDHMLSPSYLHTLLTSPSDFAVRRDTIYFISQCCSNSNIDPHLSYLAVNYLDRFFSFQGMPQPKPWVLRLLAVSCVSLAAKMKQIEHNLSDFQGSEGFIFDPQTVHRMEVLILGALKWRMRSITPFSFIPFFSSLFKLRDPPLLQALKGRATEIIFIAQNGIELLEFKASVIAAAALLSAAHELFPIQYPCFRKAIINCSYVKKEEEEEEKLVRCLKAVEEIVINGHERRMDEMEERSETAGNVLDHHFSSSESENTSATKNRGDKDEGKMRKVGYCNNQRVQMREIQQC

>A_thaliana (NP_192236.1)

MEFHLEHPLSHSSLHNNFNDDTDYETLPHSLFLVEFQHMPSSHYFHSLKSSAFLLSNRNQAISSITQYSRKFDDPSLTYLAVNYLDRFLSSEDMPQSKPWILKLISLSCVSLSAKMRKPDMSVSDLPVEGEFFDAQMIERMENVILGALKWRMRSVTPFSFLAFFISLFELKEEDPLLLKHSLKSQTSDLTFSLQHDISFLEFKPSVIAGAALLFASFELCPLQFPCFSNRINQCTYVNKDELMECYKAIQERDIIVGENEGSTETAVNVLDQQFSSCESDKSITITASSSPKRRKTSTRRY

>E_salsugineum (XP_006396556.1)

MEFLLEHPLSHSSLDNNFNDESDDYSLPLSLFLVEFQHMPSSHYFNSLKSSAFLLSNRNHAVSSIVQYSRKFDDPSLTYLAVNYLDRFLSSEDMPQSKPWILKLISLSCVSLSAKMRKPDVFVCDLPVEGEIFDAQMIERMENVILGALKWRMRSVTPFSFFAFFISLFELKEDSLALKHSIKSQATDLTFNLQHDIKFLEFKPSVIAGAALLFVSSELCPLQFPCFSNRINQCTYVNKDELMECYKAIKERDIVEENEGSTETAVNVLDQQFSSCEESDKSITITASSPKRRKTSTRPC

>A_lyrata (XP_002872788.1)

MEFHLEHPLSHSSLHNNFNDDTDDDETLPHSLFLVEFQHMPSSHYFHSLKSSAFLLSNRNHAISSIIQYSRKFDDPSLTYLAVNYLDRFLSSEDMPQSKPWILRLISLSCVSLSAKMRKPEMSVSHLPVEGEFFDAQMIERMENVILGALKWRMRSVTPFSFLAFFISLFELKEDPLVLKHSLKSQAIDLTFNLQHDIRFLEFKPSVIAGAALLFASFELCPLKFPCFSNRIYQCTFVNKDELMKCYKAIQERDIVGENEASSETAVNVLDQQFSSCESDKSITITASSPKRRKTSTRRC

>C_rubella (XP_006288328.1)

MEFHLEHPLSHSSLHNNNSNDDETAPHSLFLVEFQHMPSSHYFHSLKSSAFLLSNRHHAISSILQYSRKFDDPSLTYLAVNYLDRFLSSEDMPQPKPWILKLISLSCVSLSAKMRKPEMSVSHHLPVEGEFFDAQMIERMENVILGALKWRMRSVTPFSFFSFFISLFELKEDSFALKHSLKSQAIDLTFHLQHDIRFLEFKPSVVAGAALLFASFELCPLQFPCFSNRICQCTYVNKDELMECYKGIQERDTVVEENEGSTETAVNVLDQQFSSCCESDKSITITASSPKRRKTSTRRC

>M_notabilis (EXC11594.1)

MEFDLTDPLTLSFQEEHQSDTDIADLFASEADHMHSRNFFSRLKNSDSYVSFRSEAISLVLQAQFSCDFDPFISYLAINYMDRYLVSKQEIPRDEEGFIFDAKTVHKMELLILDALSWRMRSITPFSFFAFFMSFFELNDHPLTQALKDRASEIIFSSQSDMKFSEFKPSVIAASALLSASHELFPLQFPSFNASISDCQYVKRENLIKCFNQMQEMVAMMQVYSSNCETFSSTKTPMSVLERNKLASSESENSSTVTSTTQCDTIISEKEDNKRRMDVMREGGFVNKPSFLESVNYPYWKARMKAFIKVIDENA

>S_italica (XP_004957987.1)

MATGEWELREEDASEYEFDLENPFTSPADEPIASLLEAEGHHAPSVSAAASAARRDAAGFISKVRFGGELAVNPRVAYLALNYVDRFLSKNQLPCEQQPWAPRLLAISCLSLAAKMQRVAAFSIADIQRDEEFMFDAVTVRRMERVLLDALEWRARSVTPLAFLGFFISACYPPPRHPLQVAAVKARAVDILLRAQPEVKMAEFSPSVAAAAALLAAAGEVTAANLPVFQAGVAACPFVNSDKLRECGEVLAAACGVGPGRAAASADTPVTVLGHHRSASSASESDWTVGSAANGGDAKKRCMGPPSQWG

>Z_mays (NP_001149068.1)

MATEEWELREEDAYAYEFEFDLENPFTSPADEPIASLLDAEGHHAPSISAAASATRRAAAAFISKVRFGGELAVHPRVAYLALNYVDRFLSKRQLACEQQPWAPRLLAISCLSLAAKMQRVATFSTADIQRDEDFMFDAVTIRRMERVVLGALEWRARSVTPLAFLGFFLSACYPPPQHPPQVAAVKARAVDLLLHAQPEVKMAEFSPSVVAAAALLAAAGEVAAANLHAFQASLAACPFVNSEKLRECGEVLAAAGGVGRGRAAPSADTPVTVLGHQRSASSASETDWINGGDAKKRCMGPPSQWG

>O_sativa (NP_001059961.1)

MDMATGAKEVVVVEAYEYEFDLENPFTSPADEPIASLLDAEGHHSPSVSAAASAARREAAGFISKVRYDGELDVHPRVAYLALNYVDRYLSKRQLACERNPWAPRLLAISCLTLAAKMQRAAAISAADIQRGEEFMFDEAKIQRMEQMVLNALEWRTRSVTPLAFLGFFLSACFPQPRHPALLDAIKARAVDLLLRVQPEVKMAEFSPSVAAAAALLAAAGEVAGAHLLGFEAGVAACPFVNSEKLRECGEVMAAACGVGPSWAAAATSAETPVTVLGHHRSASSESERTTTVGSAANSADAKRRCMGPPRQWGVGGPDE

>B_distachyon (XP_003562933.1)

MDMAGEEEYAYEYEFDLENPFTSPADEPIASLLDAEGPHSPSVSAAASSARRQAAGFISKVRYDGELAVHPRVAYLALNYVDRYLSKRQLPCEHKPWAPRLLAVSCLSIAAKMQRVDAISIADIQRDEEFMFDAVSIRRMERLVLGALEWRARSVTPLAFLGFFLSECFPPPRHPPLLAAVKARAVDLLLRAQPDLCRSRLXSCADFSPCIIVSLPQEKLRECGEAMAAACGVGPAAMSADTPSTVLGHGHYRSASSESDRTVGSVANGADAKKRCCMGPPSQWG

>T_urartu (EMS53686.1)

MDMPGEDEYVYGYEYEFDLENTFTSPADEPIASLLDAEAHHAPSVSAAASAVRRDAARFISKVRYDGELAVHPRVAYLALNYVDRFLSKGQLPFERKPWAPRLLAISCLSIAAKMQRVDAISMDYIQRDEEFMFDAVTIRRMERVVLGALEWRARSVTPLAFLGFFLSACFPPPRHPALLDAVKERAVDLLLRAQPEVKMAEFSPSVVAASALLAAAGEITVAHLPAFQAAVAACPFVNSEKLRECGEVMAAVCGVVGVGVGPAASAETPVTVLGHGHYRSASSESDRTVGSAANVADAKKRCMGPPSQSQCG

>S_bicolor (XP_002463007.1)

MATEEWALPDEEDAAYDYEFEFDLENPFTSPADEPIASLLDAEAHHAPSVSAAASAARRDAVAFISKVRFSGELAVHPRVAYLALNYVDRFLSKRQLACEQQPWPRLLALSCLSLAAKMQRVATFSIDHIQRDEDFMFDAATVRRMERWVLGALEWRARSVTPFAFLSFFLSVCYPPPQHPPQVAAIKARAVDLLLRAQPEVKMAEFSPSVVAASALLAAAREVAAANLPAFQAGVAACPFVNSEKLRECGEVLAAACGVGPGRAAASADTPVTVLGHHRSASSASESDWTIGSATNGGGGAKKRCMGPPSQWG

**Ribosomal protein L5**

>A_thaliana (NP_192040.1)

MASPSLLQSSASSFHGRFSPLAAPSSARMLSPPLRNVVKVSASGTVLVEKSEAEKTQRLKTAYLERIIPALKEEFKYVNIHQVPKVQKIVVNCGIGDAAQNDKGLEAAMKDIALITGQKPIKTRARASIATFKIREDQPLGIAVTLRGDVMYSFLDRLINLALPRTRDFQGVSPSSFDGNGNYSIGVKDQGVFPEIRFDAVGKTRGMDVCISTTAKSDQEGQKLLALMGMPFREGGGGSTGAIVRKKKLKSHHFDAKGKGKR

>A_lyrata (XP_002872896.1)

MASPSLLQSSASSFHGRFSPLAAPSSARMRSPPLRNLVKVSASGTVLVEKSEAEKTHRLKTAYLERIIPALKDEFKYVNIHQVPKVQKIVVNCGIGDAAQNDKGLEAAMKDIALITGQKPIKTRARASIATFKIREDQPLGIAVTLRGDVMYSFLDRLINLALPRTRDFQGVSPSSFDGNGNYSIGVKDQGVFPEIRFDAVGKTRGMDVCISTTAKTDQEGQKLLALMGMPFREGGGGNTGAIVRKKKLKSHHFDAKGKGKTKR

>C_rubella (XP_006288476.1)

MASPSLLQSSASSFHGRFPQLAAPSSARMLSAPLRNVVKVSASGTVLVEKSEAEKTQRLKTAYLERIIPALKEEFKYVNIHQVPKVQKIVVNCGIGDAAQNDKGLEAAMKDIALITGQKPIKTRARASIATFKIREDQPLGIAVTLRGDVMYSFLDRLINLALPRTRDFQGVSPSSFDGNGNYSIGVKDQSVFPEIRFDAIGKTRGMDVCISTTAKSDQEGQKLLALMGMPFREGGGGNTGAIVRKKKLKSHHFDAKGKGKGKR

>E_salsugineum (XP_006396279.1)

MASPSLLQSSASSFHGRFSSLAVPSSARVLPPPLRNVVKVSASGTVLVEKSEAEKTQRLKTAYLERIIPALKEEFKYINIHQVPKVQKIVVNCGIGDAAQNDKGLEAAMKDIALITGQKPIKTRAKASIATFKIREDQPLGIAVTLRGDVMYSFLDRLINLALPRTRDFQGVSPSSFDGNGNYSIGVKDQSVFPEIRFDAIGKTRGMDVCISTTAKTDQEGQKLLALMGMPFREGGGGSNTGAIVRKKKLKAHHFDAKGKGKGKK

>C_clementina (XP_006444963.1)

MACPSLLHSSASSFHGRFPALSSSSYVRPTYPNPINVNGVVSVKAAAGGIVLVEKSEAEKTGRLKTTYLEKIVPLLREEFSYTNIHQVPKIEKIVVNCGIGDAAQNAKGLEAAMNDLALITGQRPVKTRARNSIATFKIREGEPLGIAVTLRGNMMYSFLDRLINLGLPRTRDFQGVNPNSFDGHGNYSIGVKEQSVFPEIRYDALGKPKGMDVCITTTAKTDKEGQRLLALMGMPFREGGGPANLIRKKKLKAHHFDSKSKGKSRR

>T_cacao (XP_007051719.1)

MACPPLLQSTASSFHGQSPFLSPPFSVRLPYGNLRNGYGGVVSVKATADVVLVEKSDAEKSNRLKTTYLEKIIPLLKEEFNYTNIHQVPKIEKIVVNCGIGDAAQNAKGLEAAMNEMAMITGQRPVKTRARNSIATFKIREGQPLGIAVTLRGNVMYSFLDRLINLGLPRTRDFQGLNPNSFDGHGNYSIGIREQSVFPEIRFDALGRPRGMDVCITTTAKSDKEGQKLLALMGMPFREGGGPSVQPRKKKLKAHHFEKKSGRGRR

>V_vinifera (XP_002278820.1)

MASPALLQSNASSFHGQFPLASSPFSVRLPYGNPRNGCGFSVKALSEIVLVEKSEAVKTNRLKTTYLEKIVPLLKEEFSYQNMHEVPKIEKIVLNCGIGDASQNAKGLEAAMNELALIAGQRPVKTRARASIATFKLREGQPVGIAVTLRGNMMYSFLDRLINLGLPRTRDFQGVNPNSFDGHGNYSIGMKDQSVFPEIRFDILGRARGMDVCITTTAKTDKEGQQLLALMGMPFREGGGSTVVQRKKKLKAHHFDSKSKGRARR

>R_communis (XP_002511913.1)

MSSPSLLQSSAAASFRGQFPIISSHPSVKVPYGNPRNGNGVVSVRATGEIVLVEKSEAEKTYRLKTTYLQKIVPLLMEEFSYSNIHQVPKIEKVVVNCGIGEAAQNAKGLEAAMNDLALITGQRPIKTRARNSIATFKIREGQPLGIAVTLRGNVMYSFLDRLINLGLPRTRDFQGVSQNSFDGHGNYSIGIRDQSVFPEIRFDALGKAKGMDVCITTTAKNDQEGQKLLALMGMPFRESSGTTVLQRKKKLKKHHFDSKSRGRR

>P_persica (XP_007218824.1)

MACPSLLRSATSSFHGQFPVVVSPPPLRLSYGNPRNGGGLVMPVKASAVVLVEKSEAEKVSRLKTTYLEKIVPQLKEEFSYTNIHQVPKIEKVVVNCGIGDAQQNAKGLEAAMNDLALITGQRPIKTRAKVSLATFKIREGQPLGIAVTLRGNVMYSFLDRLINLGLPRTRDFQGLNPNSFDGNGNYAVGIKEQSVFPEIRFDLGKGRGMDVCIRTTAKTDKEAQTLLALMGMPFRETGPVSAVRKKKLKSHHFSSKGRGRR

>F_vesca (XP_004306741.1)

MACPSILRSSTSSFHGQFPIAASPPRLSFGNPRNVSVVKAAEVVLVEKSESEKVSRLKTAYLERIIPKLKEEFSYRNIHEVPKVEKIVVNCGIGDAQQNAKGLEAAMRDLALITGQRPVKTRAKASLAQFKIREGQPLGIAVTLRGNVMYAFLDRLINLGFPRTRDFQGLNPNSFDGNGNYGVGIKEQSVFPEIKFDLGKGRGMDVCIKTTAKTDKEAQTLLALMGMPFRETGPVTEVRKKKLRSHHFSRKGTGRK

>G_max (NP_001276281.1)

MATAPSLLHSSGPPFFSQFRAFQFSSSSLFPHGNRHGNAVVSVKASASGVVLVEKSEAEKANRLKTAYTEKIIPLLMEEFSYTNKHQVPKIEKIVVNCGIGDAAQNAKGLDAAISDLALITGQRPIKTRARASLATLKIREGQPLGIAVTLRGNMMYSFLDRVINLGLPRTRDFQGVNPNSFDGHGNYSIGIKDQGVFPEIRADVVGKPRGMDICIVTTANTDQEAQKLLALMGMPFREGSGPATTIRKKKLKSHHFDAKSKGRGRR

>S_lycopersicum (XP_004246669.1)

MAASPLLLHSTASSFYNAEFPAYSVRLPVGNTKVRSSNKLTVKASATVLVDKSEAEKVNRLKTNYLEKIVPLLKEEFSYTNILQVPKVEKIVVNCGIGDAAQNSKGLDAAMNDLALITGQRPVKTRAKNAIATFKIREGQPLGIAVTLRGNVMYSFLDRLINLGLPRTRDFQGVNPNSFDGHGNYSIGFREQSVFPELSYDALGKPRGMDVCITTTAETDKEAHRLLALMGMPFREGSGGQTTFTKKKKLRAHHFDSKAKQRSRR

>S_tuberosum (XP_006361806.1)

MAASSPLLLHSTASSFYNAEFPAYSVRLPVGNPKLRSSNKLTVKASAIVLVDKSEAEKVNRLKTNYLEKIVPLLKEEFSYTNILQVPKVEKIVVNCGIGDAAQNSKGLDAAMNDLALITGQRPVKTRSKNAIATFKIREGQPLGIAVTLRGNVMYSFLDRLINLGLPRTRDFQGVNPNSFDGHGNYSIGFREQSVFPELSYDALGKPRGMDVCITTTAETDKEAHRLLALMGMPFREGAGGQTIFTKKKKLRAHHFDSKAKQRSRR

>M_truncatula (AFK46665.1)

MATTPSLLHSSASSFLSQFRALPTQFSSSSLLCHGNNRVVSVKADASGAVLVEKSEAETVYRLKTAYNDKIVPLLMEEFSYTNIHQVPKVKKIVVNCGIGEAAQNAKGLDAAILDLALITGQRPVKTRARNSVATFKIREGQPLGIAVTLRGKIMYSFLDRVINLGLPRTRDFQGVNISSFDGNGNYNIGIKDQTVFPELKSGIGTPRGMDICISTTAKTDQEGQKLLALMGMPFREGVEVTQIVRKKKLKSHHFDPKSKGRGDRAKK

>P_trichocarpa (XP_002302562.1)

MATTSLLSSSAASFYGRFPTLPPHLNARVTYGSRNGVVSVRATGDVVLVDKSEAEKSNRLKTTFLEKIVPLLIEEFSYTNIHQVPKIQKVVVNCGIGDAAQNAKGLDAAINDLALITGQRPVKTRARNSVATFKIREGQPLGIAVTLRGNLMYSFLDRLVNLGLPRTRDFQGVTANSFDGHGNYSVGIRDQSVFPEIRFDAVGKARGMDVCIATTANTDQEAQRLLALMGMPFREGGGGGGATAQPRKKKLKAHHFDSKSKGRSRR

>L_japonicus (AFK41179.1)

MATTTPSLLLHSSGSSFLAQFRPSPPQFSPHGIRHGNAVVSVKATAAGAVLVEKSEAETVFRLKRTYTEKVIPKLVEEFSYTNIHQVPKVKKIVVNCGIGEAAQNAKGLDAAVNDLALITGQRPVKTRARTSVATFKIREGQPLGIAVTLRGKVMYSFLDRLVNLGFPRTRDFQGVNTSSFDGHGNFNIGIKDQGVFPEIRFDVVGKPRGMDVCIETTAETDKEAQRLLALLGMPFREGGGSSATALPKKKLKSHHFDPKAKGKVRK

>C_arietum (XP_004492787.1)

MATTPSSLLHSSGSSFLSQFRTFPPQFSSSTSFFPHRYGTAVVSVKAADSGAVLVEKSEAENVHRLKSTYNEKIVPLLMEEFSYINIHQVPKVKKIVVNCGIGDAAQNAKGLDAAINDLALITGQRPVKTRARNSVATFKIREGQPLGIAVTLRGNMMYSFLDRVVNLGLPRTRDFQGVRINSFDGNGNFNIGVKDQTVFPELRSAIGTPRGMDICISTTAKTDQEGQRLLALMGMPFREGVEATSAIRKKKLKSHHFDPKSKGRARK

>C_sativus (XP_004133849.1)

MASSSLLSSSTSSFHGHSPFFSVRFAAPVGCGNPSNVAQLGMRVKALASTGGAIVLVEKAEAEKVNRLKSNYLEKIVPLLMDEFSYDNIHQVPKIEKIVVNCGIGDAQQNAKGLEAAINELASITGQRPVKTRAKKSIATFKIREGQPLGIAATLRGNVMYSFLDRLINLGLPRTRDFQGLNSSSFDGHGNYSIGIREQSVFPEIKFDTLGKPRGMDVCITTTAETDQEAQRLLALMGMPFRESGGAAAVMRKKKLKSHHFDSKSKGRARR

>P_vulgaris (XP_007139794.1)

MATAPSLLHSSGSSFFSQFRAFPFSSSSLVFPHGNRHGNAVVSVRASDSAVVLVEKSDAEKANRLKTAYNEKVVPLLIEEFSYPNKHQVPKIEKIVVNCGIGDAAQNSKGLDAAINDLAMITGQRPVKTRARASLATFKIREGQPLGIAVTLRGNIMYSFLDRVINLGLPRTRDFQGVNSNSFDGHGNYSIGVKDQGVFPEIRADVVGNPRGMDICITTTANTDKEGQRLLALMGMPFREGSGSAAAAPKKKLKSHHFDSKRGGRGRK

>A_trichopoda (XP_006829072.1)

MVSAILSPSLPTPLFPTSSTTRNPSLQWSSRNPKRLTVVATSSTIVLVDKAEATKVNRLKHLYNEKVVPLLKEEFSYTNIHEVPKIEKIVVNCGIGDAAQNSKGLEAAIKDMSLIAGQRPVKTRARVSIASFKLREGYPVGIAVTLRGNMMFYFLDRLINLGLPRTRDFQGLNPNSFDGHGNFSIGLREQSVFPEIAYDAIGKPRGMDICITTTAKTDNEAQKLLALLGMPLRESGPTTPLVRKKKKKAHHFDSKSKLRGKK

>Z_mays (ACG27920.1)

MAAAAVTLPSSGTPFAVATTASSSSARRCLLLPSAAPRRALRVVASAATEAPPKPTQPPTSPSGIVLVDPSEAQKVHRLKAVYDQKVVPLITEEFGYTNVHQVPKLEKIVVNCGLGAEAGNSKGLDAAMKDLAMITGQWPVKTKARKSVASFKIREGNTIGIAVTLRGRIMYNFLDRLINLGLPRTMDFLGVNPNSFDGHGNYSLGLRDQGVFPEIPYEVGGKKNGMDVSIVTSAKTDNEAFRLLVLLGMPFSENIKTDVVIRKKRLKRHHFLSKGKGKGGRR

>H_vulgare (BAK01302.1)

MAATAVTLPTSAPSPFPVAASSARRCLQLLRSPPPRRAIRVAASAATEAPPKPPPATTSGIILVDPAEAQKVHRLKTVYDTKVVPIITEEFGYTNVHQVPKLEKIVVNCGLGVDAGNNKGLEAAMKDLASITGQYPVKTKAKNSVASFKIREGNTIGIAVTLRGRVMFNFLDRLINLGLPRTMDFLGVNPNSFDGHGNYTIGLRDQGVFPEIPYEVGGKKNGMDVTIVTTAKTDNEAQRLLALLGMPFAENIKSDQFKKKRLKRHHFMSKGRGRK

>O_sativa (AAC64970.1)

HEAVTAVTLPSSPAPFPVTTTASSSRNVRLLLRSPPPRRALRVAASAAADAPPKPAPPPTSPSGIVLVDPTEAQKVHRLKAVYDQKVVPLITEEFGYTNVHQVPKVEKIVVNCGLGAEAGNSKGLESAMKDLAMITGQWPVKTKAKKSVASFKIREGNTIGIAVTLRGRVMFNFLDRLINLGLPRTMDFLGVNPNSFDGHGNFTIGLRDQGVFPEIPYEVGGKKNGMDVCIVTTAKTDNEALRLLTLLGMPFAEHIKSSVVIRKKRLKRHHFMSKGRGRR

>S_italica (XP_004985903.1)

MAATAVTLPSSGVPFPVSNTAARRCLLLPSAPSRRALRVVASAATEAPPKPTPPPTSPSGIVLVDPSEAQRVHRLKAVYDQKVVPLITEEFGYTNVHQVPKLEKIVVNCGLGADAGNNKGLESAMKDLANITGQWPVKTKAKKSVASFKIREGNTIGIAVTLRGRIMYNFLDRLINLGLPRTMDFLGVNPNSFDGHGNYSLGLRDQGVFPEIPYEVGGKKNGMDVCIVTSAKTDNEALRLLTLLGMPFSENIKSDMVIRKKRLKRHHFLSKGKGKGGRK

>B_distachyon (XP_003558929.1)

MAATAVTLPSSTSPSPCPFSVATSSRRCLFPRSPLPRRAVRVVAASAAAAEAPPKPPPATPSGIILVDPSEAQKVHRLKTVYDEKVVPIITDEFGYTNVHQVPKLEKIVVNCGLGVDAGNSKGLDAAMKDLASITGQWPVKTKAKNSVASFKIREGNTIGIAVTLRGRVMFNFLDRLINLGLPRTMDFLGVNPNSFDGHGNYTIGLRDQGVFPEIPYEVGGKKNGMDVTIVTTAKTDNEALRLLTLLGMPYAEHMKSDQFKKKRLKRHHFMSKGRGRK

**Alpha-glucosidase (first AM-related protein from Table S4)**

**(sequences used for Figure 8a; 1 sequence per species)**

>S_lycopersicum (XP_004235201.1)

MTSLKISKKHHKHFNNPFPSTPNSPFIYGTLILNSHKLPSHQIYPIGKDFQLNWSSKNGGFLSISHKSEPTRPIWSTLPGEPFISAAIAETQVEESRGSFVVKDKHVHSLSSNQTIDDVKIINESDKDQLFSSYPLFPVLMITGKVFGVSKRKKKVGFSRRKDSEKENSTCARYWILFDQKECHQVGFQVRIGKTDLQLPKRVSPTSYRIFSLKFGRIRRRRGGWFGGLKKSVTVSSFAEEKIVMKNSEGVVNNRICLTYSSEKNEKIFGFGEQFSHMNFKGKRVPIFVQEQGIGRGDQPITFAANLVSYRAGGDWSTTYAPSPFYMTSKMRSMYLEGYDYSVFDLTKDDRIQIQLHGDSLEGRILHGNSPTELIECFTRSIGRPPLLPEWIISGAVVGMQGGTDTVRSIWNEMQRYDVPVSAFWLQDWVGQRETVIGSQLWWNWEADETRYSGWKQLIQDLNKQHIKVMTYCNPCLAPMDKKTNIRRHHFEEAKKLDILVKDKNGELYMVPNTAFDVGMLDLTHPRTANWFKQILREMVDDGVRGWMADFGEGLPVDACLYSGEDPIAAHNRYPELWAKINREFVDEWKNTHVGKEGEDPEDSLVFFMRAGYRDTPKWAMLFWEGDQMVSWQKNDGIKSAVVGLLSGGLSGYALNHSDIGGYCAVNLPLPFFKYQRSEELLLRWMELAAFTTVFRTHEGNKPSCNSQFYSNNRTLSHFARLAKVYKAWKFYRIQLVKEASQKGLPICRHLFLHYPEDEDVHSLTYEQFLVGTEILVVPVLDKGKETVKAYFPIGERLSWKHIWTGKLYSTHGSEAWVEAPIGYPAIFVKEGSSVGKTFLEKLREYNVL

>S_tuberosum (XP_006358190.1)

MTTLKITKKHHKHFNNPFPSTPNSPFIYGALILNSHKLPSHQIYPIGKDFQLNWSSKNGGFLSISHKSEPTRPLWSTLPGEPFISAAIAETEVEESRGSFVVKDKHVHSLSNNQTIDDIRIINESDKDQLFSSYPLFPVLMITGKVFGVSKRKKKVRFSRRKDSDKENSTCARYWILFDQKECHQVGFQVRIGKTDVELPKRVSPRSYRNFSLKFGRIRRRRGGWFGGLKKSVTVSSLAEEKIVMKSSEGVVNNRFYLTYSSERNEKIFGFGEQFSHMNFKGKRVPIFVQEQGIGRGDQPITFAANLVSYRAGGDWSTTYAPSPFYMTSKMRSMYLEGYDYSVFDLTKDDRIQIQLHGDSLEGRILHGNSPSELIECFTGSIGRPPLLPEWIISGAVVGMQGGTDTVRSIWNEMQRHDVPVSAFWLQDWVGQRETVIGSQLWWNWEADETRYSGWKQLIQDLNTQHIKVMTYCNPCLAPMDKKPNIRRHHFEEAKKLDILVKDKNGELYMVPNTAFDVGMLDLTHPRTANWFKQILQEMVDDGVRGWMADFGEGLPVDACLYSGEDPIAAHNRYPELWAKINREFVDEWKSTHVDKEGEYLEDSLVFFMRAGYRDTPKWAMLFWEGDQMVSWQKNDGIKSAVVGLLSGGLSGYALNHSDIGGYCAVNLPFFKYRRSEELLLRWMELAAFTTVFRTHEGNKPSCNSQFYSNNRTLSHFARLAKVYKAWKFYRIQLVKEASQKGLPICRHLFLHYPEDEHIHSLTYEQFLVGTEILVVPVLDKGRETVKAYFPIGENSSWKHIWTGKLFSTQGSEAWVEAPIGYPAIFVKDGSSVGKTFLEKLREYNVL

>T_cacao (XP_007028357.1)

MHNQNSPTLIMSTLKITKKHHKHLNNPFPSTPRYLPSIQGNLFINSQTLPPHQIFPVGKDFQLLWSTRNGGSISISHQSQPSKSLWSTIPGQAFMSAALAETEVEESRGSFVVKDRDVHLVCQHQTLDDIILINPFDDKDNDFLPDHLELDRLKIDSKIADPPVLVITGHIFSKRKKKRLQSSGIYKDIKFEKREPAASARYWVLFDQKNCNQIGFQVKIGQPNFQLLHQKASPLTASGWYRRLRRKLGRYRKRKLGWSWVFTRTKGLVTVSSSEEELGELNVAEPSAEFNRVCFTYASEGNERFFGFGEQFSRMDFKGKRVPIFVQEQGIGRGDQPITFAANLVSYRAGGDWSTTYAPSPFYMTSKMRSLYLEGYNYSIFDLTQHDRVQVQIHGNAIQGRILHGNSPLEIIEHFTEAIGRPPKLPEWMISGAVVGMQGGTETVRCVWDKLTTYKVPISVFWLQDWVGQRETLIGSQLWWNWEVDTTRYPGWQQLVKDLSTHSIKVMTYCNPCLALMDEKPNKRRNLFEEAKELDILVRDQHGEPYMVPNTAFDVGMLDLTHPLTANWFKQILLEMVNDGVRGWMADFGEGLPVDAVLYSGEDPISAHNRYPELWAQINREFVEEWKSNHVGNEREDPEEGLVFFMRAGFRNSPRWGMLFWEGDQMVSWQANDGIKSSVVGLLSSGLSGYAFNHSDIGGYCAINLPIIKYHRSEELLLRWMELNAFTIVFRTHEGNKPSCNSQFYSNDQTLSHFARFAKVYKAWKFYRVQLVKEAAQKGWPICRHLFLHYPDDEQVQRFSYQQFLVGSEILVVPVLDKGKKNVKAYFPVGETCTWQQIWTGKQYQKQGCEAWVEAPIGYPAVFVKVGSTVGETFLRNLRNLDIL

>P_trichocarpa (XP_002308887.1)

MGTLKITKKHHKHLNNPFPSTPRSLPFIQGRLLFNSQTVPPNKIFSVGKDFQLLWSIKNGGSLSIYHQSQPTKALWSTIPGQAFVTAALCETEVEESRGSFAIKDRNVYLVCDHQTIEDIRVISEPDHHFDQENDHDLSSGNMSFAQKNDWKDTQFPALVITGWLFSNRRKKRHQESGIYKDIQFETRGPPTCARYWVLFDQKNNNQIGFQVRVGPPNFEFQQRISPTPLGRHRRLRWKLGKIRRRKLGWYRFFTRSRGFVAVSSSSEEEMEMKSAELTEFNRVCITYSSEGNERFYGFGEQFSHMDFKGKRVPIFVQEQGIGRGDQPITFAANLVSYRAGGDWSTTYAPSPFYMTSKMRSLYLEGYDYSVFDMTRHDRVQIQIQSNSVRGRILNGNSPSEIIENFTETIGRPPELPKWIISGAVVGMQGGTEAVRRVWDELKDHKVPVSAFWLQDWVGQRETMIGSQLWWNWEVDTTRYHGWQQLINDLGAKNINVMTYCNPCLAPTDEKPNQRRNLFEEAKKLDILVKDKYGEPYMVPNTAFDVGMLDLTHPDTAAWFKQVLQEMVDDGVKGWMADFGEGLPVDATLYSGEDPISAHNRYPELWAQINREFVEEWKSGRAGKEREDPEEALVFFMRAGFRDSPKWGMLFWEGDQMVSWQANDGIKSSVVGLLSSGISGYAFNHSDIGGYCAVNLPFIKYHRSEELLMRWMELNAFTTVFRTHEGNKPSCNSQFYSNHKTLSHFARCAKLYKAWYFYRIQLVKEAARKGLPVCRHLFLHYPNDRNVHSLSYQQFLIGTEILVVPVLDKGKKNVKAYFPEGETCSWQHIWSGKLFKEQGSEAWVEAPVGYPPVFIKAGSTVGETFVENLRNFGIL

>V_vinifera (CBI30134.3)

MVYYQKKIIKYRSCLIHCLLSCGKFHIWASQHLNMAALKISKKHHKHLNNPFPSTPSSLPLLRGSLFFNPQTVPSDQTFIVGKDFQVLWSTDNGGSLSISHQSHPSRPIWSTVPGQAFVSAALAETEVEESRGSFAIKDGNVHLLCNDQTVEDIRLINENDCYLEANELDFLSGNQGLDQKPYLKDTQFPILLLTGWVFRKKKKSFQNTEIHERLQLEAERSTYARYWVLFDQKTSNQIGFQVKFGKPNFEFRSRAFATASRRFRGLKRKLRRTGRSRLGWCWSFSRPRGFVKVSSSEEEKEEKVAESIGFNRVCLTYSSEENERFYGFGEQFSHLNFKGKRIPIFVQEQGIGRGDQPITFAVNLVSYRAAGDSSTTYAPSPHYLTSKMRSLYLEGYDYSVFDLTRKDRVQIQIHGDSVQGRILHGNSPSELIERFTETIGRLPELPEWIISGAVVGMQGGTDSVRQVWEKLQAHNTPVSAFWLQDWVGHRETLIGSQLWWNWEVDTARYWGWQNLIKDLSAQHIKVMTYCNPCLAPTNEKPNRRRDLFEEAKKLDILVKDKNGDTYMVPNTAFDVGMLDLTHPDTASWFKQILQEMVDGGVRGWMADFGEGLPVDASLYSGEDPIAAHNRYPELWAQMNREFVEEWKSAHSGKAREDPEEALVFFMRAGFRNSPKWGMLFWEGDQMVSWQANDGIKSAVVGLLSSGISGYAFNHSDIGGYCAVNLPVIKYRRSEELLLRWMEVNAFTVVFRTHEGNKPSCNSQFYSNHKTLAHFARFAKVYKAWKFYRVQLVKEAAQKGLPVCRHLFLHYPNDEHVHKLSYQQFLVGTEILVVPVLDRGKKDVKAYFPVGESCSWQHIWTGKLFAKPGSEVWVEAPIGHPAIFVKEGSIIGETFLKNLREFNIL

>R_communis (XP_002522166.1)

MATIKITKRHKKHLNNPFPSSPRSLPFIQGSLLFNSQTVPSHQIFPVGRDFQLLCSTNNGGYISVSHQSQPTRALWSSIPGQAFVSTAVAETEVEESRGSFVIKDKNVLLVCDHQSIDGIRVINQLDDIQLEEASDLDSSPGYSSFDLKKDLNDTQFPLLLITGRLFSKTSKKRTPEYGIYQDIEFNTWGPPTSARYWFLLDQKNINQIGFQVRVGQPNFEFHPRTSPTRLGKYQRLRSKLRRIRKQRLGWFRFFTRPRGFFAVTSLEETEMKVPRLTDFNRICLSYSSEANESFYGFGEQFSHMDFKGKKVPIFVQEQGIGRGDQPITFAANLVSYRAGGDWSTTYAPSPFYMTSKMRSLYLEGYDYSVFDLTRHDRVQIQIHSSSAQGRIIYGNSPSDLIERLTETIGRPPELPKWIISGAVIGMQGGTEAVRRVWDELKAYKVPISAFWLQDWVGQRETFIGSQLWWNWEVDTTRYNGWKQLIQDLAAQHIKMMTYCNPCLAPTDEKPNRKRNLFEEAKKLGILVKDEHGEPYMVPNTAFDVGMLDLTHPDTASWFKQILQEMVDDGVRGWMADFGEGLPVDATLYSGEDPISAHNRYPELWAQINREFVEEWKTNLVGKEREDPEEALVFFMRAGFRDSPKWGMLFWEGDQMVSWQANDGIKSAVVGLLSGGFSGYALNHSDIGGYCAVNMPFVKYHRSEELLMRWMELNAFTTVFRTHEGNKPSCNSQFYSNDKTLSHFARCAKMYKAWYFYRIQLVKEASQKGLPVCRHLFIHYPNDRHVHNLSYQQFLVGTEILVVPVLDKGKQNVKVYFPEGETCSWKHVWSRKLFTAQDSETWLDAPIGYPAVFIRDGSFVGETFLENLRTLGIL

>P_persica (XP_007203811.1)

MTTLKITKKHHKHFNNPFPSTPASLPLLQGNLLFNSQTVPSHQHFSIGKDFQLSWSSNNGGSLSIYHQSQPKRAIWSTIPGQAFVSAALAETEVEESRGSFVVKDRKLHLVCHHQTILDIRVIDQFDHSLEAQDQDSPSGFLDLDQKTDFKGTQFPMVLVTGWVFNMRRKKKHSHKYGTLENAQFEGKGPSTCARYWVLFEQKNRNQIGFQVKLGQPNFEFRTKASPAASGRYKGFRRRLGQFQKRRLRWFWSSARPRGFVFVSSSEEELEELKAEEFKEFNRVCLTYSSEENERFYGFGEQFSHMDFKGKRVPILVQEQGIGRGDQPITFAANLISYRAGGDWSTTYAPSPFYMTSKMRSLYLEGYDYSIFDLTKQDRVQIQIHGNSVEGRILHGTSPSELIECFTETIGRPPKLPDWIISGAVVGMQGGTESVRHIWNELKTYNAPISAFWLQDWVGQRETLVGSQLWWNWEVDSIRYTGWQQLIKDLSVQHIKVMTYCNPCLAPCHEKPNRRRNLFEEAKKLDILVKDKLGEPYMVPNTAFDVGMLDLTHPDTASWFKQNLQEMVDDGVRGWMADFGEGLPVDATLYSGEDPISAHNKYPELWAQINREFVDEWKANRVGKEVEDPEEALVFFMRAGFRDSPKWGMLFWEGDQMVSWQTHDGIKSAVVGLLSSGISGYAFNHSDIGGYCAVNLPFINYRRSEELLLRWMELNAFTTVFRTHEGNKPSCNSQFYSNDRTLSHFARFAKIYKAWRFYRVQLVQEAAQKGLPVCRHLFLHYPDDEHVHSLSYHQFLVGTEILVVPVLDKGKNNVKAYFPTGESCTWQHIWTGKHFGRQGVEATVEAPIGCPAVFVKTGSIVGETFLKNLIDLKVL

>C_sinensis (XP_006477491.1)

METIKITKKHHKHLNNPFPSTPKSLPLIQGSLAFNCEKLSSHQIFTIGNDFEILWSSNNGGYLSISHQSKPARPLWSSIPGKAFVSAALAETLVEESRGSFVIKDRHIHLLCQDQTIDDIRVINDQFHFDGPFTLHQNVQFPLVLITGWIFSKKIKKTNQSYVIYNKKDIQFETKAGYWLLFDQKTSHQIGFELKLGQPNFAIRQRRMGRIRIRKRKLGWCWSLTRPKGFVRISSTETENQPAAELKIPIPQHREFNRVFLTYSSEGNERFYGFGEQFSHMDFKGKRVPIFVQEQGIGRGDQPITFAANLVSYRAGGDWSTTYAPSPFYMTSKMRSVYLQGYDYSVFDLTRVDRVQIQIHGNSVQGRILHGNSPCELIEHFTETIGRPPELPDWIVSGAVAGMQGGTDAVRRVWDALRSYQVPVSAFWLQDWVGQRETLIGSQLWWNWEVDTTRYKGWKQLLKDLNAHHVKVMTYCNPCLAPSHEKPNRRRNLFEEAKKLDILVKDKNGEQYLVPNTAFDVGMLDLTHPDTASWFKQVLQEMVEDGVRGWMADFGEGLPVDAILYSGEDPISAHNRYPELWAQINREFVEEWKDKCTGTKREDTEEDLVFFMRAGFRDSPKWGMLFWEGDQMVSWQANDGIKSAVVGLLSSGLSGYAFNHSDIGGYCAVNLPLIKYRRSEELLLRWMELNAFTTVFRTHEGNKPSFNSQFYSNQQTLSHFARFAKVYRAWKFYRIKLVKEASQKGLPVCRHLFLHYPDDDKVQRLSYQQFLVGTEILVVPILDKGKKKVRVYFPVGETSTWQHIWTEKIFTGQGSEAWVEAPIGYPAVFVKADSIVGETFRKNLRNSDIL

>M_guttatus (EYU18620.1)

MATFKTTKKHHKHINNPFPSKPKTLPFIKGALIFDPHTLPPHRIYDIGLDFHLNWSSDNGGSLSIHHNSHPTRSIWSTVPGRAFVSAGAADTEVEESRGSFLIKDGEIHLLCNHQTIEEIRIIQQESSLLIKGRIFSVREKKKGNQESFEVVEKGQVAFYAKYWMVFDQRNCNQVGFRLRFGKPSFGQKQKLKQKLSPKSYGYKGFGRKVGRIRRVRVVGWCGCFSRKRVVVSASQEDENAAMKNAILPDFNRVCVTYSSEKNERFFGFGEQFSHMDFKGRKVPIFVQEQGIGRGDQPITFAANLVSFRAGGDDSTTYAPSPFYMTSKMKSLYLEGYNYSVFDLTKDDCVQIQIHGDTIEGRILNGNSPVELIEHFTETIGRPEKLPDWIISGAVVGMQGGTDKVRSILDELRAKDTPISAFWLQDWVGQRKTVIGSQLWWNWEVDSHRYSGWKQLIEDLSAQHIKVMTYCNPCLTPMGEKRNVRRNLFEEAVKSDILVKDSEGGIYMVPNTAFDVGMLDFTHPKTAGWFKQILQEMVDDGVRGWMADFGEGLPVDACLYSGEDPMTAHNRYPELWAKINREFVEEWKASRIGKAKEDPQEDLVFFMRAGFRESPKWASLFWEGDQMVSWQANDGIKSAVLGLLTSGISGYAFNHSDIGGYCSVSLPFFKYQRSEELLLRWMELNAFTTVFRTHEGNKPSCNSQFYSNQRTLSHFSRCAKIYKAWKFYRIELVKEASEKGLPVCRHLFLHYPEDEYVQTLTYEQFLVGTEILVVPVLDKGKGVVKVYFPQSESCSWKHVWTGKIYPKQGTESWVEAQIGYPAIFVKEGSKIGETFLRNLKAYDIL

>C_sativus (XP_004144332.1)

MTNLKVTKKHHIHLNNPFPSPPPSFPLLQGELSANYQALSSYKFFSIGKDFQLLWRSDNGGSLSIYHLSDPTRSIWSTISGQAFVSAAMVETEVEESRGSFAVKDGAVHLICNHQTIDDIKEINGCDHEFEVKEHHFPSGYLGLDLKNYEKEDAQFPMLLISGRIFNTEKKRMMKKKNKLQETSFNGDVKCNSKVLSASARYWVFFEQKSSSQIGFQVMLGQPSYEHRQIAHSRGGFNRLKFRLHRLRKRKFEWHWSLTKLKGFVRVPSSEKEVEVLRAAEEFEAFNRVCLTYSSEEKERFFGFGEQFSHMDFKGKRVPIFVQEQGIGRGDQPITFAANLISYRAGGDWSTTYAPSPFYMTSKMRSLYLEGYEYSIFDLTKNDRVQIQIHGNSVQGRILHGNSPSELIERFTETIGRPPELPGWIISGAVVGMQGGTNVVRKIWDELKAHEVPISAFWLQDWVGQRETVIGSQLWWNWEVDATRYSGWKQLIKDLGARHIKVMTYCNPCLAPTDEKQNRRRNLYEEAKALGILIKKKNGEPYMVPNTAFDVGMLDLTHPNTSSWFKKILQEMVNDGVRGWMADFGEGLPVDATLYSGEDPITAHNRYPEIWAQINREFVDEWKSKLVGKEKEDPEEALVFFMRAGFRNSPKWGMLFWEGDQMVSWQANDGIKSAVTGLLSSGLSGYAFNHSDIGGYCAVNLPFIKYRRSEELLLRWMELNAFTTVFRTHEGNKPSCNSQFYSSDRTLSQFARFAKVYSAWKFYRIQLVKEAAERGLPVCRHLFVHYPEDEYVLTLGHQQFLVGSEILVVPVLDKGKNNVNAYFPLGDNSSWQHIWTGEVYAKLGCEIKVDAPVGYPAVFIKVGSIVGETFIRNLKMFNIL

>M_truncatula (XP_003624991.1)

MAILKITKKHHKRFNNPFPSAPTTIPNVQGSLFINSKALSSQDQTFSIGNDFQLSWSTLNGGQFSISHLSQKTRPIWSTISGKAFVSAAVVDAEIEESRGSFLVKDKDVHLTCNHQTIDDIRIINEFGDHLEYEVEDLDQKCSAEETKFPPTLLITGRLFNMSKKKKRFQKYGIQGNIQFEPKGPFVYARYWVLFNQKNKHEIGFQVKIEKLNFSLSNKVVSPEASEIYKGFKKRLSSRKKKIGWCWYLSRPRGFVLVSSVEDESGVMEIPKPKEFNRVWLTYASDENERFYGFGEQFSHMNFKGKRVPILVQEQGIGRGDQPITLAANLVSYRAGGDWSTTYAPSPFYMTSKMRSLYLEGYDYTIFDLTKLDRVQIQIYGNSIEGRILHGNNPCDLIKHFTKTIGRLPELPEWIISGAIVGMQGGTDAVRRVWDELRTYDVPVSGFWLQDWVGQRETMIGSQLWWNWEVDEQRYWGWKELIKDLSTQNIKVMTYCNPCLAPVDEKNNKKRNLFEEAKQLDILVKDNNGNAYMVPNTAFDVGMLDLTHPKTATWFKQILLEMVDDGVRGWMADFGEGLPVDAVLYSGEDPISAHNRYPELWAKINREIVEEWKSKSLDNLKEEQEDGLVFFMRAGFRDSPKWGMLFWEGDQMVSWQANDGIKSSVVGLLSSGISGYAFNHSDIGGYCTVNLPIVKYRRSQELLLRWMELNSFTTVFRTHEGNKPSCNSQFYSNQQTLSHFARTAKIYTAWKFYRIQLVKEAAQKGLPVCRHLFLHYPNDEHVHNLSYQQFLVGSEFLVVPVLDKGMKKVKAYFPLGESSSWLHIWTGNVFSKQGSESWIEAPIGYPAVFIKFGSIIGETFLNNLKNLGILQ

>G_max (XP_003521128.1)

MAILKITKKHHKRFNNPFPSSVSTTIPYVQGSLFFNSKRVPSDQTFSIGTDFHLSWTSNNGGHLSISHLSHQTRPIWSTIPGQAFVSAALVDTEVEESRGSFLVKDKDVHLVCNHQTIEDIRVIEDISQFDHHLECEVADSPCVSQGLEKKSDAQEIHLPTLMITGRLFNMSKKSKRFQKHGIQATMQFEAKGPSVYARYWVLFNQKGNHEVGFQVKIEKPNFVSRNQVSKKASGVYQGFKRRLSNRKKRLDWCWYLSRPRGFVLVSSVEEEIGNLDIPKPEEFNRVWLTYASDENERFYGFGEQFSHMNFKGKRVPIFVQEQGIGRGDQPITLAANLISYRAGGDWSTTYAPSPFYITSKMRSVCLEGYDYTVFDLTRLDRVQIQIHGNSVEGRILHGNSPCELIERSTESIGRLPELPEWIISGAIVGMQGGTDAVRHIWDELRTYDVPVSAFWLQDWVGQRETLIGSQLWWNWEVDAQRYWGWKELIKDLSSQNIKVMTYCNPCLALVDKKQNKRRNLFEEAKKLDILVKDSNGNPYMVPNTAFDVGMLDLTHPKTATWFKQILREMVDDGVRGWMADFGEGLPVDAVLYSGEDPISAHNRYPELWAKINRELVEEWKSSSLDKVKEDEDEGLVFFMRAGFRDSPKWGMLFWEGDQMVSWQTNDGIKSSVVGLLSSGISGYAFNHSDIGGYCTVNLPIVKYRRSEELLLRWMELNSFTTVFRTHEGNKPSCNSQFYSNHQTMSHFARSAKVYKAWKFYRIQLVKEAAQKGLPICRHLFLHYPDDECVHRLSYQQFLVGSEFLVVPVLDKGKKKVKAYFPLGESSSWIHIWTGKVFSKQGREEWVEAPIGYPAVFVKVGSQVGETFLNNLRSLGIL

>C_arietum (XP_004493399.1)

MAILKITKKHNKLFNNPFPSAPTTIPYVRGSLFINSKALSSSDHTFSIGNDFQLYWSTINGGHLSISHLSMVNRPIWSTIPGKAFVSAAVADTEIEESRGSFLVKDKDVHLMCNHQTIDDIRMINQYEVVESPCGNSGLDLDQKSYAEDTTKFPTLLITGRLLNMSKKNKRFQKCGIEANIQFEAKGPFVYAKYWVLFNQKNKHEVGFQVKIEKPNFVSSNNKVSSEASGVYKGFKRRLSNRKKRIGWCWYLSRPRGFVLVSSVEDEIGDKVEMTKPKEFNRVWLTYASDENERFYGFGEQFSYMNFKGKRVPILVQEQGIGRGDQPITLAANLVSYRAGGDWSSTYAPSPFYMTSKMRSLYLEGYDYTIFDLTRLDRVQIQIYGNSIEGRILHGNTPCELIERFTETIGRLPELPEWIISGAIVGMQGGTDAVHRIWDELRAYDVPVSAFWLQDWVGQRETLIGSQLWWNWEVDEQRYWGWKELIKDLSTQNIKVMTYCNPCLAPVDEKHNKRRNLFVEAKQLDILVKDNNGNPYMVPNTAFDVGMLDLTHPKTATWFKQILLEMVDDGVRGWMADFGEGLPVDAVLYSGEDPISAHNRYPELWAKINREVVEEWKSNNSMDKLKNEDQEKDGLVFFMRAGFRDSPKWGMLFWEGDQMVSWQTNDGIKSSVVGLLSSGISGYAFNHSDIGGYCTVNLPIVKYRRSQELLLRWMELNSFTTVFRTHEGNKPSCNSQFYSNQQTLSHFARSAKVYTAWKFYRIQLVKEAAQKGLPVCRHLFLQYPNDEHVHNLSYQQFLVGSEFLVVPVLDKGKKKVKAYFPLGESSSWLHIWSGKIFSKQGSESWIEAPIGYPAVFIKVGSIIGETFLNNLRILGILQ

>F_vesca (XP_004303593.1)

MSSLKISKKHHKHLNNPFPGAPISLPLIQGKLLFNSSLIPQRFPIGNDFEVSWNSSEGGSLSISHRSQPNRSIWSTIPGQAFVSAAVAQTRVEESRGSFVIHDTSVDLVCHHQTIQDITQFDHASSSSSYSQGTQFPLVLVTGWVFNTGPSTCTCAKYWVLFEQKCSHQIGFQVKLGTPNFEFPFRTRLRWVWSFTRPREAQQFKEFNRVCLTYSSEETERFYGFGEQFSYMDFKGKRVPILVQEQGIGRGDQPITFAANLISYRAGGDWSTTYAPSPFYMTSKMKSLYLEGYNYCAFDLTQHDRVQIQIHKNSVEGRILHGNSPTELIECFTETIGRPPKLPDWIISGAVVGMQGGTESVRRIWNELKSYNAPVSAFWLQDWVGQRETLVGSQLWWNWEVDSTRYTGWKQLIKELSAQHIKVMTYCNPCLVPCHEKPNRRRNLFEEAKKLNILVKDKQGEPYMVPNTAFDVGMLDLTHPDTGNWFKQILQEMVDDGVRGWMADFGEGLPVDATLYSGEDPISAHNKYPELWAQLNREFVEEWKANRVGKEKDPQETLVFFMRAGFRDSPRWGMLFWEGDQMVSWQIHDGIKSAVVGLLSSGMSGYAFNHSDIGGYCAVNLPFIKYQRSEELLLRWMELNAFTTVFRTHEGNKPSCNSQFYSNERTLAHFARFAKVYKAWKFYRIQLVKEATHRGLPVCRHLFLHYPNDEHVHNLSYQQFLIGTEILVVPVLDKGMNNVKAYFPTGNSSWQHVWTGKQFTEEGFETIVEAQIGYPAVFFKTGSIVGETFLKNLRDLKIL

>P_vulgaris (XP_007162089.1)

MVCNHQTIEDIRIIEDIGVINSQFDHHLECEVPDSPSVYRRLEKKTDAQEIQLPTLMITGRLFNMTKKSKRFQRHGIKATMQFEAKGPSVYARYWILFNQKTKHEVGFQVKIEKPNFTSRNQVSETVSRGYQGFKRSRSNRKKRLGWCWYLSRPRGFLLVSSVEEEIGDLDIPKPEEFNRVWLTYASDEKERFYGFGEQFSHMNFKGKRVPIFVQEQGIGRGDQPITLAANLVSYRAGGDWSTTYAPSPFYITSRMRSVCLEGYDYTVFDLTRLDRVQIQIHGNSAEGRILHGNSPCELIERFTESIGRLPELPEWIISGAIVGMQGGTDAVRHIWDELRTYDVPISAFWLQDWVGQRKTLIGSQLWWNWEVDSQRYWGWRNWEVDSQRYWGWKELIEDLSSQNIKVMTYCNPCLAPVDEKQNKRRNLFEEAKQLDILVKDGNGNPYMVPNTAFDVGMLDLTHPKSATWFKQILGDMVDDGVRGWMADFGEGLPVDAVLYSGEDPISAHNRYPELWAKINREIVEEWKSKPLDKVKKDQEEGLVFFMRAGFRDSPKWGMLFWEGDQMVSWQRNDGIKSSVVGLLSSGISGYAFNHSDIGGYCTVNLPIVKYNRSEELLLRWMELNSFTIVFRTHEGNKPSCNSQFYSNQQTMSHFARFAKVYKAWKFYRIQLVKEAAQKGLPVCRHLFLHYPNDERVQNLSYQQFLVGSEFLVVPVLDKGKKKVKAYFPLGESSSWVHIWTGKVFSKEGSEEWVEAPIGYPAVFLKVGSLIGETFLNNLRSLGIL

>A_trichopoda (XP_006832833.1)

MAIFKVEKKHHRRLNNPFPQDPNSLKFTKAKLFCNPHLPQNQSYHIGSNFLLCYSSENGVSLSISHQSDPPRSLWSTIPGQGFISSASSDTNVTESRGSFAIHDNNTKYCNHQTLEDIRLIKSHEAIREVLVDETQFGSQNVGSKPWFSGLLQETQFPILAITGCVYSREEEAEKQLQEFTKGSKNRGPFYIANDRDSAFTIFCKNRVLGLSIGARYWLLFAQKTGHQLEFCVEIKKAYSPTHPETSKSLQKVQWKFGQHLLRLSRDRGYITIASKKKKRDKVGDRELNRVIITYSSEGDERFYGFGEQFSHMEFKGKRVPILVQEQGLGRGDQPITMAANLVSYRSGGNWSTTYAPSPFYMTSKMRSLYLEGYNYSVFDLRKRDRVQLQVYGPSARGRILHGNSPAELIEQYTETIGRLPELPDWIISGAIVGMQGGTGAVRRVWDLLQQYDTPISAFWLQDWVGQRKTIIGSQLWWNWEVDTNHYAGWSELVKDLRSHDIRTMGYCNPCLAPVDEKPNKKKHLFEEAKKLDLFVKDKFGSPYMVPNTAFDVGMLDFTNPKSRRWFKQILQEMVDGGISGWMADFGEGLPLDACLYSGEDPISAHNRYPELWAEINREFVDEWKSMNQAKQREDSEESLVFFVRAGYRGSPKWASLFWEGDQMVSWQRNDGIKSAVVGLLSSGLSGYSLNHSDIGGYCAVNFPLIKYQRSEELLLRWMELNAFTTIFRTHEGNNPSFNTQFYSNRRTFTHFARFAKVYKAWKFYRIQLVKEAAQNGLPVTRHLFIHYPDDQNVHRLTYQQFLVGSEILVVPVLDKGKKKVKAYFPVSQGSLWQNIWTGKLYGNRFCHSNHTHKGMEAWVEAPIGYPAVFVKTHSPIGETFLKSLKDLQIL

>M_notabilis (EXB28636.1)

MSTFKITKKHHKHLNNPFPSSPTSLPFIHGTLFFTSQSLPSHHLYPIGQDFQLSWRSNNGGCFSIHHKSHPTKPPIWSTLPGQAFVSAALTETEVEESRGSFAVKDNDVHLVCNHQTVQNIQVINRFEDFLELQEHYFPSGSFGFDLERDFKGINFPVLLITGWVLSMDVKNKKFQKSGTSKFDSKGCSSCAKYWVLFDQKSGDQVGFQVKLGKPNFEFGSRAYSSSSNILGKYRGFRKRLGRFRRRRLGFYWSLNKPRKVVMASSSEEEMEEIRGKESQEFNRVCFTYSSEGSERFYGFGEQFSHMDFKGKRVPIFVQEQGIGRGDQPITFAANLVSYRAGGDWSTTYAPSPFYMTSKMRSLYLEGYDYSVFDLTKHDKVQIQIYGNSVQGRILHGNSPSELIESFTGAIGRPPQLPEWIISGAVVGMQGGTETVRRVWNELGTYNVPVSAFWLQIFAFSYSYSNLYTGLGGAKGDNHWVTTMVELGSGYCKAHEKPNRRRNLFEEAKKSDILIKDKNGEPYMVPNTAFDVGMLDLTHPDTGSWFKQILEEMVDNGVRGWMADFGEGLPVDATLYSGEDPISAHNRYPELWAQINREFVEEWKSKRVGKEKEDPEEALVFFMRAGFRDSPKWGMLFWEGDQMVSWQTNDGIKSAVVGLLSSGLSGYAFNHSDIGGYCAVNFPFIKYHRSEELLLRWMELNAFTTVFRTHEGNKPSCNSQFYSNHRTLLHFARFANVYKAWKFYRIQLVKEASSKGLPVCRHLFLHYPDDEHVHSLSYHQFLIGTEILVVPVLDKGKKSVKAYFPIGQTCAWQHIWTGEVFTRQGFEAKIEAPIGYPAIFVKSEISYEVGWPCIIDTAFSRLEPGKCLPLELQQIFIPELELLSDGAIEGIFVNGFDSLA

>H_vulgare (BAJ97257.1)

MASSATAKPKTTKKHSARLNNPFPRAVPASAFRSGDAAPPLSFGPLSKLAHAHDYPVGSRFRLSWNPSLGGAVSLARVSSSSSGGEPPSRVMWETIPGVAFVSAASATTEADECRGSFALRDGRARLVPDRQSVDRIRALYRRDVETGADSLRGAVFEASDQTRFPVLLITGVVSAKKADPASSCCCGLRARARAGKPVLSARYWVFLEEKSDTQVSFSVKIADYQWSCGHADPSSPTPAATTAPRPHRITLLSLRLRLAGRIHRNMSKKKKLSAGFPPQPQEEVSALLPSPERASADEEARPEEFNRVFLTYASDRDERFYGFGEQFSYMEFKGRRVPVLVQEQGIGRGDQPITFAANLISYRSGGNWSTTYAPSPFYMTSKMRSLYLEGYDYSIFDLTKPDRVQIQVYGNSVQGRILEGESPTELITSYTGSTGRPPVLPRWITSGAVVGMQGGTDAVRRVWSQLQDHDVPVSAFWLQDWVGQRKTAIGSQLWWNWEVDDDHYAGWKDLIRDLRSDGVRTMTYCNPCLVPVCEKGNARRHLYEEAKELGILVRDEAGEPYMMPNTAFDVAMLDLTNPEACSWFKGILRGMADEGVSGWMADFGEGLPLDARLHSGEDPVAAHNRYPELWARVNREFADECKPEEGLVFFVRAGFRESSRWAMLFWEGDQMVSWQANDGIKSSVVGLLSGGLSGIPLNHSDAGGYCTVDLPLLRYRRSEELLMRWMEVNAFTVVFRTHEGNKPGSNCQFYSNSRTLAHFARCAKMYKAWEFYRIHLVKEAAEKGLPVARHLFLHYPEDRRVQELTYQQFLVGTEMLVVPVLDKGRTAVTAYFPTSDGGSWRHVWTGQEFGGGRRSGHGSVGEATVHGFEAEVSAGVGCPAVFVRVGSPVGERFTRNLRDLGVI

>B_distachyon (XP_003566851.1)

MASPAHPKTTKKHNARLNNPFPRAVPAAAFRQYGDAAPPLSFAPSSAKLAQAHDHPVGSRFRLRWDPSHGGAVSLAGIHVSSSVMWETIPGVAFVSAASAVTEADECRGSFALRDGRARLVPDRQTVDRIRALYRCDADLLRAAAFQASEETRFPVLLITGVVSARKAGPASSCCCGLRARIRAGQPIFSARYWFLLEEKNNTQVAFGVKIADYQWSCGHAGSSSPPPAGGSTARPQRTSLRLRLHLAGRVQRNSGRKKKLSSGFPVREEELSALLPRLKKEDEEEARAPEEFNRVFLTYASERDERFYGFGEQFSRMEFKGKRVPVLVQEQGIGRGDQPITFAANLLSYRSGGNWSTTYAPSPFYMTSKMRSLYLEGYDYSIFDLTKPDRVQIQVYGNSVRGRILQGHSPTELITSYTGSTGRPPVLPRWITSGAVVGMQGGTDAVRRVWSQLRDHDVPVSAFWLQDWVGQRKTAIGSQLWWNWEVDDDHYAGWNDLVRDLRRGGVRTMTYCNPCLVPMGGKANARRHLFEEAKELGILVRDESGGPYMMPNTAFDVAMLDFTNPSACAWFKGILRGMAESGVSGWMADFGEGLPLDARLHSGEDPVAAHNRYPELWARVNREFADEWKKNSSGDHGTASEEEEEEEEGLVFFVRAGFRESSRWAMLFWEGDQMVSWQANDGIKSSVVGLLSGGLSGIPLNHSDAGGYCTVDLPPFLRYRRGEELLMRWMELNAFTVVFRTHEGNRPGSNAQFYSNARTLAHFARCAKVYKAWEFYRARLVKEAAETGLPVARHMFLYYPEDRRVQGMTCQQFLVGTELLVVPVLDKGRRTVAAYFPASDGASWRHVWTGQEFGNNGHGGVGAVHGFEAEVGAEVGYPAVFVRVGSPVGERFVSNLRDLKVV

>S_italica (XP_004967735.1)

MASPALSKTAKKHHTRRLNNPFPRAVPAAAISGGDAAPRLSFAPTSKLVHAHDFPVGTRFRLRWDPSLGGQVSLSRVPSSDDARRAMWGSVPGVAFLSAASAATVADECRGSFALRDVRARLVPHRQHVDKIRAFYRCDADAGAELLRGAAFQASGATRFPVLVITGLVSAKAASSSAPCGCCGLRAGRRARSAVGKPPALSARYWILLEERTDTQVAFSVKIGDYQWTCAAHADPAKSPLATATAAPRIHRPSLRLRLPARVQRPTGKKTRLAPSREEEASTALLTAPERAEERLPEEFNRVFLTYASSRDERFYGFGEQFSRMEFKGKRVPVLVQEQGIGRGDQPITFAANLVSYRSGGNWSTTYAPSPFYMTSKMRSLYLEGYDYSIFDLTKPDRVQIQVYGNSVQGRILDGDSPTQLLTSYTESTGRPPVLPRWITSGAVVGMQGGTDTVRRVWKELQDYDVPVSAFWLQDWVGQRKTSIGSQLWWNWEVDDAHYNGWKDLVGDLRRSGIRMMTYCNPCLVPMDQKPNTKRHLFEEAKKLGILVRDESGEPYMMPNTAFDVAMLDFTNPEAHAWFKEILRGMADDGVSGWMADFGEGLPLDVRLHSGEDPVAAHNRYPELWARVNREFADEWRANNAAATAAEEKDGGGDDSEDDGLVFFVRSGFRESSRWAMLFWEGDQMVSWQANDGIKSSVVGLLSGGLSGFPLNHSDAGGYCTVDLPFLRYRRGEELLLRWMEVNAFTVVLRTHEGNKPASNCQFYSNSRTLAHFARCARMYKAWEFYRAELVAEAAAAGLPVARHLFLHYPEDERVQAMTYQQFLVGTELLVVPVLDKGRSAVTAYFPAGAGAWRHVWTGDEYGAGVQGGFEAEVEARVGYPGVFVRAGSPVGERFVSNLRDLKVL

>Z_mays (AFW79589.1)

MMASDRVESTARALRPFHSPVRAITAVAMASLKTAKKHHARLNNPFPRAVPTAAIHDGDGALRLSFASTSKLAHAHDFPVGTRFRLRWDPSRGGEVSLSRVPPSSGGGAMWETVPGVAFLSVASVATEADECRGSFALRDGGARLVPDRQHVDKIRAFYRCDAEAGGSEPDLLRAAAFRPSDATRFPVLVVTGLVSAAKKAGASPSCTCCGLRLRAAGRRARSAAAAARPVLSARYWILLEEKSDTQVRFSVKIGDYQWTCSHADPAKSPPATATTTTTTPRLHRASLRLRLSARVQRATTSKRTRLAPAPSREREEASALLPAPERAEERRPEEFNRVFLTYASSRDERFFGFGEQFSHVEFKRKRVPVLVQEQGIGRGDQPITFAANLVSYRSGGNWSTTYAPSPFYMTSKMRSLYLEGYEYSIFDLTRPDRVQIQIYGNSVQGRILDGDSPTELLTSYTESTGRPPVLPRWITSGAVVGMQGGTDAVRRVWGQLKDYDVPVSAFWLQDWVGQRKTAIGSQLWWNWELDDAHYNGWNDLVDDLRSHGIRTMAYCNPCLVPMDEKPSAKRHLFEEARKLGILVRDDAGEPYMMPNTAFDVAMLDLTNPAAHAWFKSTVLRGMVDRGVSGWMADFGEGLPLDARLHSGEDSVAAHNRYPELWARVNREIADEWRASRRRAAAESDDIVDDDDGLVFFVRSGFRESSRWAMLFWEGDQMVSWQANDGIKSSVVGLLSGGLSGFPLNHSDAGGYCTVDLPPFLRYRRSEELLMRWMEVNAFTVVLRTHEGNRPGSNCQFYSSGRTLAHFARCAKMYKAWEFYRARLVGEAARTGLPVARHLFLHYPADERVQALTYQQFLVGTEMLVVPVLDKGRTAVTAYFPAGAGAWRHVWSGNEYGTGAGVQGGFEAQVEARIGYPAVFVRAGSSVGERFVSNLRNLTVL

>O_sativa (NP_001042692.2)

MAPPPPPTPSPAQPKTRKKDHVRLNNPFPRAVPSSSLRHRDAAPPLSFSPSSKLAHGAHDFPVGPHFLLRWDPSLGGAVSLAPRRGGGGATMWETVPGVAFVSAASVDTEADECRGSFALRDGRARLVPDRQRVDRIRAVYRRDADADADADLLRVAGAAFQASEHEQARFPVVVITGVVSARKPTPSPSCLCGRRRAAAAAGRPVLSARYWILLEEKSDTQVAFRVNLGDYQWSCDHDRHATHPSPLPSPSPSTSPRTHRAGSILRLRLSTRVQRSSGGSKKKKKLAFAAAGVPADREELAPLVPAAAAKEELEFNRVWMTLASSREERFYGFGEQFSRVEFKGKRVPVLVQEQGIGRGDQPITFAANLVSYRSGGNWSTTYAPSPFYMTSKMRSLYLEGYDYSIFDLTKPDRVQIQVYGSSVQGRILHGGSPTELITSYTESTGRPPALPRWITSGAVVGMQGGTDAVRRVWKQLQDHDVPVSAFWLQDWVGQRKTSIGSQLWWNWEVDDDHYAGWNDLVRDLRRRGVRTMTYCNPCLVPMDKKANARRHLFEEAKKLGLLVRDAAGEPYMMPNTAFDVAMLDFTNPAARAWFKGEVLAVMARGGAAGWMADFGEGLPLNARLHSSGSGDDGPVAAHNRYPELWARVNREFADEWRSGEHRRVADDADDGDGDGELVFFVRAGFRESSRWAMLFWEGDQMVSWQANDGIKSSVVGLLTGGMSGFPLNHGDAGGYCTVDLPLLRYRRSEELLLRWLELSAFTVVFRTHEGNKPGSNCQFYSNNRTLAHFARCAKIYKAWEFYRIQLVEEAAEKGLPVARHLFLHYPEDQRVQKMTYQQFLVGTEMLVVPVLDKGRSTVTAYFPMSDGGLWKHVWTGDEFGGRTSRGGVGEGMSHGSEAEVEARIGFPAVFVRVGSTVGERFVRNLRDLKVL

>P_patens (XP_001760287.1)

MPKLNRVLLTYASEAGEKFYGFGEQFSCFDMKGKRVPIMVQEQGIGRGDQPITMAANLVSYRAGGDWHTTYAPSPHYLTSDMNSLFLEGYEHCVFDLTRPDCVQLQVHAGSMKGRILYGQNPPELIKEYTAAVGRMRELPAWITKGVIVGMQGGHKAVRDVWRKVKEYDVPLSAFWLQDWVGQRKTSVGWQLWWNWEVDRDHYQGWEELVRDFKACGVKTMTYCNPFIVPTDKKTNRQNDLFTIAQKAGYLVSDTIGATYMIPNTSFEAAMLDITNPETRRWFKHLMYEMVKTGVCGWMADFGESLPFDCCLHSGEDPATAHNKYPEMWAELNREFVEEWEQERLAEDFKDSDEDDDDDTLVFFMRAGYRGSPRSATLFWEGDQMVSWGANDGIKSAVTGLLSSGVSGYAFNHSDIGGYCTVDLPFIRYSRTEELLLRWMELNAFSVIFRTHEGNVPKANTQFYSNDTTLRHFTRSAKIYKAWEFYRRQLVKEAAAVGMPVVRHLFLHYPEDKYVQTIVYKQFLVGSEILVVPVLDKGHTQVQAYFPSGDVWEHVWTGHQYRAPATQGLKVWVQAPLGYPAVFVKKDSWVRQQFIHNLEKENILEKTW

>S_moellendorfii (XP_002963961.1)

NRVQLTYISHRNERFFGFGEQFSCLDLKGRRVPILVQEQGVGRGDQPITAAANILSYRSGGSWHTTYAPSPYYMTSDMRCLFLENYEYSVFDLRRHDRVQIQVHSGTMEGRVLHGQTPAELLMRYTESIGRMRELPDWIHQGAVIGMQGGTNAVNKIHAELKKHDVPIAAFWLQDWVGQRKTSIGWQLWWNWEADTDHYQGWGELVQELKSQGIRTMTYCNPLLASKRRNMLQEALAEGFLVRKPGGGVYMIKNTSFDAAVIDLTNPKARSWLKEILRDMISTGVSGWMADFGEALPFDSVIHSGNENPALLHNRFPELWAQLNREVVEEWEQEHKLQNGDIDKLVFFVRAGFMRSPKWSTLFWEGDQMVSWQRNDGIKSAVVGLLSSGISGYSLNHSDIGGYTTVNVPFLKYVRSEELLMRWMELNAFSVIFRTHEGNDPGANSQFYTNARTFQHFARCARIFKAWNFYRKELVKVPASRGMPVVRHLFLHYPQDEFVQRITYEQFLVGSELLVVPVLNRGKRKVRAYFPAGDVWVHVWTNRNYGSAEQSSSAWIDAPFGLPAVFVKAGSTVGSTFMSNLASLDGVPAS

>T_urartu (EMS48824.1)

MGHRILLMPSHNARCRVFTKGPLMTVIEPVQLATLEGAVEHRNYIMVDITGSSEDESIIDLMFTDSVKVPDSDKKDGAPADPSSPPPAATTAPRPHRINILSLRLRLAGRVHRSMSKKKKLSAGFPAQEEVSALLPPPERASAEEEARPEEFNRVFLTYASSLDERFYGFGEQFSCMEFKGRRVPVLVQEQGIGRGDQPITFAANLLSYRSGGNWSTTWITSGAVVGMQGGTDAVRRVWSQLRDHDVPVSALWLQDWVGQRKTAIGSQLWWNWEVDDDHYAGWKDLIRDLRCDGVRTMTYCNPCLVPMGEKGNARRHLYEEAKELGILVRDEAGEPYMMPNTAFDVAMLDFTNPEASSWFKGILRGMADEGVSGWMADFGEGLPLDARLHSGEDPVAAHNRYPELWARVNREFADEWKSEAGEDEEEGVVFFVRAGFRESSRWAMLFWEGDQMVSWQANDGIKSSVVGLLSGGLSGIPLNHSDAGGYCTVDLPLLRYRRSEELLMRWMEVNAFTVVFRTHEGNKPGSNCQFYSNSRILAHFARCAKMYKAWEFYRVQLVEEAAEKGLPVARHLFLHYPEDRRVQELTYQQFLVGTEMMVVPVLDKGRTAVTAYFPTSDGGSWRHVWTGEEFGGGHRSGHGSVGEATVHGFQAEVTADVGCPAVFVRVGSPVRERQSGYIYKEQPTCRARSQEVALD

>M_pusilla (XP_003058566.1)

KSVGLNQVILRYASDKHERIYGMGEQYSSLEHKGKRIPIITAEQGIGRGKQPVTFAFNRVFMGSGGNWHTTYTAIPHYVTSAARSVFLTNYSYAEFDFTDDETISILSTTPTNTLCGQIIGARSIPETVAAYTEYSGRMEPLPEWAYANGVILGMTGGSAKVRKVASMLRDAGVPLAGLWLQDWGGIRNTSIGIERVWWNWELDESNYPDWHALREEAAMNGTRLLTYTNPFLMDARGPKGRLYREAKEAGYMVQTVAGGVYRLGHEPGVSFGLLDLTNPAAVRWIEDVLHDMLKNTGAVGWMADFGEYLPFDCVLHSGEAPIAVHNRYPEDWAALNRRAMIRAGLGSKNEGGNGEGMFYSRSASSQTPRHTPLMWLGDQLVSWDAHDGMKSAVLGMLQGGLSGLALSHSDIGGYTATPGRHRTRELLMRWMELSALSDVVFRTHQGNRPLHNAQPWDTPELLDHLRDFARLHRALAPYRAELMRESSLTGMPLTRPLFMHYPHDVVASRVATQFLVGRDILAAPVMDRKTSRVHVYLPPGDTWLDVWTTQQAPAQPTGPRHGHGVWLTVDAPMGWPAVFIR

>O_lucimarinus (XP_001419443.1)

VGLNQLDFVYAMDPDERVYGLGEQFSSYNHRGRRVPVITGEQGMGRGVQPLSFMFNSVFPGSAGSWHTTYTAIPHYITHKARSVFLTNYTYSEFDFTEEESVVIRAAAPSGLITGQIIGGSSIPDVLRAYTDYAGRMTSLPEWAMNGVILGMTGGPQKVRQVYKTLGEGGVKVAGLWLQDWGGVRNTSIGIERVWWNWRLDETHYTDWDALREEIKPNGTHLLTYVNTFLMDANSDKGLLYREAKEKNYMVRDVKGEVYRLGSEPGVTFGLLDLSNPECVAWIEDIIVDMLETTGAMGWMADFGEYLPFDAVLHSGELPIEVHNRYPEDWAEVNRRAMRRAGLEGTGFFWSRSASTKSPKHSALFWLGDQMVSWDAYDGIKTAVLGGLSGGLSGLTLTHSDVGGYTAHPLKHRSEELLMRWMELNAFADAIFRTHQGNRPHHNAQPWNTPELVEHLKFCVDIHVALKPYKVELMREAQAVGLPMTRSMIIHYPYDTNAANIATQFLLGRDILVAPVLDKGATHVHVYLPPGDVWVDAWTTQRAPVQPDLIGSDEGGRGSWITVDTPMGWPAAFVRKS

>A_thaliana (CAB82818.1)

MASCLSLLVAIILCFSSLQCSNAIGKGYRLISMEKSPDDGSFIGYLQVKQSNKIYGSDITILRLFINYRTDHRLRVHITDAKKQRWEVPYNLLRREQPPNVIGKSRKSPVTVQEISGPELILIFTVDPFSFAVRRRSNGETIFNTSSSDESFGEMVFKDQYLEISTSLPKDASLYGFGENSQANGIKLVPNEPYTLFTEDVSAFNLNTDLYGSHPVYMDLRNVSGKAYAHSVLLLNSHGMDVFYRGDSLTYKVIGGVFDFYFFAGPSPLNVVDQYTSLIGRPAPMPYWSLVVKDVVDNYQKAKIPLDVIWNDADYMDGYKDFTLDLVNFPHAKLLSFLDRIHKMGMKYVVIKDPGIGVNASYGVYQRGMASDVFIKYEGKPFLAQVWPGPVYFPDFLNPKTVSWWGDEIRRFHELVPIDGLWIDMNEINATGHKASLGFKTIPTSAYHYNGVREYDAHSIYGFSEAIATHKALLAVQGKRPFILSRSTFVGSGQYAAHWTGDNQGTWQSLQVSISTMLNFGIFGVPMVGSDICGFFPPTPEELCNRWIEVGAFYPFSRDHADYYAPRKELYQWGTVAESARNALGMRYKLLPFLYTLNYEAHMSGAPIARPLFFSFPEFTECYGLSKQFLLGSSLMISPVLEQGKTQVEALFPPGSWYHMFDMTQVVVSKNGRLFTLPAPFNVVNVHLYQNAILPMQQVVAFPAGASEGYASGKLFLDDDELPEMKLGNGKSTYIDFYASVGNESVKIWSQVKEGQFALSQGLVIEKVIVLGLKGTWKVSEILLNGSSISNETKTIEVSSKEQMYVVGSEDEGESKSFMVELKGLEMLVGKDFNISWKMASTNVLSMAGNEVIAR

>E_salsugineum (XP_006399684.1)

MSPLQWFPTILIVVVVFFWSLPLPSHGSSQVLAEDEEVTVVGYGYVVRSVAVNSNQKVLTAKLDLIKPSSVYAPDIQTLSLHVSLETSERLRIRLRDSSQQRWEIPETVISRAGNHSLRRFLPEEGGGNSSENNILEDPSSDLVFTLHNTTPFGFSISRRSSGDVLFDTSPDPSDPNTYFVFKDQFLQLSSALPENRSNLYGLGEHTKRSFRLKPGDTMTMWNADIGSEHPDVNLYGSHPFYIDVRGSNGHDEAGTTHGVLLLNSNGMDVRYEGNRITYNVIGGIIDLYVFAGPSPEMVMNQYTGFIGRPAPMPYWSFGFHQCRYGYNNVSDLESVVDGYKNAGIPLEVMWTDIDYMDGYKDFTLDPVNFPEDKMKSFVDTLHKSGQKYVLILDPGINVNSSYGTYERGLKADVFIKRNGEPYLGEVWPGKVYYPDFLNPAAATFWSNEIKMFQETLPSDGVWLDMNELSNFITSPLSPGSSLDDPPYKINNLGGKTPINKKTVPATSIHFGNVSEYDAHNLYGLLEAKATHQAIVDLTGKRPFILSRSTFVSSGKYTAHWTGDNAAKWEDLAYSIPGILNFGLFGIPMVGADICGFSYDTTEELCRRWIQLGAFYPFARDHSSKGTAKQELYIWDSVASSARKVLGLRMRLLPHLYTLMYEAHVTGVPIARPLFFSFPSDTKTYEIDSQFLIGKSIMVSPALEKGTTTVDAYFPAGIWFDMFNYSIAVGGDSGKHVRLYTPADHVNVHVREGSKFSVCLFLSTTSFSVLTITVS

>A_lyrata (XP_002877443.1)

MASSLSLLVAIILCFSSLRCSNAIGKGYRLISIEKSPDDGGFIGFLQVKQSNKIYGSDITILRLFIKHETDHRLRVHITDAKRQRWEVPYNLLRREQPPRVIGKSRKSPVTVQEISGPELILSFTTDPFSFAVRRRSNRETIFNTSSSDENFGEMVFKDQYLEISTSLPKDSSLYGFGENSQPNGIKLVPNEPYTLFTEDVSAFKLNTDLYGSHPVYMDLRNVRGKSYAHSVLLLNSNGMDVLYRGGSLTYKVIGGVFDFYFFAGPSPLNVVDQYTSLIGRPAPMPYWSLGFHQCRWGYRNVSVLEEVVDNYQKAKIPLDVIWNDADYMDGYKDFTLDLVNFPHAKLLAFLDRIHKMGMKYVVINDPGIGVNASYGVYQRGMANDVFIKYEGKPFLAQMWPGPVYFPDFLNPKTVSWWGDEIRRFHELVPIDGLWIDMNEINATGNKAPLGFKTIPTSAYHYNGVREYDAHSIYGFSEAISTHKALLDVQGKRPFILSRSTFVGSGQYAAHWTGDNQGTWQSLQVSISTMLNFGIFGVPMVGSDICGFFPPTPEELCNRWIEVGAFYPFSRDHADYYAPRKELYQWGTVAESARNALGMRYKLLPFLYTLNYEAHMTGAPIARPLFFSFPDYTECYGLSKQFLLGSSLMISPVLEQGKTQVEALFPPGSWYHIFDMTQVVVSKNGKRVTLPAPLNVVNVHLYQNTILPMQQGRLVVTFPARASEGYATGKLFLDDDELPEMKIGNGQSTYVDFYASKVSFALRQGLVIERVIVLGLEGTEQTKRIEVSSKDQKYVVGSEDKGESKSFMVELKGLEILVGKDFNISWKMASTNGAH

>C_rubella (XP_006289249.1)

MSSLHWFTNIVFVVVVFCSTSLASPLHGSSQVVLEEETTVVGYGYVVRSVAVDSNEKLLTANLDLIKPSSVYAPDVKSLSLHASLETSERLRIRITDFSQQRWEIPENVIPRAGNHSPRRFVTEEVDGGNSPDSNFLTDPSSDLVFTLHNTTPFGFSVTRRSSGDILFDTSPDQSDPSTYFIFKDQFLQLSSALPENRSNLYGLGEQTKRSFRLIPGDTMTMWNADIGSENPDVNLYGSHPFYMDVRGSNGHDEAGTTHGVLLLNSNGMDVKYEGHRITYSVIGGVIDLYVFAGPSPEMVMNQYTELIGRPAPMPYWSFGFHQCRYGYKNVSDLESVVDGYAKARIPLEVMWTDIDYMDGYKDFTLDPVNFPEDKMKSFVDTLHKSGQKYVLILDPGIGVNRSYGTYNRGMEADVFIKRDGEPYLGEVWPGKVYFPDFLNPAAATFWSNEIKIFQDILPLDGLWIDMNELSNFITSPLTPGSSLDDPPYKINNFGDNAPINNKTVPATAIHFGNVSEYNAHNLYGLLEAKATHQAIVDVMGKRPFVLSRSTFVSSGKYTAHWTGDNAAKWEDLAYSIPGILNFGLFGIPMVGADICGFSHDTTEELCRRWIQLGAFYPFARDHSSLNTARQELYLWESVASSARKVLGLRMRLLPHLYTLMYEAHVSGIPIVRPLFFSFPQDTNTYEIDSQFLIGKSIMVSPALEQGKLTVDAYFPAGNWFDLFNYSFAVGGASGKHVRLDTPADHVNVHVREGSIVAMQGEAMTTRDARKTPYELLVVASRLENISGELFLDDGEKIQMGEGGGNRDWTLVKFRCYVTGKSVVLRSEVVNPEYASRMKWSIGKVTFVGFENVESVKTYEVRTGERLRSPRISLIKTVMDDDDPRFLSVEVSKLSLLVGKKFEMRLKLT

>B_vulgaris (3W37_A)

MERSKLPRYICATLAVVLPLVLCMVVEGATTSKNDNQGEAIGYGYQVKNAKVDNSTGKSLTALLQLIRNSPVYGPDIQFLSFTASFEEDDTLRIRITDANNRRWEIPNEVLPRPPPPPSPPPLSSLQHLPKPIPQNQPTTTVLSHPHSDLVFTLFHTTPFGFTIYRKSTHDVLFDATPIPSNPTTFLIYKDQYLQLSSSLPAQQAHLYGLGEHTKPTFQLAHNQILTLWNADIASFNRDLNLYGSHPFYMDVRSSPMVGSTHGVFLLNSNGMDVEYTGDRITYKVIGGIIDLYIFAGRTPEMVLDQYTKLIGRPAPMPYWAFGFHQCRWGYRDVNEIETVVDKYAEARIPLEVMWTDIDYMDAFKDFTLDPVHFPLDKMQQFVTKLHRNGQRYVPILDPGINTNKSYGTFIRGMQSNVFIKRDGNPYLGSVWPGPVYYPDFLDPAARSFWVDEIKRFRDILPIDGIWIDMNEASNFITSAPTPGSTLDNPPYKINNSGGRVPINSKTIPATAMHYGNVTEYNAHNLYGFLESQATREALVRTSNERPFLLSRSTFAGSGKYTAHWTGDNAARWDDLQYSIPTMLNFGLFGMPMIGADICGFAESTTEELCRRWIQLGAFYPFSRDHSARDTTHQELYLWESVAASARTVLGLRYQLLPYYYTLMYDANLRGIPIARPLFFTFPDDVATYGISSQFLIGRGIMVSPVLQPGAVSVNAYFPRGNWFSLFNYTSSVSVSAGTYVSLSAPPDHINVHIHEGNIVAMQGEAMTTQAARSTPFHLLVVMSDHVASTGELFLDNGIEMDIGGPGGKWTLVRFFAESGINNLTISSEVVNRGYAMSQRWVMDKITILGLKRRVRIKEYTVQKDAGAIKIKGLGLRTSSHNQGGFVVSVISDLRQLVGQAFKLELEFEGATR

**Alpha-glucosidase (All homologues of A. thaliana, V. vinifera, P. trichocarpa, S. lycopersicum, and M. truncatula; used for Figure 8b)**

>At_Gluc_1 (CAB82818.1)

MASCLSLLVAIILCFSSLQCSNAIGKGYRLISMEKSPDDGSFIGYLQVKQSNKIYGSDITILRLFINYRTDHRLRVHITDAKKQRWEVPYNLLRREQPPNVIGKSRKSPVTVQEISGPELILIFTVDPFSFAVRRRSNGETIFNTSSSDESFGEMVFKDQYLEISTSLPKDASLYGFGENSQANGIKLVPNEPYTLFTEDVSAFNLNTDLYGSHPVYMDLRNVSGKAYAHSVLLLNSHGMDVFYRGDSLTYKVIGGVFDFYFFAGPSPLNVVDQYTSLIGRPAPMPYWSLVVKDVVDNYQKAKIPLDVIWNDADYMDGYKDFTLDLVNFPHAKLLSFLDRIHKMGMKYVVIKDPGIGVNASYGVYQRGMASDVFIKYEGKPFLAQVWPGPVYFPDFLNPKTVSWWGDEIRRFHELVPIDGLWIDMNEINATGHKASLGFKTIPTSAYHYNGVREYDAHSIYGFSEAIATHKALLAVQGKRPFILSRSTFVGSGQYAAHWTGDNQGTWQSLQVSISTMLNFGIFGVPMVGSDICGFFPPTPEELCNRWIEVGAFYPFSRDHADYYAPRKELYQWGTVAESARNALGMRYKLLPFLYTLNYEAHMSGAPIARPLFFSFPEFTECYGLSKQFLLGSSLMISPVLEQGKTQVEALFPPGSWYHMFDMTQVVVSKNGRLFTLPAPFNVVNVHLYQNAILPMQQVVAFPAGASEGYASGKLFLDDDELPEMKLGNGKSTYIDFYASVGNESVKIWSQVKEGQFALSQGLVIEKVIVLGLKGTWKVSEILLNGSSISNETKTIEVSSKEQMYVVGSEDEGESKSFMVELKGLEMLVGKDFNISWKMASTNVLSMAGNEVIAR

>At_Gluc _2 (NP_201189.1)

MRSLLFVLSLICFCSQTALSWKKEEFRSCDQTPFCKRARSRTPGACSLIVGDVSITDGDLVAKLLPKAPNQGDGDQIKPLILSLSVYKDGIVRLKIDEDHSLNPPKKRFQVPDVVVSEFEEKKIWLQKVATETISGDTSPSSVVYVSDGYEAVVRHDPFEVYVREKSGDRRRVVSLNSHGLFDFEQLGRKTEGDNWEEKFRTHTDSRPSGPQSISFDVSFYDSSFVYGIPEHATSFALKPTKGPGVEESEPYRLFNLDVFEYDHESPFGLYGSIPFMVSHGKSGKTSGFFWLNAAEMQIDVLANGWDAESGISLPSSHSRIDTFWMSEAGIVDTFFFVGPEPKDVVKQYASVTGTSAMPQLFATGYHQCRWNYKDEEDVAQVDSKFDEHDIPYDVLWLDIEHTDGKRYFTWDSVLFPHPEEMQKKLAAKGRKMVTIVDPHIKRDDSYFLHKEATQMGYYVKDSSGKDFDGWCWPGSSSYIDMLSPEIRKWWGGRFSYKNYVGSTPSLYTWNDMNEPSVFNGPEVTMPRDALHVGGVEHREVHNAYGYYFHMATSDGLVMREEGKDRPFVLSRAIFPGTQRYGAIWTGDNTAEWEHLRVSIPMILTLGLTGITFSGADIGGFFGNPEPELLVRWYQVGAYYPFFRGHAHHDTKRREPWLFGERNTELMRDAIHTRYTLLPYFYTLFREANVTGVPVVRPLWMEFPQDEATFSNDEAFMVGSGLLVQGVYTKGTTQASVYLPGKESWYDLRNGKTYVGGKTHKMDAPEESIPAFQKAGTIIPRKDRFRRSSSQMDNDPYTLVVALNSSQEAEGELYIDDGKSFEFRRGSYIHRRFVFSKGVLTSTNLAPPEARLSSQCLIDRIILLGHSSGPKSALVEPLNQKAEIEMGPLRMGGLVASSGTKVLTIRKPGVRVDQDWTVKIL

At_Gluc_3 (AAD05539.1)

AFSLSLLLALILCFSPTQSYKTIGKGYRLVSIEESPDGGFIGYLQVKQKNKIYGSDITTLRLFVKHETDSRLRVHITDAKQQRWEVPYNLLPREQPPQVGKVIGKSRKSPITVQEISGSELIFSYTTDPFTFAVKRRSNHETLFNTTSSLVFKDQYLEISTSLPKEASLYGLGENSQANGIKLVPNEPYTLYTEDVSAINLNTDLYGSHPMYMDLRNVGGKAYAHAVLLLNSNGMDVFYRGDSLTYKVIGGVFDFYFIAGPSPLNVVDQYTQLIGRPAPMPYWSLGFHQCRWGYHNLSVVEDVVDNYKKAKIPLDVIWNDDDHMDGHKDFTLNPVAYPRAKLLAFLDKIHKIGMKYIVINDPGIGVNASYGTFQRAMAADVFIKYEGKPFLAQVWPGPVYFPDFLNPKTVSWWGDEIKRFHDLVPIDGLWIDMNEVSNFCSGLCTIPEGKQCPSGEGPGWVCCLDCKNITKTRWDDPPYKINATGVVAPVGFKTIATSATHYNGVREYDAHSIYGFSETIATHKGLLNVQGKRPFILSRSTFVGSGQYAAHWTGDNQGTWQSLQVSISTMLNFGIFGVPMVGSDICGFYPQPTEELCNRWIEVGAFYPFSRDHANYYSPRQELYQWDTVADSARNALGMRYKILPFLYTLNYEAHMTGAPIARPLFFSFPEYTECYGNSRQFLLGSSFMISPVLEQGKTEVEALFPPGSWYHMFDMTQAVVSKNGKRVTLPAPLNFVNVHLYQNTILPTQQGGLISKDARTTPFSLVIAFPAGASEGYATGKLYLDEDELPEMKLGNGQSTYVDFYASVGNGTMKMWSQVKEGKFALSKGWVIEKVSVLGLRGAGQVSEIQINGSPMTKKIEVSSKEHTYVIGLEDEEENKSVMVEVRGLEMLVGKDFNMSWKMGIN

>At_Gluc_4 (NP_196733.1)

MSSLHWFPNIFIVVVVFFSLRSSQVVLEEEESTVVGYGYVVRSVGVDSNRQVLTAKLDLIKPSSVYAPDIKSLNLHVSLETSERLRIRITDSSQQRWEIPETVIPRAGNHSPRRFSTEEDGGNSPENNFLADPSSDLVFTLHNTTPFGFSVSRRSSGDILFDTSPDSSDSNTYFIFKDQFLQLSSALPENRSNLYGIGEHTKRSFRLIPGETMTLWNADIGSENPDVNLYGSHPFYMDVRGSKGNEEAGTTHGVLLLNSNGMDVKYEGHRITYNVIGGVIDLYVFAGPSPEMVMNQYTELIGRPAPMPYWSFGFHQCRYGYKNVSDLEYVVDGYAKAGIPLEVMWTDIDYMDGYKDFTLDPVNFPEDKMQSFVDTLHKNGQKYVLILDPGIGVDSSYGTYNRGMEADVFIKRNGEPYLGEVWPGKVYFPDFLNPAAATFWSNEIKMFQEILPLDGLWIDMNELSNFITSPLSSGSSLDDPPYKINNSGDKRPINNKTVPATSIHFGNISEYDAHNLYGLLEAKATHQAVVDITGKRPFILSRSTFVSSGKYTAHWTGDNAAKWEDLAYSIPGILNFGLFGIPMVGADICGFSHDTTEELCRRWIQLGAFYPFARDHSSLGTARQELYLWDSVASSARKVLGLRMRLLPHLYTLMYEAHVSGNPIARPLFFSFPQDTKTYEIDSQFLIGKSIMVSPALKQGAVAVDAYFPAGNWFDLFNYSFAVGGDSGKHVRLDTPADHVNVHVREGSIVAMQGEALTTRDARKTPYQLLVVASRLENISGELFLDDGENLRMGAGGGNRDWTLVKFRCYVTGKSVVLRSEVVNPEYASKMKWSIGKVTFVGFENVENVKTYEVRTSERLRSPRISLIKTVSDNDDPRFLSVEVSKLSLLVGKKFEMRLRLT

>At_Gluc_5 (NP_566736.1)

MTLSGDSSETVEMTSTDMIFEPILEHGVFRFDCSVDHRKAAFPSVSFKNSKDREVPIVSHIVPAYIPTCGCLQDQQVVTFEFSPGTSFYGTGEVSGQLERTGKRVFTWNTDAWGYGSGTTSLYQSHPWVLVVLPTGETLGVLADTTRKCEIDLRKEGIIRIISPASYPIITFGPFSSPTAVLESLSHAIGTVFMPPKWALGYHQCRWSYMSDKRVAEIAQTFRDKKIPSDVIWMDIDYMDGFRCFTFDKERFPDPSALAKDLHSNGFKAIWMLDPGIKQEEGYYVYDSGSKNDVWISRADGKPFTGEVWPGPCVFPDYTNSKARSWWANLVKEFVSNGVDGIWNDMNEPAVFKVVTKTMPENNIHHGDDELGGVQNHSHYHNVYGMLMARSTYEGMELADKNKRPFVLTRAGFIGSQRYAATWTGDNLSNWEHLHMSISMVLQLGLSGQPLSGPDIGGFAGNATPRLFGRWMGVGAMFPFCRGHSEAGTDDHEPWSFGEECEEVCRAALKRRYQLLPHFYTLFYIAHTTGAPVAAPIFFADPIDSRLRAVENGFLLGPLLIYASTLSSQGSHELQHILPRGIWHRFDFADSHPDLPTLYLQGGSIISLAPPHLHVGEFSLSDDLTLLVSLDENGKAKGLLFEDDGDGYGYTKGRFLVTHYIAERDSSTVTVKVSKTEGDWQRPNRRVHVQLLLGGGAMLDAWGMDGEFIHIKVPSESGISELISTSNERFKLHMENTKLIPEKEVVPGQKGMELSKEPVELSSGDWKLNIVPWVGGRILSMTHVPSGIQWLHSRIDINGYEEYSGTEYRSAGCTEEYNVIERDLEHAGEEESLILEGDVGGGLVLRRKISIAKDNQRVFRIASSIEARSVGAGSGGFSRLVCLRVHPTFTLLHPTESFVSFTSIDGSKHEVWPDSGDQIYEGNNLPHGKWMLVDKSLNLRMVNRFDVSQVFKCIIHWDCGTVNLELWSKERPVSKESPLKIEHEYEVTSFP

>Vv_Gluc_1 (CBI30134.3)

MVYYQKKIIKYRSCLIHCLLSCGKFHIWASQHLNMAALKISKKHHKHLNNPFPSTPSSLPLLRGSLFFNPQTVPSDQTFIVGKDFQVLWSTDNGGSLSISHQSHPSRPIWSTVPGQAFVSAALAETEVEESRGSFAIKDGNVHLLCNDQTVEDIRLINENDCYLEANELDFLSGNQGLDQKPYLKDTQFPILLLTGWVFRKKKKSFQNTEIHERLQLEAERSTYARYWVLFDQKTSNQIGFQVKFGKPNFEFRSRAFATASRRFRGLKRKLRRTGRSRLGWCWSFSRPRGFVKVSSSEEEKEEKVAESIGFNRVCLTYSSEENERFYGFGEQFSHLNFKGKRIPIFVQEQGIGRGDQPITFAVNLVSYRAAGDSSTTYAPSPHYLTSKMRSLYLEGYDYSVFDLTRKDRVQIQIHGDSVQGRILHGNSPSELIERFTETIGRLPELPEWIISGAVVGMQGGTDSVRQVWEKLQAHNTPVSAFWLQDWVGHRETLIGSQLWWNWEVDTARYWGWQNLIKDLSAQHIKVMTYCNPCLAPTNEKPNRRRDLFEEAKKLDILVKDKNGDTYMVPNTAFDVGMLDLTHPDTASWFKQILQEMVDGGVRGWMADFGEGLPVDASLYSGEDPIAAHNRYPELWAQMNREFVEEWKSAHSGKAREDPEEALVFFMRAGFRNSPKWGMLFWEGDQMVSWQANDGIKSAVVGLLSSGISGYAFNHSDIGGYCAVNLPVIKYRRSEELLLRWMEVNAFTVVFRTHEGNKPSCNSQFYSNHKTLAHFARFAKVYKAWKFYRVQLVKEAAQKGLPVCRHLFLHYPNDEHVHKLSYQQFLVGTEILVVPVLDRGKKDVKAYFPVGESCSWQHIWTGKLFAKPGSEVWVEAPIGHPAIFVKEGSIIGETFLKNLREFNIL

>Vv_Gluc_2 (XP_002268690.1)

MPPNINSTLPLLLLFLLFTLHLSSVSAWKKEEFRTCNQTPFCKRARSRKPHSSSLFATDVAILDGALTANLRQPPPESPDQDQIKPLLFTLSVYQNGVVRVKIDEDPSLDPPKKRFEVPDVILPEFESTKLWLQRFQTETVDGDSGPSSVVYVADGYEAVLRHNPFEVYVREKQGKRRVLSLNSHGLFDFEQLRVKQEGDDWEERFKGHTDVRPYGPQSISFDVSFFDADFVYGIPEHASSFALRPTRGPGVDDSEPYRLFNLDVFEYIHDSPFGLYGSIPFMLGHGKARGTSGFFWLNAAEMQIDVLGSGWDAESGILLPESGSRIDTFWMSEAGIVDTFFFIGPGPKDVVRQYTSVTGMPAMPQLFSTAHHQCRWNYRDEEDVENVDSKFDEHDIPYDVLWLDIDHTDGKRYFTWDRVLFPNPEQMQNKLAAKGRHMVTIVDPHIRRDESFHLHKEATSKGYYVKDATGKDYDGWCWPGSSSYPDMLNPEIRSWWSEKFSLKNYVGSTPWLYIWNDMNEPSVFNGPEVTMPRDALHYGGVEHRELHNAYGYYFHMATSDGLVKRGDGKDRPFVLSRAFFPGSQRHGAIWTGDNTADWDQLRVSVPMILTLGLTGMTFSGADVGGYFGNPEMELLVRWYQLGAYYPFFRAHAHQDTKRREPWLFGERNMELMRDAIHTRYALLPYFYTLFREANTSGVPVMRPLWMEFPSDKATFSNDEAFMVGNSLLVQGIYTERAKYASVYLPGGQSWYDLRTGIIYKGGTTHKLEVSEETIPAFHRAGTIIPRKDRYRRSSTLMANDPYTLVIALNSSHAAEGELYIDNGKSFEFKQGAYIHRHFVFSDGKLTSSSLVPNASKTLFSSACVIERIIVLGHSSGPKNALIEPSNRKAEIELGPLWLRRGKSAPVLTIRKPNVPVADDWTIKIL

>Vv_Gluc_3 (CAN66951.1)

MALNHSNCDLECFSVLCFSNSKNEPVGXGYRVRSVSFDPSGKSLTARLDLIKPSPVFGPDVRNLILVASLETNDRLRIRITDSEHQRWEIPREILPRYTQLHRRVLPQNHSISPEDDHNSPENNIVSDPKSDLVFTLRRTTPFGFIVSRRSTGDILFDASSDASDAGTFLVFKDQYLQVSSALPILRSSLYGLGEHTKKTFKLAQNQTLTLWNTDIYSSNLDVNLYGSHPFYMDVRLTDNRGKVPMGTTHGVLLLNSNGMDIVYTGDRITYKAIGGVLDFYFFSGPTPEMVVQQYTELIGRPAPMPYWSFGFHQCRYGYMNXSDVEGVVAGYAKAGIPLEVMWTDIDYMDAYKDFTLDPINFPLDKMKKLVDTLHQNGQKYVLILDPGISVNQTYGTYKRGMEADIFIKRDGIPYLGSVWPGPVYFPDFVNPATEIFWGGEIKIFRDSLPIDGLWLDMNEISNFITSPPTPLSTLDDPPYKINNAGVRRPINNRTVPATSLHFGNITEYNAHNLYGILESKATSAALTKLTGKRPFILTRSTFVGSGKYAAHWTGDNAATWDDLAYSIPAVLNFGLFGIPMVGADICGFSGDKNEELCRRWIQLGAFYPFARDHSAKFTIRQELYVWDSVAATAKKVLGLRYRLLPYFYTLMYEAHTKGVPIARPLFFSFPQDPXTYGIBFQFLIGKGVMVSPVLKPGXVSVKAYFPSGNWFDLFNYSNAVSAGSGKYTTLDAPPDHINVHVREGNILXMQGEAMXTKAARKTPFQLLVVLSSSGISTGEVFLDDGEEVEMGGGGKNWSLVKFYAWVEDKKAIVGSEVMNGGFALSQKWIIDRVTLIGLTKAQTKRFKGFEVYTNEGTKTIGDSSLKVDLDGNRKFVVMEXXKLXLPIGKEFELKLNLT

>Vv_Gluc_4 (XP_002270200.1)

MAPNINPTLPLLLLLLLFTLHLSSVSAWKKEEFRTCNQTPFCKRARSRKPHSSSLFATDVAILDGALTANLRQPPPESPDQDQIKPLLFTLSVNQNGVVRVKIDEDPSLDPPKKRFEVPDVVLPEFESTKLWLQRFQTETVDGDSGPSSVVYVADGYEAVLRHNPFEVYVREKQGKRRVLSLNSHGLFDFEQLRVKQEGDDWEERFKGHTDVRPYGPQSISFDVSFFDADFVYGIPEHASSFALRPTRGPGVDDSEPYRLFNLDVFEYIHDSPFGLYGSIPFMLGHGKARGTSGFFWLNAAEMQIDVLGSGWDAESGILLPESGGRIDTLWMSEAGIVDTFFFIGPGPKDVVRQYTSVTGTPAMPQLFSTAYHQCRWNYRDEEDVENVDSKFDEHDIPYDVLWLDIEHTDGKRYFTWDRVLFPNPEQMQNKLAAKGRHMVTIVDPHIKRDESFHLHKEATSKGYYVKDATGKDYDGWCWPGSSSYPDMLNPEIRSWWSEKFSLKNYVGSTPWLYIWNDMNEPSVFNGPEVTMPRDALHYGGVEHRELHNAYGYYFHMATSDGLVKRGDGKDRPFVLSRAFFSGSQRYGAVWTGDNTADWDQLRVSVPMILTLGLTGMTFSGADVGGFFGNPETELLVRWYQLGAYYPFFRAHAHHDTKRREPWLFGERNTELMRDAIHTRYALLPYFYTLFREANTSGVPVMRPLWMEFPSDKATFSNDEAFMVGNSLLVQGIYTEQVKHASVYLPGGQSWYDLRTGIIYKGGTAHKLEVSEETIPAFQRAGTIIPRKDRYRRSSTQMANDPYTLVIALNGSHAAEGELYIDDGKSFEFKQGAYIHRHFVFSDGKLTSSSLVPNAGRTLFSSACVIERIIVLGHSSGPKNALIEPSNRKAEIELGPLWLRRGKSAPVLTIRRPNVPVADDWTIKIL

>Vv_Gluc_5 (XP_002282429.1)

MASSYLLLVVSILCIYGGCGALAAIPAKIGKGYRLISIEETANGGLLGHLQVKQKNNIYGADIPHLQLHVKHETQDRLRVHITDAEKQRWEVPYDLLPREKPLPLRQAIGRSRKTLSTPTDYPGSELIFSYTTDPFGFAVRRKSTGETLFNTTSDDSDRYGNMVFKDQYLEISTKLPKDASLYGLGENTQPHGIKLYPNDPYTLYTTDISAINLNADLYGSHPVYMDLRNTGGKAYAHSVLLLNSNGMDVFYKGSSLTYKVIGGVFDFYFFGGPTPLSVVDQYTSLVGRPAPMPYWSLGFHQCRWGYHNLSVVEDVVENYKKAQIPLDVIWNDDDHMDGHKDFTLNPVNYPRPKLLEFLNKIHDRGMKYIVIIDPGIGVNSTYGVYQRGMANDVFIKYDGEPFLAQVWPGPVYFPDFLNPKTVSWWGDEIRRFHELVPVDGLWIDMNEASNFCTGKCTIPKGKVCPSGTGPGWICCLDCKNITKTRWDDPPYKINASGLEVPIGYKTIATSAVHYNGVLEYDAHSLYGFSQSIATHKGLQGLEGKRPFILSRSTYVGSGKYAAHWTGDNKGTWDDIKYSISTMLNFGIFGVPMVGSDICGFYPAPTEELCNRWIELGAFYPFSRDHANYYSPRQELYQWDSVAKSARNALGMRYKLLPYLYTLNYEAHISGAPIARPLFFTFPTFSKCYEVSTQFLLGSGVLVSPVLDKGKTKVNALFPPGTWYSLFDLKETIVSEGDYRSLDAPLHVINVHVYQNTILPMQQGGLISKEARMTPFTLIVTFPAGATEGHAEGKLYLDDDELPEMTLGNGFSTYVDLHATVENKMVKVWSDVAEGKYALEKGWTIEKITVLGLSGSGESFALEVDGSSVSDVSHVQLTASEQHVATDKLEDEGDTRKSMMIEIQGLDLPVGKNFAMSWKMGVHG

>Vv_Gluc_6 (CBI37476.3)

MAGALTFSSDGVGALGFWKTHQYGFISGGFNRPLLKNCVPRHPSSPSRPFRKRVKKRLIGERLVIKMAEYEGKVVPADFTSGNMLFEPILEEGVFRFDCSSDDRDAAFPSLSFTNQKNRDMPIMNHKVPMYTPTFECVLGQQIVTIELPTGTSFYGTGEVSGQLERTGKRVFTWNTDAWGYGSGTTSLYQSHPWVLAVLPNGEALGILADTTRRCEIDLQKESIVKFSASSSYPIITFGPFASPTAVLTSLSHAIGTVFMPPKWSLGYQQCRWSYDSAVRVLEVARTFREKGIPCDVIWMDIDYMDGFRCFTFDQERFSDPKSLGKDLHLNGFKAIWMLDPGIKQEDGYFVYDSGSANDVWIHKADGTPFVGKVWPGPCVFPDFTQSKARSWWACLVKDFISNGVDGIWNDMNEPAVFKTVTKTMPEDNVHRGDAELGGCQNHSHYHNVYGMLMARSTYEGMKLANENKRPFVLTRAGYIGSQRYAATWTGDNLSNWDHLHMSISMVLQLGLSGQPLSGPDIGGFAGNATPRLFGRWMGVGAMFPFCRGHSETGTVDHEPWSFGEECEEVCRLALKRRYRLIPHIYTLFYMAHTTGTPVATPTFFADPKDPSLRTVENSFLMGPLLIYASTIPDQGLDELQHKLPKGIWLSFDFDDSHPDLPALYLQGGSIIPLGPPHQHVGEADPTDDLILLVALDEHGKAEGVLFEDDGDGYEFTTGGYLLTYYVAELQSSVVSVRVSKTEGSWKRPKRGLHVQLLLGGGAKIDAQGTDGEVLQITMPSEHEVSDLVSTSKEQYRNRLESAKHIPDVQEVSGHKGIELSSTPIELKSGDWALKVVPWIGGRIISMMHLPSGTQWLHSRIEANGYEEYSGVEYRSAGWSEEYTIVERNLEQAGEEESLKLEGEIGGGLVIERQISLPKDNSKVFRVDSGIIAHNVGAGSGGYSRLVCLRVHPMFNLLHPTESFVSFVSIDGSKHEVWPEAGEQSYEGNLRPNGEWMLVDKCLGLALVNRFDITEVHKCLVHWGTGTVNLELWSEQRPVSKQSPLTISHEYEVRVIP

>Pt_Gluc_1 (XP_002308887.1)

MGTLKITKKHHKHLNNPFPSTPRSLPFIQGRLLFNSQTVPPNKIFSVGKDFQLLWSIKNGGSLSIYHQSQPTKALWSTIPGQAFVTAALCETEVEESRGSFAIKDRNVYLVCDHQTIEDIRVISEPDHHFDQENDHDLSSGNMSFAQKNDWKDTQFPALVITGWLFSNRRKKRHQESGIYKDIQFETRGPPTCARYWVLFDQKNNNQIGFQVRVGPPNFEFQQRISPTPLGRHRRLRWKLGKIRRRKLGWYRFFTRSRGFVAVSSSSEEEMEMKSAELTEFNRVCITYSSEGNERFYGFGEQFSHMDFKGKRVPIFVQEQGIGRGDQPITFAANLVSYRAGGDWSTTYAPSPFYMTSKMRSLYLEGYDYSVFDMTRHDRVQIQIQSNSVRGRILNGNSPSEIIENFTETIGRPPELPKWIISGAVVGMQGGTEAVRRVWDELKDHKVPVSAFWLQDWVGQRETMIGSQLWWNWEVDTTRYHGWQQLINDLGAKNINVMTYCNPCLAPTDEKPNQRRNLFEEAKKLDILVKDKYGEPYMVPNTAFDVGMLDLTHPDTAAWFKQVLQEMVDDGVKGWMADFGEGLPVDATLYSGEDPISAHNRYPELWAQINREFVEEWKSGRAGKEREDPEEALVFFMRAGFRDSPKWGMLFWEGDQMVSWQANDGIKSSVVGLLSSGISGYAFNHSDIGGYCAVNLPFIKYHRSEELLMRWMELNAFTTVFRTHEGNKPSCNSQFYSNHKTLSHFARCAKLYKAWYFYRIQLVKEAARKGLPVCRHLFLHYPNDRNVHSLSYQQFLIGTEILVVPVLDKGKKNVKAYFPEGETCSWQHIWSGKLFKEQGSEAWVEAPVGYPPVFIKAGSTVGETFVENLRNFGIL

>Pt_Gluc_2 (XP_002310537.1)

MKPLAPLILSLLFLTSQTVLSFKREEFRNCHQTPFCKRARSRSPGACTLTPHSISISNGDLTAKLLSKTDEQIRPLILSLSVYQDGILRLKIDEDYNHPDPPVPKRRFQVPDVVLPEFESNKLWLQRLSTETVDGESSPSTVVYLSDGYDAVLRHDPFEIYIRDKKSGNQKLISLNSHQLFDFEQLRVKQEKQDSDNNEDSGSDDNWEERFRSHTDTRPYGPQSISFDVSFYNAEFVSGIPEHATSLALKPTRGPGVEKDSEPYRLFNLDVFEYLNESPFGLYGSIPLMISHGKEGRSAGFFWLNAAEMQIDVLGDGWDAESGIELVKQKSIDTFWMSEAGIVDAFFFVGPEPKDVVKQYTSVTGRPSMPQLFSIAYHQCRWNYRDEEDVENVDAKFDEHDIPYDVLWLDIEHTDGKRYFTWDPVLFPNPEEMQKKLAAKGRHMVTIVDPHIKRDDSFRLHKEATEKGYYVKDASGKDFDGWCWPGSSSYLDMVNPEIRSWWGDKFSYENYVGSTPSLYIWNDMNEPSVFNGPEVSMPRDALHHEGIEHRELHNAYGYYFHMATSNGLLKRGGGNDRPFVLSRAFFPGSQRYGSVWTGDNTADWDHLRVSVPMILTLGLSGISFSGADVGGFFGNPEPELLVRWYQLGAFYPFFRAHAHQDTKRREPWLFGEKNTRLIREAIRVRYMLLPYFYTLFREANTTGLPVMRPLWMEFPSDEITFSNDEAFMVGSSLLVQGIYTERAKYTSVYLPGKELWYDIRTGAAYKGGKTHKLEAKEESVPAFQRAGTIIPRKDRLRRSSTQMVNDPYTLVIALNSSQAAEGELYIDDGKSYEFLQGAYIHRRFVFANGKLTSINLAPSSSSKSQFSSKSILERIILLGYSPGPKNALIEPANQEVEVELGPLMLEGGRGSSVVTIRKPAVQVSDDWTIKIL

>Pt_Gluc_3 (XP_002307054.1)

MKPLLSLLLSLLFLSSQTVLSFKRDEFRNCNQTPFCKRARSRSRSPGGCTLTPHNIAISNGDLTATLLSKHEDQVRPLILSLSVYHDGILRLKIDEDYDHSDPPASKRRFQVPDVIIPEFESNKFWLQRLSTETLNDESSPSTVVYLSEGYDAVLRHDPFEIYVRDKQSGNQKLISLNSHQLFDFEQLRVKKEGEEDSETWEERFRGHTDTRPYGPQSISFDVSFYNAEFVSGIPERATSLALKPTRGPGVEKGLEPYRLFNLDVFEYLSESPFGLYGSIPLMISHGKEGRSAGFFWLNAAEMQIDVLGDGWDAESGIEWVKQKRIDTFWMSEAGIVDAFFFVGPGPKDIVRQYTSVTGRASMPQMFSIAYHQSRWNFRDEEDVENVDAKFDEHDIPYDVLWLDIDHTDGRRYFTWDSVLFPHPEEMQKKLAAKGRHMVTIVDPHIKRDNSFRLHKEATEKGYYVKDASGKDFDGWCWPGSSSYLDMVNPEIRSWWGDKFSYKNYVGSTPSLYIWNDMNEPSVFNGPEVSMPRDALHLGGIEHRELHNSYGYYFHMATSNGLLKRGGGNDRPFVLSRAFFPGSQRYGSVWTGDNTADWDHLRVSVPMILTLGLTGISFSGADVGGYFGNPGPDLLVRWYQLGAFYPFFRGHAHQDTKRREPWLFGEKNKRLIREAIHVRYMLLPYFYTLFREANTTGLPVMRPLWMEFPSDEATFSNDEAFMVGSSLLVQGIYTEGAKYASVYLPGKELWYDIRTGDAYKGGKTHKLEAAESVPAFQRAGTIIPRKDRLRRSSTQMVNDPYTLVIAVNSSQAAEGELYMDDGKSFEFLQGAYIHRRFVFANGKLTSINLAPSSSSKSQFSSKSMIERIILLGYAPGPKNAQIEPGNQKVEVELGQLMLEGRHGSSVVTIRKPAVQVSDDWTIRLL

>Pt_Gluc_4 (XP_002315944.1)

MFSFLSLSFFNYLLLLLLCFHLVNSSSTPTKIGNGYRLISLKETPDGGIGGLLQVKERNNIYGPDIPLLQLYVKHETQDRLRVRITDAEKQRWEVPYNLLPREQAPALKQTIGRSRKNLITTVQEYSGAELIFNYIADPFSFSVKRKSNGQTLFNSSSDGSSSFGVMVFKDQYLEISTQLPNDASLYGLGENTQPHGIKLFPGDPYTLYTTDISAINLNADLYGSHPVYMDLRNVKGQAYAHAVLLLNSNGMDVFYRGTSLTYKIIGGVFDFYFFSGPSPLAVVDQYTSLIGRPAAMPYWAFGFHQCRWGYHNLSVVEDVVENYKNAQIPLDVIWNDDDHMDGHKDFTLNPNNYPRPKLLAFLEKIHSIGMKYIVLIDPGIGVNSSYGVYQRGIANDVFIKYQGEPYLAQVWPGAVNFPDFLNPKTVEWWGDEIRRFHELVPVDGLWIDMNEASNFCSGLCKIPKDKQCPSGTGPGWDCCLDCKNITETRWDDPPYKINASGLQVPIGYKTIATSAVHYNGVLEYDAHSIYGFSQAIATHKALQGLEGKRPFILSRSTYVGSGKYAAHWTGDNKGTWEDLKYSISTMINFGIFGVPMVGSDICGFYPAPTEELCNRWIEVGAFYPFSRDHANYYSPRQELYQWESVAKSARNALGMRYKILPYLYTLNYEAHTTGAPIARPLFFSFPDYTECYGLSTQFLLGSSLMISPVLEQGKSQVKALFPPGSWYNMFDMTQSITSEGGQYVTLDAPLHVVNVHLYQNSILPMQQGGLISKEARMTPFTLLVSFPAGATDGKAAGKLFLDDDELQEMKLGSGSATYVDFYATVSEGTVKLWSEVQESKFALDKGWKIVKVTVLGLGGSGAPSSLEVDGKPVTGASNIELSSLEQKYITNLEVGDEKKKIMMVEVHGLEIPVGKNFAVSWKMGVSG

>Pt_Gluc_5 (XP_002317680.1)

MTAKLAYFILCFFLASCLAPLSISNGEVESQPVGYGHKVVSARVDPSVNVLAADLQLIKNSSTFGPDIQNLNFIASFDTKDRLRIRITDANKQRWEIPQDIIPRPKHNLSFGQNHVQSSLANYILSDPNSDLFFTLHNTTPFGFSLSRHSSGDVLFDASPNTSDSETFFVFKDQYIQLSFSLPKDRSSLYGLGEHTKKSFKLEPDKTPLTLWNADIASAVPDVNLYGSHPFYVDVRSESLDGKVIAGTTHGVLLLNSNGMDIIYEGDRITYKVIGGVIDLYIFAGPLPELVVQQYTELIGRPAPMPYWSFGFHQCRWGYKNVSDVEGVVAGYAKAGIPLEVMWTDIDYMDGFKDFTLDPVNFPLEKMKKFTDTLHQNGQKYVLILDPGISVNTTYGTYIRGMKADVFIRHDGIPYMGEVWPGSVYFPDFLNEAGREFWSNEIKLFHELLPFDGLWLDMNEISNFITPSSTEFSKLDDPPYKINNAAVQKPINNKTIPATSLHNGDIVEYNAHNLYGLSESKATNAALINVTGKRPFILSRSTFVGSGKYTAHWTGDNAATWDDLAYTIPSILNFGLFGIPMVGSDICGFSRNTTEELCRRWIQLGAFYPFARDHSAIDSTRQELYLWDSVAAAAKKVLGLRYQLLPYFYTLMYEAHMKGTPIARPLFFSFPQDIKTYGINSQFLVGKGVMVSPVLNSGAVSVDAYFPAGKWFDLFNHTNSVTADSGKYIKLDAPADHINVHVREGNILTLQGEAMTTKEARRTAFHLLVVLSSNENSTGEVFLDDGESVEMGGEGKNWSLVRFYGGIVGDMAMVRSIIINGEYALSQEWIVSKVTFIGLEKTKGFKWYELQTPKETKSGNSGTVASFNSNGELGMLEMSGFSLSLGEEFKLEVKLSI

>Pt_Gluc_6 (XP_002311455.1)

MFSSTVNSSSTPTKIGKGYRLISIEETPDGGIVGILQVKQNNKIYGPDIPLLQLYVKHETQDRLRVHITDAEKQRWEVPYNLLPREKAQALKQTIGRSRKNPITVQEYSGSELIFSYIADPFSFAVKRKSNGQTLFNSSSDGSGSFGEMVFKDQYLEISTQLPKDASLYGLGENTQPHGIKLYPGDPYTLYTTDISAINLNADLYGSHPVYMDLRKVKGQAYAHAVLLLNSNGMDVFYRGTSLTYKIIGGVFDFYFFSGPSPLAVVDQYTALIGRPAPMPYWAFGFHQCRWGYHNLSVVEDVVENYKKAQIPLDVIWNDDDHMDGHKDFTLNLVNYPRPKLLAFLEKIHSIGMKYIVIIDPGIGVNSSYGVYQRGIANDVFIKYEGEPYLAQVWPGAVNFPDFLNPKTVDWWGDEVRRFHELVPVDGLWIDMNEASNFCSGLCKIPKGKQCPSGTGPGWVCCLDCKNITKTRWDDPPYKINASGLQVPIGYKTIATSAVHYNGVLEYDAHSLYGFSQAIATHKALQGLEGKRPFILSRSTYVGSGKYAAHWTGDNKGTWEDLKYSISTMINFGIFGVPMVGSDICGFYPAPTEELCNRWIEVGAFYPFSRDHANFYSPRQELYQWDSVAESARNALGMRYKILPYLYTLSYEAHTTGAPIARPLFFSFPDYTECYGLSTQFLLGSSLMISPVLEQGKSQVKALFPPGSWYNLFDMTQAITSEGGQYVTLDAPLHVVNVHLHQNTILPMQQGGMISKEARMTPFALVVTFPAGASDGKAAGKLFLDDDELPEMKLASGSATYVDFYATLSQGTVKLWSEVQESKFALDKGWKISKVAVLGLGRSGAPSALEFDGKPVTAASNIELTSLEQKYLEDLQVGSEKKSSVMVEVNGLEIPVGKNFAMSWKMGISG

>Pt_Gluc_7 (XP_002298949.1)

MEKERNKTGSRNPKAISQSNSNLLLIFFLLVHWVPLISGKEVKEEVVGYGYKVGSVNSGFTGKSLTADLSLIKESSVYGDDIQHLSLVASFETKNRLRVRITDSKNQRWEIPEDIVPREGHSPENYLHYSPLKHRVLLENNLLSDPNSDLLFTLHNTTPFGFTITRKSSGDVLFDTSPDTSNPDTFLVFKDQYIQLSSRLPIKRSSLYGLGEHTKSTFKLKPKDAFTLWNADLGSANIDVNLYGSHPFYIDVRSASADDKVKAGTTHGVLLFNSNGMDIVYGGDRITYKVIGGIIDLYFFAGPLPDMVIEQYTELIGRPAPMPYWSFGFHQCRYGYKNISDVEGVVAGYAKAGIPLEVMWTDIDYMDAYKDFTFHPTNFPLEKMKKFVNTLHQNGQQYVLILDPGISVNSSYETYIRGMQADIFIKRNGIPYLGEVWPGKVYFPDFVNPAGLEFWGNEIKMFRELLPVDGLWIDMNEISNFIDPTPTPSSTLDNPPYMINNAGVRRPINNKTIPATSLHFDIMTEYNVHNLYGLLESKATNAGLINSTGKRPFVLSRSTFVGSGRYTAHWTGDDAATWDDLAYTIPSILNFGLFGIPMVGADICGFSGNTTEELCRRWIQLGAFYPFARDHSSIDTTRQELYLWDSVAATARKVLGLRYQLLPYFYTLMYEAHTKGTPIARPLFFSFPRDTKTYEVNSQFLIGKGVMVSPVLKSGATSVDAYFPAGNWFDLFNYSNSVSVSSGKYINLAAPADHINVHVHEGNILALQQEAMTTKEARKTAFHLLVVLSSTGNSTGESFLDDGESVDMGGVGKNWSLVKFSGGIVGNRVVVGSNVINGEFAVSQKWIIEKVTFLGLEKTKGQFDVLEISGLSQPLGQEFNLEKTF

>Pt_Gluc_8 (XP_006368273.1)

METVLVGSSGQQRGLTPTISKLRHHHKLPPPHSLFPTSTTTCTSAVTLASRKRRLNKKLSCRGLMSKMADHDQAKVVAADVVSGDMIFQPILEDGIFRFDCSAEARAASYPSLSFIRSSDRDTPIMSHSVPSYTPTYECVSGKQIVKFEFPDGTTFYGTGEVSGQLERTGKRVFTWNTDAWGYGPGTTSLYQSHPWVLAVLPNGEALGVLADTTLRCEIDLRKESIIQFIAPSSYPVVTFGLFASPTDVLKSLSHAIVVYAVSLSSGTVFMPPKWSLGYQQCRWSYDSDERVREIARTFREKGIPCDVIWMDIDYMDGFRCFTFDQAYPQSLVKDLHDDGFKAIWMLDPGIKKEEGYLIYDSGSENDAWIKKADGEPFVGEVWPGPCVFPDFTQSKVRAWWALLVKDFTSNGVDGIWNDMNEPAVFKTVTKTMPESNLHLGDEEIGGCQNHSHYHNVYGMLMARSTYEGIKLANENKRPFVLTRAGFIGSQRYAATWTGDNLSNWEHVHMSISMVLQLGLSGQPLSGPDIGGFAGNATPKLFGRWMGVGAMFPFCRGHSEKSTNDHEPWSFGEECEEVCRLALKRRYRLLPHIYTLFYLAHTTGIPVATPTFFADPKDPGLRTTENSFLLGPLLVFSSTIADQGMDRLHPVLPKGIWLRFDFDDSHPDLPTLYLQGGSIIPLAPPHQHVGEANLSDDLTLLVALDQNGHAEGLLFEDEGDGYEFTRGGYLLTRYVAELQSSAVTVRVSQMEGSWKRPRRRLRVQLLLGGGAMLDSWGIDGDVLKINMPTEVEVSTLVSTSEKQYRTRLGIECAKHIPELEEVSGPKGVVDLSKVPVELKNGDWIAKVVPWIGGRIISMEHLPSGTQWLHSRVEIDGYEEYSGTEYRSAGCSEEYSVIERDLEHAEEEESLILEGNIGGGLVLRRQISILKDNPKILQIDSGIIARSVGAGSGGFSRLVCLRVHPAFTLLHPTETFVSFTSIDGSKHEIWPESGDQFYQENLLPNGEWMLVDQCQGLALVNRFNINEVFKCYIHWGTGTVNLELWSEDRPVSKQSPLTVSHGYEVRGIS

>Sl_Gluc_1 (XP_004235201.1)

MTSLKISKKHHKHFNNPFPSTPNSPFIYGTLILNSHKLPSHQIYPIGKDFQLNWSSKNGGFLSISHKSEPTRPIWSTLPGEPFISAAIAETQVEESRGSFVVKDKHVHSLSSNQTIDDVKIINESDKDQLFSSYPLFPVLMITGKVFGVSKRKKKVGFSRRKDSEKENSTCARYWILFDQKECHQVGFQVRIGKTDLQLPKRVSPTSYRIFSLKFGRIRRRRGGWFGGLKKSVTVSSFAEEKIVMKNSEGVVNNRICLTYSSEKNEKIFGFGEQFSHMNFKGKRVPIFVQEQGIGRGDQPITFAANLVSYRAGGDWSTTYAPSPFYMTSKMRSMYLEGYDYSVFDLTKDDRIQIQLHGDSLEGRILHGNSPTELIECFTRSIGRPPLLPEWIISGAVVGMQGGTDTVRSIWNEMQRYDVPVSAFWLQDWVGQRETVIGSQLWWNWEADETRYSGWKQLIQDLNKQHIKVMTYCNPCLAPMDKKTNIRRHHFEEAKKLDILVKDKNGELYMVPNTAFDVGMLDLTHPRTANWFKQILREMVDDGVRGWMADFGEGLPVDACLYSGEDPIAAHNRYPELWAKINREFVDEWKNTHVGKEGEDPEDSLVFFMRAGYRDTPKWAMLFWEGDQMVSWQKNDGIKSAVVGLLSGGLSGYALNHSDIGGYCAVNLPLPFFKYQRSEELLLRWMELAAFTTVFRTHEGNKPSCNSQFYSNNRTLSHFARLAKVYKAWKFYRIQLVKEASQKGLPICRHLFLHYPEDEDVHSLTYEQFLVGTEILVVPVLDKGKETVKAYFPIGERLSWKHIWTGKLYSTHGSEAWVEAPIGYPAIFVKEGSSVGKTFLEKLREYNVL

>Sl_Gluc_2 (XP_004236811.1)

MNNHTTNKSCIIIIILSKYLILFFIFLVPFLALITKSEQVGYGYNVRSIGVDSSGRTLTAHLQLIKNSSVFGIDIQNLTLTACFEAKERLRVRITDADHERWEVPREFIPRETHLPPRSSLLEKRSSTSFPLSEETHYFHTDTVSDLAFTLYNTTPFGFTITRHSTGDVLFDTRPENDSPDTFFIFKDQYLQLSSSLPANRSSIYGLGEHTKRNFKLKHNQTLTLWNADIASANADLNLYGSHPFYMDVRSHPGGGTSHGVLLFNSNGMDIVYAGDRITYKVIGGVVDLYFFAGPVPELVMEQYTELIGRPAPMPYWSFGFHQCRYGYKDITEIKNVVAGYAKAQIPLEVMWTDIDHMDGYKDFTLDPINFPLDQMKKFVDTLHQNGQKFVLILDPGISINSSYETYKRGMQADVFIKRNGVPYLGEVWPGKVYFPDFINPRGRVFWSNEIKIFHDLLPIDGLWLDMNELSNFISSPPSPSSTLDNPPYKINNSGGLRPINEKTVPATSVHFGNTLEYDVHNLYGFLEAKTTNAALIDVTGKRPFILSRSTFVGSGKYTAHWTGDNAATWDDLAYSIPSVLSSGLFGIPMVGADICGFGRNTTEELCRRWIQLGAFYPFARDHSEKFTIHQELYIWDSVAATARKVLGLRYRLLPYFYTLMFEAHTKGIPIARPLFFSFPEDANTYTIDSQFLIGKGLMISPVLTSGAVSVNAYFPSGTWFNLFNYSNYVNMKSGSYISLDAPPDHINVHLREGNIVVMQGEAMTTRAARDTPFELVVAINNWGNSSGEVFLDDGEDVEMGGEGGKWSLVKFHTNVVNKKLYLRSNVVNEEFALSKNWRIHKVTFLGLKKGVSKINAYNLTTKIRTKIDKSAFGVLEMGGLSVLIGKEFTIELTL

>Sl_Gluc_3 (XP_004233180.1)

MKGIQFPSLNRNTFIIFFLIHLSIFSPYFAISIKQKEAQQQVIGYGYSIKSVAITGNSIFANLQLISTSSVFGTDIHNLILIASLETNDRLRIRITDVNHQRWEVPEEILRRPPPPSPPSTSNSSSENHFPITLSNPNSDLEFTLHNTTPFSFTVRRRFTGDTLFDTSPENENPDTFLIFKDQYIQISSALPTTRANLYGLGEHTKSSFKLTHNQTLTLWNADIGSSNADLNLYGSHPFYMDVRSSDPAKETAAGVSHGVLLLSSNGMDIVYTGDRIIYKVIEGLIDLYFFAGPSPEMVVDQYTQLIGRPAAMPYWSFGFHQCRWGYKNIDDVELVVESYAKARIPLEVMWTDIDYMDGFKDFTLDPVNFPLERVNFFLRKLHQNDQKYVLIVDPGISINNTYDTYRRGMEADVFIKRDNMPHQGVVWPGNVYYPDFLNPATEVFWRNEIEKFQDLVPFDGLWLDMNELSNFITSPPTPSSTFDDPPYKINNSGDHLPINYRTVPATSTHFGNTIDYNVHNLYGLLESRATYSALVNVTGKRPFILARSTFLGSGRYTSHWTGDNAATWNDLAYSIPTILNFGLFGIPMVGADICGFSSNTTEELCRRWIQLGAFYPFARDHSAKDTTPQELYSWDSVAAAAKKVLGLRYQLLPYFYMLMYKAHTKGTPIARPLFFSFPQDAKTFDISTQFLLGKGVMISPVLKQGATSVDAYFPAGNWFDLFNYSRFVSLNQGTYLTLDAPPDHINVHVREGNILVMQGEAMTTQAAQRTAFKLLVVLSSSENSTGELFVDDDDEVQMGREGGRWTFVKFNSNIIGNKIVVKSEVVNGRYALDQGLVLEKMTLLGLENVRGLKSYELVGSHQQGNTTMKEKLKQSGQFVAMEISEMSILIGKEFKLELYIIT

>Sl_Gluc_4 (XP_004236961.1)

MFFSSFLSPYSLTSLLILILCIVGVDFVSTTQIKIGQGYKLISIEKSQDGGLIGHLQVKEKNNIYGLDIPNLKLYVKHETDNRLRVHITDAKEQRWEVPYNLLQRETPPSLKQNIGGKSRKNIFSSLAVESEYSGNELIFNYKNDPFSFSVKRKSNGEILFNSSFDDSNSYNNLVFKDQYLEISTKLPKNASLYGLGENTQPHGIKIYPNDPYTLYTTDQPAINLNMDLYGSHPMYMDLRNNVNGEAKAHVVLLLNSNGMDVFYNGDSLTYKVIGGVLDFYFFSGPTPLDVVDQYTSFIGRPAPMPYWSFGFHQCRWGYHNLSVIEDVVENYNKAKIPLDVIWNDDDHMDGKKDFTLNPVNYPRPNLLAFLDKIHGRGMKYIVIVDPGIGVNNTYGVYQRGIANDVFIKYEGKPYLAQVWPGAVNFPDFLNPKTVEWWGDEIRRFRELVPIDGLWIDMNEVSNFCSGLCTIPQGRICPTGTGPGWICCLDCKNITNTRWDDPPYKINASGIQAPIGYKTIATSAVHYNGVKEYDAHSLYGFSQSIATHKALQGLEGKRPFILSRSTFVGSGHYAAHWTGDNKGTWDDLRYSISTVLNFGLFGVPMVGADICGFYPAPTEELCNRWIEVGAFYPFSRDHANYYSPRQELYQWESVAESARNALGMRYKLLPYFYTLNYEAHTTGAPIARPLFFSFPTLTELHDVSTQFLVGTSVMVSPVLDQGKTEVKVLFPPGTWYNLFDMTQAIVTKDLHYLTLDAPLSVVNVHVYENTIIPMQRGGMISKEARTTPFTLVVTLPSGTNDIEAKGNLFLDDDELPEMKLGNGQSTYIDFHATASNGTMKLWSEVQESKFALDKGWFIEKVIVLGTNGTDGAFEINVDGQPIEDGSRVKYSTTEHKYIDSMDDEVDKRKSMMMEIHGLQLPLGKNFVMSWKM

>Sl_Gluc_5 (XP_004238937.1)

MKFSFSFPFLLVLTICIIGCVKLVHTAPTKIGNGYSLIAIEESPDGGLIGYLKVKKKNKIYGPDIPNLQLYVKHETDNRLRIHITDADKQRWEVPYNLLPRESPPSLKQTIGKSRKGQLPLLSNQKYSGNELMFSYTSDPFSFSVKRKSNGQTIFNSSSEDSDPYSNLVFKDQYLEISTKLPKDASLYGLGENTQPHGIKIYPNDPYTLYTTDQSSINLNMDLYGSHPMYMDLRNVNGEAYAHAVLLMNSNGMDVFYRGDSLTYKVIGGVLDFYFFSGPTPLAVVDQYTEFIGRPAPMPYWSFGFHQCRWGYHNLSVIEDVIANYKKAKIPLDVIWNDDDHMDGKKDFTLHPVNYPGPKLRAFLKKIHAEGMHYIVINDPGIGVNKSYGTYQRGLANDVFIKYQGKPFLAQVWPGAVHFPDFLNPKTVEWWGDEIRRFHELAPIDGLWIDMNEVSNFCNGLCTIPEGRICPNGTGPGWICCLDCKNVTKTKWDDPPYKINASGIQAPIGYKTIATSATHYNGVREYDAHSLYGFSETIATHKGLQAIEGKRPFILTRATFVGSGHYAAHWTGDNKGTWEDLKYSISTVLNFGMFGVPMVGSDICGFYPAAPPLEELCNRWIQVGAFYPFSRDHANYYSPRQELYQWKSVTKSSRNALGMRYKLLPYLYTLSYEAHITGAPIVRPLFFTFPNIPELYELSTQFLVGSNVMVSPVLEKAKTKVNALFPPGTWYSLFDMTQVIVTKEPHYRSLDAPLHVVNVHLYQNTILPMQRGGMLTKEARMTPFTIVVAFPLGASEGVAKGNLFLDDDELPEMKLGNGKSTYMDFHATTSNGTVKIWSEVQESKYALDKGWYIEKVTVLGLNGIGGAFDILVDGSKVEDTSKLEFETEEHKFVDKLEDGGHKKSMMLDIKGLELPIGKNFAMSWKMGI

>Sl_Gluc_6 (NP_001234030.1)

MRAPLLLYPLLLLLLLATSAYSWKKEEFRNCDQTPFCKRARSRKPGSCNLRVVDVSISDGDLIAKLVPKEESEQPNKPLVLTLSVYQDGVMRVKIDEDQNLNPPKKRFEVPEVIEEDFLNTKLWLTRVKEEQIDGGSSSSSGFYLSDGYEGVLRHDPFEVFARESGSGKRVLSINSNGLFAFEQLREKKEGDDWEEKFRSHTDTRPYGPQSISFDVSFYGADFVYGIPERATSFALKPTKGPNVEEYSEPYRLFNLDVFEYLHESPFGLYGSIPFMISHGKARGSSGFFWLNAAEMQIDVLGSGWNSDESSKIMLPSDKHRIDTLWMSESGVVDTFFFIGPGPKDVVRQYTSVTGRPSMPQLFATAYHQCRWNYRDEEDVYNVDSKFDEHDIPYDVLWLDIEHTDGKKYFTWDRVLFPNPEEMQKKLAAKGRHMVTIVDPHIKRDESYHIPKEALEKGYYVKDATGKDYDGWCWPGSSSYTDLLNPEIRSWWSDKFSLDSYVGSTKYLYIWNDMNEPSVFNGPEVTMPRDALHHGGVEHRELHNSYGYYFHMATSDGLLKRGDGKDRPFVLARAFFAGSQRYGAIWTGDNTAEWEHLRVSVPMVLTLSISGIVFSGADVGGFFGNPDAELLVRWYQLGAYYPFFRGHAHHDTKRREPWLFGERNTQLMREAIHVRYMYLPYFYTLFREANSSGTPVARPLWMEFPGDEKSFSNDEAFMVGNGLLVQGVYTEKAKYVSVYLPGEESWYDLRSASVYKAGHTHKYEVSQDSIPSFQRAGTIIPRKDRLRRSSTQMENDPYTLVIALNSSKAAEGELYIDDGKSYEFNKVPSFIGVSHSQMGSLYLQMQPRLQLAVTHFPSECTVERIILLGLSPGAKAAIIEPGNKKVEIELGPLFIQGNRGSVPTIRKPNVRIADDWSIQIL

>Sl_Gluc_7 (XP_004246000.1)

MDKLPFPNSIIISPKCHFTTLCFVNVRVSNGSLKKKKPKLLNCANLLSSTSVSSIHRLIRGRSVNKGFIGASFVMLKMGGIEGTTAMSDARTGNMIFESILEEGVFRFDCSADDRNAAFPSISFVDPKVRETPLMSIHKVPSYIPTFECVRGQQIVNIELPSGTSFYGTGEVSGQLERTGKRILTWNTDAWGYGPGTTSLYQSHPWVLAVLPSGETLGVLADTTHRCEVDLRQESNIRFISRQSFPVITFGPFPSPIDVLVSLSHAIGTVFMPPKWSLGYHQCRWSYVPDTRVREIARTFREKKIPCDVIWMDIDYMNGFRCFTFDKERFPDPESLVEELHKSGFKAIWMLDPGIKNEKGYFAYDSGSEADVWVQTADGRPYVGDVWPGPCVFPDFTQLKARSWWANLVKDFISNGVDGIWNDMNEPAVFKTVTKTMPENNIHRGDPEFGGCQNHSYYHNVYGMLMARSTYEGMKLANGNKRPFVLTRAGFVGSQRYAATWTGDNLSTWEHLQMSIPMVLQLGLSGQPLTGPDIGGFAGNATPRMFGRWMGVGSLFPFCRAHSEADTNDHEPWSFGEECEEVCRLALERRYRLLPHIYTLFYLAHTRGTPVSAPIFFADPKDPELRKLENSFLLGPILIYASTQRDEELDTAHHKLPRGIWLSFDFDDSHPDLPALYLLGGSIIPVGPLYQHVGQADPSDDLTLLIALDENGKAEGLLFEDDGDGYEYSQGGYLLTTYVAELQSSVVTVQVAKTEGNWRRPKRRLHVRILLGKGAMLDAWGSDGEIIQLAMPSETDVSNLVSESEEKYRNRLEGAKRIPDVETISGHKGVELSRTPVVLKSGDWELKAVPWIGGRILSMDHVPSGTQWLHSRVEINGYEEYSNREYRSAGCTEEYSVIERDLEQEGESESLRLEGDIGGGLFMERYISLPKDNSKVFRIDSGIVARGVGAGSGGFSRLVCLRVHPMFTLLHPTESYVSFTSLNGSKHELWPESGEQVFEGDLRPKGEWMLVDRCLGLGLVNRFNIDQVHKCMVHWGTGTVNLELWSEERPVSKDSPLKISHEYEVQKIA

>Mt_Gluc_1 (XP_003624991.1)

MAILKITKKHHKRFNNPFPSAPTTIPNVQGSLFINSKALSSQDQTFSIGNDFQLSWSTLNGGQFSISHLSQKTRPIWSTISGKAFVSAAVVDAEIEESRGSFLVKDKDVHLTCNHQTIDDIRIINEFGDHLEYEVEDLDQKCSAEETKFPPTLLITGRLFNMSKKKKRFQKYGIQGNIQFEPKGPFVYARYWVLFNQKNKHEIGFQVKIEKLNFSLSNKVVSPEASEIYKGFKKRLSSRKKKIGWCWYLSRPRGFVLVSSVEDESGVMEIPKPKEFNRVWLTYASDENERFYGFGEQFSHMNFKGKRVPILVQEQGIGRGDQPITLAANLVSYRAGGDWSTTYAPSPFYMTSKMRSLYLEGYDYTIFDLTKLDRVQIQIYGNSIEGRILHGNNPCDLIKHFTKTIGRLPELPEWIISGAIVGMQGGTDAVRRVWDELRTYDVPVSGFWLQDWVGQRETMIGSQLWWNWEVDEQRYWGWKELIKDLSTQNIKVMTYCNPCLAPVDEKNNKKRNLFEEAKQLDILVKDNNGNAYMVPNTAFDVGMLDLTHPKTATWFKQILLEMVDDGVRGWMADFGEGLPVDAVLYSGEDPISAHNRYPELWAKINREIVEEWKSKSLDNLKEEQEDGLVFFMRAGFRDSPKWGMLFWEGDQMVSWQANDGIKSSVVGLLSSGISGYAFNHSDIGGYCTVNLPIVKYRRSQELLLRWMELNSFTTVFRTHEGNKPSCNSQFYSNQQTLSHFARTAKIYTAWKFYRIQLVKEAAQKGLPVCRHLFLHYPNDEHVHNLSYQQFLVGSEFLVVPVLDKGMKKVKAYFPLGESSSWLHIWTGNVFSKQGSESWIEAPIGYPAVFIKFGSIIGETFLNNLKNLGILQ

>Mt_Gluc_2 (XP_003602674.1)

MTNQTLRFTLLLLLCTTVFSWKKDEFRNCNQTPFCKRARSRSPGSSDLIATHVTISDGDLTANLIPKSQPDSSKPLLLTLSVHQDGILRLIIDENEHSSSKKRFHVPDVVVSQFANTKLWLPRINSEDLNGPSSSVYLSDGYSAVIRHDPFELFIRDDNSGDRVISINSHGLFDFEQLREKNEDENWEESFRTHTDKRPYGPQSISFDVSFYDADFVYGIPERATSLALKPTRGPNVEESEPYRLFNLDVFEYIHDSPFGLYGSIPFMLSHGKGRGTNGFFWLNAAEMQIDVLASGWDAESGISLPTSQNRIDTMWMSEAGVVDAFFFVGPRPKDVLRQYAAVTGGSALPQMFAVAYHQCRWNYRDEEDVKNVDAKFDEYDIPYDVLWLDIEHTDGKRYFTWDRVLFPNPEEMQKKLDGKGRRMVTIVDPHIKRDENFHLHKEASEKGYYTKDSSGNDFDGWCWPGSSSYPDTLNPEIRSWWADKFSYQSYVGSTPSLYIWNDMNEPSVFNGPEVTMPRDALHYGGVEHRELHNAYGYYFHMATSEGLLKRGEGKDRPFVLSRALFAGSQRYGAIWTGDNSADWDHLRVSVPMVLTLGLTGMSFSGADVGGFFGNPDPELLVRWYQLGAYYPFFRAHAHHDTKRREPWLFGERKTELIRDAIHVRYALLPYYYTLFREANTTGVPVARPLWMEFPSDEATFSNDEAFMVGSSILVQGIYTERAKHASVYLPGKQSWYDLRTGTVYKGGVTHKLDVTEESIPAFQRAGTILTRKDRFRRSSSQMTNDPFTLVVALNSSQAAEGELYIDDGSSFGFLEGAFIHRRFIFANGKLSSVDLAPTSGGNVRHTSDVVIERIIVLGHAHGSKNALIETSNQKVDIELGPLWVQRAHSPAFMTIRKPNVRVAEDWTIKIL

>Mt_Gluc_3 (XP_003614705.1)

MVYPHSLAFLCFSSLLLTLLFSSEVSSSSKGATKIGQGYRLVSIEETPDGALVGLLQLNQKTKIYGPDIPLLRFYAKHETENRLRVHITDANKQRWEVPYNLIPREQPPPLPQTLGKFQKNPIEVSEYSGSELLFSYISNPFSFSVKRKSNGETLFNSTSTSSDPFSSLVFKDQYLEISTKLPKDASLYGLGENTQPHGIKLYPSDPYTLYTTDISAINLNADLYGSHPMYMDLRNNGGKASAHAVLLLNSNGMDVFYKGTSLTYKVIGGVFDFYFFSGPTPLNVVDQYTTLIGRPAAMPYWAFGFHQCRWGYHNLSVVEDVVDSYKKAQIPLDVIWNDDDHMDGHKDFTLNPKNYPRPKLLNFLNKIHSIGMKYIVIIDPGIGVNSSYGVYQRGLANDVFIKYEGEPFLAQVWPGAVNFPDFLNPKTVNWWVDEIRRFHELVPVDGLWIDMNEASNFCSGKCKIPKNTICPNGTGPGWICCLDCKNITKTRWDDPPYKINASGIQAPIGYKTIATSATHYNGVLEYDAHSIYGFSQSVATHKGLLGLEGKRPFILSRSTYVGSGKYAAHWTGDNQGTWENLRYSISTMLNFGIFGVPMVGSDICGFYPQPTEELCNRWIEVGAFYPFSRDHANYYSPRQELYQWDSVAQSARNALGIRYKILPYLYTLNYEAHVSGSPIARPLFFTFPSYTKCYDVSTQFLLGSSLMISPVLEQGKTQVKALFPPGSWYSLLDWTHTITSTGGTYVTLDAPLHVVNVHLYQNTILPMQQGGLISKDARKTPFTLIVTFPAGASEGDAKGTLFIDDDELPEIKLGNGYSSFIDLYASVKQGGVKVWSEVQEGKFALDKGLIIDSISVLGLDGNVGAVASLELDGKPLIGMSSLNVTTSEHVHLEGEGNGESKTVMVTLRGLSIPVGKNFAMTWKMG

>Mt_Gluc_4 (XP_003637439.1)

MDGHKDFTVNPVNYPLPKLLNFLDRIHSIGMKYIVINDPGIAVNTKYGVYQRGMANDVFIKYEGEPFMAMVWPGAVYFPDFLNPKTVSWWADEIRRFHELVPIDGLWIDMNEVSNFCTGKCTIPKERFCPLQGEKLPNSTCCLDCTNITSTRWDDPPYKINASGNEIPIGYKTIATSAVHYNGVLEYDAHSLFGFSEAIATHKALSELQGKRPFVLSRSTYVGSGKYAAHWTGDNKGTWEDLRYTISTILNFGIFGMPMVGADICGFYPSFYPTLRYPISTEELCNRWIEVGAFYPFSRDHSSVISPRQELYQWESVAESARNALGMRYKLLPYLYTLNYEAHISGAPIARPLFFSFPTYIECYSLSTQFLLGSSLMISPVLEQGKTEVDALFPPGTWYSLFDLTQVIVSKDGTNVTLNAPLHVVNVHLYQNSILPMQQGGMISKDARMTPFSLIVTFPAGANEGEAKGNLFLDDDELPEMKLGNGYSTYIDFHASVKEGTVKVWSQVQEGKFALDKGWVIDTINVLGLNGNGAIGTIEINGKPTNVKIDTTKQNYIHGRGDGEKNIVMVGMKGLNIPVGKSFAMTWKMGS

>Mt_Gluc_5 (XP_003612579.1)

MKEALICSSYSGSYKITTKPTAIPFSPPSISLFRHRRLHRNTPFLSSIITLRRKRFIEKLISKMANYEGQVSSGSTDVRTGKMIFEPILNDGVFRFDCSLNDRDAAYPSISFVNSKDRETPITGTHKVPSYTPTFECLLEQQVVQLELPVGTSLYGTGEVSGQLERTGKRVFTWNTDAWGYGPGTSSLYQSHPWVLAVLPNGEALGILADTTRRCEIDLRKESTIRFIAPSSYPVITFGPFASPTEVLISLSKAIGTVFMPPKWSLGYQQCRWSYLSDQRVLEVAKTFREKSIPCDVIWMDIDYMDGFRCFTFDKERFRDPKSLVESLHYSGFKGIWMLDPGIKQEKGYFVYDSGSENDVWVQKADGTAFVGDVWPGPCVFPDYTQSKVRAWWANLVKDFVSNGVDGIWNDMNEPAVFKAVTKTMPESNVHRGDGELGGCQNHSFYHNVYGLLMARSTYEGMKLANENRRPFVLTRAGFSGSQRYAATWTGDNLSTWEHLHMSISMVLQLGLSGQPLSGPDIGGFAGNATPRLFGRWMGVGSLFPFCRGHSEAGTTDHEPWSFGEECEEVCRLALKRRYRLIPLIYTLFYFAHTKGIPVATPTFFADPTDPSLRKLENSFLLGPVLVYASTTRNQGLDKLEVTLPKGIWLGFDFGDAHPDLPALYLKGGSIIPAGLPLQHVGEANPSDELTLLVALDESGKAEGFLFEDDGDGYEFTRGNYLLTHYSAQLQSTAVTVSVHRTEGSWKRPKRRLHIQLLLGGGAMLDTWGVDGEVLHVNLPSEEEVSKLVSTSEKQYKERLEKAIQIPDVEDEVSGPKGMELSRTPIELKSSDWLLKVVPWIGGRIISMIHFPSGTQWLHGRIEISGYEEYSGTEYRSAGCSEEYSIINRELGHAGEEESVLLEGDIGGGLVLQRQICFPKNAANIIQINSSIIARNVGAGSGGFSRLVCLRIHPTFNLLHPSESFVSFTSINGSMHEVFPDGGEQIFEGHLIPDGEWKLVDKCLGLALVNRFNVTEVSKCLVHWDFGTVNLELWSESRPVSEQSPIQISHQYEVIRIP

**Receptor-like kinases**

>Zea_mays_ZmPK1 (NP_001105424.1)

MPRPLAALLSTACILSFFIALFPRAASSRDILPLGSSLVVESYESSTLQSSDGTFSSGFYEVYTHAFTFSVWYSKTEAAAANNKTIVWSANPDRPVHARRSALTLQKDGNMVLTDYDGAAVWRADGNNFTGVQRARLLDTGNLVIEDSGGNTVWQSFDSPTDTFLPTQLITAATRLVPTTQSRSPGNYIFRFSDLSVLSLIYHVPQVSDIYWPDPDQNLYQDGRNQYNSTRLGMLTDSGVLASSDFADGQALVASDVGPGVKRRLTLDPDGNLRLYSMNDSDGSWSVSMVAMTQPCNIHGLCGPNGICHYSPTPTCSCPPGYATRNPGNWTEGCMAIVNTTCDRYDKRSMRFVRLPNTDFWGSDQQHLLSVSLRTCRDICISDCTCKGFQYQEGTGSCYPKAYLFSGRTYPTSDVRTIYLKLPTGVSVSNALIPRSDVFDSVPRRLDCDRMNKSIREPFPDVHKTGGGESKWFYFYGFIAAFFVVEVSFISFAWFFVLKRELRPSELWASEKGYKAMTSNFRRYSYRELVKATRKFKVELGRGESGTVYKGVLEDDRHVAVKKLENVRQGKEVFQAELSVIGRINHMNLVRIWGFCSEGSHRLLVSEYVENGSLANILFSEGGNILLDWEGRFNIALGVAKGLAYLHHECLEWVIHCDVKPENILLDQAFEPKITDFGLVKLLNRGGSTQNVSHVRGTLGYIAPEWVSSLPITAKVDVYSYGVVLLELLTGTRVSELVGGTDEVHSMLRKLVRMLSAKLEGEEQSWIDGYLDSKLNRPVNYVQARTLIKLAVSCLEEDRSKRPTMEHAVQTLLSADD

>Medicago_truncatula-238 (XP_003622937.1)

MDFSFTLFLLVLAFSFKSSSSFLSSLRKGSSISVENPQDQIISQNGMFSAGFTSIGENSYSFAIWFTEPTSLDLNKTIIWMANRDQPVNGKRTKLSLLNTGNIVLLDVSLNNVWSSNTASLKPLELHLKNDGNLVLRELQGINILWQSFDSPTDTLLPGQPLTRYTKLVSSISESNHSSGFYMFFFDDENILGLHYDGRDVSSSYWPSPWLLSWDVGRSNFNSSRNAVLDSFGSFHSSDNFTFSTSDYGTVLQRMMKLDSDGVVRVYSRTNVSQNWYVSWQAFTGTCLVHGICGANSTCSYSPKIGRKCSCIPGYRMNNPNDWSYGCEPMFDFTCNKSESTFLEIKNVEFYGYDFHYIEICNYSACLDLCIQDCNCKAFQHSYWEKKGLYRCFTKTQLQNGRFYPSFKGSTYLRLPKGSTFSKRESSDPSDDICSEKLQRVYVKESENHFVKFFLWFATAIGALETVFIFSVWCSLFRSRQKTYADQHGYHLAELGFRKYSYLELKKATKGFSQEIGRGGGGVVYKGILSDGRHAAIKRLYNAQQGEGEFLAEVGIIGRLNHMNLIEMWGYCAEGKYRLLVYEYMENGSLAENLSANKLDWSKRYKIALSIGRVLAYLHEECLEWILHCDIKPQNILLDSNFEPKLADFGLSKLQNRNNLNNSSVSMIRGTRGYMAPEWIFNLPITSKVDVYSYGIVVLEMITGKSPTTGFKIVNGEEESDGRLVTWVREKRGGNISWLEEIVDSQIGLNYDKSKMEIMAKVALDCVVDDRDSRPTMSRVVEMLQYHGSDI

>Cicer_arietinum-238 (XP_004492313.1)

MNTLDFSFTLFLLVLALLFQTSPSSSSSLRKGSSLSVENPQDTIISPNGMFSAGFIAIGENAYSFAIWFTTPNSINHNNTIVWTANRDEPVNGKRSKLTLLNTGNIVLFDVSLNNVWSSNTESLEPLELHLNDDGNLVLRELQGTRILWQSFDSPTDTLLPGQSLTRYTILVASRSESNHSSGFYKFFFDDSNVLGLHYDGPDVSSSYWPKPWLLSIQVGRANFNGSRIAMLDSFGVFYSSDNFTFMTSDYGTVLQRRMKLDCDGNVRVYSRINVSQDWYVSWQAISGACIAHGICGDNSTCSYSPKHGRKCSCLPGYKVKNSSDWSNGCEPMFDFTCNKSESTFLEMKNVEFYGYDIHYVQISNYSACESLCLQDCNCKGFQHSFVELKGFYKCYTKTQLRNGRLSPFFVGTSYLRLPKGNSFFEQESSNPSDHVSSVKLQRVFVRVRENHFVKIFLWFATAIGALEMVCIFVVWCCLMRTHQKTNADQHDYHLAALGFRKFSYLELKKATKGFSEEIGRGGGGVVYKGLLSDQRHAAIKKLHNHNAKQGEGEFLAEIAIIGRLNHMNLIEMWGYCAEGKHRLLVYEYMENGSLAKNLSSNTLDWSKRYKIAFAIARVLAYLHEECLEWILHCDIKPQNILLDSNLHPKLADFGLSKLKNRNTINTNSSFSMIRGTRGYMAPEWIFNFPITSKVDVYSYGIVVLEMITGKSPTIGIKNFNEEEVCDGRLVTWVRDKMNSLSWLEEIVDPQIGSNYDKIKMEVLTKVALDCVLEDRDSRPTMSKVVEMLQNYA

>Phaseolus_vulgaris-238 (XP_007140583.1)

MTLLATFFLLASLLSFQPSRSSSSLRMGSSLSVENPQHILISPNAIFSAGFLAIGHNAYSFAIWFTEPHFHSPNTVTWMANRDQPVNGKRSKLSLTHDGNILLIDASLNTAWSSNTDSLDPVELHLKDDGNLVLRKLQGTVLWQSFDFPTDTLVPGQPLTRYTQLVSSRSHTNHSSGFYKLFFGDDNILRLVYDGPDVSSNYWPSPWRVSWDVGRTLFNSSRIAVLNSFGVFHSSDNFTSKTSDYGTVLQRRLKLDSDGNFRVYSRNSVSEKWYVSWQAITNGCLIHGVCGANSTCGYDGKSGKKCKCLPGYRLNNQSDWSHGCKPMFDLTCNRNESTFLEMRGVEFYGYDNYYIQFSNFSACEDLCLQNCSCQGFQYSYNDKKGITFKCYTKTQLLNGRRSPRFPGATYLRVSKSYSFSGEESANDTAHDHRVCSVQLQRDYIKTSGNRIVSVFMWFATALGAFEMVCVSVIWCFLIRTSQKSDADQQAYHLTATGFRKFSYSELKKATKGFSEEIGRGGGGVVYKGILSDQRHAAIKRLYNAKQGEGEFLAEVGIIGKLNHMNLIEMWGYCAEGKHRLLVYEYMENGSLAENLSTNKLDWSKKYNIAIGTARVLAYLHEECLEWILHCDIKPQNILLDSNYEPKLADFGLSKLLNRNNPNNPSISMIRGTRGYMAPEWVLNLPITSKVDVYSYGIVVLEMVTGRSTASVQEINGEETYDGRLVAWVREKRSNTNSYWVEQIIEPSIGPDYDRSKMEMLTTVALNCVNEDRDSRPSMSQVVEMLQGHGSDPYS

>Glycine_max-238 (XP_003530873.1)

MALSITLFLLASLLSFQISSSSSSLRKGSSLSVENPQHVLVSPNGMFSAGFLAIGENAYSFAIWFTEPHFHSPNTVTWMANRDQPVNGKGSKLSLTHAGNIVLVDAGFNTAWSSNTASLAPAELHLKDDGNLVLRELQGTILWQSFDFPTDTLVPGQPLTRHTLLVSARSESNHSSGFYKFFFSDDNILRLVYDGPDVSSNYWPNPWQVSWHIGRTLFNSSRIAALNSLGRFRSSDNFTFVTFDYGMVLQRRLKLDSDGNLRVYGRKSAVEKWYVSWKAIRNDCIIHGVCGPNSTCGYDPKSGRTCKCLPGYRLRNHSDWSYGCEPMFDLTCNWNETTFLEMRGVEFYGYDNYYVEVSNYSACENLCLQNCTCQGFQHSYSLRDGLYYRCYTKTKFLNGQRLPRFPGTTYLRIPKSYSLSVKESAIDSVDDHHVCSVQLQRAYIKTLESRVVRVLLWFAAALGAFEMVCIFVVWCFLIRTGQKSNADQQGYHLAATGFRKFSYSELKKATKGFSQEIGRGAGGVVYKGILSDQRHAAIKRLNEAKQGEGEFLAEVSIIGRLNHMNLIEMWGYCAEGKHRLLVYEYMENGSLAQNLSSNTLDWSKRYNIVLGTARVLAYLHEECLEWILHCDIKPQNILLDSNYQPRLADFGLSKLLNRNNPNNPSISMIRGTRGYMAPEWVFNLPITSKVDVYSYGIVVLEMVTGKSPTTSIDDINGEETYDGRLVTWVREKRSNSNTSWVEQIIDPVIGLNYDKSKIEILITVALKCVLEDRDSRPNMSQVVEMLQCHGSDSH

>Fragaria_vesca-238 (XP_004291073.1)

MDPRFLILLLSLAITSPTSSSTSDLLSEGSSLSVEKPEDVLVSPGRVFKAGFHQVGNNSYCFAIWFNEPSDSYQNRTIVWMANRDQPVNGKRSKLSLLKTGNVILTDAAQSHVWTTTTSSLSPARLSLHDSGNLVLLDLKTKVVLWQSFDSPTDTLLPLQPFTRTAKLVSTRSQSNFSSGFYKLFFDNDNLMRLLFDGPEISSAYWPNPAYVSWDNARSTYNSTRTAVLDSLGSFASSDNLTFLAADYGAKLQRRLTIDFDGNVRLYSREKPGENWKVTWQAFSDPCVIHGICGANSVCSYDPSSGRKCSCLPGYQMQNHTDWSLGCQPEFQFSYGEGESRFLQLSQVEFYGYDYNEYQNYSYDDCEQRCLELDNCKGFQYTFASGVYKCNPKTQLRSGHSWNTQGYFYLRLPKAHLFSNIDINPMKNLGLSCTEKVVDLEREYVKNRVSKPVRFLLWFACGVGGVEIICIVLVWGLLKSNEDIHGYAVTATGFKRFGYAELKTATRGFSEEIGRGAGGVVYKGVLADQRVAAIKVLYEANQGEAEFLAEASTIGKVNHMNLIEMWGYCSEGKHRILVYEYMEHGSLAQKLASNVLDWEKRFDIAVGTAKGLAYLHEECLEWVLHCDVKPQNILLDSNYQPKVADFGLSKLLNRDELKNSSFSRIRGTRGYIAPEWVYNLPITSKVDVYSYGVVVLEMVTGKNPTMVMEMSDAEQRRLITWVREKLNGTDNITSRMGEIIDPSFEDNYDVEKMEILLTIALHCVEEDKDSRPSMSQVVERLLHHGKDS

>Morus_notabilis-238 (EXB85454.1)

MATLFVSYVFFVAITSSLSSSNAHLTLTAGSSLSVERPNDVLTSPNGIFSAGFHLVEENAYYFAIWFNEPSSDKDRTIVWMANRDQPVNRKRSKLSLLKNGNLILTDVGQITIWSTGTVSRSSSQLHLDDTGNMVLRTSDGVVLWQSFDSPTDTLLPEQPLTRSGKLVSSRSQTNLSSSFYKLFFDNDNVLRLIYDSIQFSSVYWPAPWLLSWESGRSTYNNSKTAVLDSLGNFSSSDDFTFMSADYGANIQRMLRIDYDGNVRLYSRKNLGEKWVVMWQAFSDTCKIHGLCGANSLCSYSPNSGRKCSCLQGYKMRNQSDWFYGCEPEFTLTCGKNDSTFIKISNSEFFGYDFGFYPNSTFDACMDLCSQVCHCVGFQYSLYQSRDLLNFNCYPKTLLLNGYQTPSFFGDIYLKVPNTSQFSSRNSIEEFMLDCSNNITTDLPRTYRKSHVNMLVKLMLWFACGVGAFEVICIFCVWCLIIRNRQGSREDTQDYVLHTAGFKRFSFNELKKATRGFSEEIGRGAGGIVYKGVLSDSRVVAIKRLREALQGEAEFLAEVSTIGRVHHMNLIEMWGYCAEGKHRLLVYEYLEHGSLKENLSKNVLDWKMRYEISLGTARGLAYLHNDCLEWVLHCDVKPQNILLGSEYQPKVADFGFSKLLNRGELRNPSFSRIRGTRGYMAPEWVTNQSITSKVDVYSYGIVLLEMLSGRSTSAHDADGVHQETHTRGLVKWVRDKVSGNNNDMTLCMEEVVESWMKGEYAKEKMRILLEVALKCVEEDKDARPTMSEVVEMLQGP

>Theobroma_cacao-238 (XP_007018597.1)

MKLHMSSLCFSVVLSLILLPPLSSATYQKLSGGSSLSVENPRDILVSPNGTFSAGFFPVGDNAYAFAIWFSKPTCVVHNCTVVWMANRDQPVNGRRSKLSLLGTGNVILTDAAQLNVWATDTASLSPVQLQVNDYGNLLLSNSQGTILWQSFDSPTDTLLPLQSLTRYTALVSKRRKANYSSGFYKLFFDDDNVLRLLFDGPDISSVYWPGSWLVSWEAGRSTYNSSRNAMIDSLGNFSSSDDLIFRSADYGASWIQRRLTIDVDGNLRLYSREERDETWVVSWQAISQPCTIHGLCGENSLCGYAPSTGRKCSCPEGYKVKNQTDWSYGCEPEFDVSVNASEFSFIHLRNAEFYGYDISSFNNKTLKECEAICLQTVNCKGFQYKFNGNGFYSCFPKGQLRNGQQTPGFDGDIYIKLPKAYISYNNKKPFKAISLDCPRNDTIMLERSYAKSLENGIVKFMLWFASALGGVEIISIILVWWLTSASQEKNIVAQQGYLLAAAGFKRFTYDELKEATQNFNEEIGRGGVGTVYKGMLPDGRVAAVKRLNEANQGEAEFLAEVNTIGKLNHMNLIEMWGFCAEKKHRLLVYEYMEHGSLAENLSSRELDWQKRYAIALGTAKGLAYLHDECLEWVLHCDVKPPNILLDSGYQPKVSDFGLSKLLDRGKLYNSSFSKIRGTRGYMAPEWVFNRPITSKVDVYSYGIVVLEMVTGLSPTKGIQFVHSIAEREMEYRSVNWVKEKKKESNSMESWITEIVDPMLEGNYDDQKMETLVEVALHCVQEDRDARPTMSQVGERLLHHRENEN

>Prunus_persica-238 (XP_007225256.1)

MGTLFLIFLLSLAIHSPPSSSTSDSLSLSRGSSLSVEKPEDVLVSADGVFTAGFHQVGNNSFCFAIWFTEPSSSDHYQNRTIVWMANRDQPVNGKRSKLSLLKTGNLILTDAGQSNVWATTTTSLSPVHLTLQNSGNLVLQKSDHFVLWQSFEFPTDTLLPHQPLTRNTILVSMRSQSNFSSGFYKLFFDNDNLLRLLYDGLQTSSVYWFEPWLAGQDAGRSTDNSSRIAVLDSLGNFTSSDKYTLMAADYGAKSQRRLTVDSDGNVRLYSWKKPGGKWVVSGQVISDPCKIHGVCGVNSVCSYDPGHGRKCSCIPGYRLINHTDWNYGCEPQYNYTCTKGESTFFKLSKLEFFGYDYGYFTNYTYEDCENLCLKLCNCKGFQYSRIPGRPTYSCYPKTVLLNGYRSPSFFGDLYLRVPKNHLLSYQKPQGEYRLNCLGTVVGLDRNYVKTSVTGSVKFLLWFACGVGGFEIICILLVLGLLRSTRPNSNEDMQGYVLAATGFKRFSYGELKKATRGFSEEIGRGAGGIVYKGVLADQRVAAIKMLNGADPGEAEFLGEANTIGRLNHMHLIEMWGFCSERKHRLLVYEYMEHGSLAQNLSSNVLDWKKRFEIAVGTAKGLAYLHEECLEWVLHCDVKPQNILLDSNYQPKVADFGLSKLFNRGGLKNSSFSRIRGTRGYIAPEWVYNLPITSKVDVYSYGIVVLEMVTGKNPTMGVDAIDGEQRRLITWVREKVNGTTDIASRMGEIIDPSFEGNYDLKKMEILLEVALHCVEEDKDARPTMSQVVEMLLQLEKDESH

>Populus_trichocarpa-238 (XP_002319860.2)

MAISIILFFLPLIFFSSFSSSTIDRLSGASSLSVEHADDVLTSPNGVFSAGFFPVGDNAYCFAIWFSEPYSEGNRTIVWMANRDQPVNGRKSELSLRKSGNVIITDAGRLTVWSTDTVSESSVFLYLHENGNLILQKSEGGVLWQSFDSPTDTLLPQQLLTKDMQLVSSRSQGNYSSGFYKLYFDNDNVLRLLHGGPEITVYWPDPELMSCEASRSTFNSSRIAFLDSLGYFSSSDNFTFMSADYGERVQRILKLDFDGNIRLYSRKYRMDKWTVSWQAMSQPCRIHGTCGPNSICSYVPHFGRKCSCLPGFKIRDRTDWSLGCVQEFNLTCTRNETGFLKLSNVEFFGYDYGFFANYTFGMCENLCLQICDCKGFQFKFIKHAHPSNIPYCYPKTQLLNGHHSPNFEGDIYLKVQKTLPIQEIGLDCSSTVVKQLNRTYTKHQENASLKFVVRFAMVVGSVELGVIFIVWCFFIRTHRNATAGTQNYHRFTTGFRKFTLSELKKATQGFSKEIGRGAGGVVYRGMLSDHRIAAVKRLNDAYQGEAEFQAEVSTIGKLNHMNLTEMWGYCAEGKHRLLVYKYMEHGSLAEQLSSNSLGWEKRFDIAVGTAKGLAYLHEECLEWVLHCDVKPQNILLDSNYQPKVSDFGLSRPLKRGSQVNKGFSKIRGTRGYMAPEWVFNLPITSKVDVYSYGMVLLEMISGKCPAEEIENRRLVTWVREKMKQATEMSSWIEMIIDPKLEGKYDKGRMEILFEVALKCVAEDRDERPTMSQVVEMLLHQENDSELV

>Ricinus_communis-238 (XP_002526220.1)

MATSFVLIVLSLIISPLFSPSATSSSGSLNEGLPISVENPDHVLVSPNGIFSAGFYPVGDNAYSFAIWFNEPSCFNSCTVVWMANRDTPVNGRGSKLSLLKTSNLVLTDAGASSVVWETNTFSLSPSSLHLYDTGNLALVTAQEGVILWQSFDSPTDTLLPLQLFTRESVLVSSRSSTNYSSGFYKLSFDVSNILRLVYDGLDVSSSFWPDPWLLSRDAGRSSYNSSRIAMLDPFGKFISSDNFTFLATDYGILLQRRFTLDFDGNLRLYSRANVSSTWEVSWQVFSQQCKIHGVCGPNSICNYVPGFGRKCSCLPGYKMKNLADWTLGCQTEDKVSCDKNEATFLQFAHVEMYGYDFGYYLNYTLDMCKEVCLQRCDCRGFLLKHNYLVTHPENIPYCYPKTEMLNGYHATSFRGDLYLKVPKTSRSSKNLSMKQLNLECPDGAVKQLDRRYDKSHKSWSQKFLLGFVSTIGIVELLAIFGVWFFLIRSKEKSDQDYILAATGFKRFSYSELKKATRDFSEEIGRGAAGTVYKGVLDDQRVAAIKRLNDASQGEAEFLAEVSTVGKINHMNLIEMYGYCAEGKHRLLVYEYMEHGSLAENLSSKELDWRKRLEIAVGTAKGLAYLHEECLEWVLHCDVKPENILLDDDYRPKVSDFGLSRLLSRADPRNSFSRIRGTRGYMAPEWIFNMPITSKVDVYSYGMVALEMVTGKSPSLMGGQDSETGEELKHKRLVEWVNEKRNEASTKSWVKEIVDPIMGADYDAEKMENLIGVALKCVAEGKDSRPTMSHVVKMILQDEYDHWQ

>Vitis_vinifera-238 (CAN81731.1)

MDAPVLLLLLTLLLSSPLPSSTLDSLSQGSSLSVGKPEQVLISQSGIFSAGFYPVGDNAYCLAIWFTKPSYDGKHTAVWMANRNQPVNGNFSKLSLLESGDLILTDAGRFIVWTIKRVGISPVQLHLFNTGNLVLRTSDGVIQWQSFDSPTDTLLPHQPLTRNTRLVSSRTKTNFFSGFYKLYFDNNNVLSLVFDGRDASSIYWPPSWLVSWQAGRSAYNSSRTALLDYFGYFSSTDDXKFQSSDFGERVQRRLTLDIDGNLRLYSFEEGRNKWVVTWQAITLQCNIHGICGPNSICTYVPGSGSGRRCSCVPGYEMKNRTDRTYGCIPKFNLSCDSQKVGFLPLPHVEFYGYDYGYYLNYTLQMCEKLCLKICGCIGYQYSYNSDVYKCCPKRLFLNGCRSPSFGGHTYLKLPKASLLSYEKPVEEFMLDCSGNRSEQLVRSYAKARENEVLKFILWFTCAIGAVEMICISMVWCFLMKAQQNTSTDPPGYILAATGFRKFTYIELKKATRGFSEEIGRGGGGVVYKGVLSDHRVAAIKQLSGANQGESEFLAEVSTIGRLNHMNLIEMWGYCFEGKHRLLVYEYMEHGSLAQNLTSNTLDWQKRFDIAVGTAKGLAYLHEECLEWVIHCDVKPQNILLDSNYQPKVADFGLSKLQNRGGINNSRLSRIRGTRGYMAPEWVLNLPITSKVDVYSYGIVVLEMITGLRSVANAIHGTDGIGERQSLVAWVKGKMNSATAVASWIEEILDPSMESQYDMGEMEILVAVALQCVELDKDERPTMSQVVETLLRPERGNNHHY

>Solanum_tuberosum-238 (XP_006367420.1)

MNIKFSILVLPLLVVSSYCSTIFSLSSEGSLSAPQDLISSRNGKFTAGFYSVGDNAYIFAIWFTKSLADGTNTIVWMANRNQPINGQKSHLSLFKSGNLVLIDANQINVWESGTQSSTSSVELRLLDNGNLVLVTYEGQRLWQSFDSPTDTLLPEQPLTKTSKLVSRRSLTNFSSGFYQLHFNEDNVLHLVFDGIEMTSVFWPSPWLIVWDAGRSTYNDSKTAVLDRFGNFTSSDRFGFQSADYGVELQRRFSLDVDGNLRLYSLDKLSNIWKVSWQLFPAACRIHGVCGLNSLCSYDPVSGRKCSCVPGYRMKNPKDWSYGCEPEFSISCNDTSSLDFIPLRHVEFYGYDIAYFRNKTLQECKDLCLKHCDCKGFEYKYVEGNGTYGCYPKTLLFNGYVQASWPDFVYLKVPKAQRARQEQYEGNLQCDDKKVMLDRAYRRKEHGGWMKPFIWLVVVAGVFEILCVLTYFIKTRKSSNETTQGYLHLSTRFKKFTYAELKKATSNFSEEIGRGGGGIVYKGKLSDDRVAAIKSLYGASYQGEAEFLAEVSTIGNVNHMNLIEIWGYCAEGKHRLLVYEYMECGSLSDNLNANNLDWEKRFEIALGTAKGLAYLHEECLEWVLHCDVKPQNILLDSNYKPKVADFGLSKIFNRGGLDHSNFSTIRGTRGYMAPEWVFKMPITSKVDVYSYGIVLLEMITGKSPEVCADGDSGDDDSMGLGLLVTWIREKMRENSERKSWIQQIVDPALNGKFDLEKMEILLELALQCSEEDRDARPTMCEVVDKMLHPENFELKLDMLM

>Solanum_lycopersicum-238 (XP_004238122.1)

MRISYYNMNIKISILILASFLVSSYCSTIFSLSSGGSLSAPQDLISSRNGKFTAGFYSVGDNAYIFAIWFTKPLADGSNTIVWMANRDQPINGQKSHLSLYKSGNLVLIDANQINVWESDTQSSTSSVELRLLDNGNLVVVTYQGQRLWQSFDSPTDTLLPEQPLTKISKLVSRRSLTNFSSGFYQLHFNEDNVLHLVFDGIEMTSVFWPSPWLIVWDAGRSTYNDSKTAVLDRFGNFTSSDRFGFQSADYGVELQRRFILDVDGNLRLYSLDKLSNIWKVSWQLFSAACRIHGVCGLNSLCSYDPVSGRKCSCVPGYRMKNPKDWSYGCEPEFDIFCNDTSLLDFIPLRHVEFYGYDIAYFRNKTLQECKDLCLKHCDCKGFEYKYVQGNGTYGCYPKTLLFNGYVQSSWPDFVYLKVPKARRAWQEQYKGNLQCDNKKVMLDRAYRRKEHGGWMKPFIWSVVVAGVFEILCVLTYFIKTRKSSNETTQGYLHLSTRFKKFTYAELKKATSNFSEEIGRGGGGIVYKGKLSDDRVAAVKSLYGANYQGEAEFLAEVSTIGNVNHMNLIEIWGYCAEGKHRLLVYEYMEYGSLSDNLNANNLDWEKRFEIALGTAKGLAYLHEECLEWVLHCDVKPQNILLDSNYKPKVADFGLSKIFNRGGLDHSNFSTIRGTRGYMAPEWVFKMPITSKVDVYSYGIVLLEMITGKSPEVCADGGSGDDDSMGLGLLVTWIRDKMRENSERKSWIQQIVDPALNGKFDLEKMEILLELALQCSEEDRDARPTMCEVVDKMLHPENFELKLDILKI

>Eucalyptus_grandis-238 (KCW68682.1)

MNAMKNALLLLCFSLVLPFPLTTSVRLTLREGSSLSVDKPDDTLTSEDSAFAAGFYPVGENAYCFAIWFSKPSCSIPNCTVVWMANRDQPVNGRSSKLSLRKNGDLVLIDADRTVVWATSTGSSSGSSMKLLQLLDTGNLVLRDEQHVISWQSFDTPTNTLLPLQNLTRNTPLVSHRSKNNFSSGYYRLYFDNDNLLRLLFSGPKITSIYWPNPWLNSWDAGRTSWNDSRVAILDPYGNFTSSDDLKFLTSDYGAKVQRRLTVDYDGDVRVYSRSEGEEWSVSWQAKSDLCTIHGTCGPNSMCTYHPREGRSCICAPGYERENPSDWTQGCKPKFNSFCDSKVETEFVELPHADFYGYDKGVFFKMFTLEMCKKLCLDLCNCLGIQYKNWNCYPKLLLLNGQYSPGVEGGIHFRLPKSNLSSYKLPRIEYAALQCPANEVLLPRTYVKRQESSAIKILLIFVGAIAGTEAIVVLTVWCFLHGTSREKGRAHIDGYHAGPTGFRRFTYDELKKATRNFSEEIGKGSGGTVHKAVLSDSRVAAVKRLLEANQGENEFLAEVSTIGNLNHMNLIDMWGYCAEGKHRLLIYEFMEHGSLAENLSSPTELDWSKRFEIAVGSARGLAYLHEECLEWVLHCDVKPHNILLDSMYQPKVADFGLSKMLKREGLKDSGFSRIRGTRGYMAPEWIYNMPITSKVDVYSYGIVLLELVTGRSPTGGLANTEGGDENESKRLTSSVRERKRTGIPMETWIKEIVDPRLTGEYDTRMVGVLVAVALQCVEEDRDARPTMRQVVEMLLRCSDEDEDEMILA

>Sorghum_bicolor-238 (XP_002458288.1)

MAADKHRLLYPIFLSLLSVPLCSRASPWQAVGTGSSLQVDHGEIFLVSPDTTFSCGFYSSGQGTNAYYFSIWFTHSTDRTVVWTANRGSPVNGHGSKIYFNRQGNLLLTDVNGSTVWQSKTKWGKHASVALLNSGNLVVRASTDQVVWQSFDSPTDTLLPSQRLTREMRLVSQSGYHRLYFDNDNVLRLLYNGPDITSIYWPSPDYNALQNGRTRFNSSKIAVLDNDGIFWSSDGFRMIASDSGFGIKRRITIDYDGNLRMYSLNAAGNWIITGEALLQLCYVHGLCGKGGICEYSQSLKCTCPPGYNMTDPKDWNKGCSPTFNTNCGQPREDFTFIKIPHGDFYGFDLTSNQSISFEECKRICLDSCLCLSFTYKAGQGLCYTKNQLYNGQVYPYFPGDSYIKLPKKITPTYSASNHSTLTCSPKNTKVMLVSKDEYMKNSDNINWTYFYAFATILGAVELLFIMTGWYFLFKMHNIPKSMEEGYKMITSQFRRFTYRELVEATGKFKEELGKGGSGTVYRGILADKKIVAIKKLTDVRQGEEEFWAEVTLIGRINHINLVRMWGFCSEGKHRLLVYEYVENESLDKYLFGDRRTETLLSWSQRFKIALGTARGLAYLHHECLEWVVHCDVKPENILLTRDFEAKIADFGLSKLSKRDSPSFNFTHMRGTMGYMAPEWALNLPINAKVDVYSYGVVLLEIVAGSRVSSGITIDEEEMDFMQFVQEVKQMLARGGNLDIVDARLKGHFSHEQATVMVKIAVSCLEERSKRPTMDQIVKDLMVYNDEDDHPAYSL

>Triticum_urartu-238 (EMS51801.1)

MAASFWILNITFLLISSFVAPTIAEVGRANYLHKGSSLAVKHASHVIESPDGTFSFGFYNLSSTAFTLSIWFTNSADRTIAWSANRDRPVHGIGSKVKLNTDGRSMVLTDYDGTVVWRTNSLTAEADHAELMDSGNLVMKDQGGNILWQSFDHPTDTLLPGQPVTATAKLVSKDLSHPSSYYTLSFDDRYVLSLAYEGPDLSNHYWPNPDHSSWMNYRISYNSSRRAVLDKLGQFVATDNTTFRASDWGLEIKRRLTLDYDGNLRLYSLDEFHRSWYVSWVAFSQPCDIHGLCGWNGICEYSPTPRCSCPPGYVVSDPSDWSKGCKPVFNLTCGQRTGFVRIPETDFWGSDLNYTMSTSMHTCKEMCLGSCACVAFEYKTFPNACFLKSALFNGKTLPGYPGTAYLKVPESFLSQPHTSDSVLHHGHACDASNKQTVSYTTHTNDGQGAMWHYYYWFLAAFFLVEVCFIGSGWWFMSRQHSARSEIWAAEEGYRVVTDHFRSFTHKELRRATKNFKEELGHGRHGSVYKGILHDSRVVAVKKLNDVKQGEDEFEAEVSVIGKIYHMNLVRVMGVCSERNHRLLVYEYVENGSLAMFLFGDKGPLQWHQRYKVAAGVAKGLAYLHHECMDWIIHCDVKPENILLDMDFDPKISDFGFAKLLQRGQADPGSMSKVRGTRGYMAPEWVSTAPLTEKVDVYSFGVVLLELVMGSRVSERATDGREDAEAALQQLEWTIEEKMESDDLTWIDGFVDPRLEGDFVYSEVLLMLEVAAMCLEKEKSQRPSMNHVVQKFLSFD

>Aegilops_tauschii-238 (EMT24870.1)

MTTAVAFTTSISLLLLIPVALAKDHTNGGSYLARGSSVSIEDGTKATTTTILVSPNGAFACGFYRVATNAYTFSIWFRSSSAKTVAWTANRDAPVNGRGSRLAFRKDGALALLDYNGAAVWSTNTSATRASRVELLDSGDLVVVDPNGRRLWGSFDSPTDTLLPSQPMTRHTKLVSASARGLLSSGLYAFYFDNDNQLKLIYNGPEVSSVYWPDPFTTPLVNHRTTYNSSQYGVLEQTGRFAASDNFKFTASDLGDKVMRRLTLDYDGNLRLYSLNATTGGWSVSWMVFRGVCNIHGLCGKNSLCKYIPKLQCSCLRGFEVVDASDWSKGCRRKANLRATQDFSFRKVAGADFIGYDLLYWERVTIRNCKDLCLDNANCQAFGYRQGEGKCFTKVYLFNGKNFPNPHTDIYLKVPRGALSSSELASTVTHECKVHQKEANSTSLLFQDGSSNFKFGYFLSSALTLLFIEVILIIAGCWVVHKWEGRPEIIDEGYTIISSQFRIFSYRELQKATSCFQEELGSGRSGAVYKGVLDDARKVAVKKLNDVIQGEQEFRSEISVIGRIYHMNLVRIWGFCVEKTHKLLVSEFIENGSLAAVLFDHQSNSPVLQWGQRYNIALGVAKGLAYLHHECLEWIVHCDVKPENILLDRDFQPKIADFGLMKLQQRGSSAEMLSKVHGTRGYIAPEWALNLPINGKADVYSYGVVLLELVKGVRLSRWVAEGEEEVEMAGICSIEILKEKLAGEDQSWLLEFVDHRLDGEFNQSEATVMLKIAISCVQEDRSRRPSMSHVVETLLSLVE

>Oryza_sativa-238 (NP_001056908.1)

MAMRGVHIFTTLISFLLMLTTALAEDKKSYLARGSSVSTEDDTKTILVSPNGDFACGFYKVATNAFTFSIWFSRSSEKTVAWTAKRDAPVNGKGSKLTFRKDGGLALVDYNGTVVWSTNTTATGASRAELQNSGNLIVMDSEGQCLWESFDSPTDTLLPLQPMTRDTKLVSASARGLPYSGLYTFFFDSNNLLSLIYNGPETSSIYWPNPAFLSWDNGRTTYYSSRHGVLDSDGWFIATDQLNFEASDHGQKDVMRRLTLDYDGNLRLYSLNMTTGKWSVTWMAFCQVCEIHGVCGKNSLCIYKPDPRCSCLEGFEMVKPGDWSQGCSYKANATLIWNNNANSKSNHGFIFKKIPHTDFYGYDLNYSKPVTLWQCKRMCLDNADCQAFEYHKGIGKCFLKALLFNGRKSQDHYNDFYLKLPKATPYSQLLASKPSHACVMTEKEAYPSSQMLTGNNSNIKFGYFLSSALTLLVVEMTLITVGCWAANKWGRRPEIQDEGYTIISSQFRRFNYKELEKATDCFQEMLGSGGSGAVYKGILDDKRKVAVKKLNDVIHGEQEFRSELSIIGRVYHMNLVRIWGFCAEKTNKLLVSEFAENGSLDRVLSDNLGLFPVLQWSQRYNIALGVAKGLAYLHHECLEWIVHCDVKPENILLDKDFEPKIADFGLVKLVSRGSNTETQSKVHGTRGYIAPEWALNLPITGKADVYSYGVVLLELVKGNRVSRWVVDGEEEVEMAVKRTADVLKEKLASEDQSWLLDFVDCRMNGEFNYSQAATVLKIAVSCVEEDRRRRPSMSSVVEILLSLVE

>Oryza_brachyantha-238 (XP_006644497.1)

MAKLLCLVILPLITILPCSYALPQLTLRTGSSLSVEEHEQTFLTSRNGDFSCGFYEIGGNVFSFSIWFTNSKEKTVVWSANAKSPVNGHGSKVTLSREGNLVLTDVNGTVNWEGRTSSGKGTAAVLLDTGNLVIRDSTGAKLWQSFWEPTDTLLPLQPLTKGIRLVSGYYNLYFDNDNVLRLMYDGPEISSIYWPSAEYKSFDVGRTSYNSSRNAVLDTEGYFLSSDLLSIKASDWGAGIKRRLTLGYDGNLRMYSLNESDGSWIVSWQAIAKMCDVHGLCGKNGICEFLPSFRCSCPPGYTMRDPTNWGRGCQPLFSKNCSKEEEFVFVKLAQTDFYGFDLTYNQSVSLQECKKTCLDICSCSALTYKTGSGLCYTKAVLFNGFSSPNFPGDNYIKVPKNMVAKQSSLSCNRSAEIVLGPASMYGMSGTNKNYTTYYVFAAVLGALVLIFTGTSWWFLYSRNNIPKSMEAGYRMVTSQFRMFMYRELREATGKFKEEIGRGASSIVYRGVLEDKRVIAVKRLANISHSEEEFWVEMSIIGRINHMNLVRMWGFCSEGQHKLLVYEYVENESLDKYLFGNVSAERLLAWSQRFKIALGTARGLAYLHHECLEWVVHCDIKPENILLTRDFEAKIADFGLAKLSQRDSTSLNFTHMRGTMGYMAPEWALNSPINAKVDVYSYGIVLLEIVTGSRISSGIKVDGKDVELREFVQAVRHILASGDIKDIIDSRLNGHFNPEQAKVMVEVAISCLEERSIRPTMNEIAKAFLSCDDQDDHPAYSW

>Setaria_italica-238 (XP_004969487.1)

MARFLSLIVLPLLTILPSSDASPKLMLGTGSSLLVEDYKQTFLTSPNSDFSCGFYEVGGNAFSFSIWFTNTMEKTVVWSANPKSPVNGHGSMVLLNHGGNLVLTDVNGTVTWDSKTGSGKGTTVALLDTGNLIIKGSNGAVLWESFSSPTDTLLPFQPLTKATRLVSGYYSLYFDNDNVLRLMYDGPDISSIYWPSADYSVFQSGRTNYNSSRIAVLDAEGYFLSSDGLNVKSSDWGTKIKRRLKIDYDGNLRMYSLNASNGNWIISWEAIAKMCDVHGLCGQNGICQSLPSFQCSCPPGHEMIDPQIWNKGCQPQFRKTCNNTEEFEFIKIPQTDFYGFDLSYNQSISLEECKRVCLDACSCSAFTYKAGPGLCYTKAVLFNGYSYPSFPGDNYIKLPKNLGISTSLVSRKSHQTCNRDIPEIVEGSASMYGMNSVDKNWTTYYVFAAILGALVLLFTGTSWWFLSSKQNIPKSMEAGYRMVTSQFRIFTHRDLREATGKFKEEIGRGSSGIVYRGVLEDKRVVAVKKLTNFTHSEEELWAEMSIIGRINHMNLVRMWGFCSEGQHKLLVYEYVENESLDRYLFGNVSSERLIAWSQRFKIALGTARGLAYLHHECLEWVIHCDVKPENILLNRDFEAKIADFGLAKLSKRDSSSFKLTHMRGTMGYMAPEWALNLPINAKVDVYSYGVVLLEIVTGSRISSGITVDGREIELGQFVQVLKQFVESEDVKDIVDHRLQGHFNPEQAMIMLKIAVACLEERNSRPTMNDIVVSLLACAEQDDHPAYS

>Hordeum_vulgare-238 (BAJ97178.1)

MTTAMAMAAIKSAYAYTFTTSISLLLLLMILVAPANGRSHGGSYLARGTSVSVEDGTKAAATTVTILASPNGAFACGFYRVATNAFTISIWFTGSSGKTVAWTANRDAPVNGIGSRLAFRKDGALALLDYNGAAVWSTNTSATGASRVELLDSGDLVVVDADGRRLWGSFDSPTDTLLPSQPMTRHTKLVSASARGLLSSGLYTFYFDIDNQLKLIYNGPEVGSVYWPDPFINPLANHRTTYNSSQYGVLEQTGRFAASDNFKFAASDLGDRVIRRLTLDYDGNLRLYSLNATTGSWSVSWMVFRGVCNIHGLCGKNTLCRYIPKLQCSCLRGFEVVDASDWSKGCRRKANLRATQDFSFRKVAGADFIGYDLLYWERVTIQNCKHLCLDNANCQAFGYRQGEGKCFTKVYLFNGKNFPNPHTDIYLKVPKGMLLSSSELASDKVTHACHVHQKEANTSSLMFQDGSSNFKFGYFLTSALTLLFIEVVLITAGCWIVHKRDRRPEIIDEGYTIISSQFRIFSYRELQKATNCFQEELGTGGSGAVYKGVLDDERKVAVKKLNDVMQGEQEFRSEISVIGRIYHMNLVRIWGFCVEKTHRLLVSEFIENGSLATILFDHQSNSPVLQWSQRYNVALGVAKGLAYLHHECLEWIVHCDVKPENILLDRDFQPKIADFGLMKLQQRGSNAQMLSKVHGTRGYIAPEWALNLPINGKADVYSYGVVLLELVKGVRLSRWVVEGEEGVEMADICSIEILKEKLAGEDQSWLLEFVDHRLDGDFNHSEAIVMLKIAVSCVEEERSRRPSMSHVVETLLSLVE

>Brachypodium_distachyon-238 (XP_003571192.1)

MAASFWFLNLTFLLICSFIIAPTVADVGRVNYLHKGSSLSVKHASDVIQSLDGTFSFGFYNLSSTAFTLSIWFTNSADRTIAWSANRDRPVHGTGSKVKLNKDGSMVLTDYDGTVVWQINASSAEVNHAELMDSGNLVVKDRGGNILWQSFDHPTDTLLPNQPITATAKLVSTDLSHTHPSSYYALRFDDQYVLSLVYDGPDISFNYWPNPDHSSWMNYRISYNRSRRAVLDNIGQFVATDNTTFRASDWGLEIKRRLTLDSDGNLRLYSLNKLDRSWYVSWVAFSKPCDIHGLCGWNGICEYSPTPRCSCPRGYIVSDPGDWRKGCKPVFNITCGHGGQRMIFLSNPQTDFWGCDLNYTMSTSLHNCKEMCLESCACVAFVYKTDPNGCFLKSALFNGKAVSGYPGKAYFKVPESFLSRSHKYDSDLYHGHVCDASKKKTLNYETTHNRDGKGTMWYYYYWFLAVFFLVELCFIASGWWFMSTQQSARSEIWAAEEGYRVLTDHFRSFTHKELRRATKNFKEKLGHGRHGSVYKGTLHDSRVVAVKKLNDVKQGEDEFEAEVSVIGKIYHMNLVRVMGVCSEGKHRLLVFEYVENDSLAMSLFGDKGPIQWHQRYKVAAGVAKGLAYLHHGCMDWIIHCDLKPENIFLDLDFEPKISDFGFAKLLQRGQADSSSMSKVRGTRGYMAPEWVSSVPLTEKVDVYSYGVVLLELVMGCRVSELAVDGSEDAESALRQLECTIREKMESDDLTWVDGFVDPRLNGDFVHSEVLLVLEVSAMCLEKEKGQRPSMNHVVQKFLSCE

>Zea_mays-238 (AFW85677.1)

MAMRDLCILTTLVSFLVSLSSVSAAYDHSYLARGSSISTQDVTTPILVSPNGAFTCGFYKVATNAFTFSIWFSWASGKTVSWTANRDAPLNGRGSRLIFHKKGALILVDYNGMVIWSTNTTASGSDRAMLLDSGNLVVMDTDGRHLWRSFDSPTDTLLPWQPMTRDTRLVSASARGLLYSGFYAFYFATNNILTLIYNGPETSSIYWPDPFHMPWDNGRTTYNSTRYGVLDQTGRFVASDQLKFEASDLGDETMRRLTLDYDGNLRLYSLNMTSGNWSVSWMAFPQLCKIHGLCGANSLCRYRPELESCSCLEGFEMVEPSDWSKGCRRKTNTTPFSFRKLTGTDLWGYDLNYSKLVPWLMCRNMCLNDTDCQAFGYRKGTGECYPKAFLFNGRDFPDPYNDIYLKTQEAVWSSPELAPGLRHACKVTEKEAYPLSQMFVGANSSFKFGYFLSSALTLLVIEVILIIVGSWIVYKWERRPEIMDEGYMIISSQFRRFSYKELQRATKSFQEELGSGTSGAVYKGVLDDGREVAVKKLSDMMQGEQEFRSELSIIGRIYHMNLVRIWGFCAEQTHKLLVSEFVENGSLDRYLVDYQDLTYVLQWSQRYNIALGVAKGLAYLHHEWIVHCDVEPENILLDKEFEPKIADFGLVKLLSRGTGAQMLSRVHGTRGYIAPEWALNLPITGKADVYSYGVVLLELVKGVRVSSWVIEGEGVEEMSIRCSAEILKEKLAAKDPSWLMEFVDCRLNGEFNYLQAATMLEIAVSCVEEERTKRPSMDHILKTLLSLVE

>Capsella_rubella-238 (XP_006286357.1)

EDYTMSSFHFYFPSFSLSYSFFCFFLVSSVVQVGLATEPHIGLGSKLKASEPNRAWVSANGTFAIGFTRLNPADRFLLSIWFAQLPGDTTIVWSPNRNSPVTKEAVLELEATGNLVLTDQNTVVWASNTSNHGVEAAVMSESGNFLLLGTEVTTGPAIWQSFSQPSDTLLPNQPLTVSLELTSNPSPSRHGHYSLKMLQQHTSLSLGLTYNINLDPHVNYSYWSGPEISNVTGDVTAVLDDTGSFKIVYGESSTGAVYVYKNPVDETGNHNNSSNFRFSKDPVLRRLVLENNGNLRLYRWDNDMNGSSQWVPDWAAVSNPCDIAGICGNGVCSLDRTKINADCLCLPGSVKLPDQENAKLCSDNSSLVQECESNTNTNNKFKISTVQETNYYFSDRSVIENISDISSVRRCGEMCLSDCKCLASVYGLDDEKPYCWILKSLNFGGFRDPGSTLFVKTRANESYPANSNHNESKSGKSHGLRQKVLVIPIVVGMLVLVALLGMLLYYNIDRKRTLKRATKNPLILCDSPVSFTYRDLQNCTNNFSQLLGSGGFGTVYKGTVAGETLVAVKRLDRALSHGEREFITEVNTIGSMHHMNLVRLCGYCSEDSHRLLVYEYMINGSLDKWIFSSEQTARLLDWRTRFEIAVATAQGIAYFHEQCRNRIIHCDIKPENILLDESFCPKVSDFGLAKMMGREHSHVVTMIRGTRGYLAPEWVSNRPITVKADVYSYGMLLLEIVGGRRNLDMSFEAEDFFYPGWAYKELTNGTALKAVDRRLQGVTEEEEVVKALKVAFWCIQDEVSMRPSMGEVVKLLEGTSDEINLPPMPQTILELIEEGLEDVYRAMRREFNNQLSSLTVNTITTSQSYRSSSLSHATCSYSSMSPR

>Eutrema_salsugineum-238 (XP_006409083.1)

MVSFFFTFFSLFFLCIHVSSAADTISSDFTLSGDQTLVSSNETFEMGFFETGTSSNFYIGMWYKQLSQRTVVWVANRDKSVTDKNASVLKISDGNLVLLDGKNQTPVWSTGLNSTNTSSVLKAVLQDDGNLVLRDESGSVLWQSFDHPGNTWLPGVKIRLDKRTGKSQRLTSWKNDDDPSPGLFSLELDESTAYKILWNGTTQYWSSGPWNSQSRIFDSVPEMRLNYIYNFSFFTNATESYFTYTIYNHLNVSRFVMDVSGQIKQFNWLEANNLWNQFWSQPREQCQVYAYCGSFGICNEKSQPFCQCPRGFRPDSQKDWDLKDYSAGCVRNTELQCSRGDINQFLPLSNVKLPDNPDVVVITSLGNCASACQGNCSCKAYAHDQGSNRCSSWSKDVLNLQQLEEDNSEGNTLYLRLAASDMPSESSGKSNKLMIFGAVLGSLGAIALVLLVVILILRYRRRKRMGREKSDGTLSAFSYRELQNATKNFSEKLGGGGFGSVFKGALQDSSDIAVKRLEGISQGEKQFRTEVVTIGTIQHMNLVRLRGFCSEGNKKLLVYDYMPNGSLDSHLFIDQTEEKIVLGWKLRYQIALGTARGLAYLHDECRDCIIHCDIKPENILLDSQFGPKVADFGLAKLVGRDFSRVLTTMRGTRGYLAPEWISGVAITAKADVYSYGMMLFELVSGRRNTEQSENGKVRFFPSLAATVLTKDGDIRSLLDTRLDGEPVDVEELTRACKVACWCIQDEESHRPTMSQVVQILEGVLEVNPPPFPRSIQALVENDGSVVFFTESSSSSSHNSSQKQSHSSSSSSSKKTSTNNSSA

>Arabidopsis_halleri-238 (ACC68155.1)

MVSFLKLSSFFFLCFFIHGSSAVDTISGDFTLSGDQTIVSSDGTYEMGFFKPGSSSNFYIGLWYKQLSQTVLWVANRDKPVSDKNSSVLKISNGNLILLDGKNQTPVWSTGLNSTSSSVSALEAVLLDDGNLVLRTSGSGSSSNKLWQSFDHPGNTWLPGMKIRLDKRTGKSQRLTSWKSLEDPSPGLFSLELDESTAYKILWNGSNEYWSSGPWNNQSRIFDSVPEMRLNYIYNFSFFSNSTESYFTYSIYNHLNVSRFVMDVSGQIKQFTWLDGNKDWNLFWSQPRQQCQVYRYCGSFGVCSDKSEPFCRCPQGFRPKSQKEWGLKDYSAGCERKTELQCSRGDINQFFPLPNMKLADNSEELPRTSLTICASACQGDCSCKAYAHDEGSNKCLVWDKDVLNLQQLEDDNSEGTTFYLRLAASDIPNGSSGKSNNKGMIFGAVLGSLGVIVLALLVVILILRYKRRKRMRGEKGDGTLAAFSYREIQNATKNFAEKLGGGGFGSVFKGVLSDSSDIAVKRLESISQGEKQFRTEVVTIGTIQHVNLVRLRGFCSEGNKKLLVYDYMPNGSLDAHLFFNQVEEKIVLGWKLRFQIALGTARGLAYLHDECRDCIIHCDIKPENILLDSQFCPKVADFGLAKLVGRDFSRVLTTMRGTRGYLAPEWISGVAITAKADVYSYGMMLFELVSGRRNTEQSENEKVRFFPSWAATILTKDGDIRSLLDPRLEGDEVDIEELTRACKVACWCIQDEESHRPAMSQIVQILEGVLEVNPPPFPRSIQALVDTDEAVVFFTESSSSSSHNSSQKHSHSSSSSSSKKTTNNDSSA

>Arabidopsis_thaliana-238 (Q9FLV4.1)

MSSFHFYFPSVGLFSFFCFFLVSLATEPHIGLGSKLKASEPNRAWVSANGTFAIGFTRFKPTDRFLLSIWFAQLPGDPTIVWSPNRNSPVTKEAVLELEATGNLVLSDQNTVVWTSNTSNHGVESAVMSESGNFLLLGTEVTAGPTIWQSFSQPSDTLLPNQPLTVSLELTSNPSPSRHGHYSLKMLQQHTSLSLGLTYNINLDPHANYSYWSGPDISNVTGDVTAVLDDTGSFKIVYGESSIGAVYVYKNPVDDNRNYNNSSNLGLTKNPVLRRLVLENNGNLRLYRWDNDMNGSSQWVPEWAAVSNPCDIAGICGNGVCNLDRTKKNADCLCLPGSVKLPDQENAKLCSDNSSLVQECESNINRNGSFKISTVQETNYYFSERSVIENISDISNVRKCGEMCLSDCKCVASVYGLDDEKPYCWILKSLNFGGFRDPGSTLFVKTRANESYPSNSNNNDSKSRKSHGLRQKVLVIPIVVGMLVLVALLGMLLYYNLDRKRTLKRAAKNSLILCDSPVSFTYRDLQNCTNNFSQLLGSGGFGTVYKGTVAGETLVAVKRLDRALSHGEREFITEVNTIGSMHHMNLVRLCGYCSEDSHRLLVYEYMINGSLDKWIFSSEQTANLLDWRTRFEIAVATAQGIAYFHEQCRNRIIHCDIKPENILLDDNFCPKVSDFGLAKMMGREHSHVVTMIRGTRGYLAPEWVSNRPITVKADVYSYGMLLLEIVGGRRNLDMSYDAEDFFYPGWAYKELTNGTSLKAVDKRLQGVAEEEEVVKALKVAFWCIQDEVSMRPSMGEVVKLLEGTSDEINLPPMPQTILELIEEGLEDVYRAMRREFNNQLSSLTVNTITTSQSYRSSSRSHATCSYSSMSPR

>Arabidopsis_lyrata-238 (XP_002886239.1)

MVSFLKLSSFVFLCFFIHGSSAVDTISGDFTLSGDQTIVSSDGTYEMGFFKPGSSSNFYIGLWYKQLSQTVLWVANRDKPVFNKNSSVLKMSNGNLILLDSNNQTPVWSTGLNSTSSSVSALEAVLLDDGNLVLRTSGSGSSANKLWQSFDHPGNTWLPGMKIRLDKRTGKSQRLTSWKSLEDPSPGLFSLELDESTAYKILWNGSNEYWSSGPWNNQSRIFDLVPEMRLNYIYNFSFFSNSTESYFTYSIYNHLNVSRFVMDVSGQIKQFTWLDGNKDWNLFWSQPRQQCQVYRYCGSFGVCSDKSEPFCRCPQGFRPKSQKDWDLKDYSAGCERKTELQCSRGDINQFFPLPNMKLADNSEELPRTSLSICASACQGDCSCKAYAHDEGSNKCLVWDKDVLNLQQLEDDNSEGNTFYLRLAASDIPNGSSGKSNNKGMIFGAVLGSLGVIVLVLLVVILILRYRRRKRMRGEKGDGTLAAFSYREIQNATKNFAEKLGGGGFGSVFKGVLPDSSDIAVKRLESISQGEKQFRTEVVTIGTIQHVNLVRLRGFCSEGNKKLLVYDYMPNGSLDAHLFFNQVEEKIVLGWKLRFQIALGTARGLAYLHDECRDCIIHCDIKPENILLDSQFCPKVADFGLAKLVGRDFSRVLTTMRGTRGYLAPEWISGVAITAKADVYSYGMMLFELVSGRRNTEQSENEKVRFFPSWAATILTKDGDIRSLLDPRLEGDEADIEELTRACKVACWCIQDEESHRPAMSQIVQILEGVLEVNPPPFPRSIQALVDTDEAVVFFTESSSSSSHNSSQKHSHSSSSSSSKKTTNNDSSA

>Medicago_truncatula-354 (G7J708)

MQIQVFFLLFLLFTKTTSSQSTTNILPQGSSLSVEKSNNTLISSNGDFSAGFLPVGDNAFCFAVYFTKSKQPTIVWMANRDQPVNGKHSKLSLFKNGNLILTDADRKRTPIWSTSSFSPFPLQLKLQNNGNLVLSTTNGNISILWQSFDFPTDTLLPGQEINERATLVSSKSETNYSSGFYKFYFDNDNALRLLFKSPLLSSVYWPSPWVLPVDAGRSTYNVTKIALLDSFGHFMSSDAYQFVTIDYPKKLHRLLKMDHDGNPRVYSFNDKTKTWEVSWQAIAEPCEVHGICGENSMCSYDPVNGRTCYCLKGYKLKNRNDWTQGCEPEFKPADLSCDSARVEDFGFLHLQNMELYGYDLYVAKVTSLKQCQKLCLDLCEKCKAVQFKFNGVATYDCFPKTLLANGRDSHNIDGDIYLKLPKNTLLSSTIPFKHSPLNCSIALFQPLNRFYEKPSKNSILSFLTWLALGIGVFEFSIILFVWFFLFRTNKNHDDVDQVQRHLLSATGFQRFSYSELKTATRGFSKEIGRGGGGIVYKGTLDDDRVAAVKCLNEAHQGEAEFLAEISTIGMLNHMNLIDMWGYCVEGKHRLLVYEYIEHGSLAENLCSNSLDWNKRFNVAVGTAKGLAYLHEECLEWVLHCDVKPQNILLDTNFQPKVADFGLSKLLNRDERDSSAFSRIRGTRGYMAPEWVYNLRITSKVDVYSYGIVLLEMVSGKSPMEIHSVVDNSGGLEHHHRMVSWVMEKVKSAPTTMFWIEEIVDGNLEGKYDVNQVENLVKVALMCVKDDMNERPSMSQVVEMLLQSHEKRGTPLRTKLPYKRSYRPYHMIEWDVSCQLIINFSY

>Phaseolus_vulgaris-354 (XP_007136716.1)

MHINLFLLLLFISTSYAATDTLPEGSSLSVEKQSDTLLSSNGDFSAGFFQVGENAFCFSVCFTRSKEPTVLWMANRDEPVNGKDSFLSLWKNGNLVLTDAGGTVIWETATLSSSKLHVKLRNDGNLLLLTSKGTIIWQSFDSPTDTLLPTQPLTERVGLVSSRSATNHSTGFYKLYYDNDNVLRLLYKGQTFSSVYWPPSWQLPINIGRSTYNITKTALLDSLGHFTSSDGFQFRSTDYPKKVYRRLKMDSDGNLRLYSFNEEHKMWEVTWQVVPQPCMIHGICGANSLCNHDPNIGRTCYCLKGFKIKDPNDWTQGCEPEFSSSDISCGSRESLGFLHVPTTELYGYDWNVTRVKSLKECQNICLSLCDKCVAVQLKFNEFSDYNCYPKTMAFNGRDSPNFDGEIYLKLPNAILRSSTKILKHSPMNCSVGLSQKLNRFYQPPEKNSTLSFLVWFAFGVGVFELSTSFLVWFFLLRTSKHPDTKDQQHHLLSATGFQRFTYAELKSATKGFKEEVGRGAGGVVYKGTLYDNRVAAIKRLNEATQGEAEFLAEISTIGMLNHMNLIDMWGYCVDGKHRLLVYEYMEHGSLADNLFGNALDWKKRFNVAVGTAKGLAYLHEECLEWILHCDVKPQNILLDSNFLPKVADFGLSKLLNRDERGNSSFSRIRGTRGYMAPEWVYNLRITSKVDVYSYGIVVLEMVSGRSPMAIHSLENSGDIEQHRLVTWIREKIKHSPTCAFWMEEIIDPNLEGNYDVSEVEVLVKVALQCVEDDMNERPSMSQVAEMLQANQNKLLPR

>Glycine_max-354 (XP_003523978.2)

MHLSTSREVPNQPQLIVHVSLYLFIPVLYAMRMVLLFLLLLFVPTSFGTIERLPEGSSLSVEKQNDTIVSSNGDFSAGFFQVGDNAFCFSVWFTRSERPTVLWMANRDKPVNGRGSHLSLWKDGNVVLTDAGGTIIWATATLSSSQQLHLKLRNNGNLVLLASKSTNTTIIWQSFDSPTDTLLTLQPLTEQASLVSSRSTTNHSSGFYKLYFDNDNVLRLLYKGPTLSSVYFPEPWRLPMDIGRSTYNVTKTAVLDSFGRFTSSDGFQFRSTDHPKKLFRRLTMDPDGNLRLYSFDEKLKTWQVTWQLIPQPCTVHGICGANSACNYDRVVGRTCYCLKGFKVKDPNDWTQGCEPEFDPSVFSCNSGESMGFLHYPTTELYGYDWNITVVNSLEECLNLCLELCDKCVAVQFKFNDVAKYNCYPKTMVFNGRYTPNFDGEMYLKLPQAILGSSATPLNKHSTMNCTAGLSQQLERFYEAPSRNSTLSFLVWFACGMGVFELSTIFLVWFFLFRTSKNSETVDQQRHLLSATGFQRFTYAELKSATKGFKEEIGRGAGGVVYKGVLYDDRVAAIKRLGEATQGEAEFLAEISTIGMLNHMNLIDMWGYCVEGKHRMLVYEYMEHGSLAGNLFSNTLDWKKRFNVAVGTAKGLAYLHEECLEWILHCDVKPQNILLDSDFQPKVADFGLSKLLNRDERGNSTFSRIRGTRGYMAPEWVYNLPITSKVDVYSYGIVVLEMVTGRSPMEIHSLENSRGIEQRRLVMWVTDKINDAPTSGFWIEEILDPNLEGQCQVSQVEVLVKVALQCVQDDMNQRPSMSQVVEMLLSHENNVLPR

>Vitis_vinifera-354 (XP_002283062.1)

MDRTVGFFVLALLLTCYPSSSDTYDTLSEGSSLSAEKSSDVLISANGIFSAGFYQVGNNTFCFAIWFTKSLGATTVWMANRDQPVNGRGSKLSLLRNGNLLLTDAGKIMVWMINTVSTSSVRLQLLNTGNLVLYAWEKTVIWQSFDSPTDTLLPHQILTKDTSLISSRSQSNYSSGFYKLFFDSDNVVRLLFNGTEVSSIYWPDPSLVTWDAGRKTFNDSRIAVFDSLGYYRASDDLEFRSADFGAGPQRRLALDFDGNLRMYSLEETRGTWSVSWQAISQPCQIHGICGPNSLCSYTPAYGRGCSCMPGFKIVNSTDWSYGCAPETDIACNQTEVGFFPLPHVQLYGYDYGHYPNYTYERCENLCLQLCKCKAFLLNFSDGLYNCYPKTLLLNGFSSPNYPGTMYLKLPKASLFPRYDPLEEFTINCSGNTRYIQLDTTYRKGHENGSLKFLLWFAFVLGVVETAIVLLVWIFLVRVHHDPVSTMQGYILAANGFKRFSYAELKKATRGFTQEIGRGGGGMVYKGVLLDRRVAAIKRLKEANQGEAEFLAEVSTIGRLNHMNLIETWGYCIEGKHRLLVYEYMEHGSLAQKLSSNTLDWEKRFQIALGTARGLAYLHEECLEWVLHCDVKPQNILLDSNYQPKVADFGMSKLRNRGGLDNSSFSRIRGPRGYMAPEWVFNLPITSKVDVYSYGIVVLEMVTGKSPTAISDTDAQGETEQRGLIKWMRDRMNGIGARGSWIEDILDPVMQGECDMRQMEILIGVALECVEEDRDSRPTMSQVVEKLMCPEERAEQDGYLFMS

>Morus_notabilis-354 (EXB89237.1)

MDFRILFLLLPLLFQAPFSSSTNTLLPGSSLSVKSATSDVLVSPNGVFSAGFKAVGENAFCFAIWFAISSDPTIVWMANRDQPVNGRGSKLSLTKNGNLVLLDAGRVTLWSTGTRNISAVKLQLEDTGNLVLRTSEGGQSFTLWESFKSPTDTLLPQQKLTMDTDVVSSKSQTNYSSGYYKLYWDDDNVLHLLCHGPDRQLSSVYWPYPWLKYFENGRSTYNNSKTAVLNSFGVFSSSDDLFVLASDYGEKLHRRLTLDPDGNLRLYSFDMKKSTWVVTWQAFPKPCDIHGTCGPNSFCTYHHSFGQRCSCIHGFKMANHRDWFYGCEPEVKWNNSREIDGFFRVANAEFYGYDRGFFQNQTLKGCEEVCLNLTECKGFQFKFEDSVYKCYPKTQLLNGQRASNFNGDVYIRVPKANLSFYEKPGKEIELDCPSKSVILTTAYKKPHENATLMVLVWVSVGLAGMEFVCVAVVLCFLYLTRNGKDEKAQGYMLAATRFQEFTYADLRKATRGFCEEIGNGGGGTVFKGMLSDGRVAAIKRMREAHQGEAEFLAELSIIGRLNHMNLIEMWGYCAEGKHRLLVYEYMEHGSLADNLSADHALDWEKRFAIAMGSSKGLAYLHDECLEWVLHCDVKPDNILLDSDYQAKVADFGLSKIQNRGEAHNSSFSRIRGTRGYMAPEWVYNLPITSKVDVYSYGIVVLELVTGRKPTGVQIVIGGGGGTAEHGRLVTWVREKVNGASTTEAWIEEIADPILEGNFDRNEMEVLVKVALQCVEEDKEARPTIRQVVEMLQCHEDVRAITDVLIKPS

>Prunus_persica-354 (XP_007227238.1)

MAATFLCLALLFLSQFSLLASSASNNLSLGSSLSVENEADTLTSPNGLFSAGFFPVGKNAYCFGIWYTEPAAPAQNLTVVWIANRDKPVNGKSSKLTLQGNGNLILTDAGKYTIWSSNTVSNSLDRLSLNDNGNLVLQTLKGVALWESFASPTDTLLPQQQLTRNTKLVSSRSLTNYSSGFYSLFFDNDNTLRLLYDGPEVSSIYWHDPWLLSLQARRTPYNNSRVAVLDAMGNFSASDGLIVIAADYGTNLQRRLKADVDGNLRLYSRKQAGDTWVVSWQAIQQPCKIHGICGPNGLCTYDPSSGRKCSCLPGYEMRNKFDWAYGCQPATDGLNLSCKSSDRDQFDFMYLPRVEYYGYDFSIVVNTTLEKCKALCLELCDCPGFHYKFGGGHHNCYPKTQFRNGYRAPGFQGELYLKLPKSILSSYNAATKESNMCSSKLTSQLDRTYEKGKQNGSVKFMVKFASGLGGFEIVCIFVVWYFVARRGEEKEDSDVVTQGYLQAATGFRRFSYSELKKATRGFKEEIGRGGGGIVYKGVLSDQRIVAIKQLNGANQGEAEFLAEVSLIGKLYHMNLIEMWGYCAEGKHRLLVYEYMEHGSLAEKLSSEVLDLKTKFEIAVGTAKGLAYLHEECLEWVLHCDVKPQNILLDSNYNPKVADFGLSKLLNRNEVSNSNFSKIRGTRGYMAPEWVHNMPITSKVDVYSYGIVVLEMLTGKSPAESVQAIESGGETQKKMMVTWVKEKMHGAFTKTESRMGADYEQGRLEMLVKVALQCLEEDRDARPTMSQVVEMLLHHENN

>Ricinus_communis-354 (XP_002523485.1)

MAVRSFFFALPLLLVLSSQFLSSASNSLREDSFLSVENTNDVLTSPHGAFVAGFFPVGDNAYCFAIWFSEPFCSNNCTVVWMANRDEPVNGKHSHLALLKSGNLILTDAGQVTVWATNTVSESSVQLYLQESGNLVLQKLDGAILWQSFDFPTNTLLPLQPITKDWQLVSSRSESNYSSGFFRLYFDNDNVLRLLYAGPETSSIYWPDPELLSWEAGRSTYNNSRIAYFDSLGKFSSSDDFTFFAADYGVKLQRRLTIDFDGNLRLYSRKDGIDLWTVSWQAMSQPCRVHGICGPNSVCNYVPSSGRKCSCLEGFKMKDVTDWSLGCEPEYSLSCSRNESTFLVLTHVEFYGYDFVYYPNYTFDMCENVCLQRCDCKGFQLKFIKHDYPSNIPYCFAKSLLLNGHHSPSFEGDLYLKVPKTSSSSSSSVAKFSLDCFQEVVKQVDKVYTKSHENGSLKFVFWFAIIIGIIEFTVIFLVWYFLIRTHQHSGVVRAGYLQIATGFRKFSYSELKKATRGFREEIGRGAGGIVYKGILSDHRVAAIKRLIINEADQGEAEFRAEVSVIGKLNHMNLIEMWGYCAEGSHRLLVYKYMEHGSLAQNLSSNKLDWERRYDIALGTAKGLAYLHEECLEWVLHCDVKPQNILLDSDYQPKVSDFGLSHPLKRDSHEISRLSRIRGTRGYIAPEWIFNLPITSKVDVYSYGMVLLEIVTGKSPAADIGDRGLVKWVRKTIDSSTAMIFWMEKIVDLNLGGKYDKNQMEILIGVALKCAHEDKDARPTMRQVVEMLLQDGKDTKPLTVT

>Eucalyptus_grandis-354 (KCW68689.1)

MRNSSFLLVLILSILHLSSQPSSATHDTLSRGDSLSIDNPDDILISKSGVFSAGFYPVGKNAYCFAIWFSHPPCSGQNCTVVWVANRDKPVNGRYSKLSLKKNGNLVLTDASQTVVWATETDPLSCSKTPFHQLQLLDSGNLVLRDRECNVPLWQSFDYPTDVLLPGQKLTRNTPLVSSRSNCNFSSGYYKLYFDNDNVLRLLFDGPLIPSVYWPDPGRVSWEVGRTTYNDSRVAVLDSLGNFTSSDDLKFLTSDFGQKTQRMLKADHDGNIRVYSREKDGKWAVTWQGLSESCTVHGICGPNSVCSYSPSEGRSCSCPPGYVWSNDTDWTQGCAPRFESFCNKGAHAHVDFLRLPHHDFYGYDRAYFRNTTLAWCKKKCADLCDCKGFQYKFNGNRGSFDCYPKIRLLNGYFSFSFNGSIYLKLPKANLSSYQRPTKGSNSLSSGVEIVVVFLTWSFLIGTDLDKLGGYAKGYHLAGTGFTRFTYAELKKATKNFSKEIGRGSGGIVYKGVLPDHGEAAVKLLNKANQGEDEFLAEVRTIGNLNHINLITMWGYCVEGRHRLLVYEFMNHGSLAENLSSDQLDWKKRYDIAVGSAKGLAYLHEECLEWVLHCDVKPHNILLNFDYQPKVADFGFSKLLNRDSFKNTSFSRIRGTRGYMAPEWVYNMPITFKVDVYSYGIVLLEMLTGGNLVASHAIDGQVKGGNKRLTTWVREKMKPGVAMETWIREIMDPTVKGPYDIKKMEVLVTVALQCVEEDKDARPTMSQVVEMLLRHESEN

>Oryza_brachyantha-354 (XP_006644495.1)

MSKFLYTVSLSYLSVLLCCGASPWQTIGTGTSLQVDRGETLLVSPDTTFSCGFYPSGDDTNAFYFSIWFTHATDRTVVWTADSGLPVNGHGSKISLSHEGNLVLTDVNGTTVWESKTGWGKHTTVSLLNSGNLVIKASDDKIVWQSFDWPTDTLLPSQRLTREKRLVSQSGYHVLYFDNDNVLRLLYNGPDITSIYWPSPDYNAVQNGRTRFNSSKIAVLDDEGRFLSSDGFKMVALDSGLGIQRRITIDYDGNFRMYSLNASNGNWNITGEGVLQICYVHGLCGRNGICEYSPGLRCTCPAGYEMVDPENWSKGCRPTFSISCGKQREDFTFIKIPHGDYYGFDLTSNKSISFEECMLSCKDSCVCLSFTYKSGEGLCYTKDLLYNGQVYPYFPGDNYIKLPKNVASTSLVSKHPDLTCKTNASKVMLASIDAYRKNSDNIMWAYLFIFASIIGAVELVFIMIGWYFLFKMHNIPKSMEEGYKMITSQFRRFTYRELVEATGKFKEELGKGGSGTVYRGVLGDKKVVAVKKLTDVRQGEEEFWAEVTLIGRINHINLVRMWGFCSEGRHRLLVYEYVENESLDKYLFDDSGTRRLLSWSQRFKIALGTARGLAYLHHECLEWVVHCDVKPENILLNRDFEAKIADFGLSKLSKRDSTTFNFTHMRGTMGYMAPEWALNLPINAKVDVYSYGVVLLEIVTGSRVSSGITIDEEDIDLMQFVQKVKQILTSGADLDNIVDNMLKGHFNFDQAKVMVKTAVSCLEERSKRPTMDQIIKDLMVYDDEDYHPAYL

>Oryza_sativa-354 (EEE55152.1)

MGKFLCPLFISFISVLLCCAASASPWQTTGTGTSLQVDHGETFLVSPDTTFSCGFYPSGDDTNAFYFSIWFTHATDRTVVWTADSGLPVNGHGSKISLSHEGNLAFTDVNGTTVWESKTGWGKHTTVALLNSGNMVMKASDSEDKIVWQSFDWPTDTLLPSQRLTREKRLVSQSGNHFLYFDNDNVLRLQYNGPEITSIYWPSPDYTAVQNGRTRFNSSKIAVLDDEGRFLSSDGFKMVALDSGLGIQRRITIDYDGNLRMYSLNASDGNWTITGEGVLQMCYVHGLCGRNGICEYSPGLRCTCPPGYEMTDPENWSRGCRPTFSVSCGQQREDFTFVKIPHGDYYGFDLTSNKSISLEECMRICMDSCVCLSFTYKGGDGLCYTKGLLYNGQVYPYFPGDNYIKLPKNVASTSLISKHHGLTCKPNASKVMLVSIDAYRKNSDNIMWAYLYVFATIIGAVELVFIMTGWYFLFKMHNIPKSMEKGYKMITSQFRRFTYRELVEATGKFKEELGKGGSGTVYRGILGDKKVVAVKKLTDVRQGEEEFWAEVTLIGRINHINLVRMWGFCSEGRQRLLVYEYVENESLDRYLFDDSGTRNLLSWSQRFKIALGTTRGLAYLHHECLEWVVHCDVKPENILLNRDFEAKIADFGLSKLSKRDSSTFNFTHMRGTMGYMAPEWALNLPINAKVDVYSYGVVLLEIVTGTRVSSGITIEEENIDLMQFVQVVKQMLTSGEVLDTIVDSRLKGHFNCDQAKAMVKAAISCLEERSKRPTMDQIVKDLMCFLIFHRKTPTGTIEICWKCLYSRSKSKLFLSLPY

>Setaria_italica-354 (XP_004972083.1)

MRIRFVVSMPTQQCNMLGKSTSLALLIFLHSLSPCSSAPAPSQLGAGSTLSVEDHVRSFLVSPDATFSCGFLEAGENAFSFSVWYTGSADKTAVWTASPGAPVNGRGSRITFRHDGGLALADANGTAVWESKTSGGGLVISLLDTGNLVISDPSNNGGPAPWQSFDWPTDTLVPSQPLTKHMKLVAGYFSLYYDNDNVLRLLYDGPDTSSIYWPSPDNTVFGNARTNYNSSRIAVLDDTGVFLSSDNLAVRASDLGPGVKRRLTIDPDGNVRIYSLNATTGGWKVTWAAMAQPCSVHGVCGQNATCEYQPGLRCSCLPGYEMSDREDWRKGCKPMFGVTNCSQDQAAAPEERFKFVRVPQTDFYGYDLGYNSSVTFEHCKKLCLEMCLCTAFSYRLTGIGVCYPKGTLFNGYTSPNFPGSIYIKLPIDFDTSAQSVSARSSEGLACNPNGPKIVQGSPDTFRRPRNNTKWSYLYAFATVLGVLDVIFIATSWWFLSSKQSIPSSLEAGYRMVTGQFRRFTYRELKDATGNFKEELGRGGSGVVYRGVLDKGKVVAVKKLTNVAGGDEEFWAEMTVIGRINHINLVRIWGFCSQGKRKLLVYEYVENESLDRHLFATDRTLSWRERYRIALGTARGLAYLHHECLEWVIHCDVKPENILLTREFDAKIADFGLAKLSKRDGAGDSSMQLTHMRGTTGYMAPEWALNVPINAKVDVYSYGIVLLEIVIGSRISEQRTAGGERLEMLQTAEVLRQVVASGDIAPLVDGRLQGQFNPRQALEMVRISLSCMEERSKRPTMDDIAKVLMVCDDEDEHPAYRS

>Triticum_urartu-354 (EMS62449.1)

MDTFLFLAILPLLTALPCSYAQSMLSTGSSLSVEEHRQTFLTSPNDDFSCGFYEVGENAFSFSIWFTTTMEKTVVWSANPRSLVNGHGSMVSLNHNGNLVLTDVNGTVTWESKTSSGEGTVVSLLDTGNLIIKDYTGANLWESFSSPTDTLLPLQTLKKGTKLVSGYYSLYFDNDNVLRLMYDSPEISSIYWPSADYTVFQNGRTSYNSSRIAVLDTEGFFLSSDGLNVKASDWGNGVKRRLTIGSDGNLRMYSLNVSNGSWIVSWQAIAKLCDVHGLCGQNGICEFLPSFRCSCPPGYEMTDPENWNRGCKPQFSKGCSKAEEFDFIKLPQTDFYGFDLTNNVSVSLEECKKTCLDICSCSALTYKAGSGLCYTKAVLFNGYSSTNFPGDNYIKLPKNMSISRQSRITCNPDIRVVVQGSASMYGMNDVNKSYTIYYVFAAILGALVLLFIGTSWPFLYSKQNIPKSMEEGYRTVMSQFRMFIYRELREATGKFKEEIGRGGSGIVYRGVLEDKRVVAVKKLTSVSHSEEEFWAEMNIIGRINHMNLARMWGFCSEGQHKLLVYEYVESESLDKFIFGNVSAERLLAWSQRFKIALGTARGLAYLHHECLEWVIHCDIKPENILLTRDFEAKIADFGLAKLSKRDSSSFNLTHMRGTMGYMAPEWALNLPIDAKVDVYSYGVVLLEIVTGNRISSGITVDGKEVEFRQFVHAVKELLESGDVKVIADARLNGHFNPEQVAVMVKLALSCLEERNSRPTMNEIVKALLACDDEDNHPAYSW

>Brachypodium_distachyon-354 (XP_003564268.1)

MAAMRGACIFTTSIFFLSMLISVNALAKDHGSSYLARGSTVDTWDGETTAILVSPNGAFACGFYRVATNALTFSVWFHASSRRKTVVWTANRDEPVNGRGSSLAFRKDGGLALLDYNGTAVWSTNTTATSASHAKLLDNGNLVVMDPGGRSLWGSFDSPTDTLLPSQPMTRNTKLVSASARGLLYSGLYTLYFDSDNQLKLIYNGPEISSIYWPNPFNKPWVNKRSTYNSSRYGILEETGRFVASDKFEFEASDLGDKVMRRLTLDYDGNLRLYSLNPTSGNWSVSWMAFHRVCDIHGVCGKNSMCKYIPKLQCSCLKGFEVIDASNWSEGCRRKANITASWDKHRRDNANITASWDKHRRANANSTTTQDFSFRKLAETDFYGYDLAYDEWIPFSKCRNMCLGYVDCQAFGYRKGEGKCFPKVYLFNGKNFPDPPNDIYLKVPKGLLPSPELASTIAYECKVHEKEANVSLQMLKGGTSKFKFGYFLSSALTLLFIEVTLIIAGCCVVYKSERRVEIADEGYMIISNQFRIFSYRELQKATRCFQEELGSGGSGAVYKGVLDDERKVAVKKLNDVIQGEQEFRSELSVIGRIYHMNLVRIWGFCAEKTHRLLVSEFIENGSLDRALFDYQSLFPVLQWSQRYKIAVGVAKGLAYLHTECLEWIVHCDVKPENILLDEDFEPKIADFGLVKLLTRGSNTEMLSRVCGTRGYIAPEWALNLPITGKVDVYSYGVVLLELVKGVRVSRWLVEGEEGVEMAVRCSTQILKEKLAGEDQSWLLEFVDYRLDGEFNHSEAILMLKIAVSCVEEERSRRPSMGHVVETLLSLVE

>Sorghum_bicolor-354 (XP_002437895.1)

MRDLCIFTTFVSFLISLSSVSVANDQSYLARGSSISTQDVTTAILVSPNGAFTCGFYKVATNAFTFSIWFSWASGKTVSWTANRDAPVNGRGSRLIFRKNGGLILVDYNGMVIWSTNTTASRSDRAMLLNSGNLVVMDTDGRHLWRSFDSPTDTLLPLQPMTRNTKLVSASARGLLYSGFYAFYFASNNILTLIYNGPETSSIYWPDPFYMPWDNGRTTYNSTRYGVLDQTGRFVASDQLEFEASDLGDEMMRRLTLDYDGNLRLYSLNITTGSWSVSWMAFPRMCNIHGLCGANSLCKYRLELESCSCLEGFEMIEPSDWSKGCRRKTNTMPFSFRKLPGTDLWGYDLNYSELAPWWMCRDMCLNNTNCQAFGYRKGTGECYPKAFLFNGRNFADPYNDIYLKTPKAVWSSPELAPGPIHICKVTEKEAYPSSQMFAADHSSFKFGYFLSSALTLLVIEVTLIIVGCWVVNKWERRPETMDEGYMIISSQFRRFSYKELERATKCFQEELGSGTSGAVYKGVLDDGREVAVKKLSNMMQGEQEFRSELSVIGRIYHMNLVRIWGFCAEQTHKLLVSEFVENGSLDRFLFDYQDLTYVLQWSQRYNIALGVAKGLAYLHHEWIVHCDVEPENILLDKEFEPKIADFGLVKVLSRGAGAQMLSRVHGTRGYIAPEWSLNLPITGKADVYSYGVVLLELVKGVRVSSWVIEGEEVVEMSIRCSAEILKEKLATQDLSWLLEFVDCRLDGEFNYLQAATMLKIAVSCVEEERSKRPSMDHILKTLLSLVE

>Eutrema_salsugineum-354 (XP_006396235.1)

MPCTYLLLPLLLCLLFPLSSSESKVIIKGNQTILSFNSIFRLGFFSPTNGESFTSSQSSNWYVGIWYASIPTPTYVWVANRNRPLSDPHSSTLELTSTGNLIARNSHDGVVWQSDNNHPGTDFRFSDTGNLILIGDHGSPVWQSFENPTDTWLPGMNVTGLTAMTSWRTPFDPSPGLYSLRLSPSFNEFQLVFNGTTPYWSTGNWTGESFVGVPEMTVPYIYRFHFVNPYTPAASFWYIVTPSDASPEPRLTRFLVDYNGQLKQYTWEPQTNSWNMFWLQPEDPCRVHGLCGQLGFCSSKLLKPCACIRGFRPKIDAAWRSEDFSDGCLRESADSCDGRDTFEAVGDLRYDGDVEISRFQVSKSSCARSCLGNCSCVGFYHNDKSNLCKILLESPLNLKNSSSWTGITDDILYIRDPRKGNSKGNISKFVIILCSVVGSITVLGFVPLILLRRNRKRKKTRKEDEDGFAVLNLKVFSFKELQKATDGFSEKLGHGGFGAVFKGTLPESSTSVAVKRLERPGSGEGEFRAEVCTIGNIQHVNLVRLRGFCSENLHRLLVYDYMPNGSLSSYLSRTSPKLLSWESRFRIALGTAKGIAYLHEGCRDCIIHCDIKPENILLDGDYNAKVSDFGLAKLVGRDFSRVLATMRGTWGYVAPEWISGLPITTKADVYSFGMTLLELIGGRRNVIVDSDTPGEKEDKGEKESEKWFFPPWAAREIIQGNVDLVVDSRLNGEYNIEEATRMATVAIWCIQDNEEIRPAMGTVVKMLEGVVVVTVPPPPKLIQALVSGDSYQGVDHGLSGTSCSEGRGCSDLVTGLSSPGSRSSFGRPSSP

>Arabidopsis_thaliana-354 (NP_567172.4)

MPCTTYLPLLLLLFLLPPPSVQSKVIIKGNQTILSFKAIFRLGFFSTTNGSSNWYLGISYASMPTPTHVWVANRIRPVSDPDSSTLELTSTGYLIVSNLRDGVVWQTDNKQPGTDFRFSETGNLILINDDGSPVWQSFDNPTDTWLPGMNVTGLTAMTSWRSLFDPSPGFYSLRLSPSFNEFQLVYKGTTPYWSTGNWTGEAFVGVPEMTIPYIYRFHFVNPYTPTASFWYIVPPLDSVSEPRLTRFMVGANGQLKQYTWDPQTQSWNMFWLQPEDPCRVYNLCGQLGFCSSELLKPCACIRGFRPRNDAAWRSDDYSDGCRRENGDSGEKSDTFEAVGDLRYDGDVKMSRLQVSKSSCAKTCLGNSSCVGFYHKEKSNLCKILLESPNNLKNSSSWTGVSEDVLYIREPKKGNSKGNISKSIIILCSVVGSISVLGFTLLVPLILLKRSRKRKKTRKQDEDGFAVLNLKVFSFKELQSATNGFSDKVGHGGFGAVFKGTLPGSSTFVAVKRLERPGSGESEFRAEVCTIGNIQHVNLVRLRGFCSENLHRLLVYDYMPQGSLSSYLSRTSPKLLSWETRFRIALGTAKGIAYLHEGCRDCIIHCDIKPENILLDSDYNAKVSDFGLAKLLGRDFSRVLATMRGTWGYVAPEWISGLPITTKADVYSFGMTLLELIGGRRNVIVNSDTLGEKETEPEKWFFPPWAAREIIQGNVDSVVDSRLNGEYNTEEVTRMATVAIWCIQDNEEIRPAMGTVVKMLEGVVEVTVPPPPKLIQALVSGDSYRGVSGTSCSEGHGCSDLNTGLSSPGSRSSFGRPSP

>Arabidopsis_lyrata-354 (XP_002875034.1)

MPSCITYLHLLLLLLLFLLPPLSSSVDNNKVIIKGNHTILSFKAIFRLGFFSTTNGSSNWYLGISYASMPTPTHVWVANRIRPVSDPDSSTLELTSTGHLIVRNSRDGVVWRTDNKEPGTDFRFSETGNLILINDDGSPVWQSFDNPTDTWLPGMNVTGLTAMTSWRTLFDPSPGFYSLRLSPGFNEFQLVYKGATPYWSTGNWTGEAFVGVPEMTIPYIYRFHFVNPYTPAASFWYIVPPLDAVSEPRLTRFMVGANGQLKQYTWDPQTQSWNMFWLQPEGPCRVYSLCGQLGFCSSELLKPCACIRGFRPKNDDAWRSDDYSDGCRRENGESGEMSDTFEAVGDLRYDGDVKMSRLQVSKSSCAKTCLGNSSCVGFYHNENSNLCKILLESPINLKNSSSWTGISNDGNISKSIIILCSVVGSISVLGITLLVPLILLKRSRKRKKTRKQDEDGFAVLNLKVFSFKELQAATNGFSDKVGHGGFGAVFKGTLPGSSTFVAVKRLERPGSGESEFRAEVCTIGNIQHVNLVRLRGFCSENLHRLLVYDYMPQGSLSSYLSRTSPKLLNWETRFRIALGTAKGIAYLHEGCRDCIIHCDIKPENILLDSDYNAKVSDFGLAKLLGRDFSRVLATMRGTWGYVAPEWISGLPITTKADVYSFGMTLLELIGGRRNVIVNSDTLGEKDTEPEKWFFPPWAAREIIQGNVDSVVDSRLNREYNMEEVTRMATVAIWCIQDNEEIRPAMGTVVKMLEGVVEVTVPPPPKLIQALVSGDSYRGVSGTTCSESRGCSDLNTGLSSPGSRSSFGRPSP

>Medicago_truncatula-4055 (G7J0P4)

MILMIHSPIFFTIFILLFHFQHSLSFSLSVERHENDIIVSPKGTFTAGFYPVGENAYSFAIWFTQKHKNLSNPTVVWMANRDQPVNGKRSTLSLLKTGNLILTDAGQFNVWSTNTNSSKKLELFLCDTGNLILREHNTIVSNFLWQSFDSPTDTLLPDQSFTRYMQLISSKSKNDYSSGFYKLLFNYDNLLCLIYDGPQVSRVYWPVPWLLSWQSGRSTYNSSKVAILSPLGEFSSSDNFTITTSDYGTLLQRRLSLDHDGNVRVYSRKHGQEKWSVSAQFRIGSCNIQGICGPNGVCSYNFRSGRKCSCLPGYIRIDNQDWFQGCKPSFQLSCDNKTSSNTRFQHLPHVKFYGYDYGTYANYTYKQCKHLCMRLCECIGFEYTYRKQSGTYSCHPKTKFLNGFHSPHFQKSIFLLLPKNNIDFSHENIVKDDSLVCSQNAPKQLVRLYVKGKDNRSVKFMLWFASGLGGIEFFCFFMVWCFLLKNRKHSSEGNHNYVLAAAATGFTKFTYSELKHATKNFSQEIGKGACGTVYKGVLSDNRVAAIKRLHEANQGESESEFLAEVSIIGRLNHMNLIGMWGYCAEGKHRLLVYECMENGTLADNLSSNKLDWSKRYNIAMGTAKGLAYLHEECLEWILHCDIKPQNILVDSDYQPKVADFGLSKLLNRDDFDHSKFSRIRGTRGYMAPEWVFNMEITSKVDVYSYGVVVLEMITGKSPTTGIQIKDKEELCHERLVTWVREKRRKGLEVGCWVEQIVDPKLGSNYDVKKMETLADVALDCVKEEKDVRPTMSQVVERLLRDEHN

>Cicer_arietinum-4055 (XP_004500314.1)

MILTMNSSPILFALLILLFHFHYSSSSFSLSVENLEQDIIVSPKRIFTAGFYPVGENAYSFAIWFTQKHKHLNDATIVWMANRDQPVNGKKSTLSLLKTGNLILTDAAQSIVWSTNTASSKPLQLSLRDTGNLILQEHNNSNSIILWQSFDFPTDTLLPQQTLTTFTKLISSRSETNYSSGFYKLFFDNYNLLCLVYDGLEVSSVYWPDPLILDWQTSRSTYNSNRVATLNRFGNFSSSDGFTFRTSDYGTVLQRRLKLDFDGNVRVYSRENGQEKWSMSGQFNQQPCKIHGICGPNSTCSYDPITGRKCYCLPGYSIINNQDWSQGCKPSFQVSCNNNNKLESRFKLLPHVEFYDYDYGFQKNYTYKHCKHLCLRLCECIAFQFRYVKNKGLFNCYPKTRLLNGRSSTKFGGSLFLRLPKNNSLFSHENSVEEDVNSLVCLRNEGVKQLGRSYVNAKENGSLKFMLRFAGGLGGVELLGFFLVWCFLFRSSKNSNEDNNGYVLAAATGFRRFNYSELKQATKGFSQEIGCGAGGKVYKGVLSDNRIAAIKRLHEAHHGESESEFLAEVNIIGRLNHMNLIGMWGYCAEGKHRLLVYEYMEKGTLADNLSSNELDWGKRYNIALGTAKGLAYLHEECLEWVLHCDIKPQNILVDSDYQPKVADFGLSKLLNRDDLNNSNFSRIRGTRGYMAPEWVFNSQITSKVDVYSYGVVVLEMITGKSPTTGIQIKDGEELCHERLVTWVREKRRKGIEVGCWVEQIVDPALGSNYDVKKMETLANVALDCVAEERDMRPSMSQVVERLQSQSASYLIVFTN

>Phaseolus_vulgaris-4055 (XP_007134942.1)

MATSSPTFIAILLLMLLHLHSSAALPLSVGSPDEDVIVSRPKGTFTAGFRRVGENAYCFGVWFSQAEKHVVWMANRDVPVNGKRSSLSLLKNGNLVLSDAGQFDVWDTKTLSAKPLELHLHDTGNLVLREQTNRSSVLWQSFNFPTDTLLPGQILTRFTTLVSSRSEGNYSSGFYKLFFDNDNVFRLLYEGPQVSSVYWPDPWLVSNNVGSGNGRSSYNSSRVAVLDELGKFSASDAFSYKTIDYGLLLQRRLTLDYDGNVRIYSRENGQEDWSITGQFKSQPCFIHGICGPYSICNHEQATGRKCVCLEGYSWIDSQDWSLGCKPNFQPTCDNKTEYRFVALYEVDFYGYDYGSSFSNYTYKQCEKWCSELCQCKGFQFSFPQDNGPFWCYPKTQLLNGHRSPGFRGQFFLRLPKGSAKENNLQNNEGLVCPRNAEKVLERPYVKGKENGSVKFVLRFAVGLGGFEVVCIFMVWCFLFRSSNHLVREDQQGYVLAAAAGCRRYTYSELKQATKGFSEEIGRGAGGTVYKGMLSDNRIAAIKKLHEFADQGESEFLTEMSIIGRLNHMNLIGMWGYCVDGKHRILVYEYMENGSLANNLSSNSLDWSKRYNIAMGMAKGLAYLHEECLEWILHCDIKPQNILLDSDYQPKVADFGLSKPLNRNNLNNSSFSRIRGTRGYMAPEWVFNLQITSKVDVYSYGIVVLEMITGRSPMIGVQDTEPGAESHERLATWVREKRRKAPEGASWVEQIVDPTLGSDYNVKQLEILAKVALDCVEEEKDVRPSMSQVVERLQSHEHGS

>Glycine_max-4055 (XP_003529974.2)

MNANMASSPTLIPFLLIFFLFHFHRSSSLPLSVENPEDDVIVSSPKGTFTAGFSPVGENAYSFAIWFSTQATTKTVVWMANRDQPVNGKRSTLSLLKTGNLVLTDAGQFDVWSTNTLSSKTLELHLFDTGNLVLREQSNQSAVLWQSFGFPTDTLLPGQIFTRFTKLVSSRSEGNHSSGFYNLYFDNDNVFRILYDGPQVSSVYWPDPWLVSDNVGFGNGRSTYNSSRVAVLDNLGEFSASDHFSFKTIDYGLLLQRRLTLDHDGNVRVYSRKNGEENWSITGQFKSQPCFIHGICGPNSICSHEQVIGRKCSCLEGYSWIDSQDWTLGCKPNFQPTCDNKTEYRFVPYYEVDFYGYDYGSSFSNYTYKQCEKLCSGLCECMGFQYSFARENGLFWCYPKRQLLNGHHSPGFTGQIFLRLPKNDVQENRVQNSDDLACSRNAEKVLERPYVKGKENGSVKFMLWFAIGLGGFEVLCIFMVWCFLFRSSNHLVSADQQGYVLAAATGFRRYTYSELKQATKGFSEEIGRGAGGTVYKGVLSDKRIAAIKKLHEFADQGESEFLTEVSIIGRLNHMNLIGMWGYCVEGKHRMLVYEYMENGSLAHNLPSNALDWSKRYNIAVGMAKGLAYLHEECLEWILHCDIKPQNILLDSDYQPKVADFGLSKPLNRNNVNNSSFSRIRGTRGYMAPEWVFNLQITSKVDVYSYGIVVLEMITGRSPMIGVQVTELGADQSHNERLATWVRERRRKAREGECWVEQIVDPTLGSDYDVEQMEILTTVALECVEEEKDVRPSMSQVVERLQSHDS

>Vitis_vinifera-4055 (CAN81830.1)

MDAPVLLLLLTLLLSSPLPSSTLDSLSQGSSLSVGKPEQVLISQSGIFSAGFYPVGDNAYCLAIWFTKPSYDGKHTAVWMANRNQPVNGNFSKLSLLESGDLILTDAGRFIVWTIKXVGISPVQLHLFNTGNLVLRTSDGVIQWQSFDSPTDTLLPHQPLTRNTRLVSSRTKTNFFSGFYKLYFDNNNVLSLVFDGRDASSIYWPPSWLVSWQAGRSAYNSSRTALLDNFGYFSSSDDFKFQSSDFGERVQRRLTLDIDGNLRLYSFEEXRNKWVVTWQAITLQCNIHGICGPNSICTYVPGSGSGRRCSCIPGYEMKNRTDRTYGCIPKFNLSCDSQKVGFLLLPHVEFYGYDYGYYPNYTLQMCEKLCLEICGCIGYQYSYNSDVYKCYPKRLLLNGYRSPSFVGHIYLKLPKASLLSYEKPVKEFMLDCSGNRSEQLVRSYAKAHENEVLKFILWFACAIGAVEMVCICMVWCFLMKAQQNTSTDPPGYILAATGFRKFTYTELKKATRGFSEEIGRGGGGVVYKGVLSDHRVAAIKQLSGANQGESEFLAEVSTIGRLNHMNLIEMWGYCFEGKHRLLVYEYMEHGSLAQNLTSNTLDWQKRFDIAVGTAKGLAYLHEECLEWVLHCDVKPQNILLDVNYQPKVADFGLSKLQNRGEINNSRLSRIRGTRGYMAPEWVLNLPITSKVDVYSYGIVVLEMVTGRRSASMAIHGTDGIGERQSLVAWVKGKMNGATAVASWMKEILDPSMEGEYDMGEMEILVAVALQCVELDKDERPTMSQVVETLLRPERGNNXHY

>Ricinus_communis-4055 (XP_002526221.1)

MATSLSTSLILIVFSLIISNLFSYSSSSTLKGPLNEGSSLSAENPDRVLISPSGIFSAGFYPVGDNAYSFAIWFNEPSCFNSCTVVWMANRDTPVNGRGSKLSLHKTSNLVLTDAGVSVTIWETNTFSVSSSSLYLYDTGNLALITIKERVILWQSFDLPTDTLLPLQLFTRDSLLVSSRSSTNYSSGFYKLSFDVSNILRLVYDGFDVSSSFWPDPWLLDREAGRSSYNSSRIAMLDSFAVDYGNLLQRRLTLDFDGNLRLYSRANESSTWEISWQIISQPCKIHGVCGPNSICSYNPGFGRKCSCLPGYKMKNLADWTLGCETEDKVSCDMNEATFLQFSHVEMYGYDFGYFLNYTLDMCEDVCLRRCDCRGFILKYVFQNHPENVPYCFPKTQMLNGYDSPSFRGDLYLKVPKTSHSDNSSIKQLSLDCPDGAVKQLERRYDKSDGSLLQKFLFAFASIIGIIEILATIFVRFLLIRSKEKSDQDYILAGTGFKRFSYSELKKATRDFSEEIGRGAAGTVYKGVLDGQRVAAIKRLNDASQGETEFLAEVSTVGKINHMNLIEMYGYCAEGKHRLLVYEYMEHGSLAENLSSKELDWRKRLEIAVGTAKGLAYLHEECLEWVLHCDVKPENILLDDDYRPKVSDFGLSRLLSRADPRNSFSRIRGTRGYIAPEWIFNMPITSKVDVYSYGMVALEMVTGKSPSLMGGQDSETGEELKHKRLVEWVNEKRNGASTKSWVKEIVDPIMGADYDAEKMENLIGVALKCVAEGKDSRPTMSQVVKMILQDEYDHWQ

>Setaria_italica-4055 (XP_004969488.1)

MPLLSRGSSLLVEEYKHTFLTSPDGDFSCGFYEVGRNAFSFSIWFTNTAEKTTVWSANPKYPVNGQGSKVLLNHDGNLVLTDVNGTVTWDSKTSSGKGTTVVLLDTGNLVIRNGNSEILWGSFSSPTDTLLPFQPLTKGTRLVSGYNSLYFDNDNVLRLMYDGPDISSIYWPSAEYTVYKNGRTNYNSSRIAVLDAEGYFQSSDLLKVKSSDWGTMTKRRLTLDYDGNLRMYSLNASRGSWIVSWEAVAKMCGVHGLCGKNGICEYWPSLHCSCPPGYEMTDPQNWNKGCRPNFSKICNGSYKAEDFEFIKLPHTDFYGFDQTYNVSISLEECKKACLEICSCAGFTYKTGPGLCYTKVLLFNGYRYPHFPGDNYIKLPKNLGISTSSDSRKSRLTCNRNIPEIVQGSSSMYGINSVDKNWTTYYVFAAILGALVLLFTGTSWWFLSSKQDIPKSMEAGYRMVTNQFRMFTHRELKEATGKFKEEIGRGGSGIVYRGVLEDKRVVAVKKLTNFSHSEEELWAEMSIIGRINHMNLVRMWGFCSEGQHKLLVYEYVENESLDRYLFGNVSSERLISWSQRFKIALGTARGLAYLHHECLEWVIHCDIKPENILLTRDFEAKIADFGLAKLSRRDRSSFNLTHMRGTMGYMAPEWALNSPINTKVDVYSYGVVLIEIVTGSRISSGVTVDGTEVEIKQFVQVLKQYTESGSVKDIVDQRLHGQFNPEQAMVMLKIATACLEERNSRPSMHDIVKALLACDDEDDHPAYSW

>Triticum_urartu-4055 (EMS64044.1)

MARFVISLIVLLPLISLLALRSCAAASVGHTLGAGSSLSVEDHERPFLVSPDATFSCGFLPAGEVDNAFYFSVWFTAATDRTAVWTANPGAPVNGRVSRVSFGADGKLALADANGTTVWDSKAASNNYFTVSLLDTGNLRVVDPSTGRPVWQSFDWPTDTLLPSQALTKDRKLVAGYYALYYDNDNVLRLLYDGPEIASIYWPNPGISVFDNGRTNYNSSRIGVLDDTGVFLSSDNLRVEASDLGASGVKRRLTIEQDGNVRMYSLDAAGGWTVTWAAVKQPCSVRGLCGKNAVCEYQPFLRCSCAPGYEMVDRRDWRKGCNPTFSLPTTTPTNCSTSGKRVAHTDFYGYDLGFNQSVTFQYCKTICLSMCSCAAFQYRTDGKGGCYPKGILFNGYTSPTPEGTIYLKLPSDLNAPATPPPAVLDCDQNAAIVPPAYADMYGTPSSGPNLSYLFWFAAVLGFLEALFIATAWWFLSGQESMPSSLMAGYRLVMGTQFRRFTYRELKKATGNFNEELGRGGSGVVYRGVLDKTTVVAVKELTNVVQGEEEFWAEMAVFGRINHINLVRIWGFCSEGKHKLLVYEYVENRSLDRHLFGEDIGKALAWRERFKIALGAAKGLAYLHHECLEWVIHCDVKPENILLTRDLDPKIADFGLSKLSGRKAVADGMQLSQMRGTAGYMAPEWVLGLPIDAKVDVYSYGIVLLEILMGSRITEQTTVDRAERLQMSQIVQALKQVVASGDVVSLVDSRLNGLFNPQQAMEMLKISLSCMEERSSRPTMDDIFKALIACDDEDEHPAYLS

>Aegilops_tauschii-4055 (EMT08973.1)

MPRLFFYPVALLPLLSTLLCSCASPWQSTISTGTSLQVDRGKVLLVSPDTTFSCGFYPSGNSTNAFYFSIWFTHATDKTVVWTANPCSPVNGQGSRISLNREGNLVLTDVNDSTAWESKTGWGKHTTVALLDTGNLVINDSTGKTVWQSFDLPTNTLLPTQHLTRANKLVSQSDSYHVLYFDNDNVLRLLYNGPDITSIYWPSPDYNALQNGRTRFNSSKIAVLDREGKFLSSDGFKMIASDSGLGIQRRITIDYDGNFRMYSLNASSGNWSITGQGVQQMCYVHGLCGKNGICEYSPAGRPRCTCPPGYKMVDPENWDRGCKPTFSIQCGQPQEDFQFVKVPHGDFYGFDLTSNKSISLGECRRICLESCMCISFTYKAGEGLCYTKNVLYNGQVYPYFLGDNYFKLPKSVSSTSPAANHPGITCSPERSKVMVVSADAYIKNSDHISWAYLYIFAAILGAVELFFIMTGWYVLFKMHNIPKSMEEGYKMITSQFRRFTYRELVEATGKFKEELGKGGNGVVYRGILGDKKVVAVKKLTDVRKGEEEFWAEVTLIGRINHMNLVRMYGFCSEGQHRLLVYEFVENESLDRYLFYGRGTERLLSWGQRFKIALGTARGLAYLHHECLEWIVHCDVKPENILLTREFEAKIADFGLSKLSERDSSSLNFTQMRGTTGYMAPEWVMNLPIDAKVDVYSFGVVLLEIVTGSRVSSGVTVDEDEMGLMQIPSGATEGGEGVGFMQFVQAVKQMLANGAELDIVDARLKGHFNHKQATVMVKIAISCLDERSKRPTMDQIARNLMECDDEDYHPAYF

>Brachypodium_distachyon-4055 (XP_003566977.1)

MIRLIYPILVSFLSTLLCSCASPWQTISTGTSLQVDHERVFLISPDTTFSCGFYPSGNDTNAFYFSVWFTHASDRAVVWTANPHFLVNGHRSRISLNKEGNLVLTDVDGSTTWESNTSWGKHTTAALLDSGNLVIKTSTDKIIWQSFDSPTHTLLPSQHLTRNNRLVSQSDYHVLYFDNDNVLRLLYNGPDITSIYWPSPDYNAIQNGRTRFNSTKVAVLDHEGNFLSSDGFKMIASDLGLGIQRRITIDYDGNFRMYSLNASNGNWTITGAAIQQMCYVHGLCGRNGICEYSLHLRCTCPPGYKMADPENWNKGCKPTFSIECGQPHEDFTFVKIPHGDFYGFDLTSNESISFKECMQICMKSCMCMSFTYKNGEGLCYTKNLLFNGQVYPYFPGDSYFKLPKISLTPKDDGISCRPKESKVMLVFANAYIKNPDNISWSYFYIFAAILGAVELLFIMTGWYVLFKAHNIPKSMEEGYKMITSQFRRFTYHELVEATGKFKEEVGKGGNGIVYRGILGDKKVVAVKKLTDVRKGEEEFWAEVTLIGKINHMNLVRMYGFCSEGHHRLLVYEFVENESLDKYLFYDSNTERLLSWSQRFQIALGAARGLAYLHHECLEWIVHCDVKPENILLTRDFQAKIADFGLSKLSKRDSSNFNFTYMRGTTGYMAPEWVLNLPIDAKVDVYSYGVVLLEIVTGSRVSSGVTVGEEVMDLMQISSGVSIGEEEMDLLGIVDARLKGHFNHEQATTMLKIAVSCLDERSKRPTMDQITKDLMVYNDEDFHPAYF

>Hordeum_vulgare-4055 (BAK06806.1)

MAATRAAYIFTTSILFLLLLLLIPVALAKDHRTHAASYLARGSSVSIEDGTIATTTTILASPNGLFGCGFYKVATNAFVLSIWFTGSSARTVAWTANRDAPVNGRGSRLAFRKDGGLALLDYGGMPVWSTNTTATGASRAELLDSGSLVVLDPDGRSLWTSFDSPTDTLLPSQPMTRNIKLVSASARGLLYSGFYTLYFDSDNVLRLIYNGPEINSIYWPDPFNKPWGNGRTTYNSSRHAVLEQSGQFVSSDNFTFEASDLGDMVMRRLTLDYDGNLRLYSLNQTSGHWSVSWMAFRRVCNIHGLCGQNSICKYSYMPKLECSCVEGFEVVDASDWSKGCRRKANMTARKDKQRKQEASINATQIFSFRKLAKTDFYGYDLAYAAPVSFLTCKLMCLDNVDCQAFGYRQGEGKCYPKVILFNGKNFPRPYNDIYLKIPKGASSLELASTANHTCRVHEKEANASSEMFKDGTSKFKFGYFLSSALTLLFVEVILIITGCWVVHKWERRPEIIDEGYMIISSQFRIFSYKELQKATNCFQEELGSGGSGAVYKGVLDDERKVAVKKLNDVIQGEQEFRSEISVIGRIYHMNLVRIWGFCVEKTHRLLVSEFIENGSLATILFDHQSNSPVLQWGQRYNIALGVAKGLAYLHHECLEWIVHCDVKPENILLDRDFQPKIADFGLMKLQQRGSSAQMLSKVHGTRGYIAPEWALNLPINGKADVYSYGVVLIELVKGVRLSRWVVEGEEEVEMADICSIEILKEKLASEDQSWLLEFVDHRLDGDFNHSEALMMLKIAVSCVEEERSRRPNMSHVVETLLSLVE

>Zea_mays-4055 (DAA58196.1)

MAALLYLHPYLAVLPLLISPICSGASPWRTMTTGSHMRGEDHDKVTLLSPDATFSCGFHEVGTNALTFSIWYTPSASASATERTVVWTANPYSAERGQHSPVNKYGSRLSLNRDGNLVLTDTNGSTVWETKTSSGRHTTAALLDSGNLVIRDSSSGSNKVVWQSFRSPTDTLLPGQELTKDTRLVSGYHHLYFDNDNVLRMLYDGPEITSIYWPSPDYNALKNGRNRFNSTRVAVLDDLGTFVSSDGFRIEASDSGPGVKRRITIGYDGNFRMYSLNASTGAWRVTGQAVIQMCYVHGLCGRNGLCDYLGGLRCRCPPDYEMVDPTNWNRGCKPMFLTTDDGKEFTFVEQPHADYYGFDLSSNESVPFEACRDMCLNSSACLSFTYKGGDGWCYTKGLLYNGQVFPYFPGDSYMKVPKSFNSSAAYSSISNQKEALTCGPAGSAELMLGPASMYGTKKDNINWTYLYVFAAVLGALEMLVIATGWYLFFNKHSIPKSMEDGYKLVTNPFRRFTYRELAEATGKFKEELGRGGAGVVYRGVLEDKKVVAVKKLTDVRQGEEEFWAEVTLIGRINHINLVRMWGFCSEGTKRLLVYEYVENESLDKYLFGERSAESLLGWSQRYKIALGTARGLAYLHHECLEWVVHCDVKPENILLTRDFDAKIADFGLAKLAKQGSTSLNFTHMRGTMGYMAPEWALNSPISAKVDVYSYGVVLLEIVTGIRASSGIVLDERQIDFRQFVQEAKHILSTGSVSDIVDDRLQGHFHADQAVAMVKIAFSCLEERRKRPTMDEIVKVLMSCGDDDDYHPAYSY

>Arabidopsis_lyrata-4055 (XP_002891094.1)

MAEKTPFLKLLPLLLLLLLHFPLSSSTIPLGSVIFASGSNQNWPSPNSTFSVSFVPASSPNSFLAAVSFAGNVPIWSAGTVDSRGSLRLLTSGSLRLTNGSGTTIWDSGTDRLGVTSGSIEDSGEFILRNNRSIPVWSSFDNPTDTIVQSQNFTVGKILRSGLYSFQLETSGNLTLRWNTSTIYWNLGLNSSISSNLSSPSLGLVLRTNGVVSIFDSNLRGGVDTVYSGDYGDSDTFRFLKLDDGNLRIYSSASRNSGPVNAHWSAVDQCLVYGYCGNFGICSYNDTNPICSCPSGNFDFVNVNDRRKGCRRKVELSDCSGNTTMLDLPHTRLFTYENDPNSEIFFAGSSPCRANCLSSVTCLASVSMSDGSGNCWQKQPGSFFTGYQRPSVPSTSYVKVCAPVVSNPPLIATKVDSNNSKVHLWIVAVAVMAGLLGLVAVEVGLWWCCCRKNPRFGTLSSHYTLLEYASGAPVQFTYKELQRCTKSFKEKLGAGGFGTVYKGVLTNRTVVAVKQLEGIEQGEKQFRMEVATISSTHHLNLVRLIGFCSQGRHRLLVYEFMRNGSLDNFLFTTDSGKFLTWEYRFSIALGTAKGITYLHEECRDCIVHCDIKPENILVDDNYAAKVSDFGLAKLLNPKDNRYNMSSVRGTRGYLAPEWLANLPITSKSDVYSYGMVLLELVSGKRNFDVSEKTNHKKFSIWAYEEFQKGNTEAILDTRLGEDQTVDMEQVMRMVKTSFWCIQEQPLQRPTMGKVVQMLEGITEIKNPPCPKTISEVSVSGNSMSTSRASMLVASGPTRSSSSSATRSFQTMGITSSGPASTRISEGSMLGS

>Eutrema_salsugineum-4055 (XP_006394665.1)

MGFTRLKSTNRFLLSIWFAKLPGDPAIVWSPNRNSPVSKEAVLELEATGNLVLSDQTTIVWTSNTSNHGVKSAVMSESGNFLLLGTEVTAAQAIWQSFSQPADTLLPNQQLTVSLELTSNPSPSRHGHYSLKMLQQHTSLSLGLTYNINLDPHANYSYWSGPEISNVTGDVTAILDDTGSFKIVYGESSTGAVYVYKNPADENRNYNNSSNSRLTRKPILRRLVLEDNGNLRLYQWDNDMNGSSQWVPEWAAVSNPCDIAGTCGNGVCNLDRTKKHADCFCLPGSVKLPDQENAKLCSDNSSLVQECESNINRNGTFKISTVQETNYYFSERSVIENYSDIGNVRKCGDMCLSNCKCVASVYGLDDEKPYCWILKSLNFGGFRDPGSTLFVKTRANESYPSNSNHHGNDSKSHGSHGLRQKVLVIPIVVGMLVLVALLGMLLYYNVDRKRTLKKAAKNSLILCDSPVSFTYRDLQNSTNNFSQLLGSGGFGTVYKGIIAGETLVAVKRLDRVLSHGEREFITEVNTIGSMHHMNLVRLCGYCSEDSHRLLVYEYMINGSLDKWIFSSSDRTARLLDWQTRFEIAVATAQGIAYFHEQCRNRIIHCDIKPENILLDENFCPKVSDFGLAKMMGREHSQVVTMIRGTRGYLAPEWVSNRPITVKADVYSYGMLLLEIVGGRRNLDMSFDAEDFFYPGWAYKELTNGTALKVVDRRLQGVAEEEEVVKALKVAFWCIQDEVSVRPSMGEVVKLLEGSSDEINLPPMPQTILELIEEGLEDVYRAMRREINNQLSSFTVNTITTSRSYLSSSRSHATCSYSSMSPR
