# Supplementary figures and images for: A novel bioinformatics pipeline to discover genes related to arbuscular mycorrhizal symbiosis based on their evolutionary conservation pattern among higher plants
Source: BMC Plant Biol. 2014 Dec 3;14:333. doi: 10.1186/s12870-014-0333-0 (PMC4274732; doi:10.1186/s12870-014-0333-0)

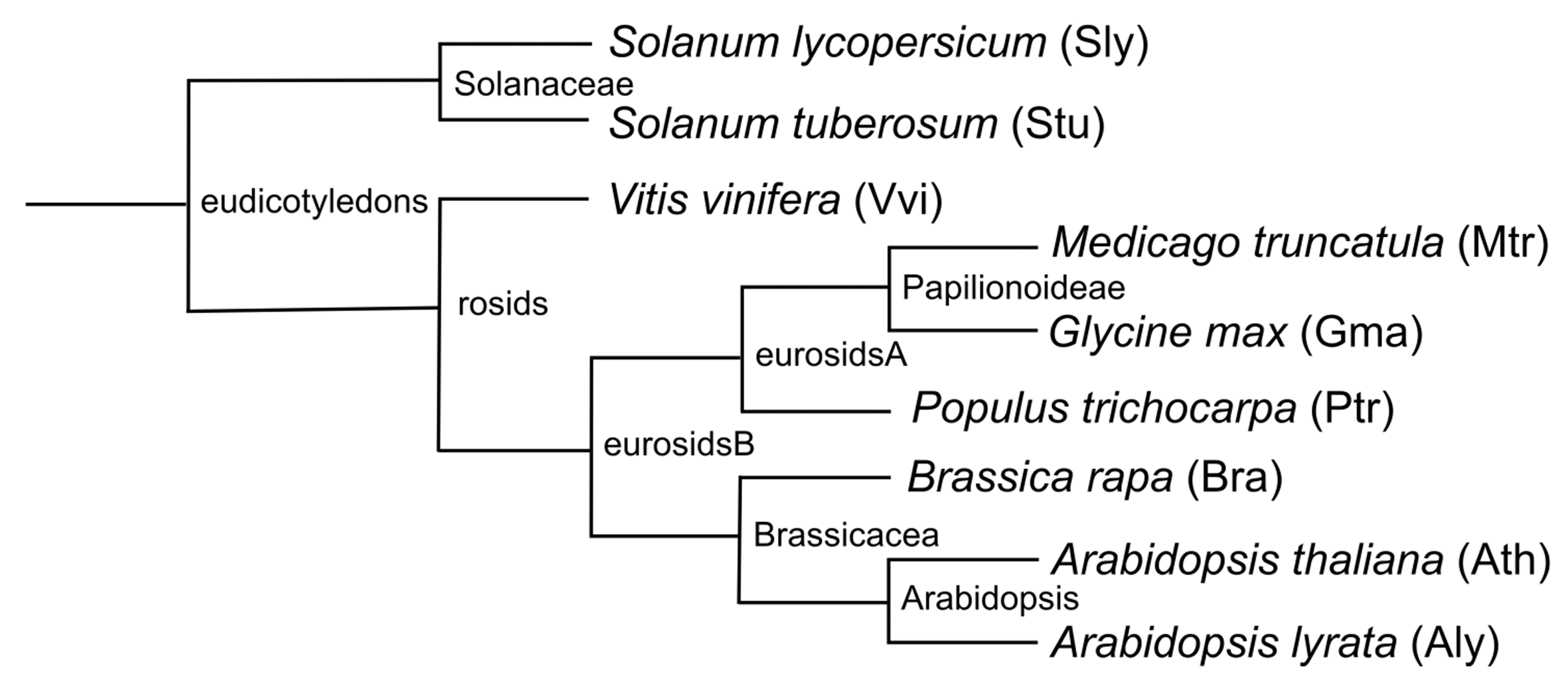

Supplement: Additional file 2: Figure S1. — Conceptual phylogenetic tree of plant species for clustering with Hieranoid. This qualitative phylogenetic tree was used for pair-wise comparison of protein sequences in a step-wise scheme for the construction of protein clusters by Hieranoid. Distances between nodes are irrelevant for this procedure and were set randomly at 4. [file 12870_2014_333_MOESM2_ESM.png]

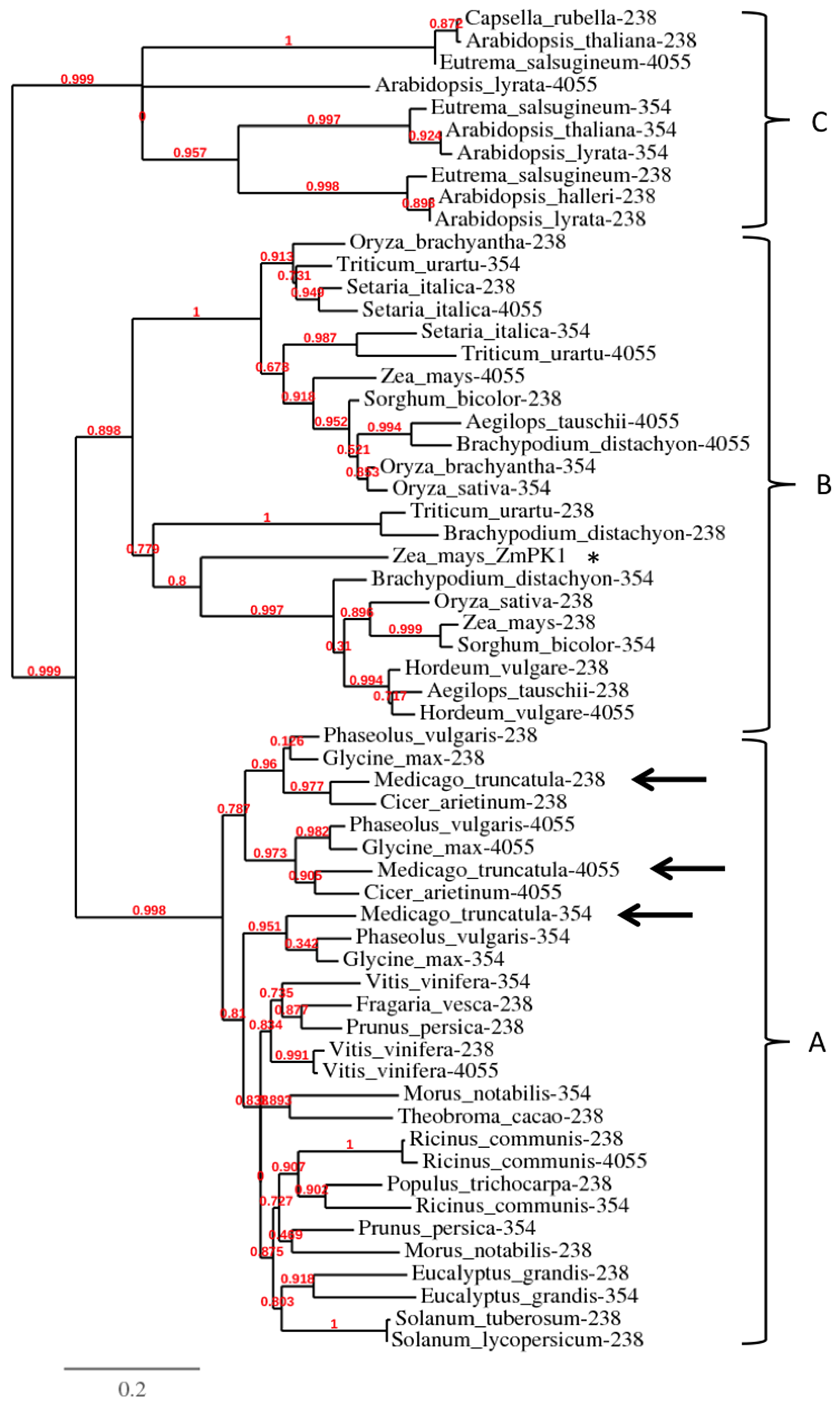

Supplement: Additional file 11: Figure S5. — Phylogenetic tree of receptor-like protein kinases of diverse plant species. An unrooted phylogenetic tree was generated with the three M. truncatula sequences identified in Additional file 14: Table S6b (Task3_238, Task3_354, and Task3_4055; see arrows), together with the closest homologues from diverse plant species as indicated. Maize lectine-like protein kinase (ZmPK1; asterisk) is included for reference. [file 12870_2014_333_MOESM11_ESM.png]

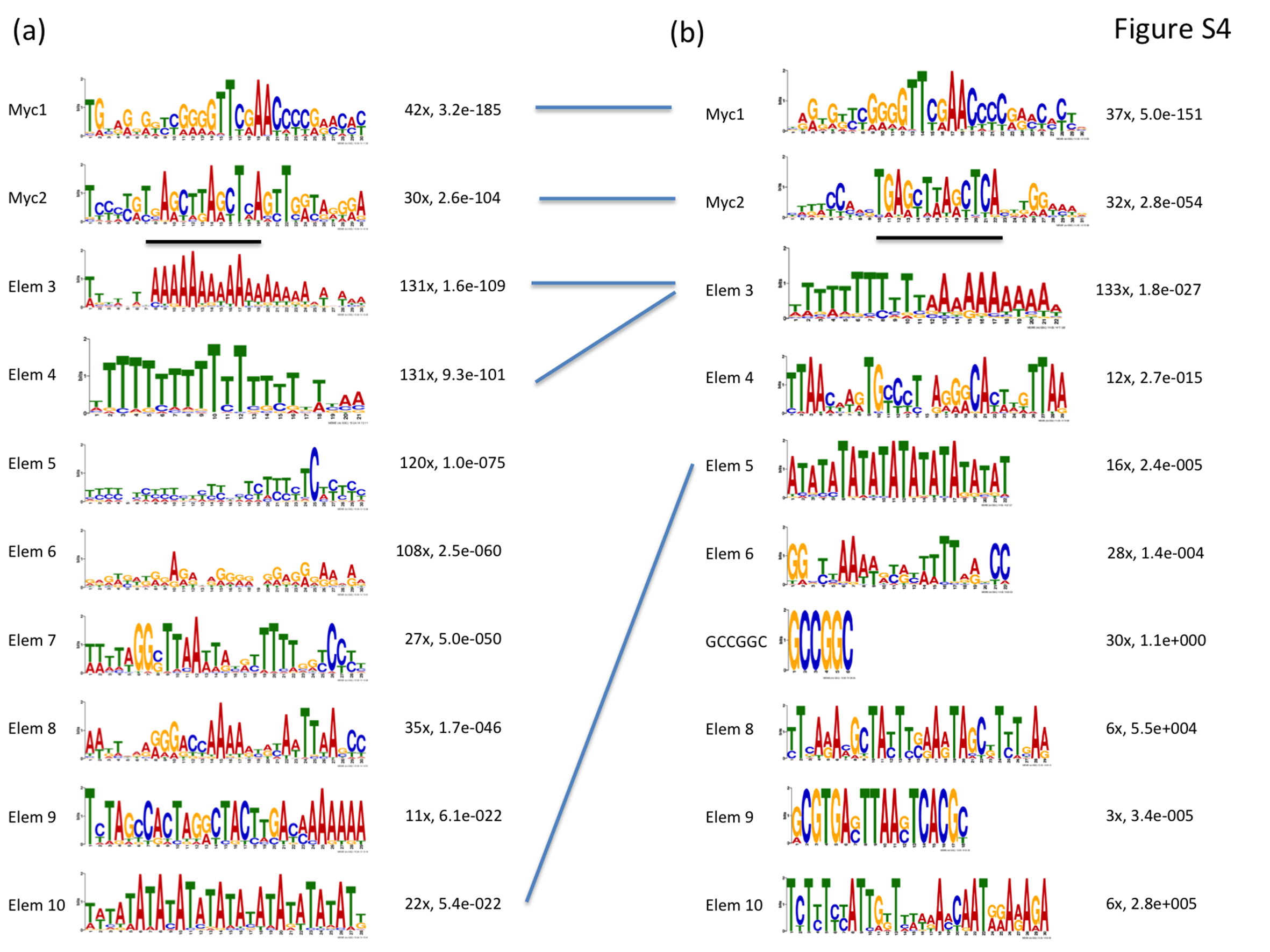

Supplement: Additional file 15: Figure S4. — Potential cis-regulatory elements predicted from 190 AM-induced promoters. Potential regulatory elements were predicted by MEME for any elements between 6-30 nt (a), and for palindromic elements between 6-30 nt (b). Numbers behind the sequences represent the number of occurrences among the 190 promoters analyzed, and an E-value provided by MEME. Blue lines link related sequences that came up with both searches; black lines underline common sequence elements between related predictions. [file 12870_2014_333_MOESM15_ESM.png]

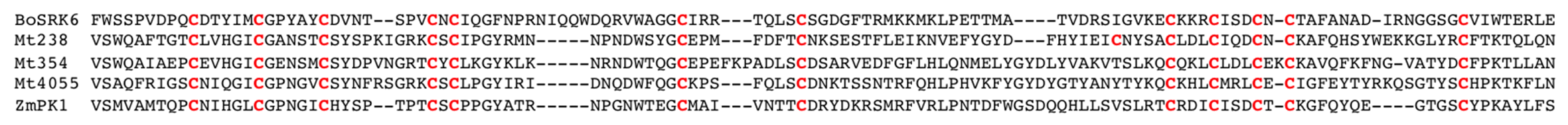

Supplement: Additional file 18: Figure S6. — Conservation of the C-rich region of S_RLK homologues. Alignement of the C-rich region of Brassica oleracea S_RLK6 (BoSRK6), ZmPK1, and the three M. truncatula proteins identified in this study (Mt238, Mt354, and Mt4055). [file 12870_2014_333_MOESM18_ESM.png]
