# Supplementary material for: A novel bioinformatics pipeline to discover genes related to arbuscular mycorrhizal symbiosis based on their evolutionary conservation pattern among higher plants
Source: BMC Plant Biol. 2014 Dec 3;14:333. doi: 10.1186/s12870-014-0333-0 (PMC4274732; doi:10.1186/s12870-014-0333-0)
Supplement: Additional file 6: File S4. — References cited in Additional file 5: Table S1. [file 12870_2014_333_MOESM6_ESM.docx]

**Favte et al. - Supplemental File S4**

**References in Supplemental Tabe S1**

**Ané, J.-M., Kiss, G.B., Riely, B.K., Penmetsa, R.V., Oldroyd, G.E.D., Ayax, C., Lévy, J., Debellé, F., Baek, J.M., Kalo, P., Rosenberg, C., Roe, B.A., Long, S.R., Dénarié, J., and Cook, D.R.** (2004). Medicago truncatula DMI1 required for bacterial and fungal symbioses in legumes. Science **303,** 1364-1367.

**Arrighi, J.F., Barre, A., Ben Amor, B., Bersoult, A., Soriano, L.C., Mirabella, R., de Carvalho-Niebel, F., Journet, E.P., Gherardi, M., Huguet, T., Geurts, R., Denarie, J., Rouge, P., and Gough, C.** (2006). The Medicago truncatula lysine motif-receptor-like kinase gene family includes NFP and new nodule-expressed genes. Plant Physiol. **142,** 265-279.

**Breuillin, F., Schramm, J., Hajirezaei, M., Ahkami, A., Favre, P., Druege, U., Hause, B., M., B., Kretzschmar, T., Bossolini, E., Kuhlemeier, C., Martinoia, E., Franken, P., Scholz, U., and Reinhardt, D.** (2010). Phosphate systemically inhibits development of arbuscular mycorrhiza in *Petunia hybrida* and represses genes involved in mycorrhizal functioning. Plant J. **64,** 1002-1017.

**Endre, G., Kereszt, A., Kevei, Z., Mihacea, S., Kaló, P., and Kiss, G.B.** (2002). A receptor kinase gene regulating symbiotic nodule development. Nature **417,** 962-966.

**Feddermann, N., Duvvuru Muni, R.R., Zeier, T., Stuurman, J., Ercolin, F., Schorderet, M., and Reinhardt, D.** (2010). The *PAM1* gene of petunia, required for intracellular accommodation and morphogenesis of arbuscular mycorrhizal fungi, encodes a homologue of VAPYRIN. Plant J. **64,** 470-481.

**Gobbato, E., Marsh, J.F., Vernie, T., Wang, E., Maillet, F., Kim, J., Miller, J.B., Sun, J., Bano, S.A., Ratet, P., Mysore, K.S., Dénarie, J., Schultze, M., and Oldroyd, G.E.D.** (2012). A GRAS-type transcription factor with a specific function in mycorrhizal signaling. Current Biology **22,** 2236-2241.

**Groth, M., Takeda, N., Perry, J., Uchida, H., Draexl, S., Brachmann, A., Sato, S., Tabata, S., Kawaguchi, M., Wang, T.L., and M., P.** (2010). NENA, a Lotus japonicus homolog of Sec13, is required for rhizodermal infection by arbuscular mycorrhiza fungi and rhizobia but dispensable for cortical endosymbiotic development. Plant Cell **22,** 2509-2526.

**Harrison, M.J., Dewbre, G.R., and Liu, J.Y.** (2002). A phosphate transporter from *Medicago truncatula* involved in the acquisiton of phosphate released by arbuscular mycorrhizal fungi. Plant Cell **14,** 2413-2429.

**Imaizumi-Anraku, H., Takeda, N., Charpentier, M., Perry, J., Miwa, H., Umehara, Y., Kouchi, H., Murakami, Y., Mulder, L., Vickers, K., Pike, J., Downie, J.A., Wang, T., Sato, S., Asamizu, E., Tabata, S., Yoshikawa, M., Murooka, Y., Wu, G.J., Kawaguchi, M., Kawasaki, S., Parniske, M., and Hayashi, M.** (2005). Plastid proteins crucial for symbiotic fungal and bacterial entry into plant roots. Nature **433,** 527-531.

**Kalo, P., Gleason, C., Edwards, A., Marsh, J., Mitra, R.M., Hirsch, S., Jakab, J., Sims, S., Long, S.R., Rogers, J., Kiss, G.B., Downie, J.A., and Oldroyd, G.E.D.** (2005). Nodulation signaling in legumes requires NSP2, a member of the GRAS family of transcriptional regulators. Science **308,** 1786-1789.

**Kanamori, N., Madsen, L.H., Radutoiu, S., Frantescu, M., Quistgaard, E.M.H., Miwa, H., Downie, J.A., James, E.K., Felle, H.H., Haaning, L.L., Jensen, T.H., Sato, S., Nakamura, Y., Tabata, S., Sandal, N., and Stougaard, J.** (2006). A nucleoporin is required for induction of Ca2+ spiking in legume nodule development and essential for rhizobial and fungal symbiosis. Proc. Natl. Acad. Sci. U. S. A. **103,** 359-364.

**Lévy, J., Bres, C., Geurts, R., Chalhoub, B., Kulikova, O., Duc, G., Journet, E.P., Ané, J.M., Lauber, E., Bisseling, T., Denarie, J., Rosenberg, C., and Debellé, F.** (2004). A putative Ca2+ and calmodulin-dependent protein kinase required for bacterial and fungal symbioses. Science **303,** 1361-1364.

**Liu, J.Y., Versaw, W.K., Pumplin, N., Gomez, S.K., Blaylock, L.A., and Harrison, M.J.** (2008). Closely related members of the Medicago truncatula PHT1 phosphate transporter gene family encode phosphate transporters with distinct biochemical activities. J. Biol. Chem. **283,** 24673-24681.

**Messinese, E., Mun, J.H., Yeun, L.H., Jayaraman, D., Rouge, P., Barre, A., Lougnon, G., Schornack, S., Bono, J.J., Cook, D.R., and Ane, J.M.** (2007). A novel nuclear protein interacts with the symbiotic DMI3 calcium- and calmodulin-dependent protein kinase of Medicago truncatula. Mol. Plant-Microbe Interact. **20,** 912-921.

**Mitra, R.M., Gleason, C.A., Edwards, A., Hadfield, J., Downie, J.A., Oldroyd, G.E.D., and Long, S.R.** (2004). A Ca2+/calmodulin-dependent protein kinase required for symbiotic nodule development: Gene identification by transcript-based cloning. Proc. Natl. Acad. Sci. U. S. A. **101,** 4701-4705.

**Murray, J.D., Duvvuru Muni, R., Torres-Jerez, I., Tang, Y., Allen, S., Andriankaja, M., Li, G., Laxmi, A., Cheng, X., Wen, J., Vaughan, D., Schultz, M., Sun, J., Charpentier, M., Oldroyd, G., Tadege, M., Ratet, P., Mysore, K.S., Chen, R., and Udvardi, M.** (2011). *Vapyrin*, a gene essential for intracellular progression of arbuscular mycorrhizal symbiosis, is also essential for infection by rhizobia in the nodule symbiosis of *Medicago truncatula*. Plant J. **65,** 244-252.

**Peiter, E., Sun, J., Heckmann, A.B., Venkateshwaran, M., Riely, B.K., Otegui, M.S., Edwards, A., Freshour, G., Hahn, M.G., Cook, D.R., Sanders, D., Oldroyd, G.E.D., Downie, J.A., and Ane, J.M.** (2007). The Medicago truncatula DMI1 protein modulates cytosolic calcium signaling. Plant Physiol. **145,** 192-203.

**Pumplin, N., Mondo, S.J., Topp, S., Starker, C.G., Gantt, J.S., and Harrison, M.J.** (2010). *Medicago truncatula* Vapyrin is a novel protein required for arbuscular mycorrhizal symbiosis. Plant J. **61,** 482-494.

**Radutoiu, S., Madsen, L.H., Madsen, E.B., Felle, H.H., Umehara, Y., Gronlund, M., Sato, S., Nakamura, Y., Tabata, S., Sandal, N., and Stougaard, J.** (2003). Plant recognition of symbiotic bacteria requires two LysM receptor-like kinases. Nature **425,** 585-592.

**Riely, B.K., Lougnon, G., Ané, J.M., and Cook, D.R.** (2007). The symbiotic ion channel homolog DMI1 is localized in the nuclear membrane of *Medicago truncatula* roots. Plant J. **49,** 208-216.

**Saito, K., Yoshikawa, M., Yano, K., Miwa, H., Uchida, H., Asamizu, E., Sato, S., Tabata, S., Imaizumi-Anraku, H., Umehara, Y., Kouchi, H., Murooka, Y., Szczyglowski, K., Downie, J.A., Parniske, M., Hayashi, M., and Kawaguchi, M.** (2007). NUCLEOPORIN85 is required for calcium spiking, fungal and bacterial symbioses, and seed production in Lotus japonicus. Plant Cell **19,** 610-624.

**Salzer, P., Bonanomi, A., Beyer, K., Vögeli-Lange, R., Aeschbacher, R.A., Lange, J., Wiemken, A., Kim, D., Cook, D.R., and Boller, T.** (2000). Differential expression of eight chitinase genes in *Medicago truncatula* roots during mycorrhiza formation, nodulation, and pathogen infection. Mol. Plant-Microbe Interact. **13,** 763-777.

**Smit, P., Raedts, J., Portyanko, V., Debelle, F., Gough, C., Bisseling, T., and Geurts, R.** (2005). NSP1 of the GRAS protein family is essential for rhizobial Nod factor-induced transcription. Science **308,** 1789-1791.

**Stracke, S., Kistner, C., Yoshida, S., Mulder, L., Sato, S., Kaneko, T., Tabata, S., Sandal, N., Stougaard, J., Szczyglowski, K., and Parniske, M.** (2002). A plant receptor-like kinase required for both bacterial and fungal symbiosis. Nature **417,** 959-962.

**Wang, E.T., Schornack, S., Marsh, J.F., Gobbato, E., Schwessinger, B., Eastmond, P., Schultze, M., Kamoun, S., and Oldroyd, G.E.D.** (2012). A common signaling process that promotes mycorrhizal and oomycete colonization of plants. Current Biology **22,** 2242-2246.

**Yano, K., Yoshida, S., Muller, J., Singh, S., Banba, M., Vickers, K., Markmann, K., White, C., Schuller, B., Sato, S., Asamizu, E., Tabata, S., Murooka, Y., Perry, J., Wang, T.L., Kawaguchi, M., Imaizumi-Anraku, H., Hayashi, M., and Parniske, M.** (2008). CYCLOPS, a mediator of symbiotic intracellular accommodation. Proc. Natl. Acad. Sci. U. S. A. **105,** 20540-20545.

**Zhang, Q., Blaylock, L.A., and Harrison, M.J.** (2010). Two *Medicago truncatula* half-ABC transporters are essential for arbuscule development in arbuscular mycorrhizal symbiosis. Plant Cell **22,** 1483-1497.
