# Supplementary material for: A novel bioinformatics pipeline to discover genes related to arbuscular mycorrhizal symbiosis based on their evolutionary conservation pattern among higher plants
Source: BMC Plant Biol. 2014 Dec 3;14:333. doi: 10.1186/s12870-014-0333-0 (PMC4274732; doi:10.1186/s12870-014-0333-0)
Supplement: Additional file 17: File S5. — Results from MEME analysis for palindromic regulatory elements in AM-induced promoters. (compare with Figure 9 and Additional file 15: Figure S4). [file 12870_2014_333_MOESM17_ESM.pdf]

For further information on how to interpret these results or to get a copy of the MEME software please access http://meme.nbcr.net.

If you use MEME in your research, please cite the following paper:
Timothy L. Bailey and Charles Elkan, "Fitting a mixture model by expectation maximization to discover motifs in biopolymers", *Proceedings of the Second International Conference on Intelligent Systems for Molecular Biology*, pp. 28-36, AAAI Press, Menlo Park, California, 1994.

DISCOVERED MOTIFS | BLOCK DIAGRAMS OF MOTIFS | PROGRAM INFORMATION | EXPLANATION

DISCOVERED MOTIFS

Motif Overview

Motif 1

- 5.0e-151
- 37 sites

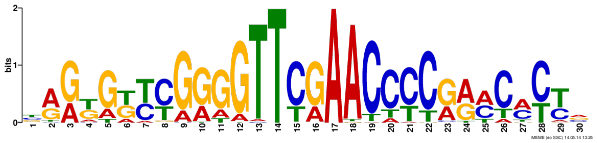
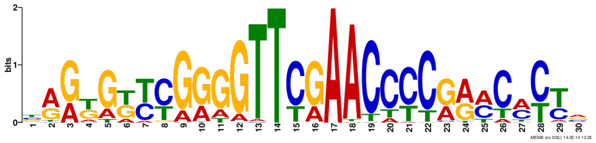

Motif 2

- 2.8e-054
- 32 sites

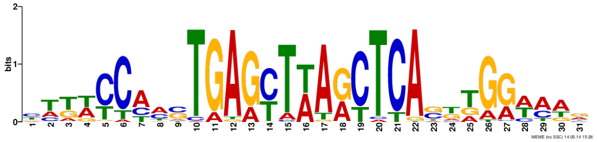
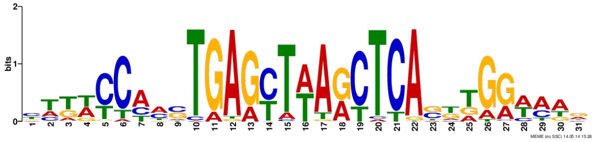

Motif 3

- 1.8e-027
- 133 sites

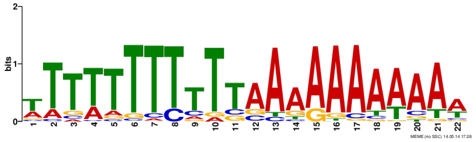
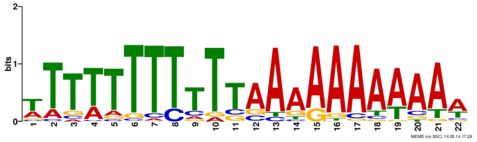

Motif 4

- 2.7e-015
- 12 sites

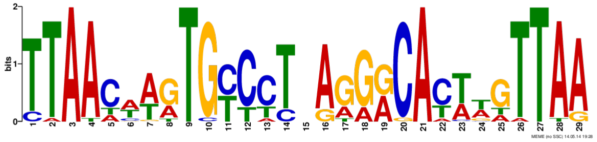
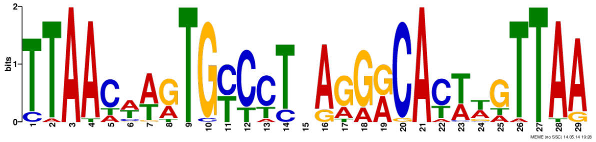

Motif 5	• 2.4e-005<br>• 16 sites	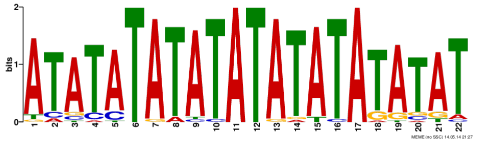	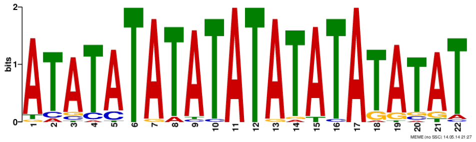
Motif 6	• 1.4e-004<br>• 28 sites	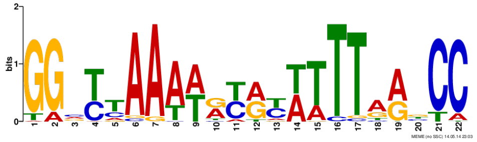	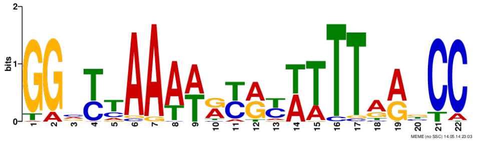
Motif 7	• 1.1e+000<br>• 30 sites	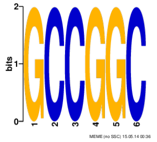	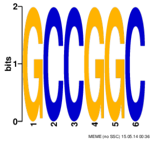
Motif 8	• 5.5e+004<br>• 6 sites	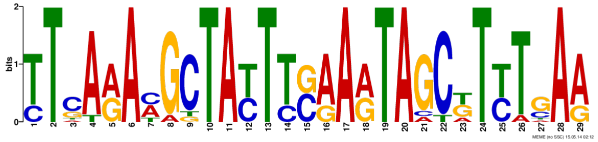	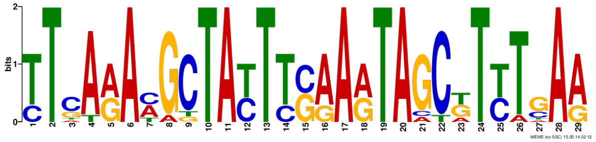
Motif 9	• 3.4e+005<br>• 3 sites	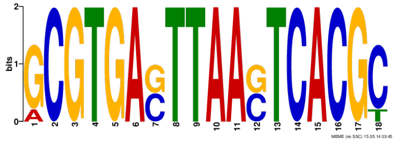	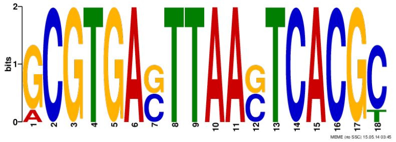
Motif 10	• 2.8e+005<br>• 6 sites	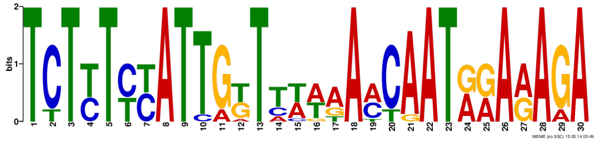	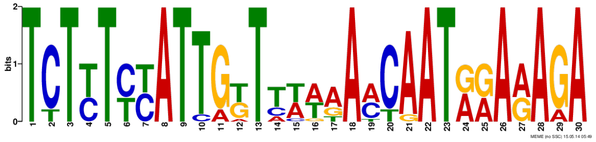

Further Analysis

Submit all motifs to MAST [?] FIMO [?] GOMO [?] BLOCKS [?] Mouse-over buttons for more information.

Motif 1

Next Top

Summary ?

E-value	5.0e-151
Width	30
Sites	37

show more

Sequence Logo ?

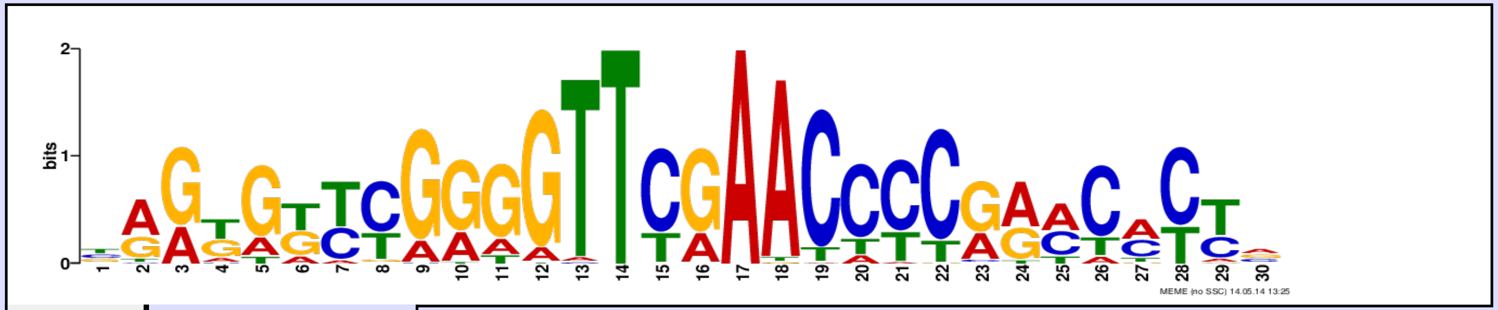

Standard | Reverse Complement

Download LOGO ? Orientation: standard SSC: off Format: web (png) Width: 30 cm Height: 7.5 cm Download

Regular expression ?

[TCG][AG][GA][TG]G[TG][TC][CT]G[GA]GGTT[CT][GA]AACC[CT]C[GA][AG][AC]C[AC][CT][TC][AGC]

Further Analysis ?

Submit this motif to MAST ? FIMO ? TOMTOM ? GOMO ? BLOCKS ? Mouse-over buttons for more information.

Data Formats ?

View the motif in ○ PSPM Format ? ○ PSSM Format ? ○ BLOCKS Format ? ○ FASTA Format ? ○ Raw Format ? or ● Hide

Sites ?

Click on any row to highlight sequence in all motifs.

Name	Strand	Start	*p*-value	Sites		
MTR_5g098020	+	792	4.28e-20	TGGAGAAGTG	GGGTGTCCGGGGTTCGAACCCCGGCCCCTG	CATATAACAT
MTR_4g091020	+	850	1.60e-19	TTGTTATATG	CAGGGGCCGGGGTTCGAACCCCGAACACCC	CACTTATTCA
MTR_7g012070	+	504	1.10e-17	CGAAGAAGTG	GGGTGTCTGGGGTTCGAACCCCGACCCCTA	CATATAATAT
MTR_2g101560	+	1486	7.59e-17	CATTTGTATG	CAGGGGCTGAGGTTCGAACCCCGAACACCC	CACTTATTCA
MTR_3g072170	+	1105	1.72e-16	TGAATAAGTG	GGGAGTTCGAGGTTCGAACCCCGACCCCTG	CATATATTAT
MTR_5g027000	+	1350	6.97e-16	ATATTATATG	CAGGGGCTGGGGTTCAAACCCCAGACACTC	AACTTCTCCA
MTR_4g069810	-	1602	1.26e-15	GTGAAGAAGT	AAGTGTCCGGGATTCGAACCCCGGCCCCTG	CATAAATTAT
MTR_7g020870	-	1161	2.57e-15	CATATATATG	TAAGGTACGGGGTTCGAACCCCGACCACCA	CAAAAAAAAA
MTR_3g084200	+	1182	2.57e-15	ATAAAATATG	CAAGGTCCGGGGTTCAAACCTCGGCCACTA	CCCAAAAAAG
MTR_6g012630	-	313	3.40e-15	AGAAATTGAG	CAAGGTCCGGGGTTCAAACCCCGGCTACCA	CAAAAAAAAA
MTR_7g024890	+	1480	2.83e-14	ATAATATATA	CAAAGTCCGAGGTTCAAACCCCGGCCACCA	ACAAAAAAAA
MTR_4g077550	-	1310	4.11e-14	ATAAAATATG	CAATGTCTGAGGTTCGAACCTCGACCACCA	CCAAAAAGAA
MTR_3g116430	+	236	4.11e-14	ATAATTTATG	TAGGAGCTGGGGTTTAAACCCCGGACACCC	CATTTATTCA
MTR_4g130270	-	326	2.75e-13	TGAATAAGTG	AGGTGTTCGAGGTTTGAACCCTGACCCCTA	CATATAACAA
MTR_4g129010	+	222	7.50e-13	TGAATAAGTG	AAATGTTCGAGGTTCGAACTTCGACCCCTG	CATATATTAT
MTR_1g101370	-	990	1.43e-12	AAGCCGAATG	CCATGATCGGGGTTCGAACCCCGATACCTC	CACTTCTGTG
MTR_3g082250	+	221	2.41e-12	ATGTTATATA	CAGGAGTCAGAGTTCAAACTCCGGACACCC	CACTTATTCA
MTR_1g105120	+	860	2.41e-12	ATAATATATG	CAGCGGTCGGGGTTCAAACCACAGACATCC	CACTTTTTCA
MTR_1g098300	+	343	6.60e-12	ATGATTTATG	CAGGGGCAGGGGCTCGAACCCTGACACCCC	ACTTCTCCAC
MTR_4g081190	-	165	6.60e-12	AGGCCGGATG	TCATGATTGGGGTTCGAACCCCGGTACCTC	CACTTGTGTG
MTR_4g104020	+	167	7.28e-12	ATAATATATG	CAAGGTTCATGGTTCGAATCCCGGACACCA	TCAAATAGAG
MTR_1g040500	-	1769	1.30e-11	TTTTTTTTTT	TGGTGGTCAGGATTTGAACTCCGAACCTTA	TATATATGAT
MTR_7g085310	-	668	1.73e-11	TTGTTATATT	CAGAGGTCGATGTTTGAACTTCGAACACCC	TACTTATTAT
MTR_8g062790	-	797	3.62e-11	ATAATATATA	TAGATGTTGGGGTTCGAACCTCAGATATCC	CACTTCTTCA
MTR_5g020810	+	612	9.62e-11	ATAATGGTTG	GTGTATCTGAGGTTTGAATCTCGACCCCTA	CATATATTAT
MTR_8g022270	-	1393	1.75e-10	TTTTTTTCTT	GTGATGACGGGGTTTGAACCCCCAACCTTG	CATATATTAT

Name	Strand	Start	*p*-value	Sites
MTR_1g061530	+	1260	2.65e-10	CGTTATATAA TAAGGGTTGGAGTTTTAACCTCGAACATCC AATTCTTTTT
MTR_1g061540	-	1291	3.99e-10	ATAATATATA TAAGATCGAGTGTTCGAACTCCAGACACCA CCAATAAAAT
MTR_5g005950	+	1093	8.13e-10	TTTTTTTTTG TGGCGACTGGGGTTTGAACATCAAATCTTA CATATATTAT
MTR_4g047610	-	1513	2.71e-09	TATACAAGTG AAAGAGTTGGAGTTCAAACCCTAATCACAG CGTCCGATCT
MTR_2g096180	-	1255	3.37e-09	TATTATATGG TTATGGTTAGTGTTTGAACTCCGAATCTTG CATATTTTTA
MTR_4g015650	-	686	4.48e-09	TGAATAAATA GGGTATATGAGGTTTAAACCTCAACTTCTA CATGTAATAA
MTR_2g017750	-	126	4.81e-09	CTGACAAGTG GAGTATACAAGGTTCGAACTCCGATTTTTA TATATATTAG
MTR_7g088790	+	1285	1.03e-08	TAATAAATCT GGATGATTAGGTTTCGAACACTAACCTCCA TGAAAGATCT
MTR_6g069050	-	835	1.18e-08	AAAATATATA CAGTTGTCGGTATTCGAATATCAAACACCT CACTTATAAT
MTR_8g068300	+	1651	1.53e-08	TTGCTCCTCT CAAGGTCTCGAGTTCGATTCCCCAACATTA CCCCCCCCCC
MTR_2g012790	+	105	2.42e-08	AAATCAGACG TTGCGATCGAAGATCGAACCCTGATTCCTT CACTTATATA

Block Diagrams

The height of the motif "block" is proportional to -log(p-value), truncated at the height for a motif with a p-value of 1e-10.
Click on any row to highlight sequence in all motifs. Mouse over the center of the motif blocks to see more information.

Name	Lowest *p*-value
MTR_1g105120	2.41e-12
MTR_4g091020	1.60e-19
MTR_4g047610	2.71e-09
MTR_2g017750	4.81e-09
MTR_4g104020	7.28e-12
MTR_8g022270	1.75e-10
MTR_1g040500	1.30e-11
MTR_5g005950	8.13e-10

Motif Location

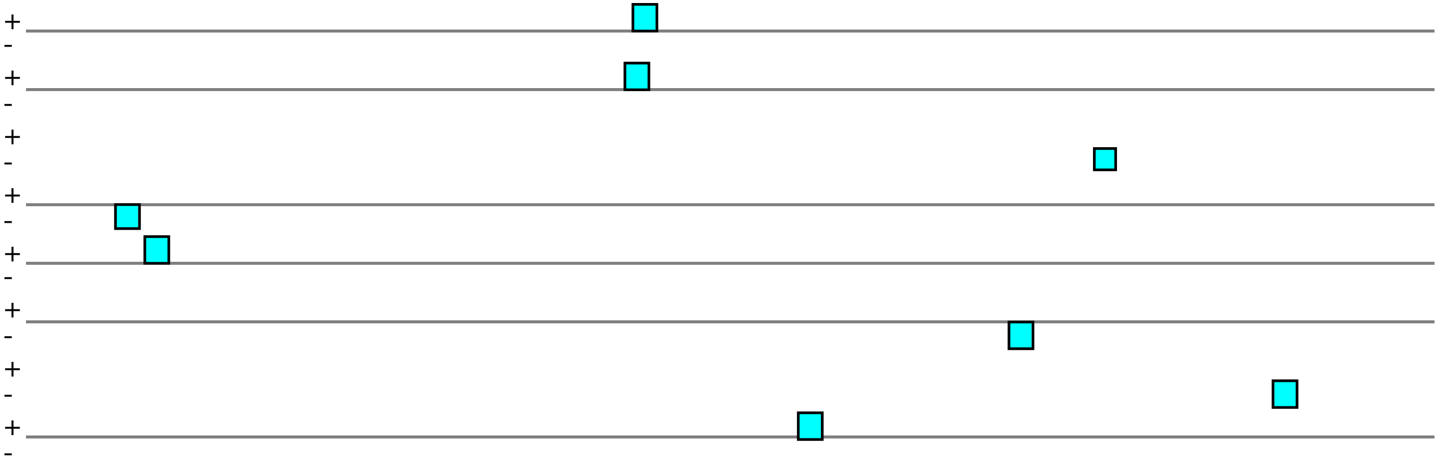

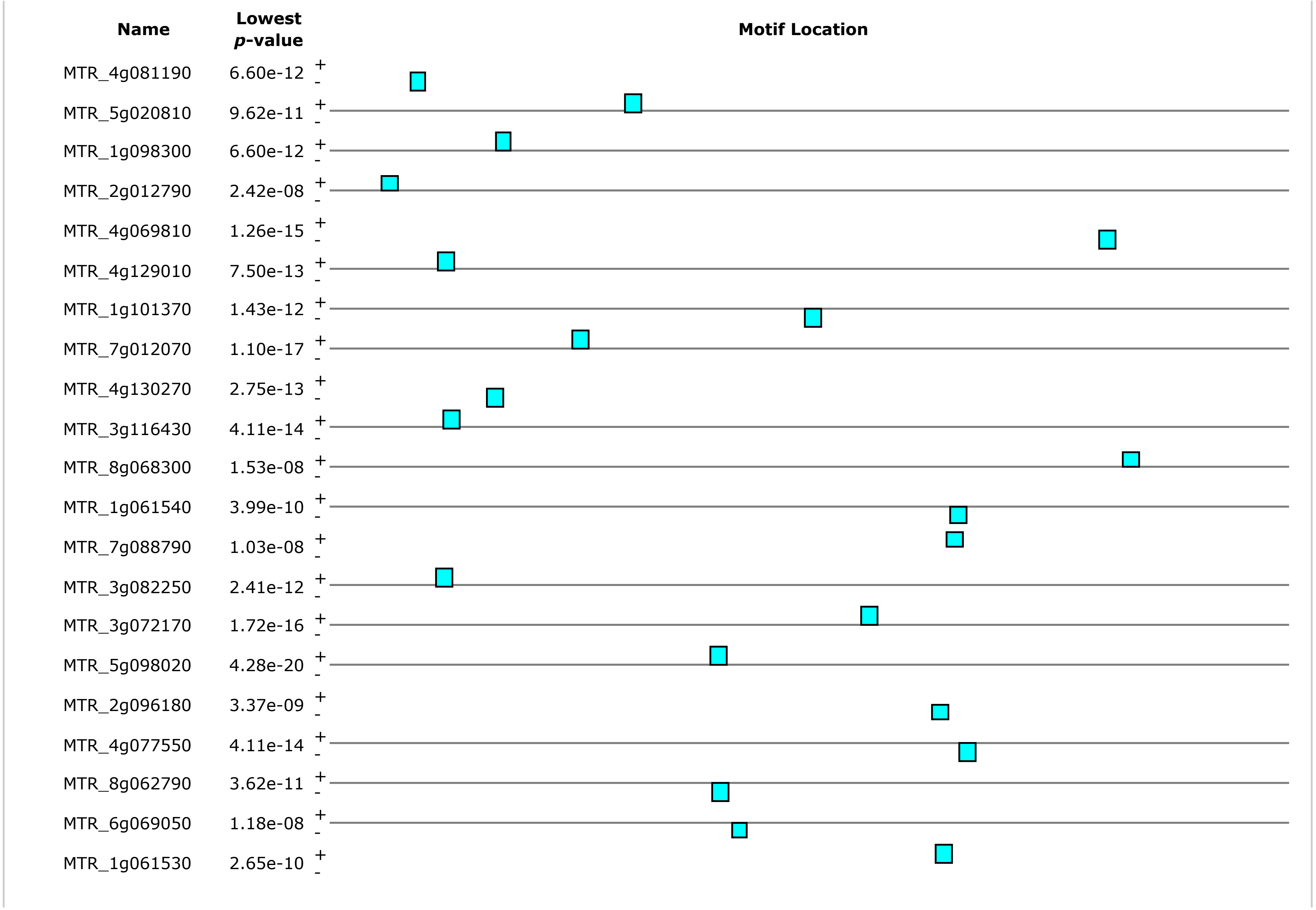
Name
Lowest
p-value
Motif Location
MTR_4g081190 6.60e-12
MTR_5g020810 9.62e-11
MTR_1g098300 6.60e-12
MTR_2g012790 2.42e-08
MTR_4g069810 1.26e-15
MTR_4g129010 7.50e-13
MTR_1g101370 1.43e-12
MTR_7g012070 1.10e-17
MTR_4g130270 2.75e-13
MTR_3g116430 4.11e-14
MTR_8g068300 1.53e-08
MTR_1g061540 3.99e-10
MTR_7g088790 1.03e-08
MTR_3g082250 2.41e-12
MTR_3g072170 1.72e-16
MTR_5g098020 4.28e-20
MTR_2g096180 3.37e-09
MTR_4g077550 4.11e-14
MTR_8g062790 3.62e-11
MTR_6g069050 1.18e-08
MTR_1g061530 2.65e-10

Name	Lowest *p*-value	Motif Location
MTR_4g015650	4.48e-09	
MTR_2g101560	7.59e-17	
MTR_6g012630	3.40e-15	
MTR_3g084200	2.57e-15	
MTR_5g027000	6.97e-16	
MTR_7g020870	2.57e-15	
MTR_7g085310	1.73e-11	
MTR_7g024890	2.83e-14	

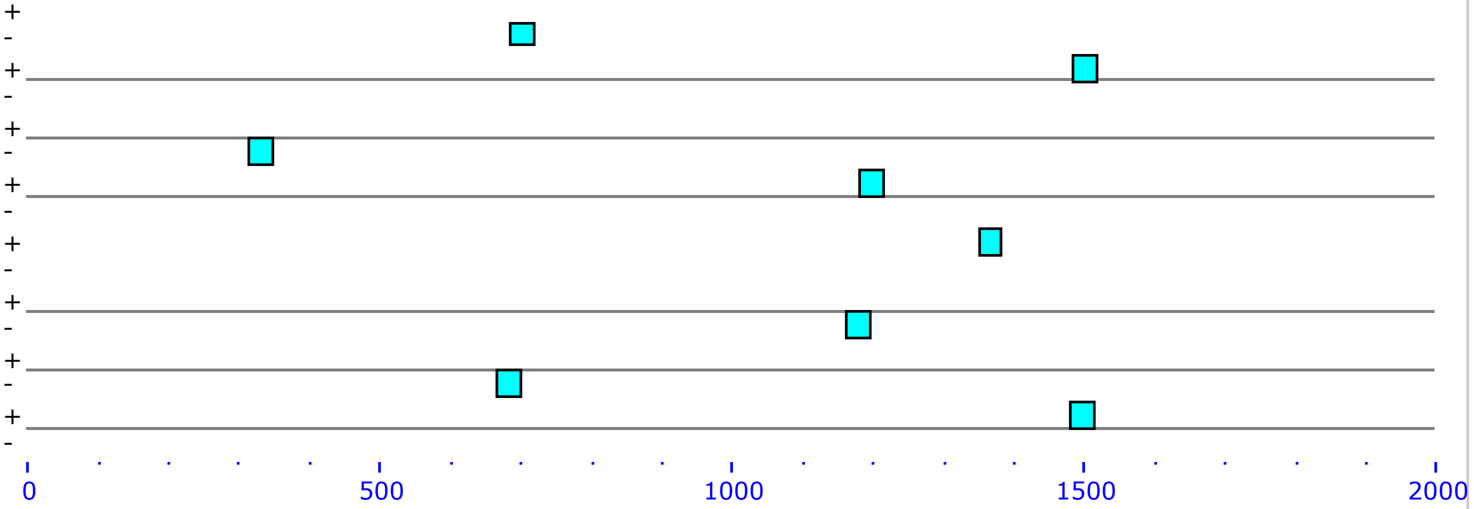

Time 7852.9 secs.

Motif 2

Previous Next Top

Summary ?

E-value	2.8e-054
Width	31
Sites	32

show more

Sequence Logo ?

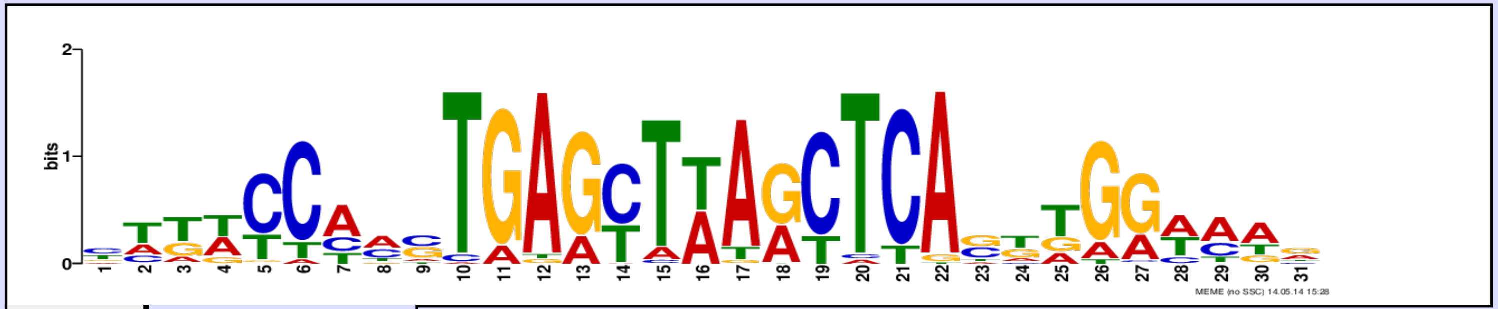

Standard | Reverse Complement

Download LOGO [?] Orientation: standard SSC: off Format:
web (png) Width: 31 cm Height: 7.5 cm Download

Regular expression [?]

[CT][TA][TG][TA][CT]C[ACT][AC][CG]TGA[GA][CT]T[TA]A[GA][CT]TCA[GC][TG][TGA]G[GA][AT][AC][AT][GA]

Further Analysis [?]

Submit this motif to MAST [?] FIMO [?] TOMTOM [?] GOMO [?] BLOCKS [?] Mouse-over buttons for more information.

Data Formats [?]

View the motif in PSPM Format [?] PSSM Format [?] BLOCKS Format [?] FASTA Format [?] Raw Format [?] or Hide

Sites [?]

Click on any row to highlight sequence in all motifs.

Name	Strand	Start	*p*-value	Sites [?]		
MTR_5g098020	-	840	9.58e-15	TATAAATAGA	TTATCCCCGTGAGCTTAGCTCAGTTGGTAGG	GATATTGTAT
MTR_3g082250	+	171	5.55e-14	ATAGAAACAT	TTGTCCCTGTGAGCTTAGCTCACTTGGTAAA	GGATAATGCA
MTR_4g091020	-	800	7.73e-14	ATGCATTGTC	CCTGCCAACTGAGCTATGCTCACGGGGACGC	CATGTTATTG
MTR_4g069810	-	1553	2.72e-13	ATGCAATGTC	CCTACCAACTGAGTTATGCTCACGGGGACAC	TAAATATCCA
MTR_2g101560	+	1437	1.16e-12	ATTGTGTCGT	TAGTCCCCATGAGCTTAGCTCATTTGGTAAG	GGATAATGCA
MTR_6g012630	-	422	1.33e-12	TAAAAAGAAA	TTGTCTTCGTGAGCTTAACTCAGTTGGTAAG	GACAATGCAT
MTR_5g027000	+	1301	2.30e-12	TGGTTTATCA	TATCCCCCGTGAGCTTAGCTCAGTTGATAGG	AATATTGCAT
MTR_3g072170	-	1154	4.45e-12	GAAATGTCAA	GAGTCCCCATGAGCTTAGCTCATCTGGTAAG	GGATAATGCA
MTR_7g012070	-	552	4.45e-12	AAAAATAAAA	GGTTCCCCGTGAGCTTAACTCAGTTGATATG	GATATTGCAT

Name	Strand	Start	*p*-value	Sites
MTR_5g005950	-	1141	1.38e-11	GATGTTTTTA TTGTCCTCGTGAACTTAACTCAGTTGATATG GACAATGTAT
MTR_4g091000	+	758	1.75e-11	AGTTTTATAA GAGTCCTTGTGAGTTTAGCTCAGTTGATATG GACAATGCAT
MTR_7g024890	-	1431	2.51e-11	ATGCATTGTC CTTACCAACAGAACTAAGCTCACGAGGACAA CTAGTAAAAG
MTR_1g098300	-	294	1.51e-10	ATGTATTGTC TCTACCAATTGAACTAAGCTCACGGGAACTT GACGCTTAAT
MTR_8g022270	-	1344	2.08e-10	ATGCATTGTC CTCACCAACTGAGTTAAACTTACGAGAACAG AGAATCTGTT
MTR_4g077550	-	1358	2.31e-10	TCACCTAAAT CTCTCTTTGTGAGCTTAGTTCAGTTGGTATG GACAATGCAT
MTR_4g023730	+	1335	2.56e-10	AGAAAATAAT GTATCATTGTGAGCTTAACTCAGTTGGTCGG GATATTGCAG
MTR_7g085310	-	716	2.84e-10	GGGACTTGGA TTTGTCCCATGAACTTAACTCAGTTGGCAGA GACATATATT
MTR_4g130270	-	277	2.84e-10	AATGCATGTT TCTGCCAGCTGAGCTATGCTCACGGGACTAT ACACTTATGC
MTR_7g020870	-	441	5.22e-10	ATGCATTGTC CATACCAACTGAGCTAGACTCACGATGACAT AAATAAGTTA
MTR_8g062790	+	845	7.02e-09	ATACATTATC TTTACCAACTAAGATAAACTCACGTGCACAA TCTCATTCAC
MTR_1g026070	-	1666	1.17e-08	ATGCATTGTA CATACCAGCTAAATTAAACTCACATAGACAC CCCCTTTTAT
MTR_4g104020	+	118	1.17e-08	ATATTAAAAG AGTTTTATGTGAGCTTAGCTCAATTGGTAGT GATAATGCAT
MTR_3g116430	+	186	2.08e-08	TAAATACACC AATTATCCTTGAGCTTAACTCACTTGATAAG AGATAATGCA
MTR_1g040500	+	1720	2.25e-08	ATAGTTGACT AAATTCTCATGGACTTAGCTCAGTTGATAAG AACAATGCAT
MTR_7g076920	-	1782	3.09e-08	ACAATGAGTT CTAGCCTGCCGTGTTTAGCTCACATGACAAC TACCACAATA
MTR_5g020810	-	660	5.32e-08	GCATTGGTCT CTTTGTCCCCGAATTTAACTCAATTGGTAGA GATATTGCAT
MTR_2g025170	+	1414	7.75e-08	GATAATAACA CATGCCCACTAAACTTTACTCAATAAGACAA TAACACACAG
MTR_8g095090	-	1515	1.12e-07	ACCATTAGAC CAATCCAACTGGGTTATATTCATTTGAATAG TGTAGTTCAC
MTR_1g061540	-	1339	1.49e-07	TTAAATTTTA TTGTTACTGTAAATTTAGTTCAGTTGGTAGA GACAATGCAT
MTR_4g015650	+	636	2.27e-07	AGAAGACAAC ATGTTTCCGTAAACATAACTTAGTTGGTCAA AACAATATAT
MTR_7g012340	+	1434	2.79e-07	GCAAAGAGTT TTATCCTGCCGTGTTTAGCTCAAATGACAAC TACCACAATA
MTR_7g115050	-	368	2.99e-07	ATACAATGTT CATATCAACTAAACTAGACTCACGAAGATTT TGTACCTTAG

Block Diagrams

The height of the motif "block" is proportional to -log(p-value), truncated at the height for a motif with a p-value of 1e-10.

Click on any row to highlight sequence in all motifs. Mouse over the center of the motif blocks to see more information.

Name	Lowest *p*-value
MTR_4g091020	7.73e-14
MTR_7g012340	2.79e-07
MTR_4g104020	1.17e-08
MTR_8g022270	2.08e-10
MTR_1g040500	2.25e-08
MTR_4g091000	1.75e-11
MTR_7g076920	3.09e-08
MTR_5g005950	1.38e-11
MTR_5g020810	5.32e-08
MTR_7g115050	2.99e-07
MTR_1g098300	1.51e-10
MTR_4g069810	2.72e-13
MTR_7g012070	4.45e-12
MTR_4g130270	2.84e-10
MTR_2g025170	7.75e-08
MTR_3g116430	2.08e-08
MTR_1g061540	1.49e-07
MTR_3g082250	5.55e-14
MTR_3g072170	4.45e-12
MTR_5g098020	9.58e-15

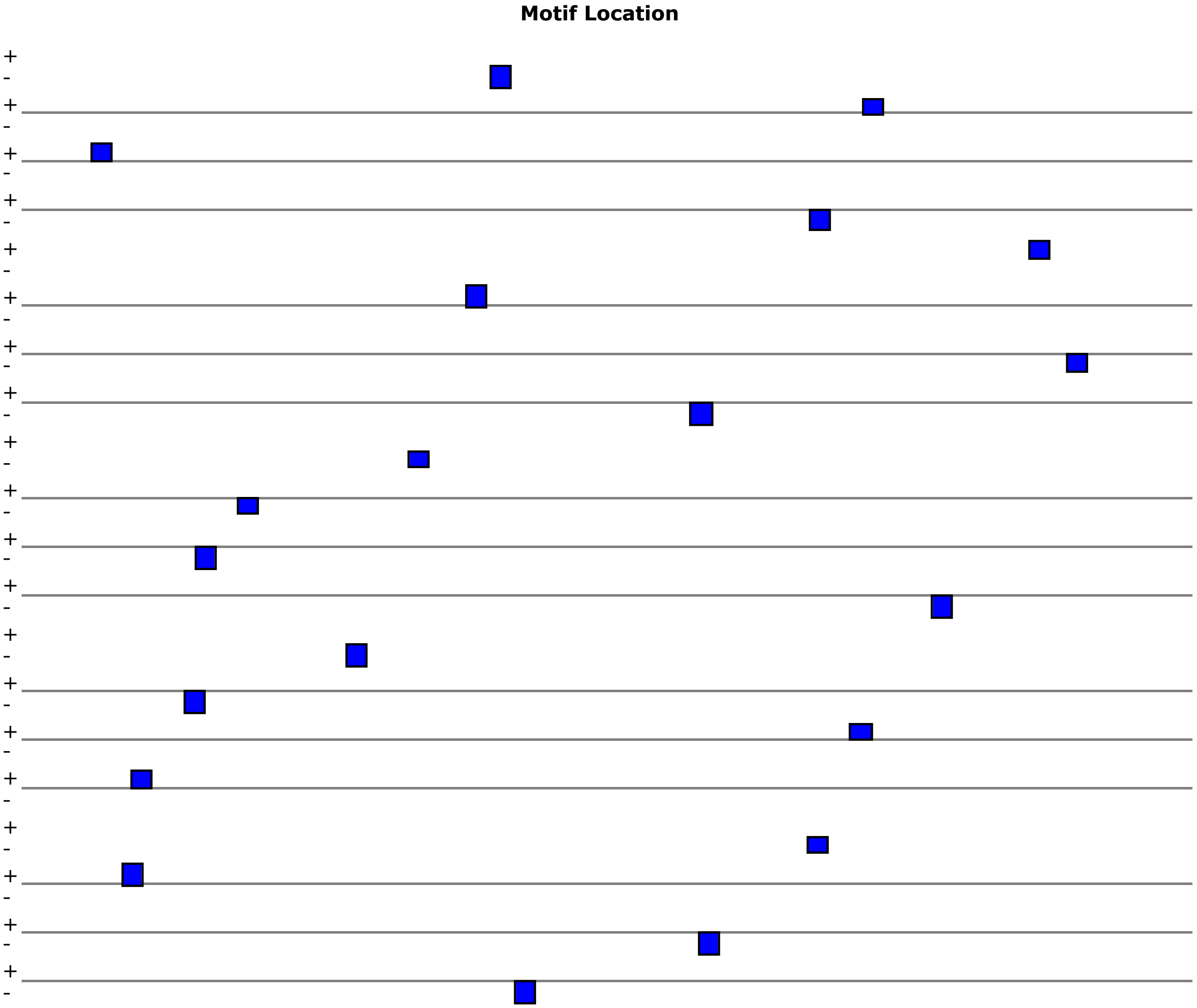

Name	Lowest *p*-value
MTR_4g077550	2.31e-10
MTR_8g062790	7.02e-09
MTR_1g026070	1.17e-08
MTR_4g015650	2.27e-07
MTR_2g101560	1.16e-12
MTR_6g012630	1.33e-12
MTR_4g023730	2.56e-10
MTR_5g027000	2.30e-12
MTR_8g095090	1.12e-07
MTR_7g020870	5.22e-10
MTR_7g085310	2.84e-10
MTR_7g024890	2.51e-11

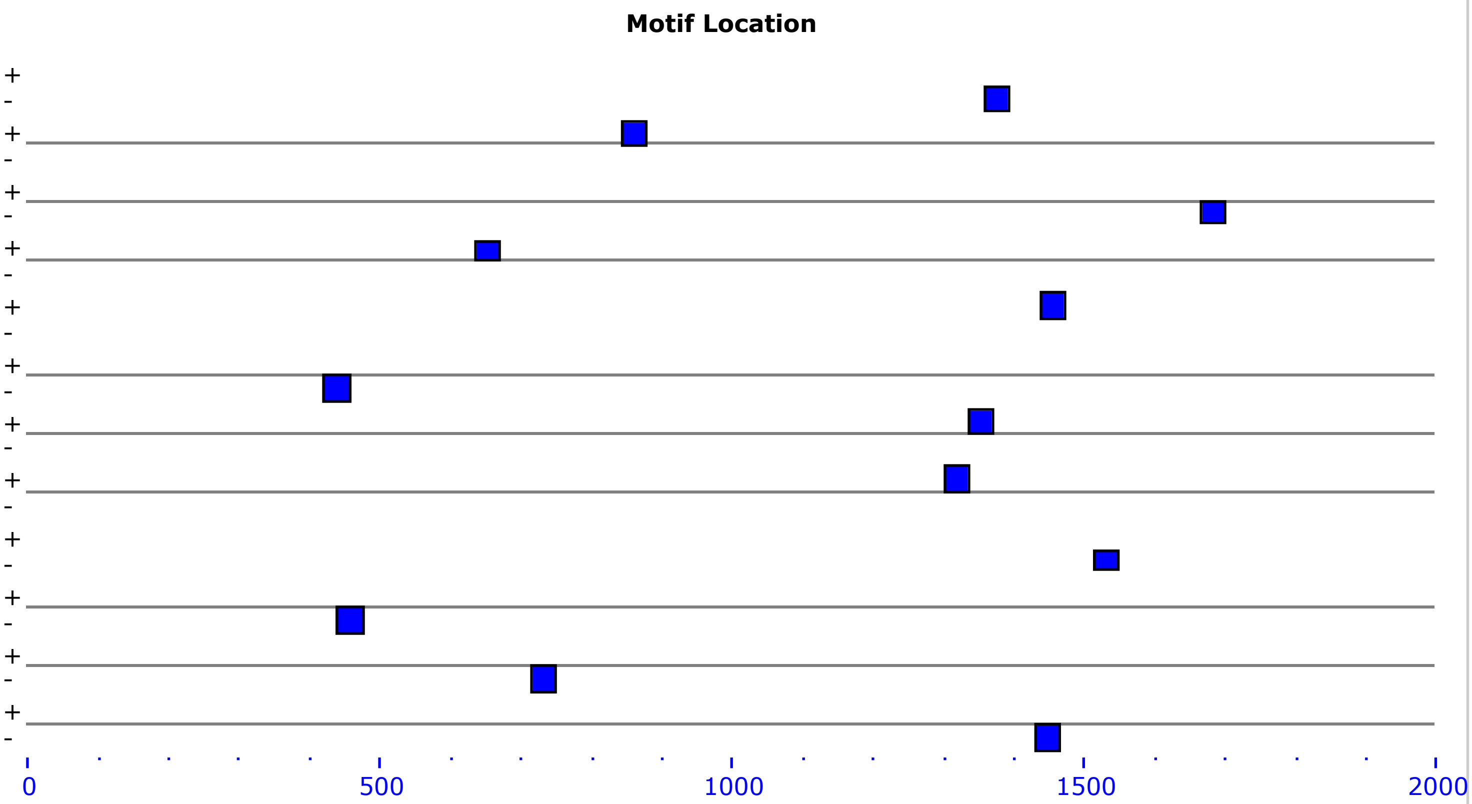

Time 15277.5 secs.

Motif 3

Previous Next Top

Summary ?

E-value	1.8e-027
Width	22
Sites	133

show more

Sequence Logo ?

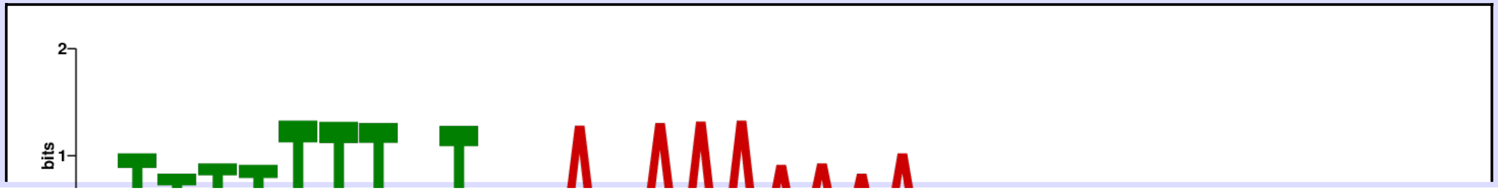

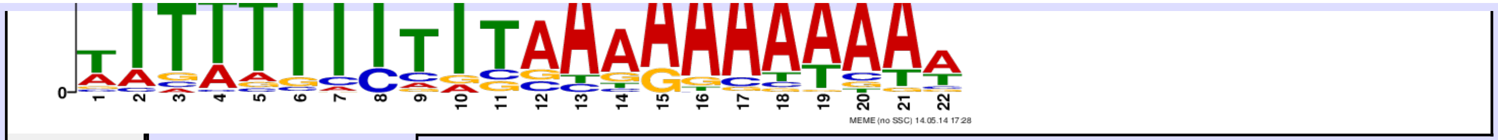

Standard | Reverse Complement

Download LOGO [?] Orientation: standard SSC: off Format: web (png) Width: 22 cm Height: 7.5 cm Download

Regular expression [?]

[TA]TT[TA]TTTTTTTAAAAAAA[AT]AA[AT]

Further Analysis [?]

Submit this motif to MAST [?] FIMO [?] TOMTOM [?] GOMO [?] BLOCKS [?] Mouse-over buttons for more information.

Data Formats [?]

View the motif in ○ PSPM Format [?] ○ PSSM Format [?] ○ BLOCKS Format [?] ○ FASTA Format [?] ○ Raw Format [?] or ● Hide

Sites [?]

Click on any row to highlight sequence in all motifs.

Name	Strand	Start	*p*-value	Sites [?]		
MTR_7g077010	+	790	2.34e-09	TCCTTTCTTT	GTTTTTTTTTTAAAAAAAAAAT	AATAATAAAA
MTR_7g012330	-	1616	5.50e-09	AAATGAATAG	TTTTTTTTCTTCAAAAAAAAAT	TGAATAATTT
MTR_7g116650	-	1765	3.27e-08	ACAACTTTTT	TTTTTCTTCTTAAAAAAAAAAA	AAAACAACTA
MTR_1g079120	-	694	3.27e-08	AGTTTTCTAT	TTTTGTTCCTTAAAAAAAAAAA	GTTTTCTATT
MTR_4g091020	+	387	5.11e-08	AACGAAGGTA	TATATTTTTTCAAAAAAAAAAA	AAAAAGGACA

Name	Strand	Start	*p*-value		Sites	
MTR_5g027000	-	1768	6.99e-08	AAGTTAGACA	ATCTTTTTTTTAAAAAAAACAA	AAGGAATATA
MTR_8g041570	+	94	6.99e-08	ATGAATGTCA	TTTATTTTTGTCAAAAAAAAAA	TGACATAGGC
MTR_3g107810	-	1380	6.99e-08	TGACAATCTA	ATCTTTTCTTTAAGAAAAAAAA	AAAGGACCAA
MTR_7g088790	-	1773	1.12e-07	TATTGACTGT	TTTTTTATTTGAAAAAAAAAAA	GATATGGAAT
MTR_6g087680	+	1442	1.12e-07	AACAACACAC	TCTTTTCTTTTAAAAAAAAAAA	AAAAACACAC
MTR_4g104020	+	315	1.12e-07	TTTTTTTTGT	TTTTTTTTTACAAGAAAAAAAA	AAGTCATTTA
MTR_6g009260	+	1902	1.50e-07	CTAGTTTGAG	TTGTTTTTTTTGACAAAAAAAT	ATAGTTTGAG
MTR_1g114150	+	1035	1.72e-07	ATTTAAATAA	ATTTTGTTTTTACAAAAAAAAA	AAATTGTTAA
MTR_1g101130	-	930	1.72e-07	ATTTAAATAA	ATTTTGTTTTTACAAAAAAAAA	AAATAGTTAA
MTR_6g012630	-	1789	2.02e-07	TTTGCTACAG	CTTTTTTCATGAAAAAAAAAAA	GCCACTTTTT
MTR_7g098040	+	1129	2.02e-07	CACTTTAATT	TTTATTTTATCAAAAAAAAAAT	ATTAGTGACT
MTR_4g047610	+	1741	2.66e-07	CGCGTTGAAA	CTTTATTTTTGAAGAAAAAAAA	ATGTTACAAT
MTR_1g061530	+	1137	3.06e-07	ACGTTGTTCT	ATCATTTTCTCAAAAAAAAAAA	AAAAGTTGTT
MTR_6g012970	+	792	3.06e-07	ATTTAGAAAA	TATTTTTTATTAAAAAAATAAA	AAATATTTTT
MTR_2g011940	-	438	3.47e-07	CAAATATAAG	TTTTTTTTTGTGAAGAAAAATA	TAAGTTCACA
MTR_5g018320	+	1706	4.61e-07	AGTCAAAAAG	TTGTTGTTTTTAATAAAAAAAA	AAATTATTTT
MTR_8g039990	-	717	5.19e-07	CTGTGCGAAT	TATTATTCCTTAAAAAAAAAAA	AAAAAAGTCA
MTR_4g119550	+	1225	5.19e-07	CGTGACATTT	GTGATTTTTTTAAAAAAATAAT	TAAAAAAAGT
MTR_8g068300	+	162	5.19e-07	TGAGAAACAC	GTTTTTTTGATAAAAAAAAAAA	AAATGATGTT
MTR_2g084020	+	603	5.94e-07	ATAGAAATAA	TTATTTTTCTTCAAAAAAACAA	CTCATTTATT
MTR_1g061540	+	300	5.94e-07	ATTAATTATT	TTTTACTTTTTAAAAAAAAAAT	TGAGTCTATA
MTR_2g095800	+	1037	5.94e-07	AACATGAGCA	TATATTTTTGTAAAAAAAAAAT	AAAAGCGTGA
MTR_8g091690	+	804	5.94e-07	TAAATGTTTA	TTTTGTTTGTTAAAAAAAACAT	AAATGTTTAT
MTR_1g019240	-	398	6.75e-07	ATAATATTTT	CTTATTTCTGTCAAAAAAAAAA	AAAAATCTTA
MTR_7g087570	-	521	7.64e-07	TATATAATAT	GTTTTTTTGTTGAAGAAAAGAA	ATATATTTTA
MTR_8g074530	-	999	7.64e-07	GTTTAGCTCA	ATTTCTTCTTTAAAAAAGAAAA	TAAGTTAAAA

Name	Strand	Start	*p*-value	Sites		
MTR_4g086540	-	1562	1.11e-06	TTAAAAATAC	CTTTTTCTCTCAACAAAAAAAA	ATACCTTTTA
MTR_3g116430	-	954	1.11e-06	GTTTAACACT	ATTCTTTTGTTAAAGAAAAAAT	ATTAACACTA
MTR_7g085310	-	805	1.25e-06	AGACACAAGT	GTTCTTTTTTTAATAAAAAAAT	AGACGATTGG
MTR_3g104820	+	568	1.25e-06	TGTTATGTAA	CTTATGTTATTAAAAAAAAAAA	TTGATATACC
MTR_7g086610	-	1643	1.42e-06	TAAGTGGTGC	CATTTTTCTTTAAAAAAAATAA	ATAAGTGGTA
MTR_2g089100	-	1674	1.60e-06	TTTGAGAAGT	CTAATTTTTTGAAAGAAAAAAA	TTGTAGGTCC
MTR_1g105120	+	3	1.60e-06	AA	TTTTTGTCTTTAATAAAATAAT	GAGTAAATAG
MTR_1g082290	-	1813	1.79e-06	ATTTCATTAC	TTTCTTTTCTTAAAAAAAATAT	CATTACTTTC
MTR_7g108430	+	1575	1.79e-06	GACTAATTCC	TATATTTTGTCCAAGAAAAAAA	AAACACTCCT
MTR_3g079320	+	179	2.03e-06	ATGTATCAAA	TTTTTGTTTTTGAAGAATTAAA	TGTATCCAAC
MTR_5g016320	+	671	2.03e-06	CTATACGTAG	CACTTTTTTTTAAAAAAAACAT	TCTTCTTCAA
MTR_6g081120	+	8	2.26e-06	CATGCAT	TTTCGTTTCTTCAAAAAATAAA	CCAAAACAAA
MTR_1g095850	-	1163	2.26e-06	AATAAAGAGA	GATACTTTCTTAAAAAAAAAAA	AAAAAACTAA
MTR_4g133890	+	1316	2.26e-06	CTCACACTTT	CTCTTTTCATTAAAAAAATAAA	AATATAATCA
MTR_5g005950	-	1052	2.26e-06	AAAATGATGT	TTTTTTTTTGTCTAGAAAAAAT	GATATTTTTA
MTR_4g091000	-	683	2.26e-06	AATTTCACCA	CTTTATCCTTCAAAAAAAAAAA	TCACCCTTCA
MTR_3g044600	-	1231	2.85e-06	ATAAGTATAC	TTTTTGTTAGTAAAAAAATAAA	TTTTTTTGTT
MTR_4g023950	-	1151	2.85e-06	ATAAGTATAC	TTTTTGTTAGTAAAAAAATAAA	TTTTTTTTGT
MTR_3g034180	+	375	2.85e-06	TAAATTTAAT	GTGTTTGTTTTAAAAAAAAAAT	TCTTTTTTAT
MTR_8g087720	+	334	2.85e-06	ATTGCCTAAC	TTTTTTATTTTAAAAAAATATT	TCACCCCTTA
MTR_5g029620	+	1208	3.54e-06	AATATATTCT	CCTTTTTTCTTAATAAAAAAAG	TTTACATTTA
MTR_4g130270	+	1297	3.54e-06	AAAATGTTTT	AATAGTTTTTTAAAAAACAAAA	TTAAATAAAA
MTR_4g091010	+	305	3.54e-06	TTAATTCGAT	AAATTTTCTTCCAAAAAAAAAA	AAACTCGATA
MTR_2g099720	-	1221	3.97e-06	TGTGTGTTCA	ATGTTGTTTTGATAAAAAAAAA	TCAATAAATT
MTR_4g023070	+	466	3.97e-06	AAATTCCTAT	TTTAATTTCTGGAAAACAAAAA	TTTAGATTTT
MTR_4g129010	+	1892	4.41e-06	ACCAGTTACC	ATTCTTTCTTTAAAAGAAAATA	TTTCATTTGG

Name	Strand	Start	*p*-value	Sites		
MTR_2g035320	-	591	4.91e-06	TATGCATATC	TTTTTCTTATTAAAAAACTAAA	CATATCTTTT
MTR_3g115940	+	1635	4.91e-06	ATCATATAAA	TTGTGTTTCTTAAAAAACTAAT	AATAATGCAC
MTR_3g100980	-	506	5.48e-06	ATAATAAGTT	TTCAAGTTCTTAAAAAAAAAAA	GTTGTCGAGC
MTR_5g022560	-	927	5.48e-06	ACACTCTCAT	CTCTCTCCTTTAAAAAAAAAAA	TCTGCACTCC
MTR_6g073040	-	1231	5.48e-06	AATATTTTCC	AATTTTTTTTGTAGAAAAAAAA	TTTAAAAACT
MTR_7g076990	+	1788	5.48e-06	ATAATTTATT	TAGTATTTGTCAAAAAAAAAAA	AAGATAATTT
MTR_4g061340	+	832	6.08e-06	AAGATACAGG	GTTTATTTTAGAAAAAAAAAAC	TTACAAGGAC
MTR_7g086220	+	952	6.08e-06	TATATTCTAT	TTTGATTTCTTAAAAAAAAAAT	TATCTAATGA
MTR_7g083570	+	581	6.73e-06	TTAAATATTC	TTTCATTTTTCAAAAACAAAAT	ATGCTTTAAC
MTR_7g035020	-	677	6.73e-06	TATATTCCAT	TTTTATGTCTTAAAAAAAAAAC	TATCATTCGC
MTR_5g083030	+	1351	6.73e-06	AACTTTGAAT	TTTATTTTTGTCAGAAATTAAA	TTGGATCGCG
MTR_1g017910	-	1534	6.73e-06	CATATCGTAC	ATATTTTTTATAAAAAAAAATA	ATAAAAATAA
MTR_8g068050	+	603	6.73e-06	AAAAACAAGA	TGTTTTTTGTTGAGGAAAAAAA	TGAAGATGTT
MTR_6g079630	+	1046	6.73e-06	GCTACTATAA	TTGTCTCTTTTAAAAAAAAATT	CCAAGACCTC
MTR_2g098490	+	232	6.73e-06	GATACTATTG	GTTATTTTCTTCATAAAATAAG	TGTATATATT
MTR_7g012070	+	745	7.48e-06	CACACATAAT	TTTATTTTGTTCTAGAAATAAA	TATTTGGCAG
MTR_8g008820	+	756	8.28e-06	TGCACAAATA	ATTAATCCTTCAAAAAAAAAAT	AATGGACAAA
MTR_2g007970	-	1193	8.28e-06	TAAGGCATTG	CATTTTTTATTAATAAAATAAA	CAACAACAAT
MTR_1g098300	-	1256	8.28e-06	AGTTAAATGA	GTTATTTTTTGTAGGAAAAAAA	TATTTTAATG
MTR_5g018570	+	1374	9.16e-06	CAACTGCTTC	TTGTATTTATTCAAGAAATAAA	ATCTAATATT
MTR_3g084200	+	1976	1.01e-05	TGAGAAATTC	GTCTTGTTCTTCAAAAAAAATT	GAA
MTR_2g062840	-	180	1.01e-05	ATAATCACAA	ACCATTCTTTCAAAAAAAAAAA	ACAATCATAG
MTR_4g053630	-	1875	1.01e-05	TAACTTGCAT	GTTATTTTCATAATAAAAAAAT	CAGTTGCCGG
MTR_3g083630	-	268	1.01e-05	ACCAACCTTT	CATTTGTTTTTAAAAACCAAAA	TGATATTTAA
MTR_1g089400	+	136	1.12e-05	AATGGTTAAT	ATCTATTTTGCAAAAAAAAAAG	AAGAAGTCCA
MTR_1g101370	+	1244	1.12e-05	CGTCCTGTCT	CTTATTCCCTCAAAAAAGAAAA	TGTCCTATCT

Name	Strand	Start	*p*-value	Sites		
MTR_7g115050	-	522	1.12e-05	AGAAATTCAT	ATTTTTCTATTAAAAAAAACTT	CGTAGCACTT
MTR_4g021210	+	991	1.36e-05	CATTAATTTT	TAGATGTTTGTAAAAAAAAAAT	AAAAAAAAAA
MTR_4g015650	-	1767	1.36e-05	AAATGAAACT	TTTTTCTCTTGGTAGAAAAAAT	AGAAAAACTT
MTR_7g080180	+	1643	1.36e-05	ATCTTGAGTA	TCTATTCTCATAAAAAAAAAAA	AAAAAACTAA
MTR_7g024890	+	1838	1.65e-05	ATAATTTAAC	ACATTTCTCTCAAAAAAAAAAA	AATTAACACA
MTR_7g112440	-	129	1.65e-05	CCTATGTGAA	ATGGTTTTCTCAAAGAAAAAAT	TTGATGTAGG
MTR_8g062790	+	1007	1.65e-05	TTTTTCTGGT	GTATTTTTTTTGAAGAAAACTT	AGGTACAATT
MTR_4g113820	+	399	1.65e-05	GCGATATAAA	GTTAATTTATTCAAAAAAAATA	ATATAAAGCA
MTR_2g081600	+	170	1.65e-05	AAAAAACGTG	ACATTTTTTTTAAGGGAAAAAA	TGGGGCGGTT
MTR_3g027150	+	364	1.81e-05	ACAAAATGAA	ATTTTCATCTTAAAGAAAAAAC	TAACTTGTGT
MTR_4g077550	+	1468	1.81e-05	CATGTAAAAA	CTCTTTATCTCCAAAAAAAAAT	ATGTAAAAAC
MTR_3g072170	-	765	1.81e-05	TACAATATAT	TCTAGCTCCTTAAAAAAAAAAA	AAAACACTAC
MTR_8g075990	+	1238	1.81e-05	TTGTCACTTA	CATTTTTCTGTAAAAAAATATT	ATCCAACAAC
MTR_1g021110	+	617	1.99e-05	GAATAAATTA	TTTTTCTCATGAATAAAATAAA	CAAAACACTT
MTR_5g029690	+	1706	1.99e-05	CACTTGTCAT	TTCTCTATTTTCAAAAAATAAA	GTTTTATCGT
MTR_5g098020	+	1383	1.99e-05	ATTTTAATGA	TATTTGTTTGGCAAAAATAAAA	TTTAGCGTCC
MTR_3g107650	-	1181	1.99e-05	TAACATGTCA	TTGTTGTCTACAAAGAAATAAA	ACATGGTATA
MTR_4g106730	-	1238	2.18e-05	GGCAGCTACT	GCCAGTTTTTCAAAAAAAAAAT	TGTTTTTTTT
MTR_1g102230	-	959	2.18e-05	CCTCGTAAAA	ATTTTATTTTCCAAAAAAACAT	CTATAATAGA
MTR_5g073260	-	1252	2.18e-05	AAAATTTAAA	AATTATTCTTTAAAAAAATTAA	ATATTAAAAA
MTR_4g118770	-	1976	2.18e-05	CGT	GTAATTTTGATAAAAAAAAAAA	TAAAAAAAAT
MTR_5g094380	+	256	2.18e-05	TTCGCAAATT	TCTATTTTCTTCATAAAATCAA	ACACGCAAGT
MTR_4g081190	+	480	2.40e-05	CTCAACCTAA	ATTTGTATTAGAAAAAAAAAAA	CTACCTATTC
MTR_6g060570	+	1895	2.62e-05	TCACCTCATT	GTCACTTTCTCGAGAAAAAAAT	AACTCAGAAA
MTR_8g095090	-	1771	2.62e-05	AGTTTCGTTT	ATTAATCTTTCATAAAAAAAAA	ATACATAACA
MTR_1g102160	+	1842	2.62e-05	ATATTTCTTT	ATTATTTCCTCTAAAAAAAATA	ACGGAAGATT

Name	Strand	Start	*p*-value	Sites		
MTR_8g038210	-	138	2.62e-05	ATTAAATTTG	GTGTATATTTTGAAGAAAAAAA	AATTGATAGG
MTR_4g023730	-	281	2.87e-05	AGGTGACTAA	GTGACTTTTTTTAAAAAAAAAA	GGTGACGTGT
MTR_4g115120	-	298	2.87e-05	CCTTTCGAAT	ATATGTTTCTTACAAAAAAGAA	TGGTGATAAG
MTR_3g100600	+	1607	3.14e-05	AAGAAGAAAA	AATTGTTCTACAAAGAAAAAAT	GTTCTTGAGA
MTR_1g087030	+	242	3.43e-05	TCATGCTAAC	CATTGTTTGTGCAAAAAGAAAA	GGCAATCGGC
MTR_2g100350	+	1047	3.75e-05	ATAAGAATTA	TTTACTCTTTTCAAAAAATAGT	TTTTTCTATG
MTR_5g078030	+	218	3.75e-05	ATATAATACA	TATACTCTTTCAAAAAATAAAA	ATTACAAACA
MTR_3g061170	+	320	4.46e-05	GACTAGACAT	AATAGTTTTAGGAAGAAAAAAA	ATGAAGAATC
MTR_2g096180	-	1437	4.46e-05	AATATTTAGT	ACTATTTTATGATAAAAAAAAT	ATTTAGTAAT
MTR_5g018610	+	1114	4.46e-05	AAAGAAGGTA	AATTTTGTTTTAAAAAAATTAA	GATGCTTATT
MTR_8g087780	+	1823	4.46e-05	TAAAATACAA	TATTTTTTTGGGAAAAATTATA	GTATTAAAAT
MTR_1g086970	-	1525	4.86e-05	AATAGTATGT	TTCATATTTTGACAAAAAAAAA	TCGTATATTT
MTR_5g033490	-	1921	5.29e-05	TATATGGATG	TAGCTTTTTTCAAGAAACAGAA	AAATCACAAA
MTR_4g122750	+	226	5.29e-05	GTAATTACTC	ATGTTCACTTTAAAAAAAAATA	ATTTTTTGTT
MTR_7g009410	-	437	5.75e-05	AATACAAAAA	TAGTTTTTGTCCATAAAAAATT	GTATAAGACC
MTR_1g018470	-	1364	6.25e-05	TTTTGTTGGA	TTTCCTTCTTTACAAAAAAGTA	ACTTATTTTA
MTR_6g029180	+	1811	6.25e-05	AAATAATTTA	ACATATTTATTAAAAAAAAAAT	TATAAGAAAT
MTR_5g043550	+	1270	6.25e-05	TTAACAATAA	GGGTTTCCCTCAAAAAAAAAAA	AAATGAGGGT
MTR_7g037430	+	376	6.79e-05	TATATAAACG	ATTAACTCTTTAAAAAAAATAT	ATATAAACGA
MTR_4g071090	-	1512	7.37e-05	ATTAATTTTA	TTTTTTATTTTTAGAAAAAATG	TCTCTTTATA
MTR_7g098160	+	1234	7.37e-05	TCTCATATCA	TATTACTTGATAAAAAAAAAAA	TTGAAGGAAT
MTR_1g040500	+	1660	7.37e-05	TTATTAATAA	ATATTGTCGTTCAAAGAAAAAT	CATACAAAGG
MTR_2g025170	+	1543	8.00e-05	GTAAATGGGG	ATGAATATATTAAAAAAAAAAA	TCCACGTGGA
MTR_7g070810	-	588	1.10e-04	AACCAATGAT	TCCCTTTTGTTACCAAAAAAAA	GATCAACCAA

Block Diagrams

The height of the motif "block" is proportional to -log(p-value), truncated at the height for a motif with a p-value of 1e-10.
Click on any row to highlight sequence in all motifs. Mouse over the center of the motif blocks to see more information.

**Name**	**Lowest *p*-value**
MTR_1g105120	1.60e-06
MTR_7g086220	6.08e-06
MTR_7g076990	5.48e-06
MTR_4g091010	3.54e-06
MTR_2g098490	6.73e-06
MTR_6g073040	5.48e-06
MTR_5g022560	5.48e-06
MTR_4g091020	5.11e-08
MTR_3g107810	6.99e-08
MTR_4g047610	2.66e-07
MTR_2g081600	1.65e-05
MTR_5g043550	6.25e-05
MTR_8g091690	5.94e-07
MTR_4g104020	1.12e-07
MTR_7g077010	2.34e-09
MTR_6g079630	6.73e-06
MTR_1g040500	7.37e-05
MTR_8g087780	4.46e-05
MTR_4g091000	2.26e-06

Motif Location

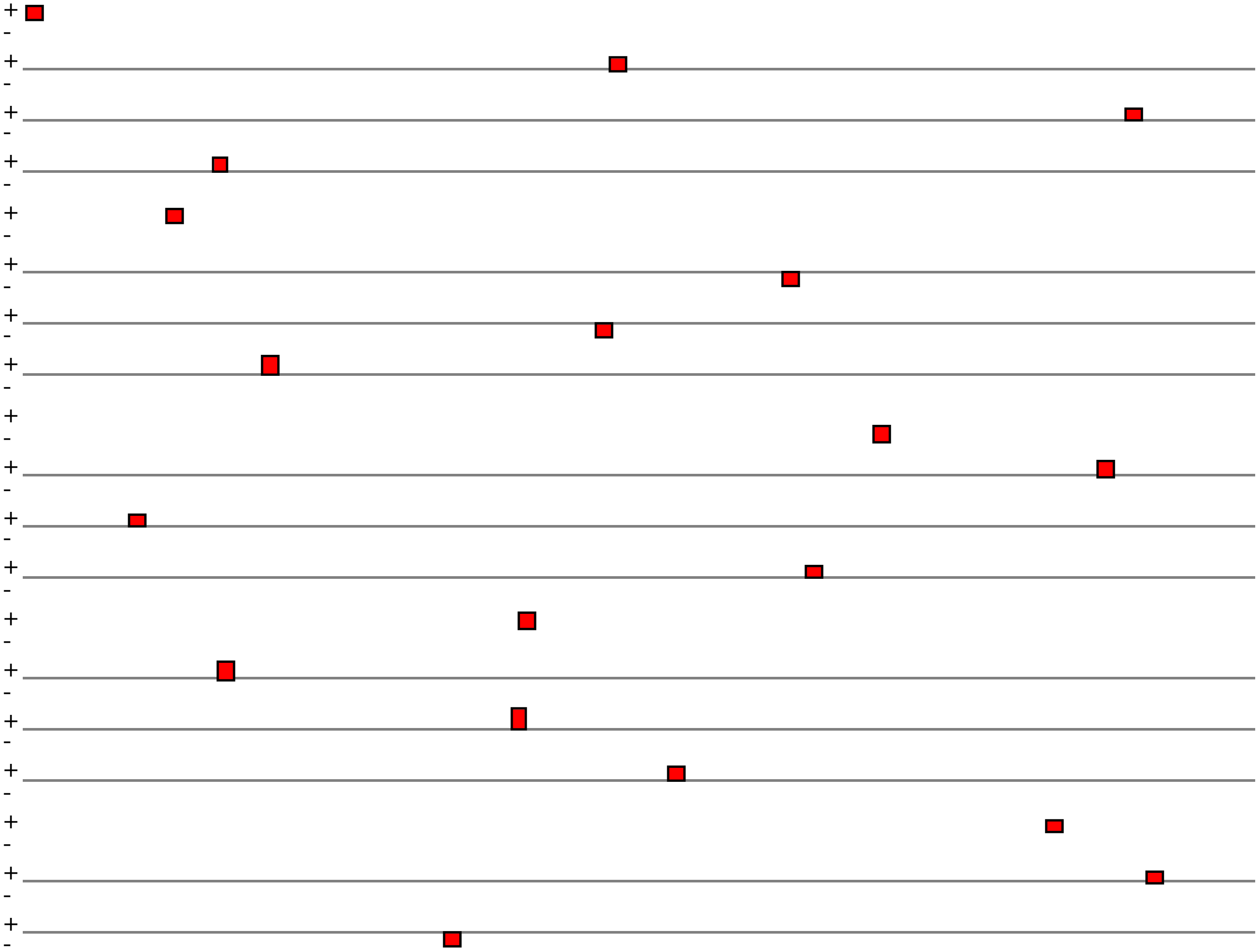

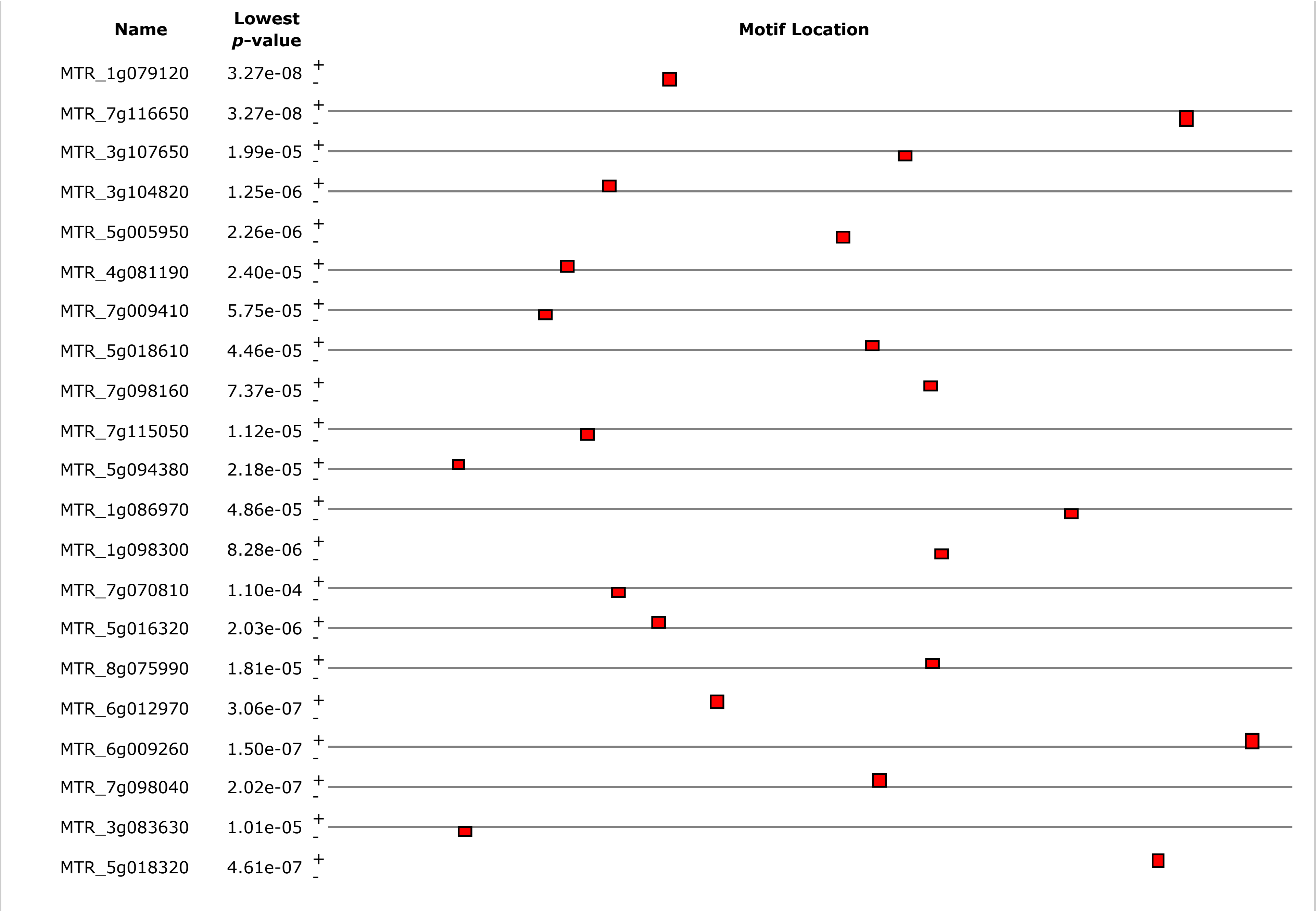
Name
Lowest p-value
Motif Location
MTR_1g079120 3.27e-08
MTR_7g116650 3.27e-08
MTR_3g107650 1.99e-05
MTR_3g104820 1.25e-06
MTR_5g005950 2.26e-06
MTR_4g081190 2.40e-05
MTR_7g009410 5.75e-05
MTR_5g018610 4.46e-05
MTR_7g098160 7.37e-05
MTR_7g115050 1.12e-05
MTR_5g094380 2.18e-05
MTR_1g086970 4.86e-05
MTR_1g098300 8.28e-06
MTR_7g070810 1.10e-04
MTR_5g016320 2.03e-06
MTR_8g075990 1.81e-05
MTR_6g012970 3.06e-07
MTR_6g009260 1.50e-07
MTR_7g098040 2.02e-07
MTR_3g083630 1.01e-05
MTR_5g018320 4.61e-07

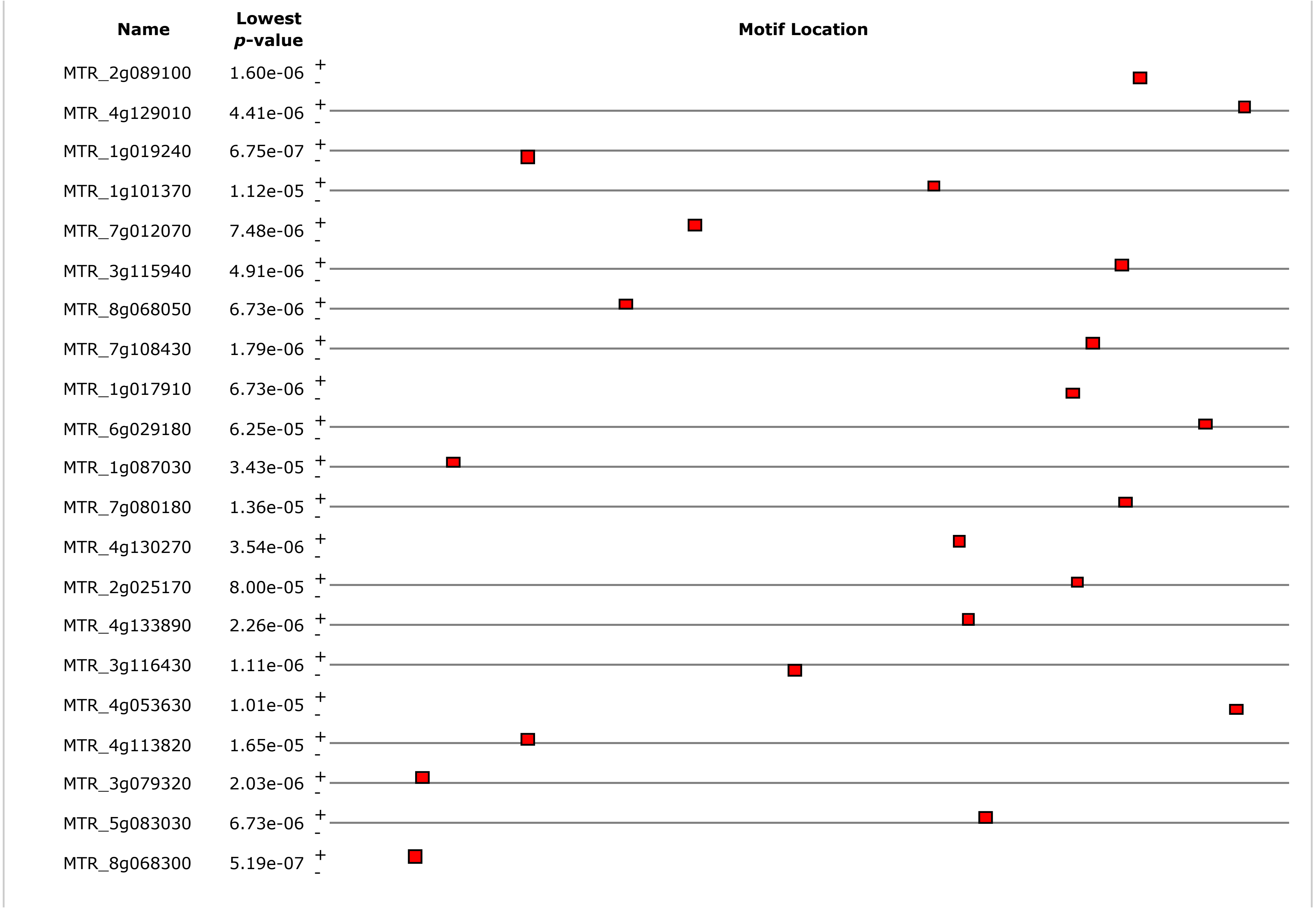
Name
Lowest p-value
Motif Location
MTR_2g089100 1.60e-06
MTR_4g129010 4.41e-06
MTR_1g019240 6.75e-07
MTR_1g101370 1.12e-05
MTR_7g012070 7.48e-06
MTR_3g115940 4.91e-06
MTR_8g068050 6.73e-06
MTR_7g108430 1.79e-06
MTR_1g017910 6.73e-06
MTR_6g029180 6.25e-05
MTR_1g087030 3.43e-05
MTR_7g080180 1.36e-05
MTR_4g130270 3.54e-06
MTR_2g025170 8.00e-05
MTR_4g133890 2.26e-06
MTR_3g116430 1.11e-06
MTR_4g053630 1.01e-05
MTR_4g113820 1.65e-05
MTR_3g079320 2.03e-06
MTR_5g083030 6.73e-06
MTR_8g068300 5.19e-07

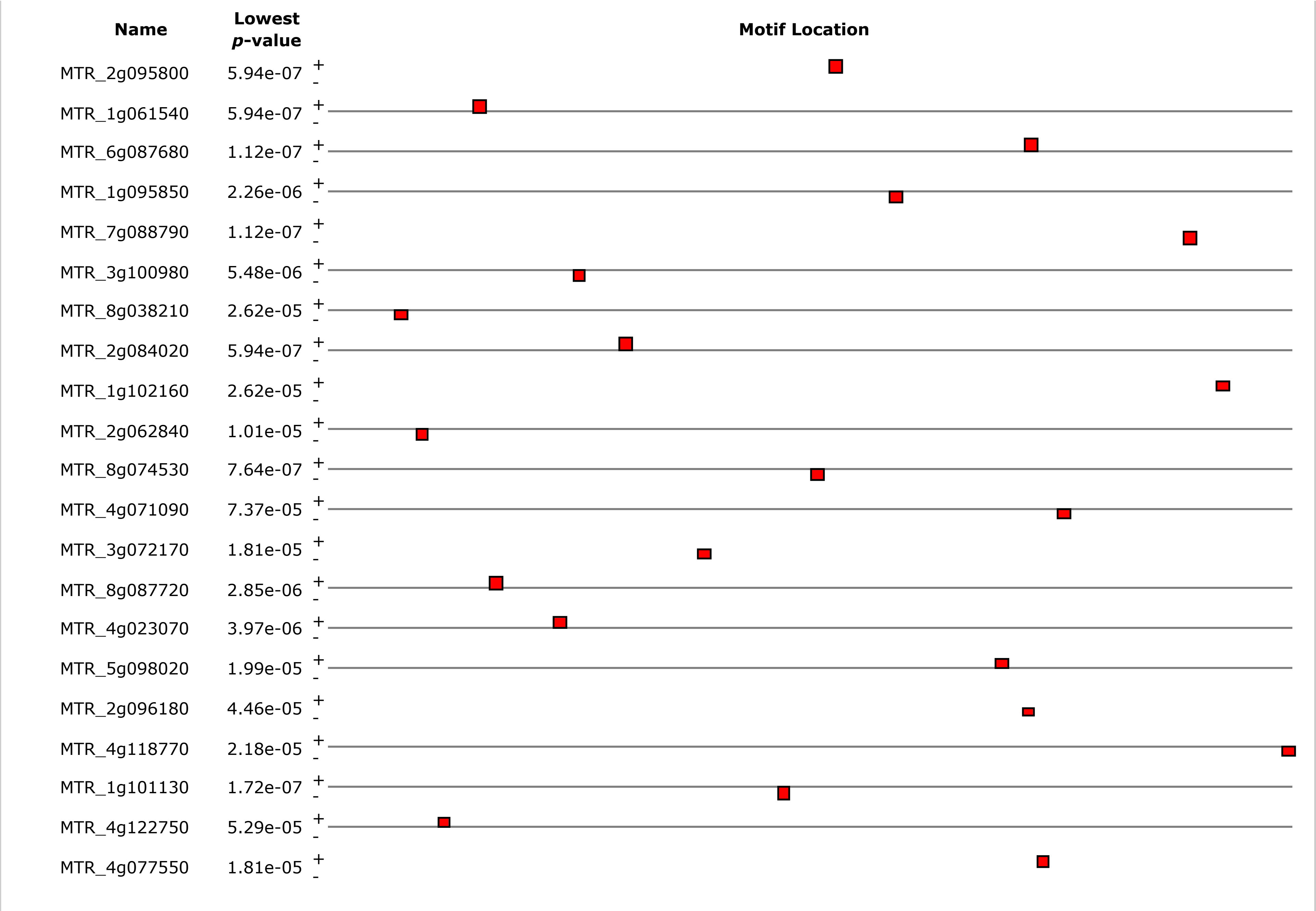
Name
Lowest
p-value
Motif Location
MTR_2g095800 5.94e-07
MTR_1g061540 5.94e-07
MTR_6g087680 1.12e-07
MTR_1g095850 2.26e-06
MTR_7g088790 1.12e-07
MTR_3g100980 5.48e-06
MTR_8g038210 2.62e-05
MTR_2g084020 5.94e-07
MTR_1g102160 2.62e-05
MTR_2g062840 1.01e-05
MTR_8g074530 7.64e-07
MTR_4g071090 7.37e-05
MTR_3g072170 1.81e-05
MTR_8g087720 2.85e-06
MTR_4g023070 3.97e-06
MTR_5g098020 1.99e-05
MTR_2g096180 4.46e-05
MTR_4g118770 2.18e-05
MTR_1g101130 1.72e-07
MTR_4g122750 5.29e-05
MTR_4g077550 1.81e-05

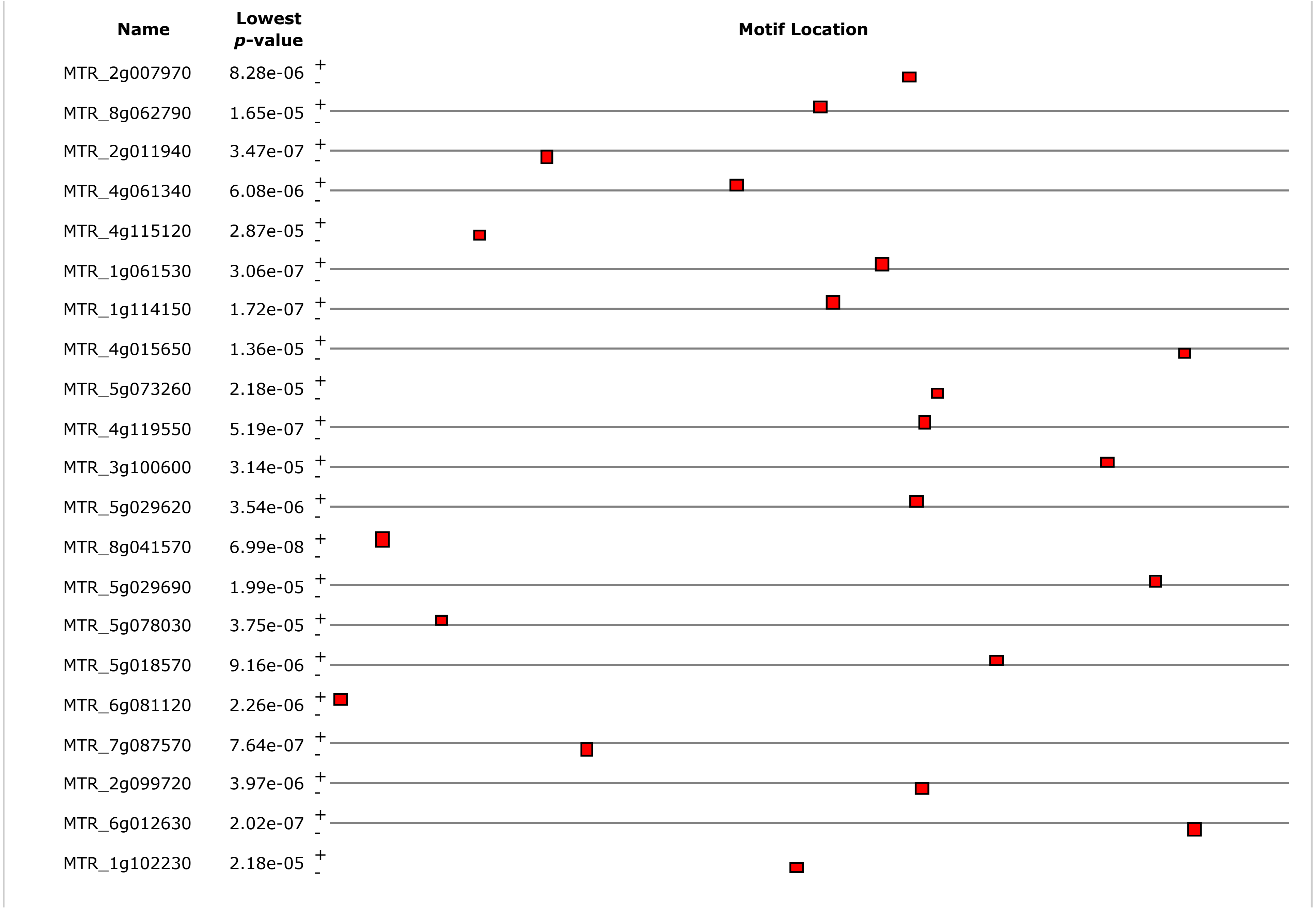

Name
Lowest p-value
Motif Location
MTR_2g007970 8.28e-06
MTR_8g062790 1.65e-05
MTR_2g011940 3.47e-07
MTR_4g061340 6.08e-06
MTR_4g115120 2.87e-05
MTR_1g061530 3.06e-07
MTR_1g114150 1.72e-07
MTR_4g015650 1.36e-05
MTR_5g073260 2.18e-05
MTR_4g119550 5.19e-07
MTR_3g100600 3.14e-05
MTR_5g029620 3.54e-06
MTR_8g041570 6.99e-08
MTR_5g029690 1.99e-05
MTR_5g078030 3.75e-05
MTR_5g018570 9.16e-06
MTR_6g081120 2.26e-06
MTR_7g087570 7.64e-07
MTR_2g099720 3.97e-06
MTR_6g012630 2.02e-07
MTR_1g102230 2.18e-05

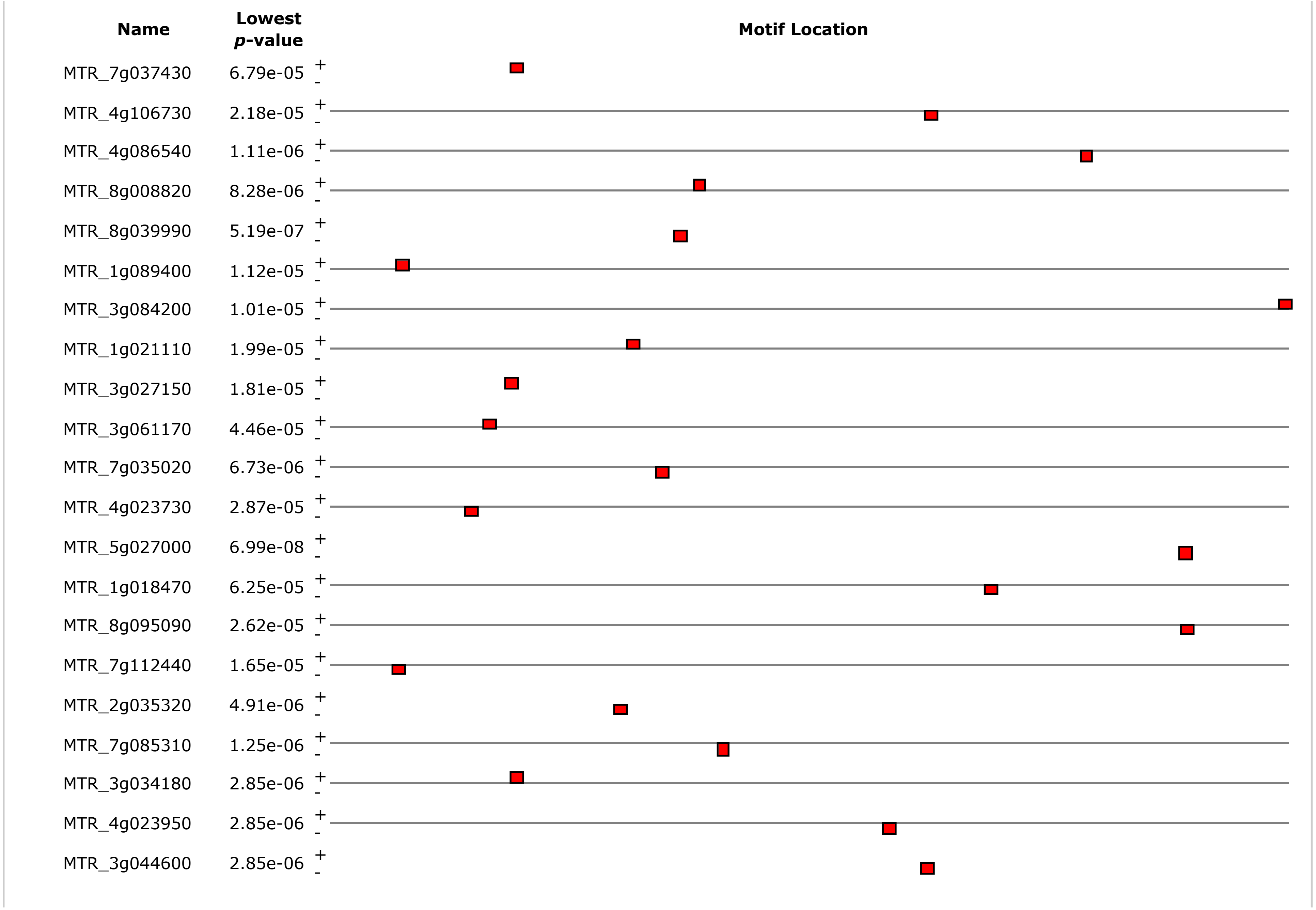
Name
Lowest p-value
Motif Location
MTR_7g037430 6.79e-05
MTR_4g106730 2.18e-05
MTR_4g086540 1.11e-06
MTR_8g008820 8.28e-06
MTR_8g039990 5.19e-07
MTR_1g089400 1.12e-05
MTR_3g084200 1.01e-05
MTR_1g021110 1.99e-05
MTR_3g027150 1.81e-05
MTR_3g061170 4.46e-05
MTR_7g035020 6.73e-06
MTR_4g023730 2.87e-05
MTR_5g027000 6.99e-08
MTR_1g018470 6.25e-05
MTR_8g095090 2.62e-05
MTR_7g112440 1.65e-05
MTR_2g035320 4.91e-06
MTR_7g085310 1.25e-06
MTR_3g034180 2.85e-06
MTR_4g023950 2.85e-06
MTR_3g044600 2.85e-06

Name	Lowest *p*-value
MTR_6g060570	2.62e-05
MTR_7g012330	5.50e-09
MTR_1g082290	1.79e-06
MTR_5g033490	5.29e-05
MTR_7g024890	1.65e-05
MTR_7g086610	1.42e-06
MTR_4g021210	1.36e-05
MTR_7g083570	6.73e-06
MTR_2g100350	3.75e-05

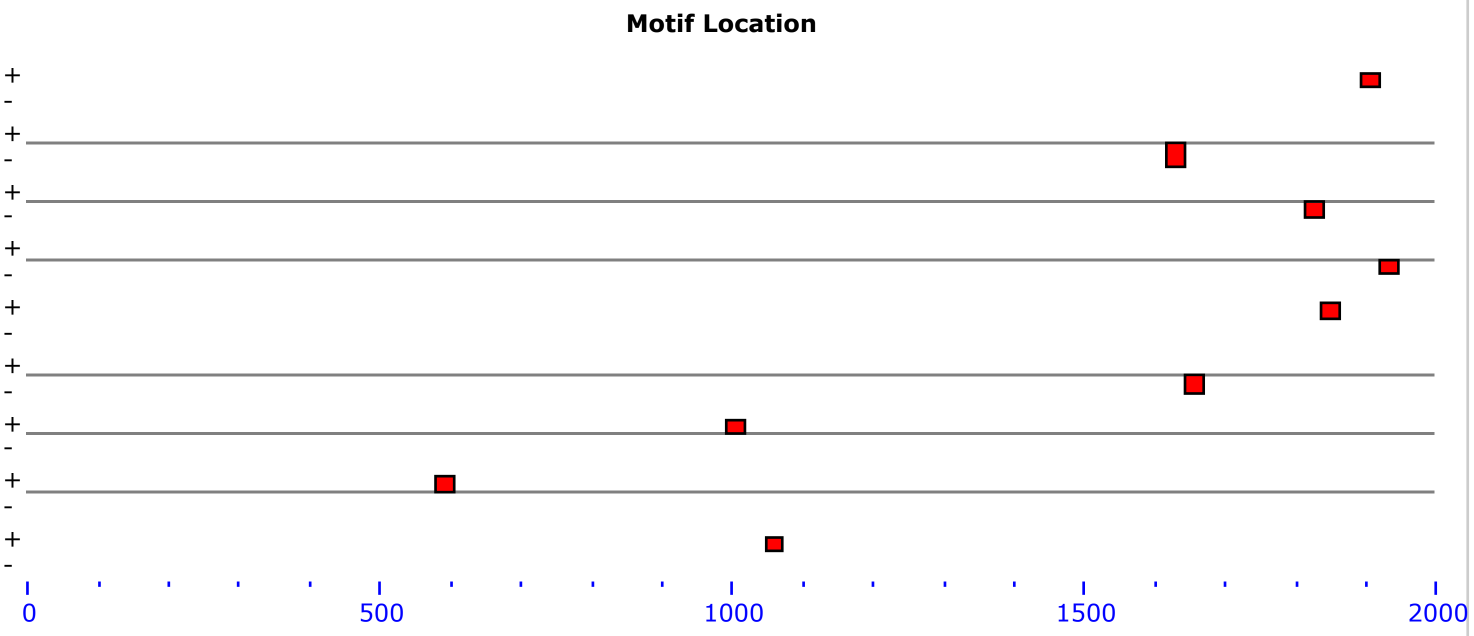

Time 22431.9 secs.

Motif 4

Previous Next Top

Summary [?]

E-value	2.7e-015
Width	29
Sites	12

show more

Sequence Logo [?]

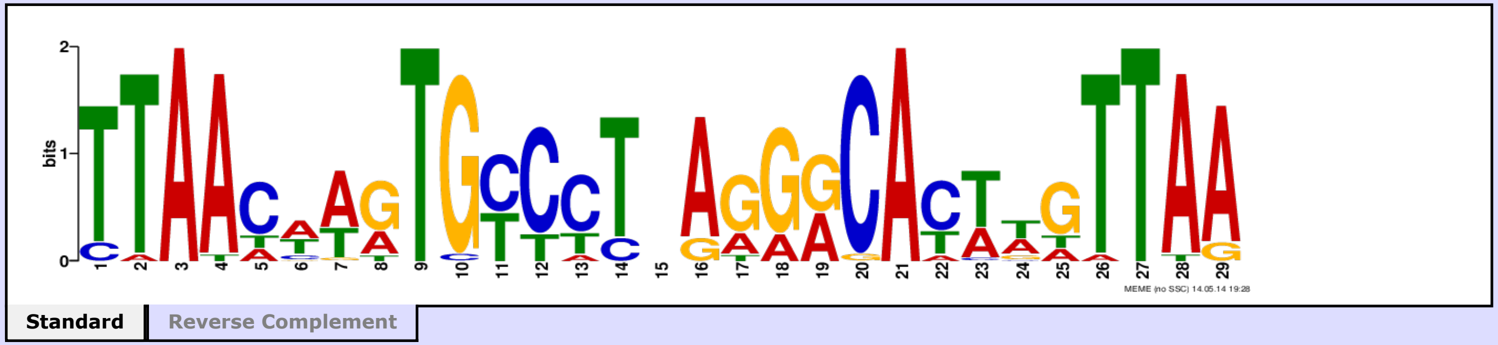

Download LOGO [?] Orientation: standard SSC: off Format:

web (png) Width: 29 cm Height: 7.5 cm Download

Regular expression [?]

TTAAC[AT][AT][GA]TG[CT][CT][CT]T[TAGC]A[GA][GA][GA]CA[CT][TA][TA]GTTAA

Further Analysis [?]

Submit this motif to MAST [?] FIMO [?] TOMTOM [?] GOMO [?] BLOCKS [?] Mouse-over buttons for more information.

Data Formats [?]

View the motif in ○ PSPM Format [?] ○ PSSM Format [?] ○ BLOCKS Format [?] ○ FASTA Format [?] ○ Raw Format [?] or ● Hide

Sites [?]

Click on any row to highlight sequence in all motifs.

Name	Strand	Start	*p*-value	Sites [?]		
MTR_7g024890	-	1049	1.78e-13	TTTAAGGTCC	TTAACTAATGCCCCGGAGGCACTAGTTAA	ATATTCGTAT
MTR_3g061170	-	1613	5.03e-13	AAATGTAATG	TTAACCGGTGCCTTCGGGACACTAGTTAA	TGAAACCATA
MTR_1g101130	-	204	9.65e-13	TTTGAACTTC	TTAAAAAGTGTCCTTAGAACACTTGTTAA	CATTTTCCCA
MTR_1g095850	+	1128	1.63e-12	TAATAATATA	TTAACAAGTGTCATGAGGGCACTTTTTAG	TTTTTTTTTT
MTR_2g011940	-	1716	5.81e-12	TTGGATCTGG	TTAACAAGTCTCCTGAGGGCATTGTTTAA	AGAACCCACA
MTR_7g012330	+	1389	9.21e-12	AAGTGTAGTA	TTAATTTGTGTCCTTAGAGCATATGTTAA	GAAATTTAAA
MTR_5g029690	-	1522	1.20e-11	TTTTAATACT	TTAACCAATGCCATTGGGGCATTAGTTAG	CATTTTCCTA
MTR_5g018320	-	168	1.56e-11	GTTTGGCTTT	TAAACTTGTGCCTTAAGGGCACAAATTAA	CATTTTCCAT

Name	Strand	Start	*p*-value	Sites
MTR_3g044600	-	1844	2.82e-11	TTTTATCATG TTAACTTTTGTCCTTAAGACATATGTTAA AAAACTCAAA
MTR_4g023950	-	1764	2.82e-11	TTTTATCATG TTAACTTTTGTCCTTAAGACATATGTTAA AAAACTCAAA
MTR_5g083030	-	1545	6.75e-11	AAGGGTCTTG TTAATGAGTGTCTTCAGAGCACTTTTTAA GCATTCCACA
MTR_3g116430	-	487	8.46e-11	TTTAATCTTC TTATTAAGTGCTCTAAGAGCACTTGTTAG CATTTACCAT

Block Diagrams

The height of the motif "block" is proportional to -log(p-value), truncated at the height for a motif with a p-value of 1e-10.
Click on any row to highlight sequence in all motifs. Mouse over the center of the motif blocks to see more information.

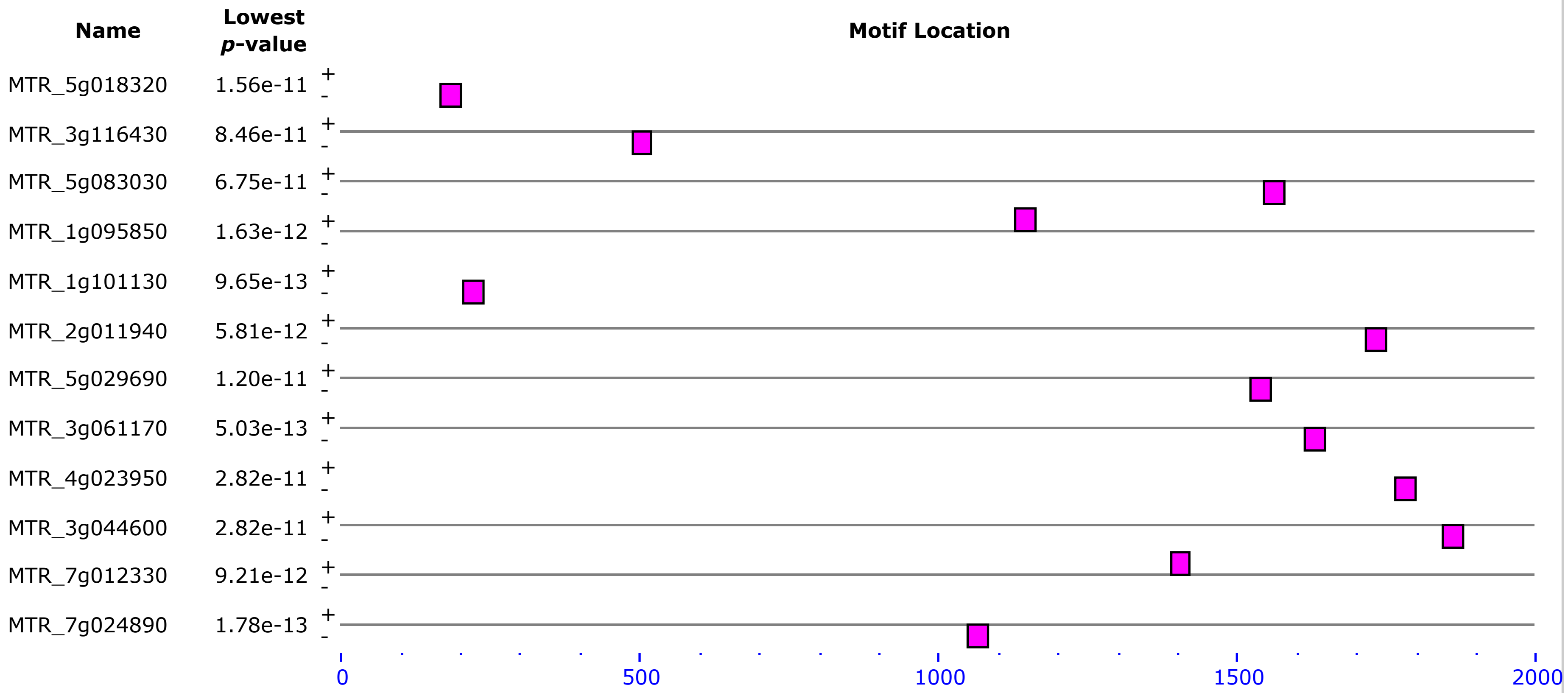

Time 29667.2 secs.

MOTIF 5

Previous Next Top

Summary

E-value	2.4e-005
Width	22
Sites	16

show more

Sequence Logo

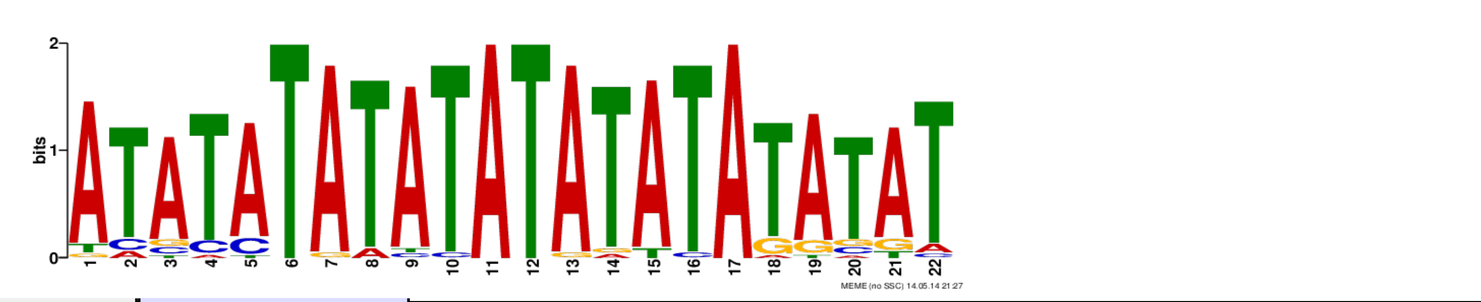

Standard Reverse Complement

Download LOGO Orientation: standard SSC: off Format: web (png) Width: 22 cm Height: 7.5 cm Download

Regular expression

ATATATATATATATATATATAT

Further Analysis

Submit this motif to MAST FIMO TOMTOM GOMO BLOCKS Mouse-over buttons for more information.

Data Formats

View the motif in PSPM Format PSSM Format BLOCKS Format FASTA Format Raw Format or

Hide

Sites

Click on any row to highlight sequence in all motifs.

Name	Strand	Start	*p*-value	Sites		
MTR_8g106040	+	423	7.24e-11	ACACACACAC	ATATATATATATATATATATAT	ATATATATAT
MTR_3g116430	+	882	7.24e-11	TTCAGACATC	ATATATATATATATATATATAT	ATATATATAT
MTR_8g068050	+	676	7.24e-11	GTTAAGTATT	ATATATATATATATATATATAT	ATTATTAAAT
MTR_5g018320	+	128	7.24e-11	TTGAGCCCCC	ATATATATATATATATATATAT	ATATATATAT
MTR_7g098160	+	423	7.24e-11	GCGTGTGTGT	ATATATATATATATATATATAT	GGAGTTTCTA
MTR_7g076920	+	1699	7.24e-11	TAAAACTAAA	ATATATATATATATATATATAT	ATATATATAT
MTR_2g081600	+	22	7.24e-11	CATTTCCTTA	ATATATATATATATATATATAT	ATATATATAT
MTR_7g086610	-	1608	1.30e-09	AGTGGTACCA	TTATATATATATATATATATAT	TGCACCTTTA
MTR_2g007970	-	1960	5.46e-09	TTCAATATAT	ATTTCTATATATATATATATAT	CACGTACAAT
MTR_5g022560	-	361	1.35e-08	AGAAGGGATT	GTGTCTATATATATATATATAT	TAACGGAAGA
MTR_7g073430	+	1965	1.46e-08	TAGTATACCA	ACCCCTATATATATATATATAT	TATAAGAGTT
MTR_2g011940	-	1455	1.63e-08	TTTTTTGAAC	AAGCATATATATATATATATAT	TATATACACA
MTR_7g088790	+	1940	9.26e-08	AAATCATTCC	ATCTCTGTCTATATATATATAT	AGAGTGCCAA
MTR_1g021110	+	944	1.54e-07	AAGTAAGCTC	ACATATATTTATATTTATATGT	TAAAAAATTA
MTR_5g018570	+	1262	2.11e-07	AACTTGTAAC	AAACATAAACATATATATATAT	TTCTATCATT
MTR_7g086220	-	1545	2.11e-07	ACCCCATAAG	ATAATTATATATATATATATAA	CAAAGAATTA

Block Diagrams

The height of the motif "block" is proportional to -log(p-value), truncated at the height for a motif with a p-value of 1e-10.
Click on any row to highlight sequence in all motifs. Mouse over the center of the motif blocks to see more information.

Name	Lowest	Motif Location

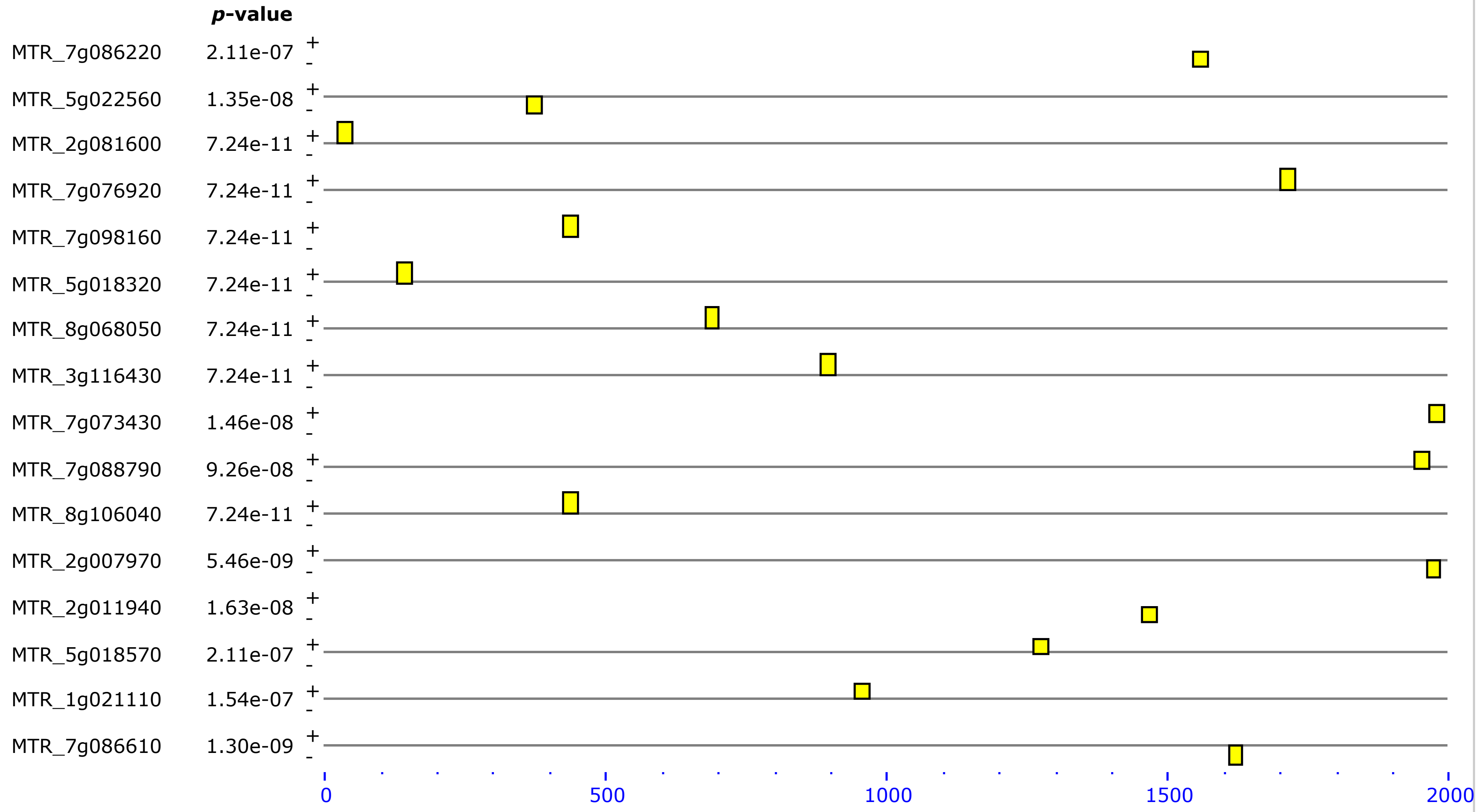

Time 36759.6 secs.

MOTIF 6

Previous Next Top

Summary

E-value	1.4e-004
Width	22
Sites	28

show more

Sequence Logo

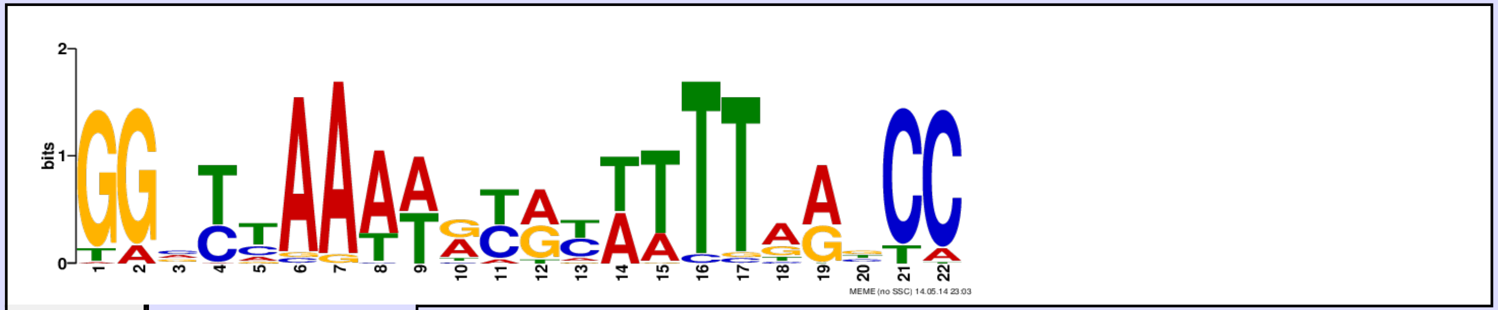

Standard | Reverse Complement

Download LOGO Orientation: standard SSC: off Format: web (png) Width: 22 cm Height: 7.5 cm Download

Regular expression

GG[CAG][TC][TC]AA[AT][AT][AG][TC][AG][CT][TA][TA]TT[AG][AG][GTC]CC

Further Analysis

Submit this motif to MAST FIMO TOMTOM GOMO BLOCKS Mouse-over buttons for more information.

Data Formats

View the motif in PSPM Format PSSM Format BLOCKS Format FASTA Format Raw Format or Hide

Sites

Click on any row to highlight sequence in all motifs.

Name	Strand	Start	*p*-value	Sites
MTR_6g012970	-	504	1.09e-10	AAAAGATAGG GGGCCAAAAGTGCAATTAAGCC TTAATATTTT
MTR_3g061170	-	1051	1.22e-09	CAAAACACGA GGACCAAAAGTGTAATTAAGCC TAAAAAGAAA
MTR_4g091020	-	1378	1.42e-09	CTAATTACAA GGATCAAATACATATTTAAGCC TAAATATTAC
MTR_3g115940	+	458	1.64e-09	TATCATTAAT GGGTTAATTGTGTTTTTAGTCC TTATAAATAT
MTR_3g034180	-	1280	2.44e-09	ATAATTACAG GGATGAAAAGCATATTTAAGCC AAAAATAAAA
MTR_1g061540	-	1450	2.44e-09	GAAAGATAGG GGTCCAAAAGTGCAATTAAGCC TTAAACATTT
MTR_5g083030	+	687	2.44e-09	ATTAGATAGG GGTCCAAAAGTGCAATTAAGCC CGGTTAATAT
MTR_7g116650	-	1379	4.62e-09	ATAAATTTAA GGGTTAATTATGTTTTTGGTCC CTATAAATAT
MTR_4g069810	-	554	1.19e-08	TAAAAGAGAC GGACAAGAAGTGCATTTAAGCC GGAAAACTAT
MTR_1g021110	-	814	1.49e-08	ATATTTATAA GGACTAGAAACATATTTAACCC TATTATTAAT
MTR_1g017910	-	1457	3.48e-08	AATATTTTCC GACTTCAATGCGCATTTGGTCC TCTAACTTTG
MTR_7g024890	-	214	3.85e-08	ATATTTATAG GGACTAAAAACTTAATTAACCC AAAATTAAAT
MTR_3g116430	-	14	4.72e-08	GTTAAATCTA GGCTAAATATCACTTTTGGTCC TTTTAGTTTA
MTR_3g082250	+	584	5.21e-08	TTTTAAATTA GGCTAAATTATATTTTTGGTCC TTTAATTATT
MTR_6g069050	+	1105	6.34e-08	TTATTTATTA GGCTTGATTGCAGTTTTGACCC CCTAAGTATG
MTR_5g016320	-	41	7.66e-08	CTAATTGCAA GGATGAAATACATATTTAAACC AAAAATAAAT
MTR_7g034740	+	1528	9.26e-08	GTATTTGAAG TGGCTAAAAACAAATTTAAGCC TTGTAATAAT
MTR_1g098300	+	1211	1.75e-07	GAAAGTGCAA GAACCAATAATATATTTAAGCC TTTATAAAAT
MTR_2g062840	+	1371	2.47e-07	ATTTTTTTTA GGGTTAATATCTCTTTTCGTCC CTGTAATATT
MTR_4g081190	-	87	2.47e-07	AAAAAAAAAA GGCTAAACTACAGTTTTGGCCC TTATGTTTGC
MTR_8g087780	-	1294	4.07e-07	AATTTTATAA GGACTAAAAATTTATTTAACTC TAAAACATAT
MTR_3g089170	+	1587	5.61e-07	CAAGTTTTCC TGCTTAAATGCTCATTTAAGCA TGTTTGAATA
MTR_8g039990	-	1621	6.55e-07	AAAAAACTAT GGCCAAATACCACTTTTTGGTC CTTTAAGTTT
MTR_4g086540	+	1513	9.60e-07	AAGTTGATGA TGACCAAAATTGCATTTAAGTC AAAATAAAAA
MTR_1g102230	-	1049	1.39e-06	ATTAATTATT TAGTTAATAGTGTTTTTCACCC ATGTAATATA
MTR_2g012790	+	254	1.49e-06	CACTCATTTT GGGATCAAAATGTTTTTTACCC TAAAGTTAAA

Name	Strand	Start	*p*-value	Sites
MTR_7g098040	+	1646	1.72e-06	CTCAGTGAAA GGTCTGAAAGCAAATTTAACTC AGAGTATTGT
MTR_4g023070	+	1323	3.40e-06	TTTATCTTGT GGTTCAGATGTACTTTTAATCT CAAAACCAAA

Block Diagrams

The height of the motif "block" is proportional to -log(p-value), truncated at the height for a motif with a p-value of 1e-10.
Click on any row to highlight sequence in all motifs. Mouse over the center of the motif blocks to see more information.

Name	Lowest *p*-value
MTR_4g091020	1.42e-09
MTR_7g034740	9.26e-08
MTR_8g087780	4.07e-07
MTR_7g116650	4.62e-09
MTR_4g081190	2.47e-07
MTR_1g098300	1.75e-07
MTR_5g016320	7.66e-08
MTR_6g012970	1.09e-10
MTR_2g012790	1.49e-06
MTR_7g098040	1.72e-06
MTR_4g069810	1.19e-08
MTR_3g115940	1.64e-09
MTR_1g017910	3.48e-08
MTR_3g116430	4.72e-08
MTR_5g083030	2.44e-09

Motif Location

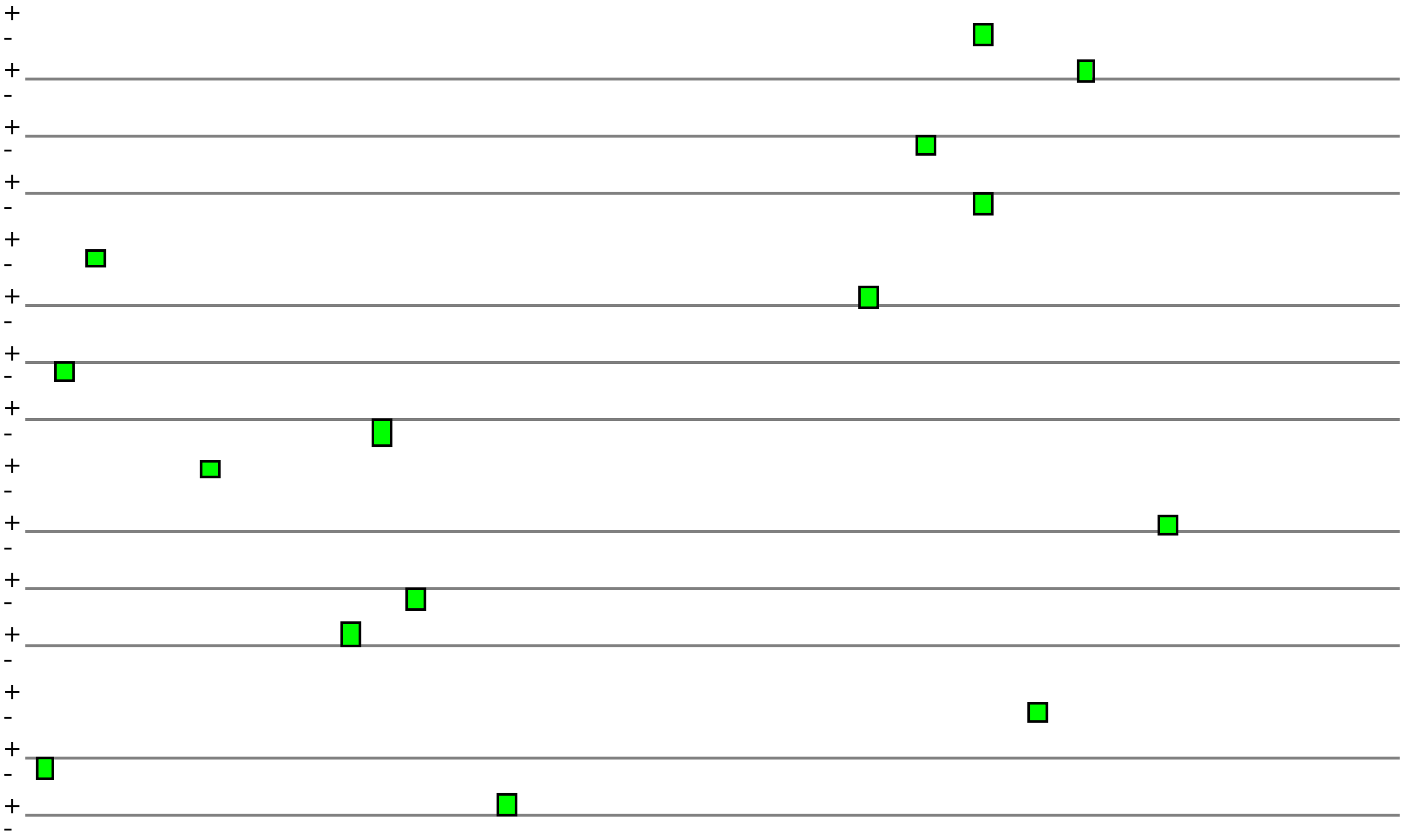

Name	Lowest *p*-value
MTR_1g061540	2.44e-09
MTR_3g082250	5.21e-08
MTR_2g062840	2.47e-07
MTR_4g023070	3.40e-06
MTR_6g069050	6.34e-08
MTR_1g102230	1.39e-06
MTR_4g086540	9.60e-07
MTR_3g089170	5.61e-07
MTR_8g039990	6.55e-07
MTR_1g021110	1.49e-08
MTR_3g061170	1.22e-09
MTR_3g034180	2.44e-09
MTR_7g024890	3.85e-08

Motif Location

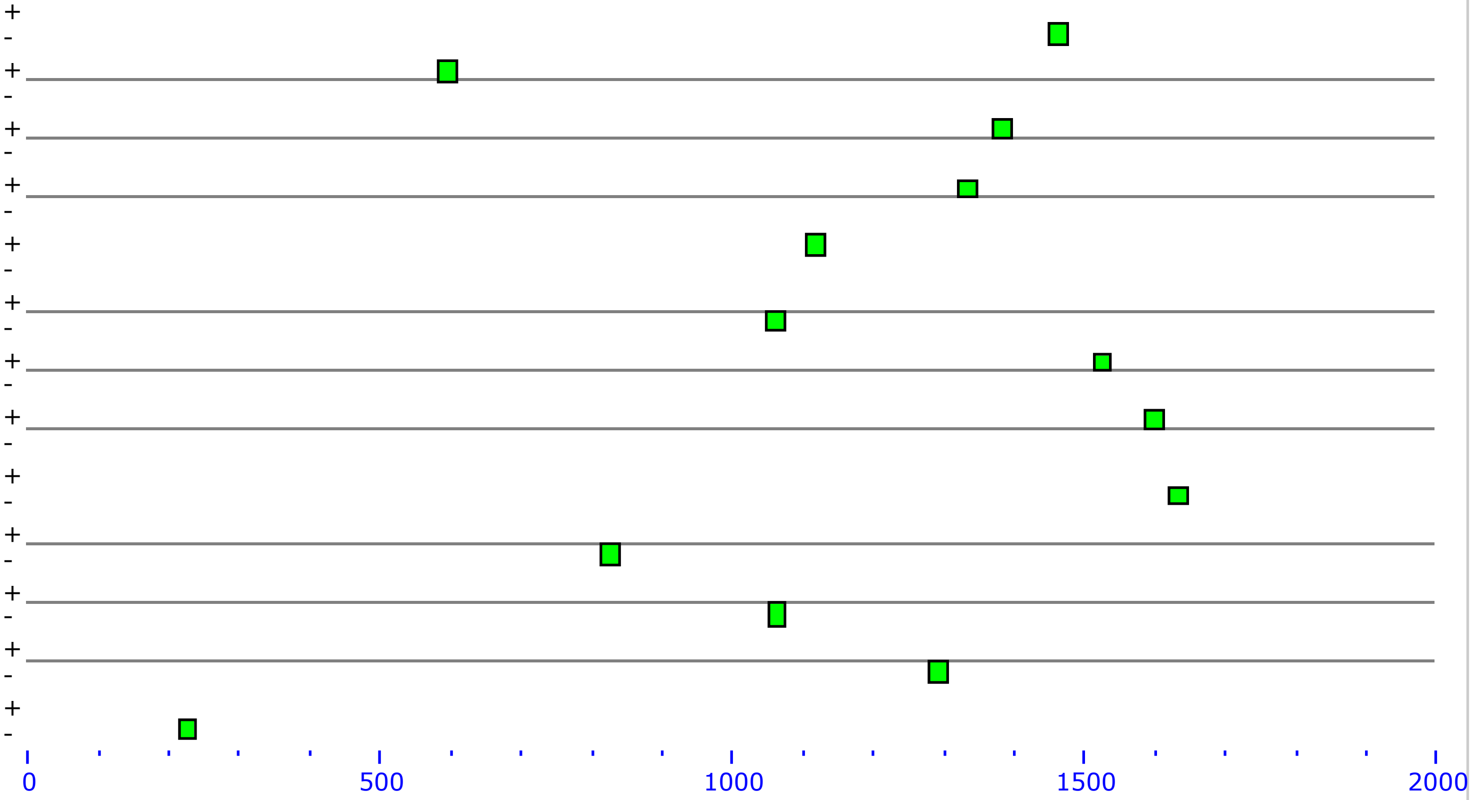

Time 42529.1 secs.

MOTIF 7

Previous Next Top

Summary

E-value	1.1e+000
Width	6

Sequence Logo

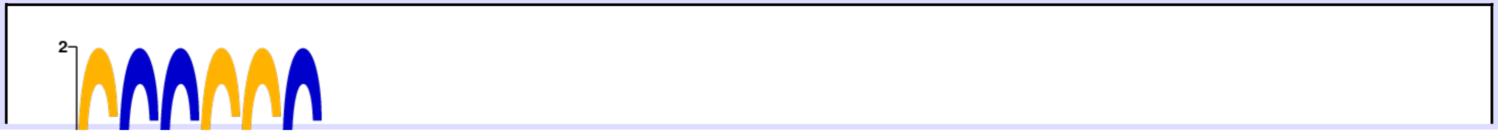

Sites 30
show more

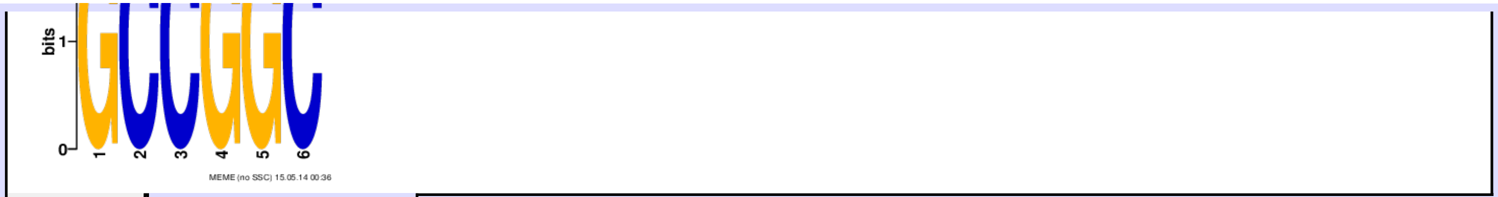

Standard | Reverse Complement

Download LOGO [?] Orientation: standard SSC: off Format: web (png) Width: 6 cm Height: 7.5 cm Download

Regular expression [?]

GCCGGC

Further Analysis [?]

Submit this motif to MAST [?] FIMO [?] TOMTOM [?] GOMO [?] BLOCKS [?] Mouse-over buttons for more information.

Data Formats [?]

View the motif in ○ PSPM Format [?] ○ PSSM Format [?] ○ BLOCKS Format [?] ○ FASTA Format [?] ○ Raw Format [?] or ● Hide

Sites [?]

Click on any row to highlight sequence in all motifs.

Name	Strand	Start	*p*-value	Sites [?]		
MTR_5g018570	+	692	1.33e-05	ACAAAAGGTT	GCCGGC	GGGAGTTGAA
MTR_4g122750	+	1591	1.33e-05	GCCCCTGTAG	GCCGGC	TTGACCTATT
MTR_2g096180	+	1685	1.33e-05	CCGCGTTATC	GCCGGC	AACTCGTCGG
MTR_8g074530	+	41	1.33e-05	ATTAATAAAG	GCCGGC	ACAGTTTGGA

Name	Strand	Start	*p*-value	Sites
MTR_3g100980	+	1608	1.33e-05	AATTTCAAAT GCCGGC AGCAATGAAT
MTR_2g081630	+	1861	1.33e-05	ATCTCAATTA GCCGGC AAAAGCAGGC
MTR_4g053630	+	1864	1.33e-05	AAACATTTAA GCCGGC AACTGATTTT
MTR_4g133890	+	1577	1.33e-05	AGAAGGATGA GCCGGC TTGGCCGGCC
MTR_2g025170	+	576	1.33e-05	GAAGTGAAGA GCCGGC AATAAGTGTT
MTR_4g130270	+	1877	1.33e-05	CCATCTTTCA GCCGGC TATCACAATT
MTR_8g068050	+	1206	1.33e-05	TGAGGATATA GCCGGC ACCAGCATTA
MTR_3g115940	+	1723	1.33e-05	CTTCCAAAGG GCCGGC TTAAATTTTC
MTR_1g101370	+	350	1.33e-05	AAAAACTAAG GCCGGC TATTTTTTCA
MTR_4g129010	+	1826	1.33e-05	AGTTTAGTTA GCCGGC CAAGGTCGAT
MTR_2g089100	+	963	1.33e-05	ACAAGAGTGG GCCGGC TAGTATACCC
MTR_5g018320	+	398	1.33e-05	AGGTTGGCAA GCCGGC TAAGGATCCA
MTR_7g098040	+	1309	1.33e-05	TGTTGTGCCT GCCGGC TGCTTATGAT
MTR_5g016320	+	1646	1.33e-05	AATTCTTTAA GCCGGC ACACATCACT
MTR_1g098300	+	1882	1.33e-05	TTCCAACTGT GCCGGC CATGGTCGGC
MTR_7g115050	+	1847	1.33e-05	CAACGAATTA GCCGGC CTTTAGAACC
MTR_7g098160	+	256	1.33e-05	TGCCCGTCTT GCCGGC TGCATATGAT
MTR_8g087810	+	613	1.33e-05	CTATTATTTA GCCGGC TAATTGATAA
MTR_3g104820	+	1883	1.33e-05	GAAATTTGGT GCCGGC CATAGCTTTG
MTR_1g079120	+	739	1.33e-05	CAAATTCTTG GCCGGC TTGTTTAATT
MTR_8g022270	+	1804	1.33e-05	CTATGCCAAA GCCGGC CATATAGCAT
MTR_7g034740	+	1652	1.33e-05	ATATTACTTA GCCGGC AACTCTTCTT
MTR_2g081600	+	1861	1.33e-05	TTCTCAATTA GCCGGC AAAAGCAGGC
MTR_4g091010	+	1892	1.33e-05	CTGCCCTTGA GCCGGC ATTCTTCTTA
TC94453	+	201	1.33e-05	ACTCGTGTGT GCCGGC TGCAACCACT
TC104091	+	1515	1.33e-05	TCTAGTTATA GCCGGC CTCTATAGTA

Block Diagrams

The height of the motif "block" is proportional to -log(p-value), truncated at the height for a motif with a p-value of 1e-10.
Click on any row to highlight sequence in all motifs. Mouse over the center of the motif blocks to see more information.

Name	Lowest *p*-value
TC104091	1.33e-05
TC94453	1.33e-05
MTR_4g091010	1.33e-05
MTR_2g081600	1.33e-05
MTR_7g034740	1.33e-05
MTR_8g022270	1.33e-05
MTR_1g079120	1.33e-05
MTR_3g104820	1.33e-05
MTR_8g087810	1.33e-05
MTR_7g098160	1.33e-05
MTR_7g115050	1.33e-05
MTR_1g098300	1.33e-05
MTR_5g016320	1.33e-05
MTR_7g098040	1.33e-05
MTR_5g018320	1.33e-05
MTR_2g089100	1.33e-05
MTR_4g129010	1.33e-05
MTR_1g101370	1.33e-05

Motif Location

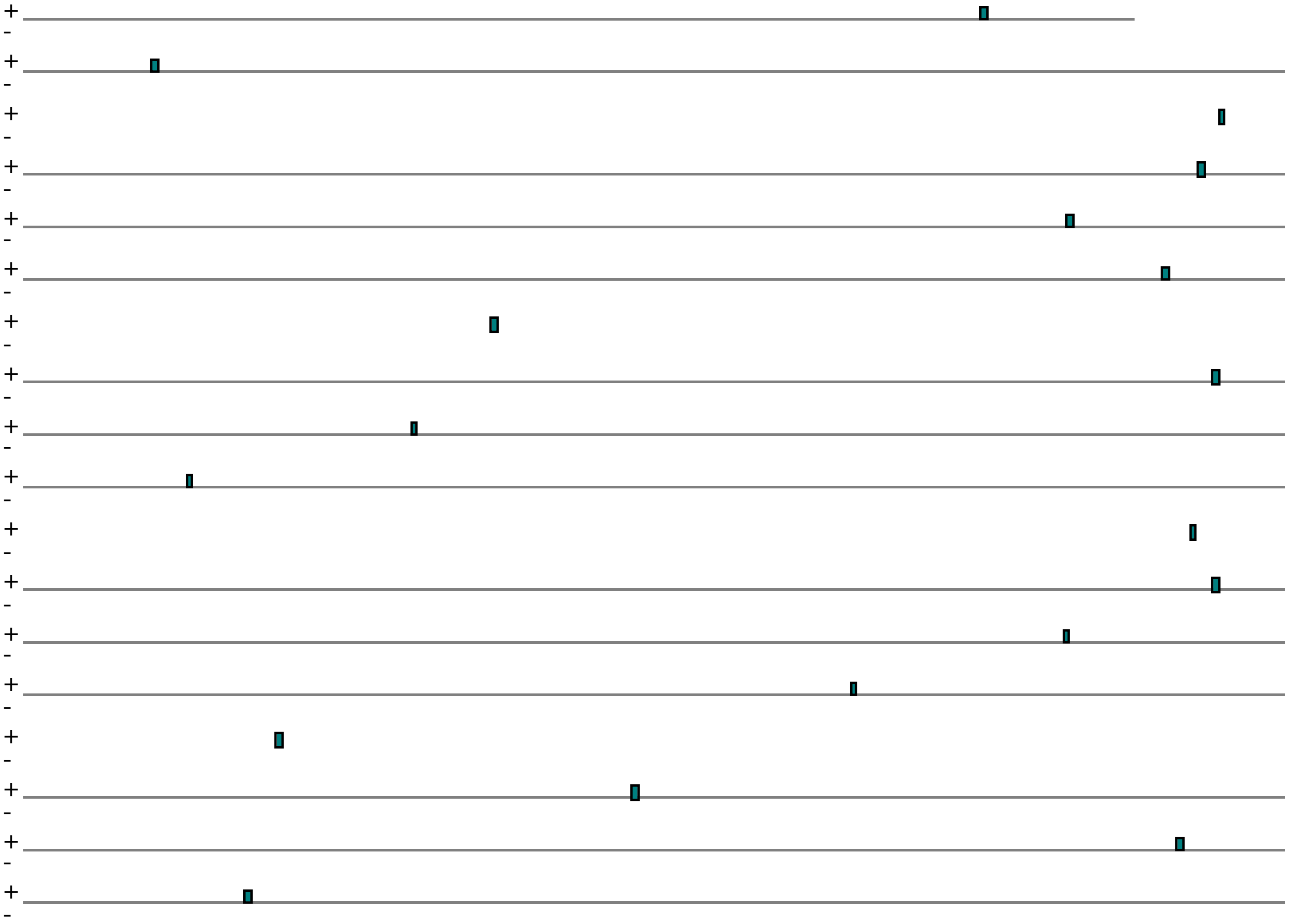

Name	Lowest *p*-value	Motif Location
MTR_3g115940	1.33e-05	
MTR_8g068050	1.33e-05	
MTR_4g130270	1.33e-05	
MTR_2g025170	1.33e-05	
MTR_4g133890	1.33e-05	
MTR_4g053630	1.33e-05	
MTR_2g081630	1.33e-05	
MTR_3g100980	1.33e-05	
MTR_8g074530	1.33e-05	
MTR_2g096180	1.33e-05	
MTR_4g122750	1.33e-05	
MTR_5g018570	1.33e-05	

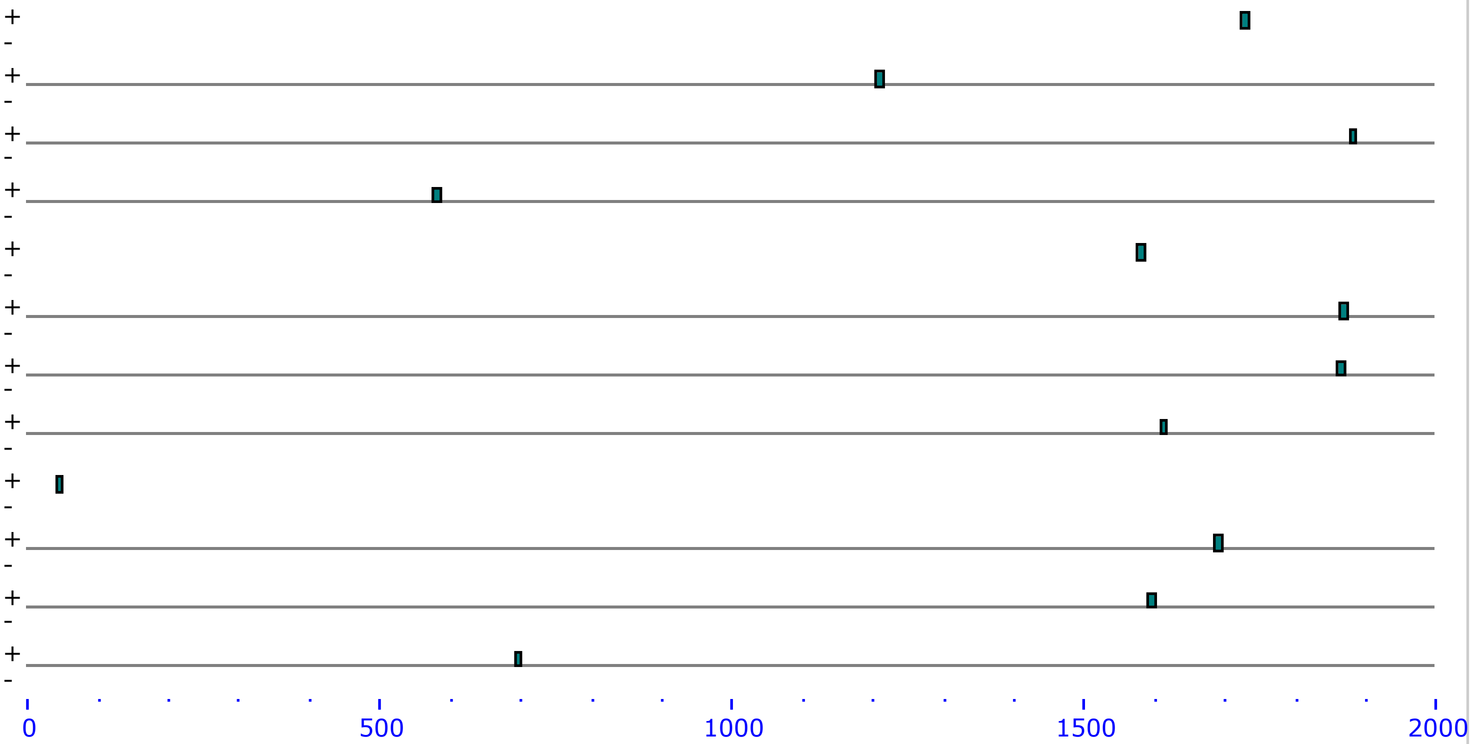

Time 48121.1 secs.

MOTIF 8

Previous Next Top

Summary ?

E-value	5.5e+004
Width	29
Sites	6

show more

Sequence Logo ?

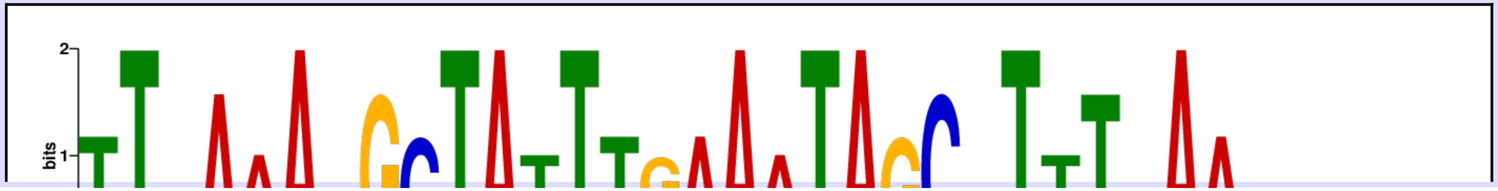

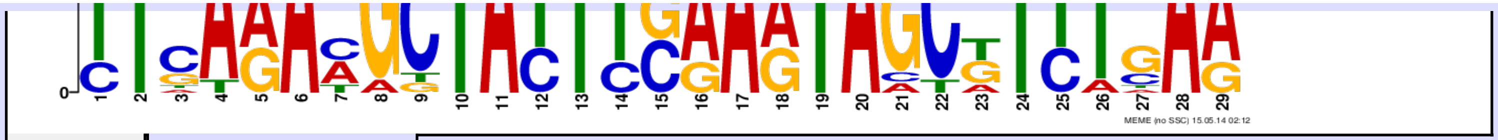

Standard | Reverse Complement

Download LOGO [?] Orientation: standard SSC: off Format: web (png) Width: 29 cm Height: 7.5 cm Download

Regular expression [?]

[TC]T[CG]A[AG]A[AC]GCTA[TC]T[TC][GC][AG]A[AG]TAGC[GT]T[TC]T[GC]A[AG]

Further Analysis [?]

Submit this motif to MAST [?] FIMO [?] TOMTOM [?] GOMO [?] BLOCKS [?] Mouse-over buttons for more information.

Data Formats [?]

View the motif in ○ PSPM Format [?] ○ PSSM Format [?] ○ BLOCKS Format [?] ○ FASTA Format [?] ○ Raw Format [?] or ● Hide

Sites [?]

Click on any row to highlight sequence in all motifs.

Name	Strand	Start	*p*-value		Sites [?]	
MTR_2g095800	+	1213	4.75e-15	AAGCTATAAG	CTCAAACGCTACTTGGAATAGCTTCTGAA	AAATAGCTTA
MTR_4g091020	+	565	4.75e-15	AAGCTATAAA	CTCAAACGCTACTTGGAATAGCTTCTGAA	AAACAGCTTA
MTR_2g098490	+	661	1.04e-14	AAGCTATAAG	CTCAAACGCTATTTGGAATAGCTTCTGAA	AAATAGCTTA
MTR_2g081600	+	301	7.60e-13	CAGCTATAAG	TTAAAAAGCTACTTGAAATAGCTTTTCAA	AAAACGCTAT
MTR_4g015650	-	841	5.53e-12	AATTTAATGT	TTGTGACGGTACTTCAAGTAGCGTTTAAA	TTTATAATTT

Name	Strand	Start	*p*-value	Sites
MTR_7g012070	-	1463	4.58e-11	ATAACATATT TTGAGATATTATTTCAAATAGCATTTGAA CTTATTTTCT

Block Diagrams

The height of the motif "block" is proportional to -log(p-value), truncated at the height for a motif with a p-value of 1e-10.
Click on any row to highlight sequence in all motifs. Mouse over the center of the motif blocks to see more information.

Name	Lowest *p*-value
MTR_2g098490	1.04e-14
MTR_4g091020	4.75e-15
MTR_2g081600	7.60e-13
MTR_7g012070	4.58e-11
MTR_2g095800	4.75e-15
MTR_4g015650	5.53e-12

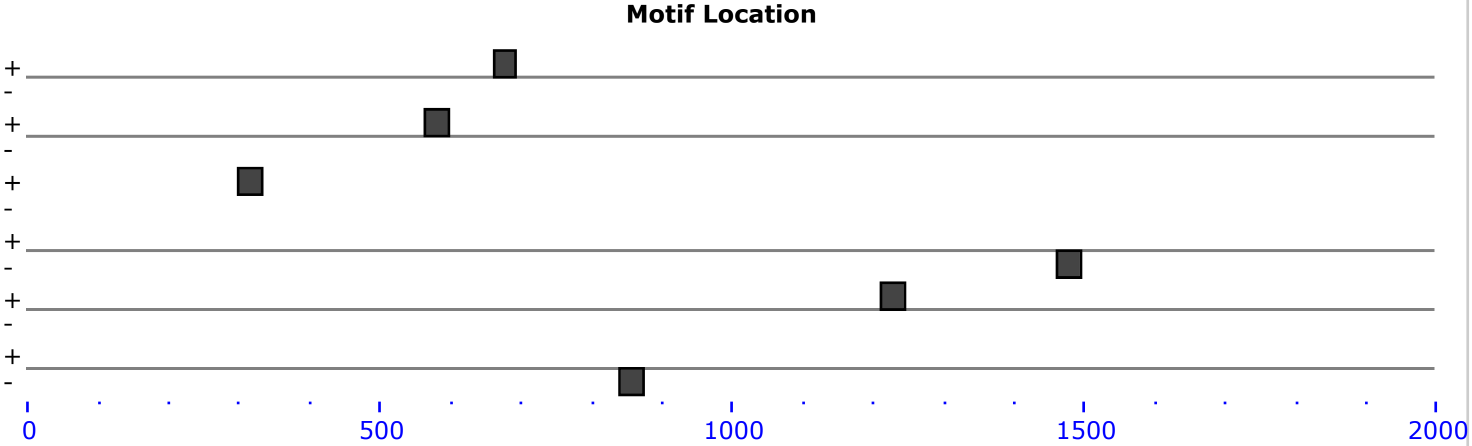

Time 53883.1 secs.

Motif 9

Previous Next Top

Summary

E-value	3.4e+005
Width	18
Sites	3

show more

Sequence Logo

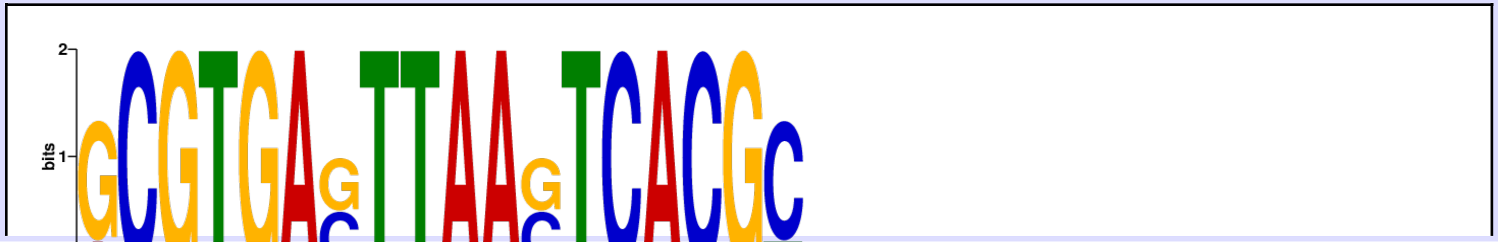

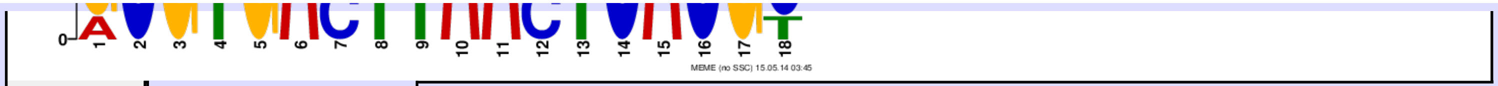

Standard | Reverse Complement

Download LOGO [?] Orientation: standard SSC: off Format: web (png) Width: 18 cm Height: 7.5 cm Download

Regular expression [?]

GCGTGA[CG]TTAA[CG]TCACGC

Further Analysis [?]

Submit this motif to MAST [?] FIMO [?] TOMTOM [?] GOMO [?] BLOCKS [?] Mouse-over buttons for more information.

Data Formats [?]

View the motif in ○ PSPM Format [?] ○ PSSM Format [?] ○ BLOCKS Format [?] ○ FASTA Format [?] ○ Raw Format [?] or ● Hide

Sites [?]

Click on any row to highlight sequence in all motifs.

Name	Strand	Start	*p*-value	Sites [?]		
MTR_5g017550	+	885	6.16e-12	TTTTACACAT	GCGTGACTTAAGTCACGC	TGCATAGTTA
MTR_4g113820	+	659	6.16e-12	AAATATCCAT	GCGTGAGTTAACTCACGC	AAGTGTTAAT
MTR_4g124790	+	1587	3.39e-11	TTTTGTACTA	ACGTGAGTTAAGTCACGC	AACATATGTT

Block Diagrams [?]

The height of the motif "block" is proportional to -log(p-value), truncated at the height for a motif with a p-value of 1e-10.

Click on any row to highlight sequence in all motifs. Mouse over the center of the motif blocks to see more information.

Name	Lowest *p*-value	Motif Location
MTR_4g113820	6.16e-12	
MTR_4g124790	3.39e-11	
MTR_5g017550	6.16e-12	

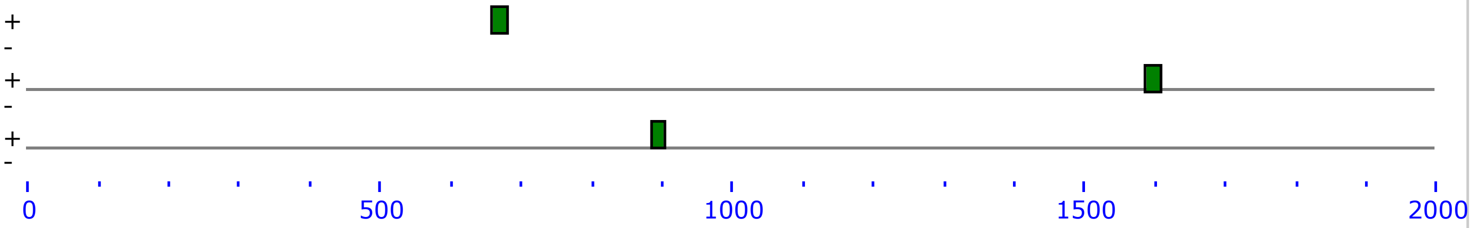

Time 59443.7 secs.

Motif 10

Previous Top

Summary

E-value	2.8e+005
Width	30
Sites	6

show more

Sequence Logo

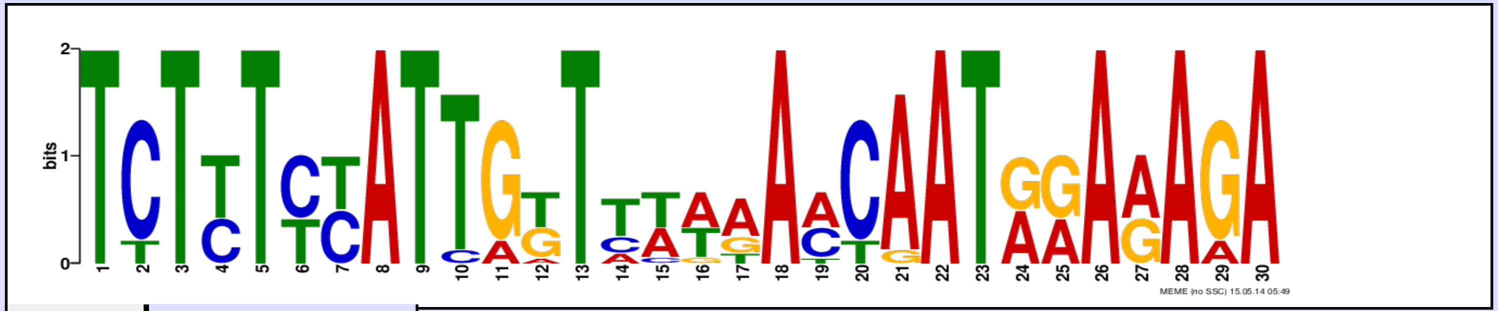

Standard | Reverse Complement

Download LOGO Orientation: standard SSC: off Format: web (png) Width: 30 cm Height: 7.5 cm Download

Regular expression

TCT[TC]T[CT][CT]ATTG[TG]T[TC][TA][AT][AG]A[AC]CAAT[AG][GA]A[AG]AGA

Further Analysis

Submit this motif to MAST FIMO TOMTOM GOMO BLOCKS Mouse-over buttons for more information.

Data Formats

View the motif in PSPM Format PSSM Format BLOCKS Format FASTA Format Raw Format or Hide

Sites

Click on any row to highlight sequence in all motifs.

Name	Strand	Start	*p*-value	Sites		
MTR_4g113820	-	224	1.93e-14	TTCTTTTTTC	TCTCTCCATTGTTTTTAACCAATAAAAAGA	GAGAAAAACA
MTR_8g068300	+	842	4.05e-14	CTTTTTTCTC	TCTTTTCATTGGTAAAAAACAATGGAGAGA	GAAAAAGGAA
MTR_2g081630	+	235	1.82e-13	TTTTTTTCTC	TCTCTCTATTGTTTTGGACCAATAAAAAGA	GAGAAAATAC
MTR_2g035320	+	1631	2.00e-13	TTCTTTTTTC	TCTCTCCATTATTTTTGACCAATGAAAAGA	GAGAAAAGAA
MTR_5g022560	-	1146	2.19e-12	CCTTTTCCTC	TTTTTCTATTGATCAAAAACAATGGAAAGA	GAAAAAGGAA
MTR_2g089100	+	635	4.41e-11	TTTTTTTTTT	TCTCTCTATCATTTTTTACCAATAAAAAAA	AATTGTTATA

Block Diagrams

The height of the motif "block" is proportional to -log(p-value), truncated at the height for a motif with a p-value of 1e-10.
Click on any row to highlight sequence in all motifs. Mouse over the center of the motif blocks to see more information.

Name	Lowest *p*-value	Motif Location
MTR_5g022560	2.19e-12	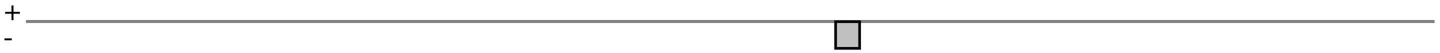

Name	Lowest *p*-value
MTR_2g089100	4.41e-11
MTR_4g113820	1.93e-14
MTR_2g081630	1.82e-13
MTR_8g068300	4.05e-14
MTR_2g035320	2.00e-13

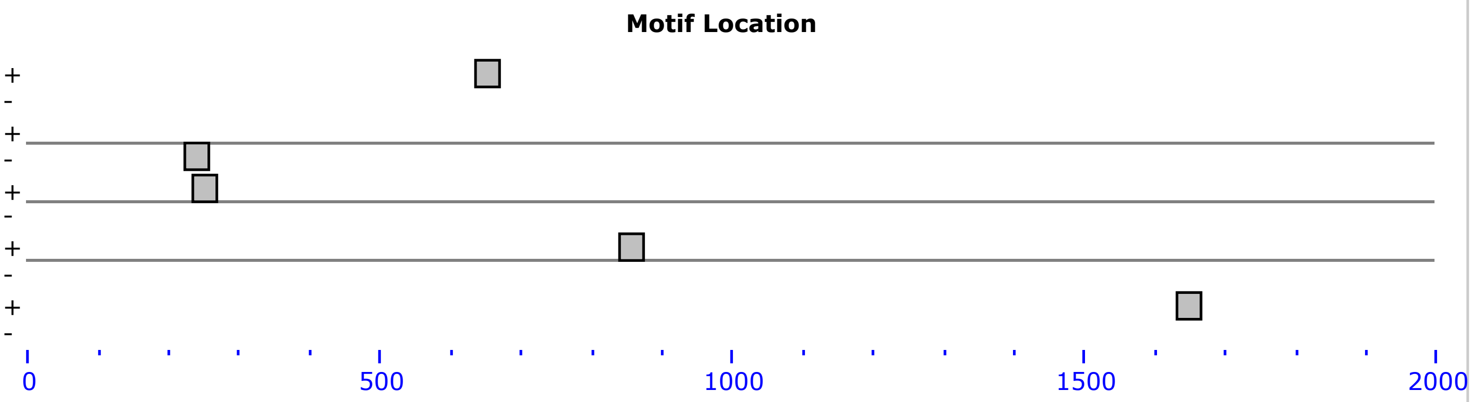

Time 65691.4 secs.

ALL MOTIFS

Top

Combined Block Diagrams

Non-overlapping sites with a *p*-value better than 0.0001.
The height of the motif "block" is proportional to -log(p-value), truncated at the height for a motif with a p-value of 1e-10.
Click on any row to highlight sequence in all motifs. The motif blocks have tool tips with more information.

Name	Combined *p*-value
TC104091	6.73e-01
TC94453	9.59e-01
MTR_1g105120	4.44e-06
MTR_7g086220	1.23e-01
MTR_7g076990	5.56e-01

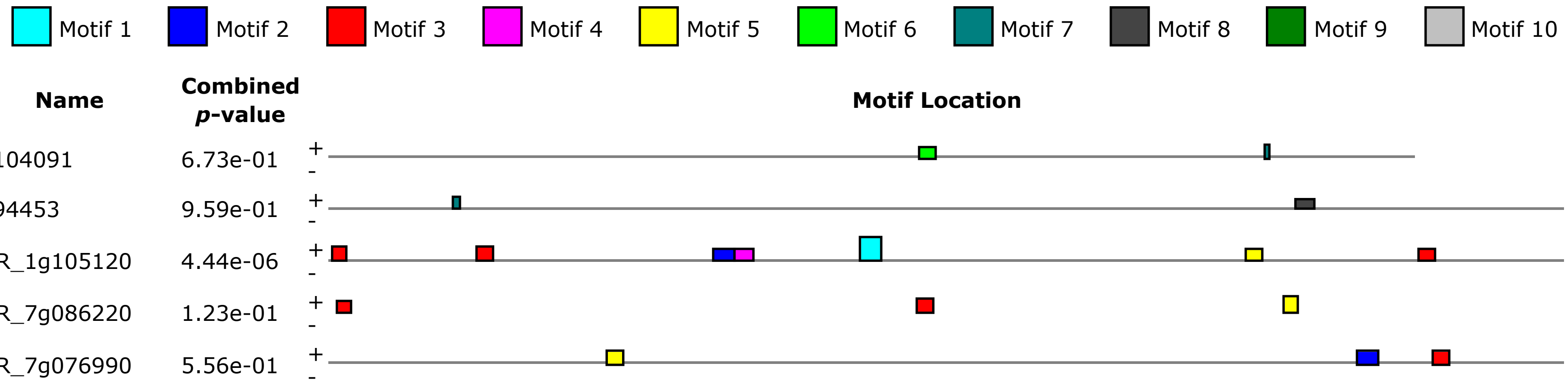

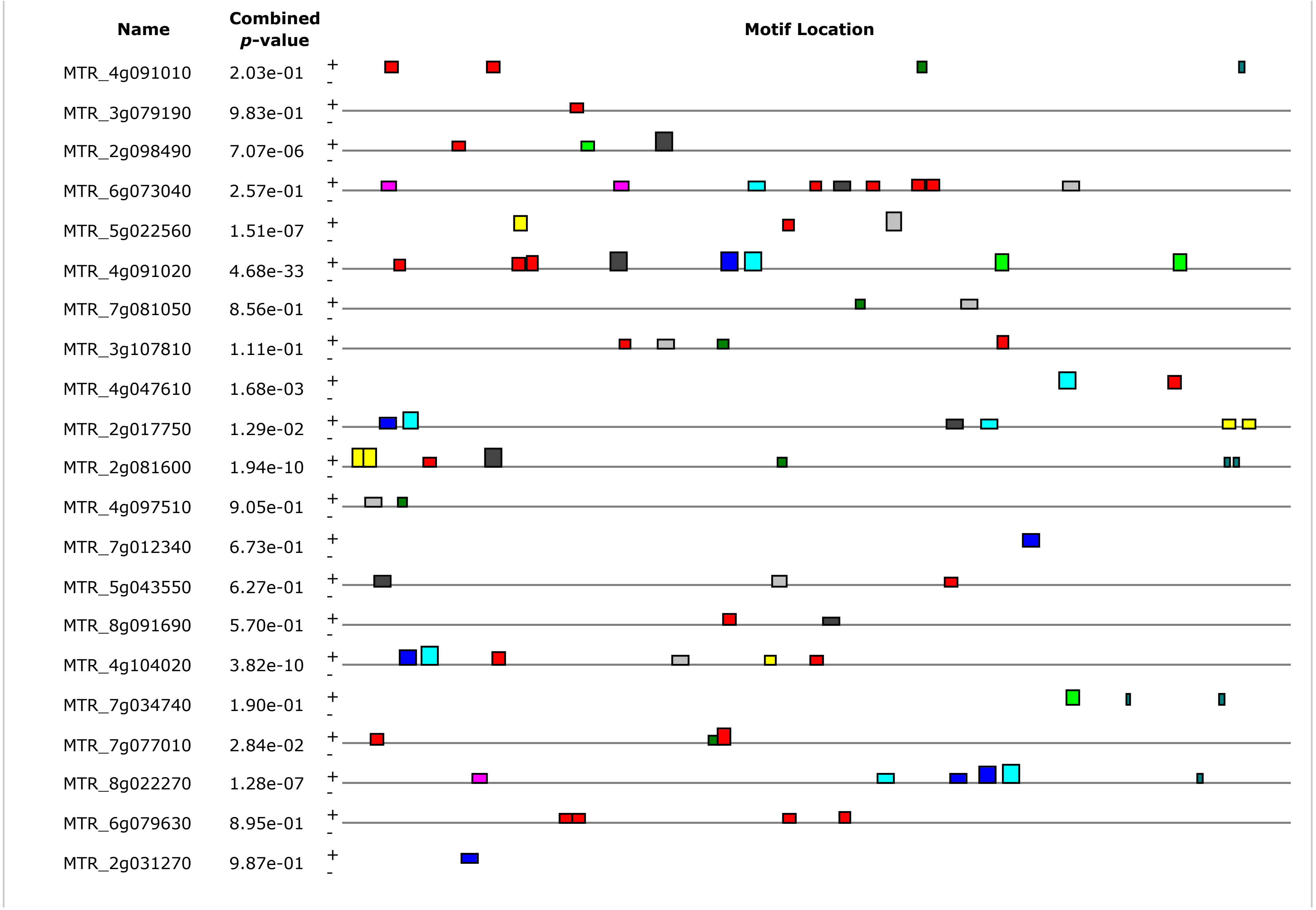
Name
Combined p-value
Motif Location
MTR_4g091010 2.03e-01
MTR_3g079190 9.83e-01
MTR_2g098490 7.07e-06
MTR_6g073040 2.57e-01
MTR_5g022560 1.51e-07
MTR_4g091020 4.68e-33
MTR_7g081050 8.56e-01
MTR_3g107810 1.11e-01
MTR_4g047610 1.68e-03
MTR_2g017750 1.29e-02
MTR_2g081600 1.94e-10
MTR_4g097510 9.05e-01
MTR_7g012340 6.73e-01
MTR_5g043550 6.27e-01
MTR_8g091690 5.70e-01
MTR_4g104020 3.82e-10
MTR_7g034740 1.90e-01
MTR_7g077010 2.84e-02
MTR_8g022270 1.28e-07
MTR_6g079630 8.95e-01
MTR_2g031270 9.87e-01

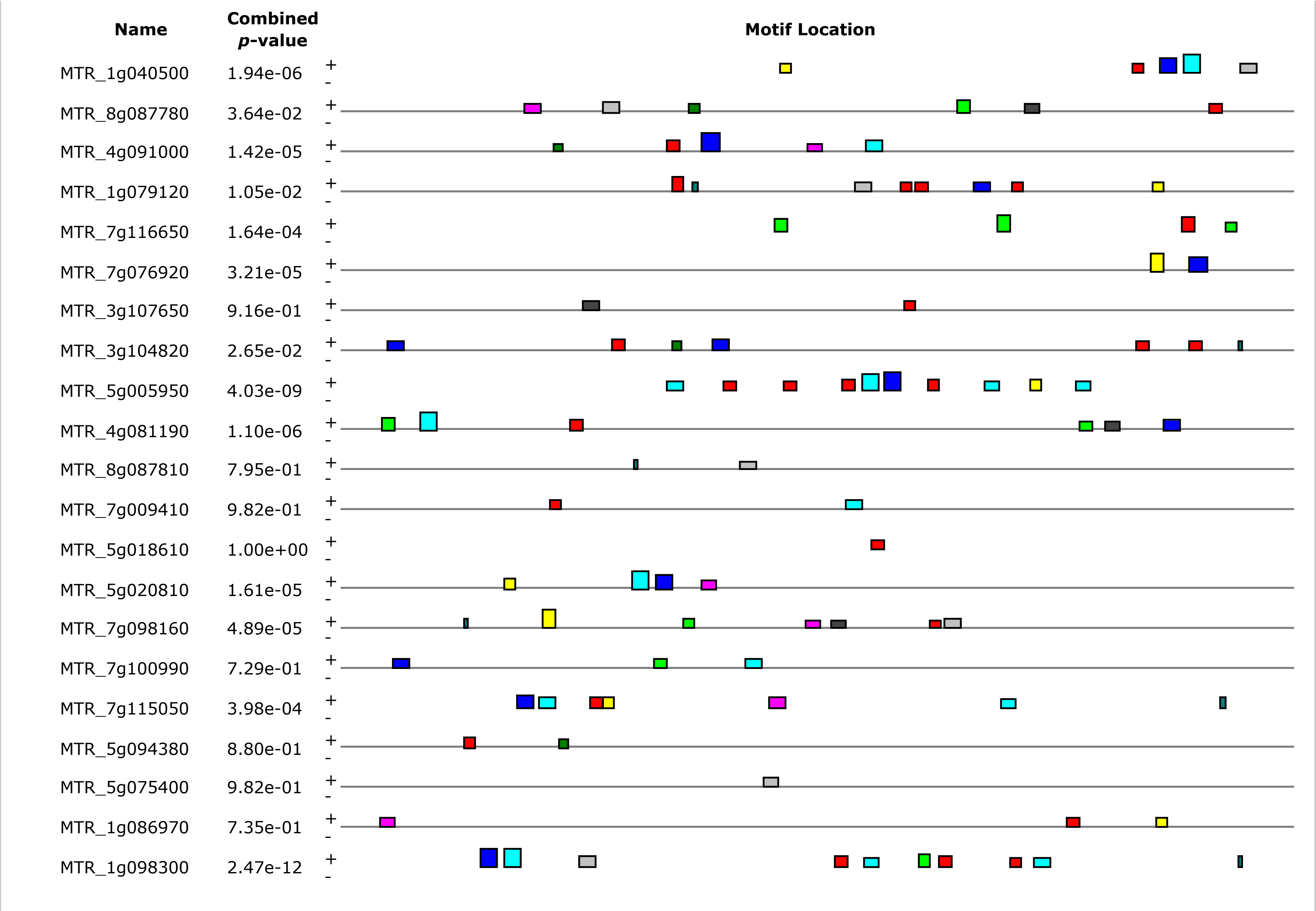
Name
Combined p-value
Motif Location
MTR_1g040500 1.94e-06
MTR_8g087780 3.64e-02
MTR_4g091000 1.42e-05
MTR_1g079120 1.05e-02
MTR_7g116650 1.64e-04
MTR_7g076920 3.21e-05
MTR_3g107650 9.16e-01
MTR_3g104820 2.65e-02
MTR_5g005950 4.03e-09
MTR_4g081190 1.10e-06
MTR_8g087810 7.95e-01
MTR_7g009410 9.82e-01
MTR_5g018610 1.00e+00
MTR_5g020810 1.61e-05
MTR_7g098160 4.89e-05
MTR_7g100990 7.29e-01
MTR_7g115050 3.98e-04
MTR_5g094380 8.80e-01
MTR_5g075400 9.82e-01
MTR_1g086970 7.35e-01
MTR_1g098300 2.47e-12

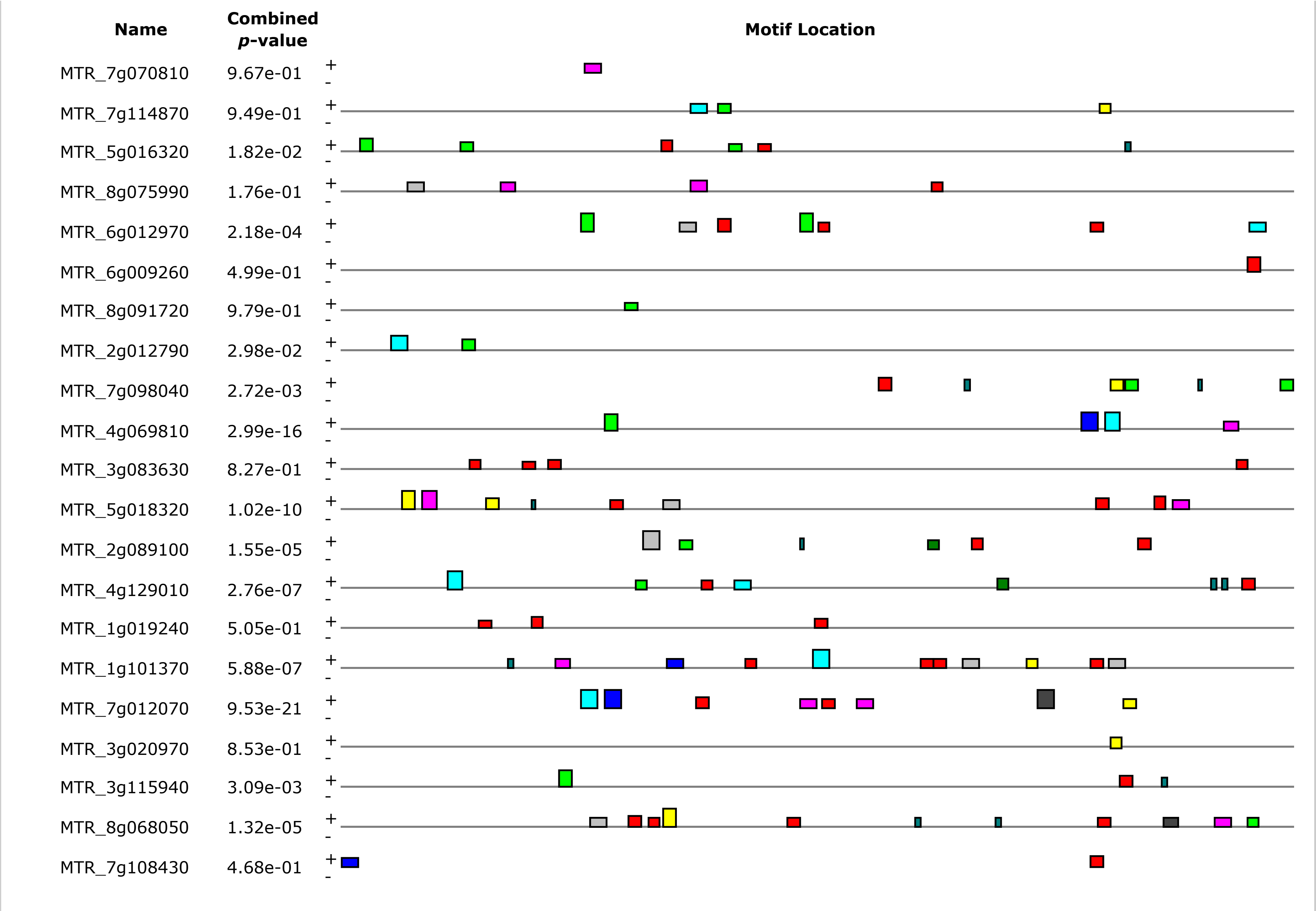
Name
Combined p-value
Motif Location
MTR_7g070810 9.67e-01
MTR_7g114870 9.49e-01
MTR_5g016320 1.82e-02
MTR_8g075990 1.76e-01
MTR_6g012970 2.18e-04
MTR_6g009260 4.99e-01
MTR_8g091720 9.79e-01
MTR_2g012790 2.98e-02
MTR_7g098040 2.72e-03
MTR_4g069810 2.99e-16
MTR_3g083630 8.27e-01
MTR_5g018320 1.02e-10
MTR_2g089100 1.55e-05
MTR_4g129010 2.76e-07
MTR_1g019240 5.05e-01
MTR_1g101370 5.88e-07
MTR_7g012070 9.53e-21
MTR_3g020970 8.53e-01
MTR_3g115940 3.09e-03
MTR_8g068050 1.32e-05
MTR_7g108430 4.68e-01

Name	Combined *p*-value
MTR_1g017910	3.16e-03
MTR_6g029180	8.13e-01
MTR_1g087030	4.81e-01
MTR_7g080180	4.14e-01
MTR_8g038220	3.65e-01
MTR_4g130270	2.03e-10
MTR_2g025170	1.24e-01
MTR_4g133890	1.96e-01
MTR_3g116430	1.94e-23
MTR_4g053630	3.53e-01
MTR_8g078300	9.29e-01
MTR_4g113820	1.85e-10
MTR_7g073430	1.60e-01
MTR_2g081630	4.16e-06
MTR_3g079320	6.63e-01
MTR_5g083030	4.10e-07
MTR_8g068300	1.89e-09
MTR_2g095800	4.14e-09
MTR_1g061540	4.42e-09
MTR_6g087680	3.80e-03
MTR_1g095850	9.34e-05

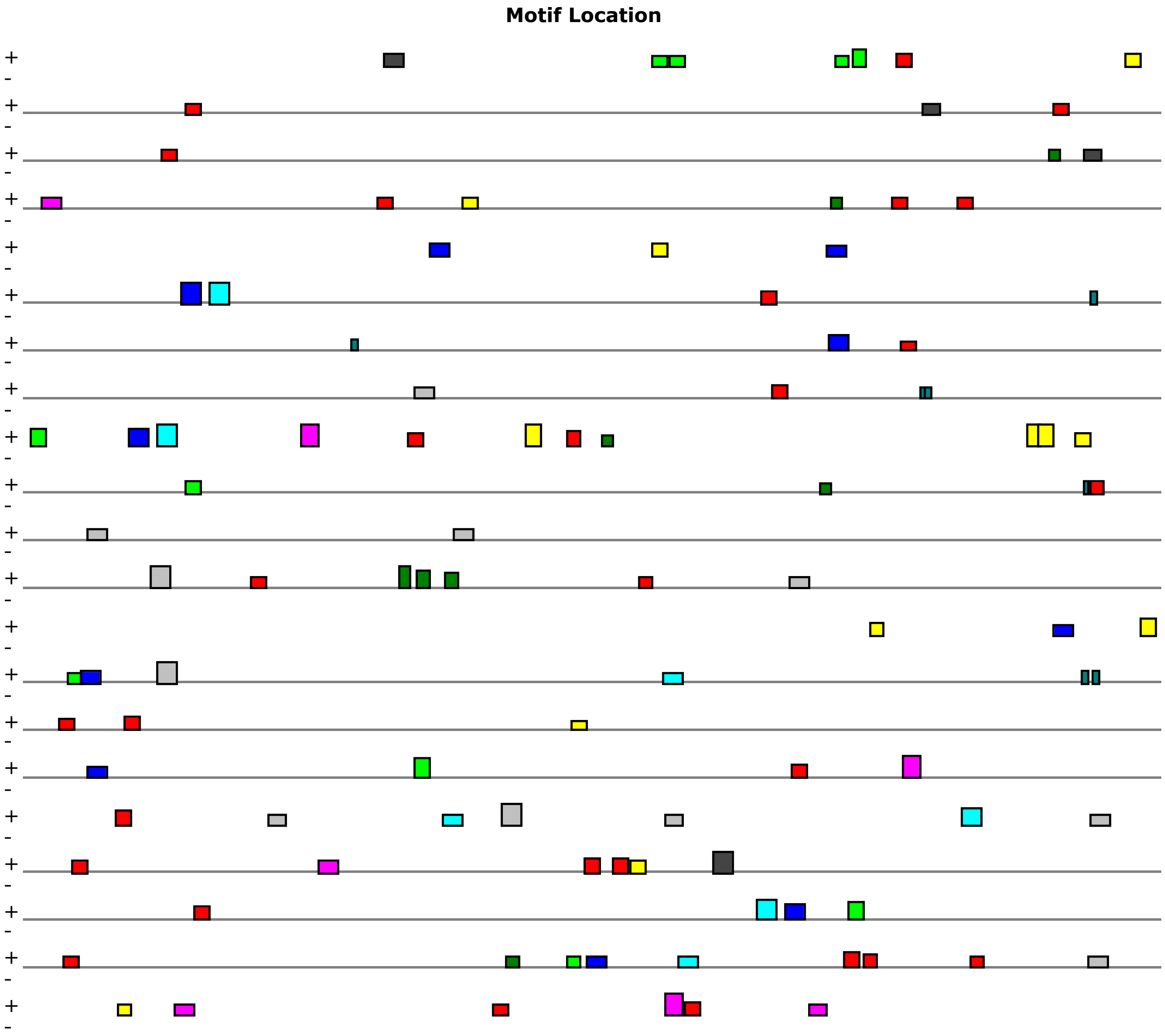

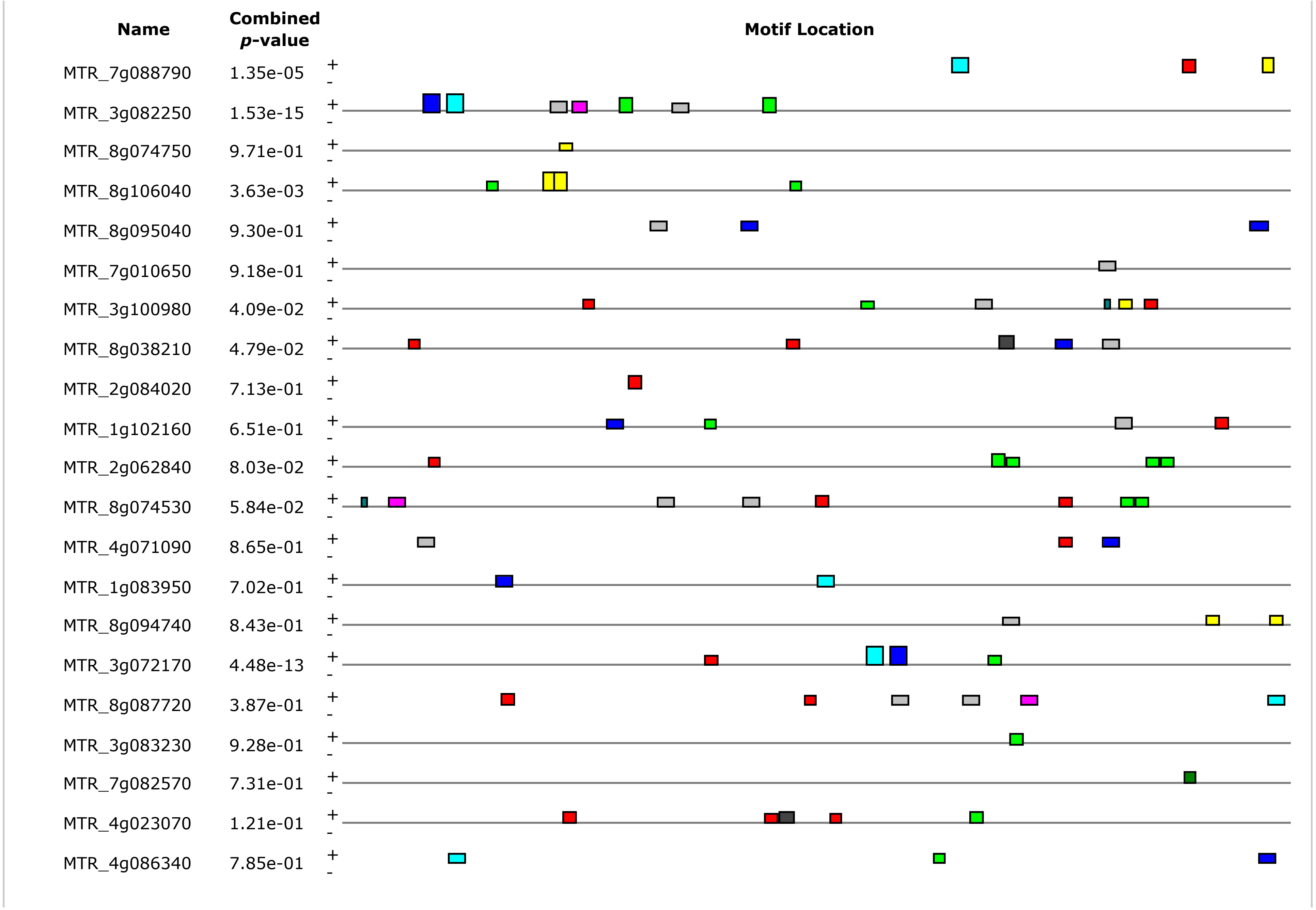

Name
Combined p-value
Motif Location
MTR_7g088790 1.35e-05
MTR_3g082250 1.53e-15
MTR_8g074750 9.71e-01
MTR_8g106040 3.63e-03
MTR_8g095040 9.30e-01
MTR_7g010650 9.18e-01
MTR_3g100980 4.09e-02
MTR_8g038210 4.79e-02
MTR_2g084020 7.13e-01
MTR_1g102160 6.51e-01
MTR_2g062840 8.03e-02
MTR_8g074530 5.84e-02
MTR_4g071090 8.65e-01
MTR_1g083950 7.02e-01
MTR_8g094740 8.43e-01
MTR_3g072170 4.48e-13
MTR_8g087720 3.87e-01
MTR_3g083230 9.28e-01
MTR_7g082570 7.31e-01
MTR_4g023070 1.21e-01
MTR_4g086340 7.85e-01

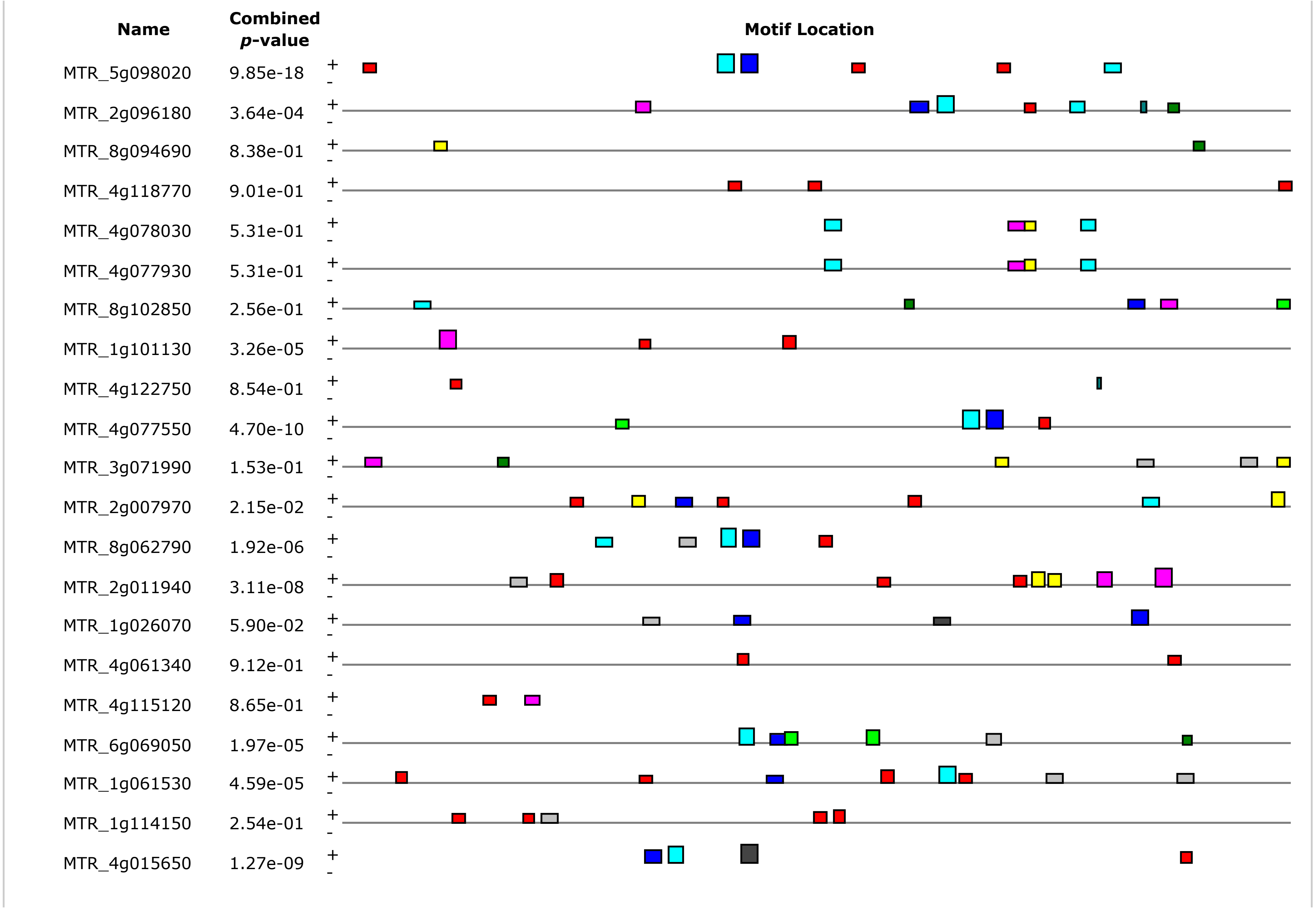
Name
Combined p-value
Motif Location
MTR_5g098020 9.85e-18
MTR_2g096180 3.64e-04
MTR_8g094690 8.38e-01
MTR_4g118770 9.01e-01
MTR_4g078030 5.31e-01
MTR_4g077930 5.31e-01
MTR_8g102850 2.56e-01
MTR_1g101130 3.26e-05
MTR_4g122750 8.54e-01
MTR_4g077550 4.70e-10
MTR_3g071990 1.53e-01
MTR_2g007970 2.15e-02
MTR_8g062790 1.92e-06
MTR_2g011940 3.11e-08
MTR_1g026070 5.90e-02
MTR_4g061340 9.12e-01
MTR_4g115120 8.65e-01
MTR_6g069050 1.97e-05
MTR_1g061530 4.59e-05
MTR_1g114150 2.54e-01
MTR_4g015650 1.27e-09

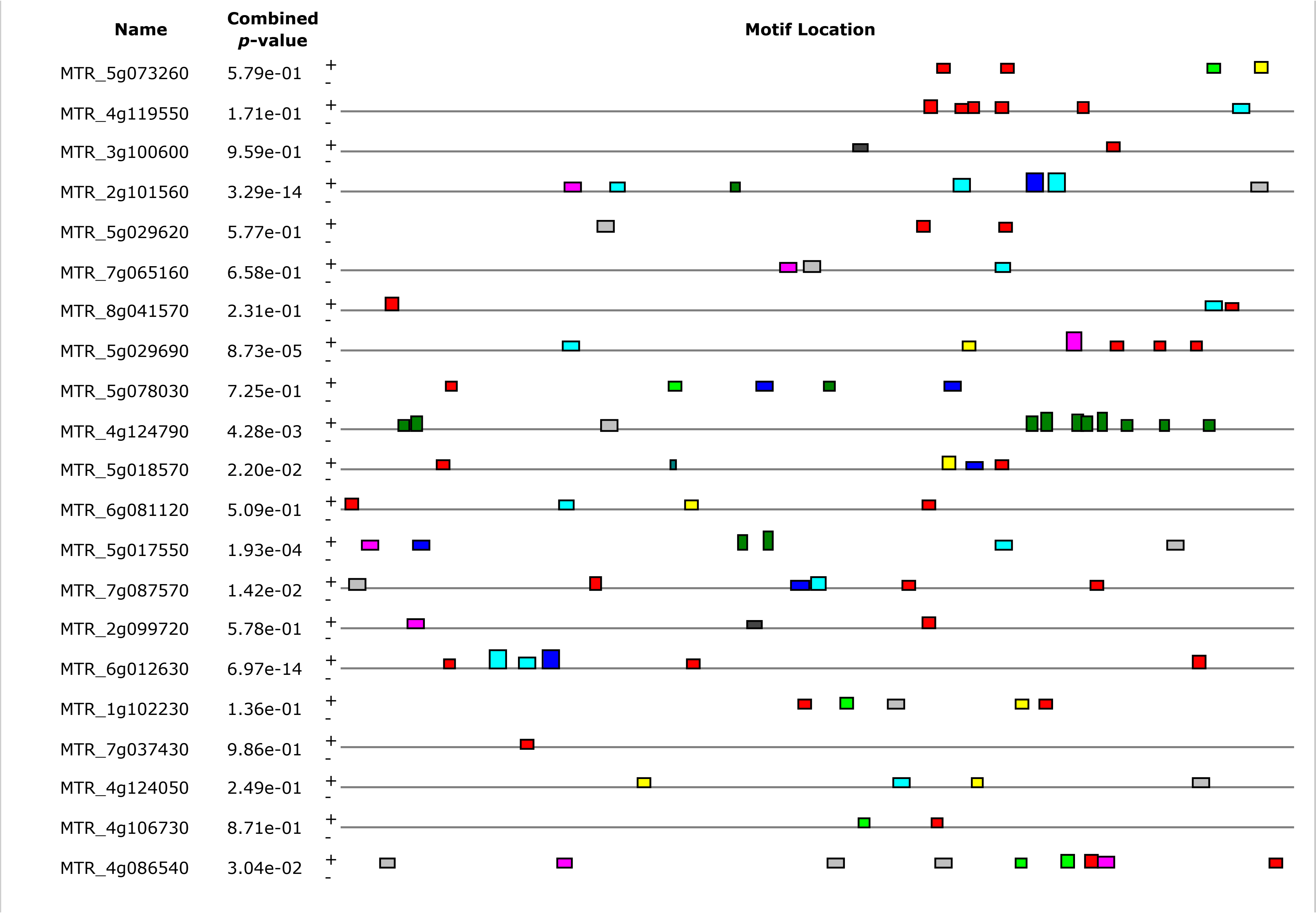
Name
Combined p-value
Motif Location
MTR_5g073260 5.79e-01
MTR_4g119550 1.71e-01
MTR_3g100600 9.59e-01
MTR_2g101560 3.29e-14
MTR_5g029620 5.77e-01
MTR_7g065160 6.58e-01
MTR_8g041570 2.31e-01
MTR_5g029690 8.73e-05
MTR_5g078030 7.25e-01
MTR_4g124790 4.28e-03
MTR_5g018570 2.20e-02
MTR_6g081120 5.09e-01
MTR_5g017550 1.93e-04
MTR_7g087570 1.42e-02
MTR_2g099720 5.78e-01
MTR_6g012630 6.97e-14
MTR_1g102230 1.36e-01
MTR_7g037430 9.86e-01
MTR_4g124050 2.49e-01
MTR_4g106730 8.71e-01
MTR_4g086540 3.04e-02

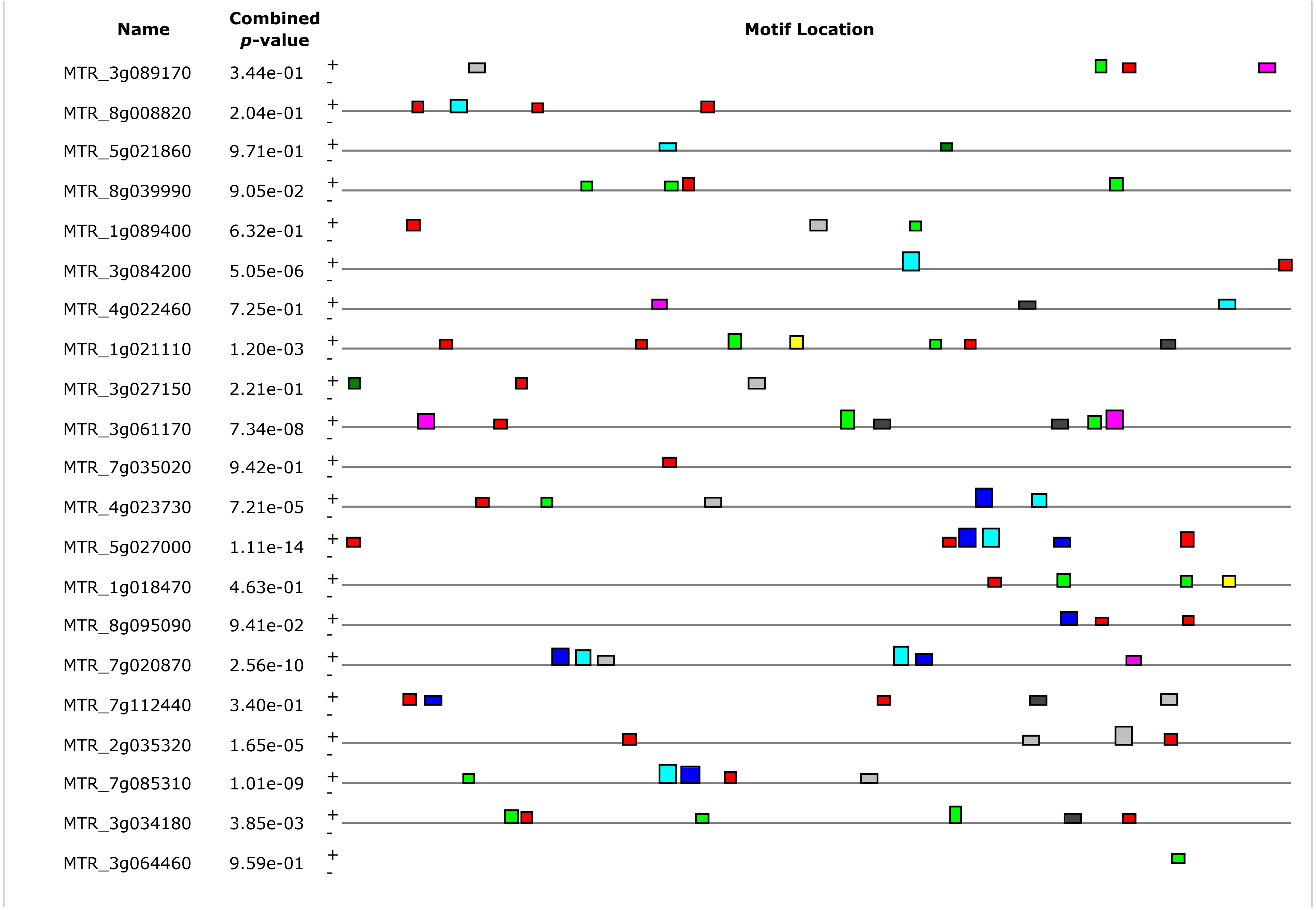
Name
Combined p-value
Motif Location
MTR_3g089170 3.44e-01
MTR_8g008820 2.04e-01
MTR_5g021860 9.71e-01
MTR_8g039990 9.05e-02
MTR_1g089400 6.32e-01
MTR_3g084200 5.05e-06
MTR_4g022460 7.25e-01
MTR_1g021110 1.20e-03
MTR_3g027150 2.21e-01
MTR_3g061170 7.34e-08
MTR_7g035020 9.42e-01
MTR_4g023730 7.21e-05
MTR_5g027000 1.11e-14
MTR_1g018470 4.63e-01
MTR_8g095090 9.41e-02
MTR_7g020870 2.56e-10
MTR_7g112440 3.40e-01
MTR_2g035320 1.65e-05
MTR_7g085310 1.01e-09
MTR_3g034180 3.85e-03
MTR_3g064460 9.59e-01

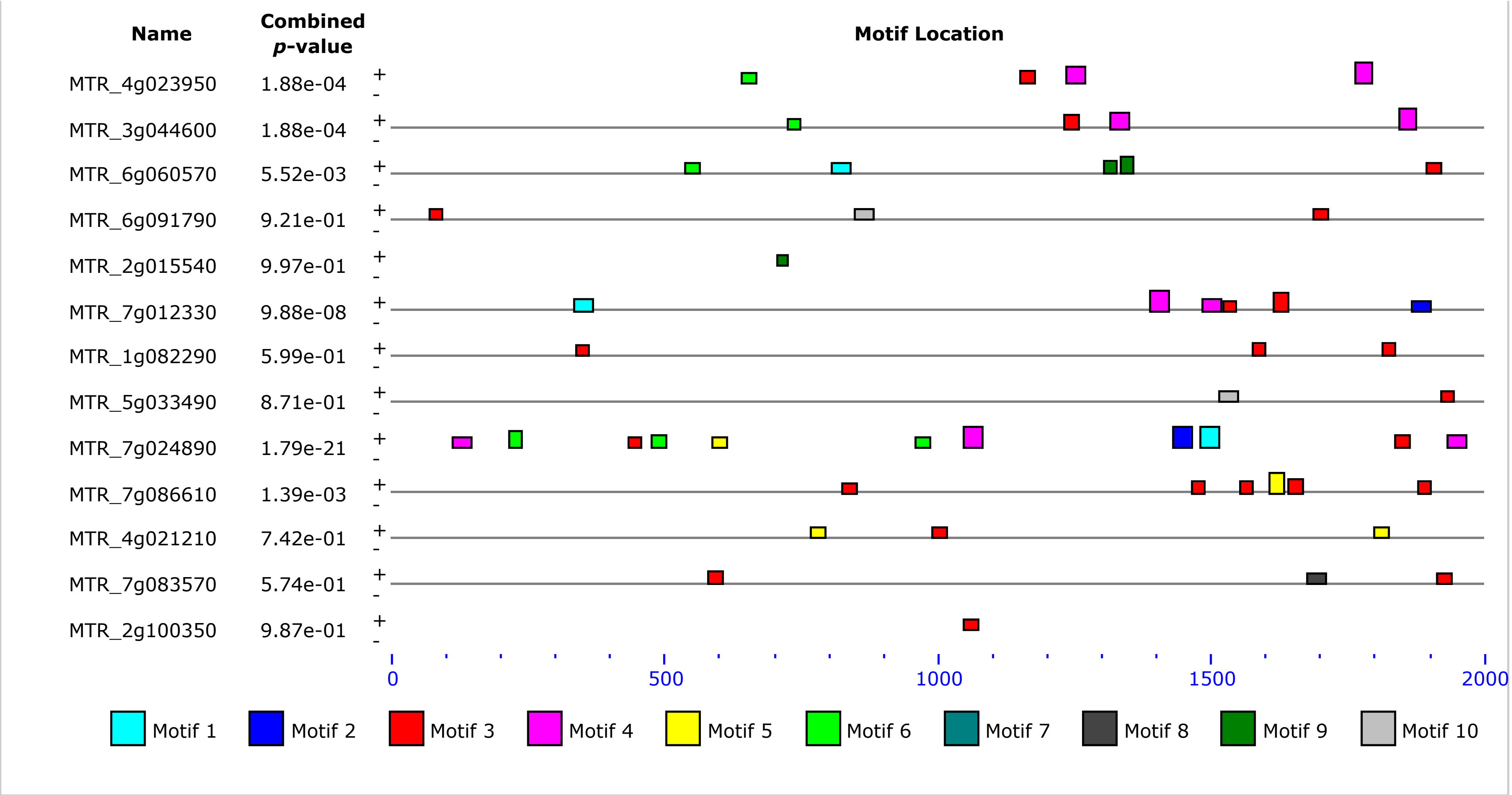

Top

MEME version

4.9.0 (Release date: Wed Oct 3 11:07:26 EST 2012)

Reference

Timothy L. Bailey and Charles Elkan, "Fitting a mixture model by expectation maximization to discover motifs in biopolymers", *Proceedings of the Second*

International Conference on Intelligent Systems for Molecular Biology, pp. 28-36, AAAI Press, Menlo Park, California, 1994.

show training set...

Command line summary

```
meme -dna -maxsize 431000 -nmotifs 10 -minw 6 -maxw 30 -revcomp -pal prom2k_MtrAMLCM.fa -o outrevpalAMLCM2kbmaxw30
```

Letter frequencies in dataset
A: 0.346   C: 0.154   G: 0.154   T: 0.346

Background letter frequencies (from dataset with add-one prior applied):
A: 0.346   C: 0.154   G: 0.154   T: 0.346

Stopping Reason

Stopped because nmotifs = 10 reached. Program ran on *rserv01.vital-it.ch*.

show model parameters...

EXPLANATION OF MEME RESULTS

Top

The MEME results consist of

- The **overview** of all discovered motifs.
- Information on each of the **motifs** MEME discovered, including:
  1. A **summary table** showing the width, number of contributing sites, log likelihood ratio, statistical significance, information content and relative entropy of the motif.
  2. A **sequence LOGO**.
  3. Downloadable **LOGO files** suitable for publication.
  4. A **regular expression** describing the motif.
  5. Some **further analysis** that can be performed on the motif.
  6. A list of **data formats** describing the motif.
  7. The **contributing sites** of the motif sorted by *p*-value and aligned with each other.
  8. The **block diagrams** of the contributing sites of the motif within each sequence in the training set.
- A **combined block diagram** showing an optimized (non-overlapping) tiling of all of the motifs onto each of the sequences in the training set.
- The **version** of MEME and the date it was released.
- The **reference** to cite if you use MEME in your research.
- A description of the **sequences** you submitted (the "training set") showing the name, "weight" and length of each sequence.

- The **command line summary** detailing the parameters with which you ran MEME.
- The reason why MEME **stopped** and the name of the CPU on which it ran.
- This **explanation** of how to interpret MEME results.

Motifs

For each motif that it discovers in the training set, MEME prints the following information:

Summary Table

This summary table gives the main attributes of the motif.

E-value
: The statistical significance of the motif. MEME usually finds the most statistically significant (low *E*-value) motifs first. The *E*-value of a motif is based on its log likelihood ratio, width, sites, the background letter frequencies (given in the command line summary), and the size of the training set. The *E*-value is an estimate of the expected number of motifs with the given log likelihood ratio (or higher), and with the same width and site count, that one would find in a similarly sized set of random sequences. (In random sequences each position is independent with letters chosen according to the background letter frequencies.)

Width
: The width of the motif. Each motif describes a pattern of a fixed with as no gaps are allowed in MEME motifs.

Sites
: The number of sites contributing to the construction of the motif.

Log Likelihood Ratio
: The log likelihood ratio of the motif.The log likelihood ratio is the logarithm of the ratio of the probability of the occurrences of the motif given the motif model (likelihood given the motif) versus their probability given the background model (likelihood given the null model). (Normally the background model is a 0-order Markov model using the background letter frequencies, but higher order Markov models may be specified via the **-bfile** option to MEME.)

Information Content
: The information content of the motif in bits. It is equal to the sum of the **uncorrected** information content, `R()`, in the columns of the LOGO. This is equal relative entropy of the motif relative to a uniform background frequency model.

Relative Entropy
: The relative entropy of the motif, computed in bits and relative to the background letter frequencies given in the command line summary. It is equal to the log-likelihood ratio (llr) divided by the number of contributing sites of the motif times `1/ln(2)`,

```
re = llr / (sites * ln(2)).
```

Sequence LOGO

MEME motifs are represented by position-specific probability matrices that specify the probability of each possible letter appearing at each possible position in an occurrence of the motif. These are displayed as "sequence LOGOS", containing stacks of letters at each position in the motif. The total height of the stack is the "information content" of that position in the motif in bits. The height of the individual letters in a stack is the probability of

the letter at that position multiplied by the total information content of the stack.

Note: The MEME LOGO differs from those produced by the Weblogo program because a **small-sample correction is NOT applied**. However, MEME LOGOs in PNG and encapsulated postscript (EPS) formats **with small-sample correction (SSC)** are available by clicking on the download button with "SSC" set to "on" under Download LOGO. The MEME LOGOs without small sample correction are similarly available. Error bars are included in the LOGOs with small-sample correction.

Modern web browsers supporting the canvas element and it's text manipulation functions as described in the html 5 standard, can render the sequence LOGOs without needing the images. The browsers which work with this feature are:

- Firefox 3.5 and above
- Safari 4 and above
- Google Chrome 4 and above

Unfortunately Internet Explorer 8 does not support any html 5 features.

The information content of each motif position is computed as described in the paper by Schneider and Stephens, "Sequence Logos: A New Way to Display Consensus Sequences" but **the small-sample correction,** e(n)**, is set to zero for the LOGO displayed in the MEME output.** The corrected information content of position i is given by

```
R(i) for amino acids   = log2(20) - (H(i) + e(n))   (1a)
R(i) for nucleic acids =    2    - (H(i) + e(n))    (1b)
```

where H(i) is the entropy of position i,

```
H(l) = - (Sum f(a,i) * log2[ f(a,i) ]).             (2)
```

Here, f(a,i) is the frequency of base or amino acid a at position i, and e(n) is the small-sample correction for an alignment of n letters. The height of letter a in column i is given by

```
height = f(a,i) * R(i)                              (3)
```

The approximation for the small-sample correction, e(n), is given by:

```
e(n) = (s-1) / (2 * ln(2) * n),                     (4)
```

where s is 4 for nucleotides, 20 for amino acids, and n is the number of sequences in the alignment.

The letters in the logos are colored as follows.
For DNA sequences, the letter categories contain one letter each.

NUCLEIC ACIDS	COLOR
A	RED
C	BLUE
G	ORANGE
T	GREEN

For proteins, the categories are based on the biochemical properties of the various amino acids.

AMINO ACIDS	COLOR	PROPERTIES
A, C, F, I, L, V, W and M	BLUE	Most hydrophobic[Kyte and Doolittle, 1982]
NQST	GREEN	Polar, non-charged, non-aliphatic residues
DE	MAGENTA	Acidic
KR	RED	Positively charged
H	PINK	
G	ORANGE	
P	YELLOW	
Y	TURQUOISE	

J. Kyte and R. Doolittle, 1982. "A Simple Method for Displaying the Hydropathic Character of a Protein", J. Mol Biol. 157, 105-132.

Note: the "text" output format of MEME preserves the historical MEME format where LOGOS are replaced by a simplified probability matrix, a relative entropy plot, and a multi-level consensus sequence.

Download LOGO

Logos can be generated on the fly by the meme webservice and you may specify a number of options to customize them to your needs. The options are:

Orientation
: Only valid for nucleotide motifs. Generate the standard view or the reverse complemented view of the motif.

SSC
: Use small sample correction and show errorbars on the image. Small sample correction is used by the Weblogo program.

Format
: The format of the generated image. If the image is to be used on the web then png is recommend. If the image is to be published then eps is recommended.

Width
: The width of the generated image in centimetres.

Height
: The height of the generated image in centimetres.

Regular Expression

This is a regular expression (RE) describing the motif. In each column, all letters with observed frequencies greater than 0.2 are shown; less-frequent letters are not included in the RE. MEME regular expressions are interpreted as follows: single letters match that letter; groups of letters in square brackets match any of the letters in the group. Regular expressions can be used for searching for the motif in sequences (using, for example, PatMatch) but the search accuracy will usually be better with the PSSM (using, for example MAST.)

Further Analysis

Either as a group or individually the motifs have a number of options for further analysis.

MAST
: Finds the best matching positions for a set of motifs in each sequence provided to it, ranked by the combined score of each sequence. For more information about MAST please read the introduction.

FIMO
: Finds all matches for a motif. For more information about FIMO please read the introduction.

TOMTOM
: Compares a single motif to a database of motifs. For more information about TOMTOM please read the introduction.

GOMO
: Identifies possible roles of DNA binding motifs. For more information about GOMO please read the introduction.

BLOCKS
: Submit to Blocks Multiple Alignment Processor where you can do several things like create phylogeny trees and search the blocks against a database of other blocks (protein only). For more information about BLOCKS Multiple Alignment Processor please visit the website.

Data Formats

The extracted data is avaliable in the following formats.

PSPM Format
: The motif itself is a position-specific probability matrix giving, for each position in the pattern, the observed frequency ("probability") of each possible letter. The probability matrix is printed "sideways"--columns correspond to the letters in the alphabet (in the same order as shown in the simplified motif) and rows corresponding to the positions of the motif, position one first. The motif is preceded by a line starting with "letter-probability matrix:" and containing the length of the alphabet, width of the motif, number of occurrences of the motif, and the *E*-value of the motif.

Note: Earlier versions of MEME gave the posterior probabilities--the probability after applying a prior on letter frequencies--rather than the observed frequencies. These versions of MEME also gave the number of *possible* positions for the motif rather than the actual number of occurrences. The output from these earlier versions of MEME can be distinguished by "n=" rather than "nsites=" in the line preceding the matrix.

PSSM Format

The position-specific scoring matrix corresponding to the motif is printed for use by database search programs such as MAST. This matrix is a log-odds matrix calculated by taking 100 times the log (base 2) of the ratio *p/f* at each position in the motif where *p* is the probability of a particular letter at that position in the motif, and *f* is the background frequency of the letter (given in the command line summary section.) This is the same matrix that is used above in computing the *p*-values of the occurrences of the motif in the Sites and Block Diagrams sections. The scoring matrix is printed "sideways"--columns correspond to the letters in the alphabet (in the same order as shown in the simplified motif) and rows corresponding to the positions of the motif, position one first. The scoring matrix is preceded by a line starting with "log-odds matrix:" and containing the length of the alphabet, width of the motif, number of characters in the training set, the scoring threshold (obsolete) and the motif *E*-value.

Note: The probability *p* used to compute the PSSM is *not* exactly the same as the corresponding value in the Position Specific Probability Matrix (PSPM). The values of *p* used to compute the PSSM take into account the motif prior, whereas the values in the PSPM are just the *observed* frequencies of letters in the motif sites.

BLOCKS Format

For use with BLOCKS tools.

FASTA Format

The FASTA format as described here.

Raw Format

Just the sites of the sequences that contributed to the motif. One site per line.

Sites

MEME displays the occurrences (sites) of the motif in the training set. The sites are shown aligned with each other, and the ten sequence positions preceding and following each site are also shown. Each site is identified by the name of the sequence where it occurs, the strand (if both strands of DNA sequences are being used), and the position in the sequence where the site begins. When the DNA strand is specified, '+' means the sequence in the training set, and '-' means the reverse complement of the training set sequence. (For '-' strands, the 'start' position is actually the position on the **positive** strand where the site ends.) The sites are **listed in order of increasing statistical significance** (*p*-value). The *p*-value of a site is computed from the the match score of the site with the position specific scoring matrix for the motif. The *p*-value gives the probability of a random string (generated from the background letter frequencies) having the same match score or higher. (This is referred to as the **position *p*-value** by the MAST algorithm.)

Block Diagrams

The occurrences of the motif in the training set sequences are shown as coloured blocks on a line. One diagram is printed for each sequence showing all the sites contributating to that motif in that sequence. The sequences are **listed in the same order as in the input** to make it easier to compare multiple block diagrams. Additionally the best *p*-value for the sequence/motif combination is listed though this may not be in ascending order as with

the sites. The *p*-value of an occurrence is the probability of a single random subsequence the length of the motif, generated according to the 0-order background model, having a score at least as high as the score of the occurrence. When the DNA strand is specified '+', it means the motif appears from left to right on the sequence, and '-' means the motif appears from right to left on the complementary strand. A sequence position scale is shown at the end of each table of block diagrams.

Combined Block Diagrams

The motif occurrences shown in the motif summary **may not be exactly the same as those reported in each motif section** because only motifs with a position *p*-value of 0.0001 that don't overlap other, more significant motif occurrences are shown.

See the documentation for MAST output for the definition of position and combined *p*-values.
